# Evaluating the genome and resistome of extensively drug-resistant *Klebsiella pneumoniae* using native DNA and RNA Nanopore sequencing

**DOI:** 10.1093/gigascience/giaa002

**Published:** 2020-02-04

**Authors:** Miranda E Pitt, Son H Nguyen, Tânia P S Duarte, Haotian Teng, Mark A T Blaskovich, Matthew A Cooper, Lachlan J M Coin

**Affiliations:** 1 Institute for Molecular Bioscience, The University of Queensland, 306 Carmody Road, Brisbane, Queensland, 4072, Australia; 2 The Peter Doherty Institute for Infection and Immunity, The University of Melbourne, 792 Elizabeth Street, Melbourne, Victoria, 3000, Australia

## Abstract

**Background:**

*Klebsiella pneumoniae* frequently harbours multidrug resistance, and current diagnostics struggle to rapidly identify appropriate antibiotics to treat these bacterial infections. The MinION device can sequence native DNA and RNA in real time, providing an opportunity to compare the utility of DNA and RNA for prediction of antibiotic susceptibility. However, the effectiveness of bacterial direct RNA sequencing and base-calling has not previously been investigated. This study interrogated the genome and transcriptome of 4 extensively drug-resistant (XDR) *K. pneumoniae* clinical isolates; however, further antimicrobial susceptibility testing identified 3 isolates as pandrug-resistant (PDR).

**Results:**

The majority of acquired resistance (≥75%) resided on plasmids including several megaplasmids (≥100 kb). DNA sequencing detected most resistance genes (≥70%) within 2 hours of sequencing. Neural network–based base-calling of direct RNA achieved up to 86% identity rate, although ≤23% of reads could be aligned. Direct RNA sequencing (with ∼6 times slower pore translocation) was able to identify (within 10 hours) ≥35% of resistance genes, including those associated with resistance to aminoglycosides, β-lactams, trimethoprim, and sulphonamide and also quinolones, rifampicin, fosfomycin, and phenicol in some isolates. Direct RNA sequencing also identified the presence of operons containing up to 3 resistance genes. Polymyxin-resistant isolates showed a heightened transcription of *phoPQ* (≥2-fold) and the *pmrHFIJKLM* operon (≥8-fold). Expression levels estimated from direct RNA sequencing displayed strong correlation (Pearson: 0.86) compared to quantitative real-time PCR across 11 resistance genes.

**Conclusion:**

Overall, MinION sequencing rapidly detected the XDR/PDR *K. pneumoniae* resistome, and direct RNA sequencing provided accurate estimation of expression levels of these genes.

## Introduction

*Klebsiella pneumoniae* is one of the leading causes of nosocomial infections, with reports of mortality rates as high as 50% [1–5]. This opportunistic pathogen commonly exhibits multidrug resistance, which severely limits treatment options [6]. A high abundance of resistance is frequently encoded on plasmids, accounting for the rapid global dissemination of resistance [1, 6]. Common therapeutic options for multidrug-resistant infections include carbapenems, fosfomycin, tigecycline, and polymyxins [7]. However, resistance is also rapidly developing against these antibiotics, resulting in the emergence of extensively drug-resistant (XDR) and subsequent pandrug-resistant (PDR) strains [6–9].

One of the major contributors to the advent of antibiotic resistance is the inability for current detection methodologies to readily and accurately assess bacterial infections, in particular, the resistance profile [10]. Rapid sequencing has been proposed as a way to determine antibiotic resistance, including approaches that use high-accuracy short reads, as well as those that exploit real-time single-molecule sequencing such as Oxford Nanopore Technologies (ONT). The ONT MinION platform is a portable single-molecule sequencer that can sequence long fragments of DNA and stream the sequence data for further data processing in real time, detecting the presence of bacterial species and acquired resistance genes [11–15]. Moreover, the long reads coupled with the ability to multiplex samples have immensely aided with the assembly of bacterial genomes [16–18]. This capability allows for the rapid determination of whether resistance is residing on the chromosome or plasmid(s). Of particular interest are high levels of resistance encoded on plasmids because these genes can rapidly be transferred throughout the bacterial population via horizontal gene transfer. However, a limitation of DNA sequencing is accurately identifying whether the presence of an acquired resistance gene or mutation is facilitating resistance.

ONT has recently released a direct RNA sequencing capability, which sequences native transcripts. Other sequencing technologies rely on fragmentation, complementary DNA (cDNA) conversion, and PCR steps that create experimental bias and hinder the accuracy of determining gene expression [19, 20]. The ability for MinION sequencing to read long fragments enables full-length transcripts to be investigated. To date, only a few direct RNA sequencing publications exist, which include eukaryote transcriptomes, primarily yeast (*Saccharomyces cerevisiae* [19, 21]) and recently, *Homo sapiens* [22]. This sequencing has additionally been implemented in viral transcriptomics [23–25]. Only 1 prior study by Smith et al. has applied this sequencing to bacterial 16S ribosomal RNA (rRNA) to detect RNA modifications [26]. Notably, resistance to certain antibiotics, such as aminoglycosides, can arise via RNA modifications, which cannot be detected once RNA is converted to cDNA [26]. Furthermore, library preparation time is halved for direct RNA sequencing owing to the absence of cDNA synthesis. Bacterial transcription differs significantly from that of eukaryotes in that transcription and translation occur simultaneously. As a result, bacterial messenger RNA (mRNA) transcripts lack poly(A) tails and alternative splicing; however, genes can be co-transcribed if regulated via an operon [27]. The poly(A) tail is critical for the library preparation for ONT sequencing; thus, we have established a methodology for adding this component onto transcripts.

In this study, we applied MinION sequencing to interrogate both the genome and the transcriptome (via direct RNA sequencing) for XDR *K. pneumoniae* clinical isolates. Of interest was to compare the potential for RNA sequencing to provide a better correlation to the resistance phenotype than DNA sequencing. These isolates have previously undergone “traditional” whole-genome sequencing (Illumina) and antimicrobial susceptibility testing [28]. An extended panel of antibiotics was tested in this study to identify PDR isolates. Three strains were selected from this cohort that exhibited resistance to all 24 antibiotics or antibiotic combinations tested, a high abundance of antibiotic resistance genes (≥26), and differing lineages (ST11 [16_GR_13], ST147 [1_GR_13], and ST258 [2_GR_12]). In addition, these isolates harbour polymyxin resistance, which is facilitated by a disruption in or upstream of *mgrB*. Variations in the *mgrB* gene result in increased expression of the *pmrCAB* and *pmrHFIJKLM* operon, enable the addition of phosphoethanolamine and/or 4-amino-4-deoxy-L-arabinose (Ara4N) to lipid A, and subsequently facilitate polymyxin resistance [29]. These pathways associated with polymyxin resistance were further explored using direct RNA sequencing and compared against a polymyxin-susceptible XDR isolate (ST258; 20_GR_12). This research aimed to assemble these genomes, discern expression of resistance genes, and ascertain the time required for detection. Furthermore, we sought to compare DNA and RNA sequencing as modalities for the rapid identification of acquired antibiotic resistance.

## Methods

### Bacterial strains and growth conditions

XDR *K. pneumoniae* clinical strains were sourced through the Hygeia General Hospital, Athens, Greece [28]. Antimicrobial susceptibility assays (Supplementary Table S1), sequence typing, and detection of acquired resistance genes have previously been determined [28]. Strains were stored at −80°C in 20% (v/v) glycerol, the identical stock was used as per the prior study, and the extended panel of antimicrobial susceptibility testing conducted similarly [28]. When required for extractions, glycerol stocks were grown on lysogeny broth agar and 6 morphologically similar colonies were selected for inoculation. The inoculum was grown in lysogeny broth overnight at 37°C shaking at 220 rpm. This overnight inoculum was used for both DNA and RNA extractions.

### High molecular weight DNA isolation

DNA was extracted from 10 mL of overnight culture using the DNeasy Blood and Tissue Kit (Qiagen: Chadstone, Victoria, Australia) according to manufacturer's guidelines, with the addition of an enzymatic lysis buffer pre-treatment (60 mg/mL lysozyme). Following the DNeasy extraction, high molecular weight (HMW) DNA was isolated using the MagAttract HMW DNA Kit (Qiagen: Chadstone, Victoria, Australia) in accordance with the manufacturer's instructions. An additional proteinase K treatment at 56°C for 10 min followed by supplementation of RNase A (1 mg) for 15 min at room temperature was included to increase DNA purity. Several direct extractions from bacterial overnight cultures using the HMW kit were performed; however, low DNA yield was observed and the initial DNeasy extraction was essential. An additional purification step following the HMW DNA extraction was critical for 2_GR_12 because carbohydrate contamination (260/230 ratio: ≤0.3) was identified potentially owing to a thickened capsule. This purification included the Monarch^®^ PCR & DNA Cleanup Kit (New England BioLabs: Notting Hill, Victoria, Australia) using the protocol to isolate fragments >2,000 bp.

### RNA extraction, mRNA enrichment, and poly(A) addition

The overnight inoculum was subcultured in 10 mL of cation-adjusted Mueller Hinton broth to reflect the media used for minimum inhibitory concentration (MIC) assays. Cultures were grown to mid-log phase (OD_600_ = 0.5–0.6). RNA was extracted via the PureLink^TM^ RNA Mini Kit (Thermo Fisher Scientific: Mulgrave, Victoria, Australia) in accordance with the manufacturer's protocols, which included using Homogenizer columns (Thermo Fisher Scientific: Mulgrave, Victoria, Australia). To remove DNA contamination, the TURBO DNA-free^TM^ kit (Thermo Fisher Scientific: Mulgrave, Victoria, Australia) was implemented. A minor adjustment was an increased concentration of TURBO DNase (4 U) incubated at 37°C for 30 min. The RNeasy Mini Kit (Qiagen: Chadstone, Victoria, Australia) clean-up protocol was used to purify and concentrate RNA samples. The rRNA was depleted via the MICROB*Express*^TM^ Bacterial mRNA Enrichment Kit (Thermo Fisher Scientific: Mulgrave, Victoria, Australia). Minor protocol changes included adding ≥2 µg of DNA-depleted RNA, and the enriched mRNA was precipitated for 3 h at −20°C. Poly(A) addition was performed using the Poly(A) Polymerase Tailing Kit (Astral Scientific: Gymea, New South Wales, Australia) in accordance with the manufacturer's alternative protocol (4 U input of Poly(A) Polymerase). The input RNA concentration was ≥800 ng, and RNA samples were incubated at 37°C for 1 hour. Poly(A)^+^ RNA was purified using Agencourt AmpureXP (Beckman Coulter Australia: Gladesville, New South Wales, Australia) beads (1:1 ratio).

### Extraction quality control

DNA and RNA were quantitated using Qubit®2.0 (Thermo Fisher Scientific: Mulgrave, Victoria, Australia) and purity determined with a NanoDrop 1000 Spectrophotometer (Thermo Fisher Scientific: Mulgrave, Victoria, Australia). DNA fragment sizes were measured using the Genomic DNA ScreenTape & Reagents (Integrated Sciences: Willoughby, New South Wales, Australia), and sizes from 200 to >60,000 bp were analysed on a 4200 TapeStation System (Integrated Sciences: Willoughby, New South Wales, Australia) (Supplementary Fig. S1). RNA fragment size was checked using an Agilent RNA 6000 Pico kit and run on a 2100 Bioanalyzer (Agilent Technologies: Mulgrave, Victoria, Australia) for the initial RNA extract (RIN: ≥8.5), after rRNA depletion and after poly(A) tailing (Supplementary Fig. S2).

### ONT library preparation and sequencing

RNA libraries (≥600 ng poly(A)^+^ RNA) were prepared using the Direct RNA Sequencing kit (SQK-RNA001). The Rapid Barcoding Sequencing kit (SQK-RBK001) was used for HMW DNA samples (1_GR_13, 16_GR_13, 20_GR_12; 300 ng input each). Isolate 2_GR_12 (300 ng input) was prepared separately using the Rapid Sequencing Kit (SQK-RAD003). Libraries were sequenced with MinION R9.4 flowcells, and the raw data (fast5 files) were base-called using Albacore 2.1.1 for DNA sequencing (Supplementary Fig. S3). Reagents and flowcells were sourced through ONT (Littlemore, Oxford, United Kingdom). For benchmarking purposes, RNA reads were additionally base-called with Albacore 2.2.7, Guppy 3.0.3, and the Chiron v0.5 [30] RNA base-caller, which was trained in-house [31].

### Real-time resistome detection emulation

The real-time emulation was performed after sequencing, and the time required to detect antibiotic resistance was determined as previously described [14]. Briefly, this pipeline aligns Albacore base-called reads via BWA-MEM (BWA, RRID:SCR_010910) [32] to an antibiotic resistance gene database. Antibiotic resistance genes were obtained from the ResFinder 3.0 database [33]. This dataset comprises of 2,131 genes, which were clustered on the basis of 90% identity to form 611 groups or gene families. The detection of false-positive results is reduced using the multiple sequence alignment software kalign2 [34], a probabilistic finite state machine [35], and once the alignment score reached a threshold, the resistance gene was reported.

### Assembly of genomes

To assemble genomes with both Illumina and ONT reads, SPAdes v3.10.1 (SPAdes, RRID:SCR_000131) [36] was used. Hybrid assemblers included npScarf [37] and Unicycler v0.3.1 [38]. Assemblers using only ONT reads included Canu v1.5 (excluding reads <500 bp) (Canu, RRID:SCR_015880) [39] and the combination of Minimap2 v2.1-r311 and Miniasm v0.2-r168-dirty; Racon (git commit 834,442) was used in both cases to polish the assemblies [40, 41]. Consensus sequences were determined using Mauve (snapshot_2015–02-13) to construct the final assembly (Mauve, RRID:SCR_012852) [42]. The output from each assembly software package is reported in Supplementary Table S2. Genomes were annotated using the Rapid Annotation using Subsystem Technology (RAST), which also provided a list of virulence genes [43]. The locations of acquired antibiotic resistance genes were determined using ResFinder 3.0 [33], and plasmids were identified via PlasmidFinder 1.3 [44]. To discern whether plasmid sequences have previously been reported, contigs underwent a BLASTn analysis against the NCBI database [45] (BLASTN, RRID:SCR_001598).

### RNA alignment and expression profiling

Base-called RNA reads were converted to DNA (uracil bases changed to thymine) and aligned using BWA-MEM [32] to the updated genome assemblies. BWA-MEM was selected owing to shorter transcripts being produced by bacteria (Supplementary Fig. S3) and the lack of introns and alternative splicing. Similar parameters to the BWA-MEM ont2d function were used, but seed length was reduced (-k 14) to compensate for shorter reads (-k 11 [minimum seed length, bp] -W20 [bandwidth] -r10 [gap extension penalty] -A1 [match score] -B1 [mismatch penalty] -O1 [Gap open penalty] -E1 [Gap extension penalty] -L0 [Clipping penalty]). Multi-mapping reads were removed via SAMtools (secondary alignment: flagged as 256) [46], and BEDTools coverage (BEDTools, RRID:SCR_006646) [47] was used to ascertain the expression of resistance genes in counts per million (cpm) mapped reads (after removing reads mapping to rRNA). BEDTools intersect [47] was used to identify potential operons and co-expression of genes. To compare against qRT-PCR results, read counts were normalized to the housekeeping gene, *rpsL* [48]. Read alignments were further visualized using Integrative genomics viewer 2.3.59 [49].

### Whole-transcriptome gene expression and estimation of expression confidence intervals

We identified genes that were differentially expressed in 1 sample (vs all remaining samples) using a quasi-likelihood F-test in EdgeR (edgeR, RRID:SCR_012802) [50] with a false discovery rate threshold of 0.01. Expression levels (in cpm) were extracted for every significant gene in any 1 of these 1-vs-remaining differential expression analyses to generate an expression heat map. The expression heat map is based on the log_10_(cpm) for each of these genes. To estimate the 90% confidence intervals (CIs) in cpm estimates from direct RNA sequence data, we assumed that the observed counts were generated from a binomial distribution with unobserved probability of success (*p*). We estimate the fifth and 95th percentiles from a β-distribution with shape parameters equal to the number of reads mapped to a given gene (α) and the number of reads mapped elsewhere (β) plus a pseudo-count of 0.1. The 90% CI is calculated as the difference between the expression levels at the fifth and 95th percentile.

### Quantitative real-time reverse transcriptase PCR (qRT-PCR)

First strand cDNA synthesis was performed on 1 µg of total DNA-depleted RNA using SuperScript III (Thermo Fisher Scientific: Mulgrave, Victoria, Australia). Primers used are displayed in Supplementary Table S3. Samples were prepared in triplicate via the SYBR Select Master Mix (Thermo Fisher Scientific: Mulgrave, Victoria, Australia) and expression detected using a ViiA 7 Real-time PCR system (Thermo Fisher Scientific: Mulgrave, Victoria, Australia). Cycling conditions were as follows: hold 50°C (2 min), 95°C (2 min) followed by 50 cycles of 95°C (15 sec), 55°C (1 min). A melt curve was included to determine the specificity of the amplification and a no template control to detect contamination or primer dimers. Results were analysed with QuantStudioTM Real-Time PCR Software, and triplicates were averaged, normalized to the housekeeping gene *rpsL* [48] and relative expression determined via the 2*^−^^ΔΔCT^* method [51].

## Results

### Antibiotic resistance and the location of acquired resistance in the genome

This study assayed 9 additional antibiotics or antibiotic combinations to further characterize the phenotypic resistance of these isolates (Supplementary Table S1). Strains 1_GR_13, 2_GR_12, and 16_GR_13 were non-susceptible to all antibiotics including the 24 antibiotics tested previously [28]. 20_GR_12 was only susceptible to gentamicin and polymyxins.

MinION DNA sequencing for all isolates was run for ≥20 hours, which generated 1.19 Gb (215×) for 1_GR_13, 0.39 Gb (67×) for 2_GR_12, 0.56 Gb (101×) for 16_GR_13, and 0.64 Gb (115×) for 20_GR_12 (Supplementary Table S2). Across the differing assembly tools, the chromosome sequence commonly circularized as a 5.0–5.4 Mb contig including plasmids ranging between 13 and 193 kb with the exception of 2_GR_12. Aligning ONT reads to the final assembly revealed that DNA sequencing had 90% accuracy across isolates.

Using the capacity for MinION sequencing to read long fragments of DNA, the locations of antibiotic resistance genes were clearly resolved (Table 1). All genomes were circular except for 2_GR_12, where 3 plasmids remained linear. This was partly due to difficulties extracting DNA, not retaining long fragments, and subsequently, lower coverage of the genome (Supplementary Fig. S1, Table S2). Amongst the 4 isolates, the chromosome size ranged between 5.1 and 5.5 Mb, which encoded resistance genes *blaSHV-11, fosA*, and *oqxAB*. Most resistance (≥75%) mapped to plasmids.

**Table 1:** Final assembly of XDR *K. pneumoniae* isolates and location of antibiotic resistance genes

Isolate	ST	Contig	Length (bp)	Coverage	Contig ID*	Resistance genes**
1_GR_13	147	1	**5,181,675**	1	C	*blaSHV-11, fosA, oqxA, oqxB*
		2	**192,771**	1.95	P: IncA/C2	*aadA1, ant(2'')-Ia, aph(6)-Id, ARR-2, blaOXA-10, blaTEM-1B, blaVEB-1, cmlA1, dfrA14, dfrA23, rmtB, strA, sul1, sul2, tet(A), tet(G)*
		3	**168,873**	2	P: IncFIB_pKpn3_, IncFII_pKP91_	*aadA24, aph(3')-Ia, aph(6)-Id, dfrA1, dfrA14, strA*
		4	**108,879**	1.53	P: IncFIB_pKPHS1_	
		5	**55,018**	14.10		
		6	**53,495**	2.36	P: IncR, IncN	*aadA24, aph(3')-Ia, aph(6)-Id, blaVIM-27, dfrA1, mph(A), strA, sul1*
2_GR_12	258	1	**5,466,424**	1	C	*blaSHV-11, fosA, oqxA, oqxB*
		2	197,872	1.3	P: IncFIB_pKpn3_, IncFIIK	*aadA2, aph(3')-Ia, catA1, dfrA12, mph(A), sul1*
		3	175,636	1.49	P: IncA/C2	*aadA1, ant(2'')-Ia, aph(3'')-Ib, aph(6)-Id, ARR-2, blaOXA-10, blaTEM-1A, blaVEB-1, cmlA1, dfrA14, dfrA23, rmtB, sul1, sul2, tet(A), tet(G)*
		4	95,481	1.61	P: IncFIB_pQil_	*blaKPC-2, blaOXA-9, blaTEM-1A*
		5	**43,380**	1.91	P: IncX3	*blaSHV-12*
		6	**13,841**	4	P: ColRNAI	*aac(6')-Ib, aac(6')Ib-cr*
16_GR_13	11	1	**5,426,917**	1	C	*blaSHV-11, fosA, oqxA, oqxB*
		2	**187,670**	0.88	P: IncFIB_pKpn3_; IncFIIK	*aac(3)-IIa, aac(6')Ib-cr, aadA2, aph(3')-Ia, blaCTX-M-15, blaOXA-1, catB4, dfrA12, mph(A), sul1*
		3	**155,161**	0.99	P: IncA/C2	*aadA1, ant(2'')-Ia, aph(3'')-Ib, aph(6)-Id, ARR-2, blaOXA-10, blaTEM-1B, blaVEB-1, cmlA1, rmtB, sul1, sul2, tet(A), tet(G)*
		4	**63,589**	1.49	P: IncL/M_pOXA-48_	*blaOXA-48*
		5	**5,234**	188.49		
		6	**4,940**	97.77	P: ColRNAI	
20_GR_12	258	1	**5,395,894**	1	C	*blaSHV-11, fosA, oqxA, oqxB*
		2	**170,467**	1.77	P: IncFIB_pKpn3_; IncFIIK	*aph(3')-Ia, blaKPC-2, blaOXA-9, blaTEM-1A*
		3	**50,979**	1.42	P: IncN	*aph(3'')-Ib, aph(6)-Id, blaTEM-1A, dfrA14, sul2, tet(A)*
		4	**43,380**	1.78	P: IncX3	*blaSHV-12*
		5	**13,841**	10.82	P: ColRNAI	*aac(6')-Ib, aac(6')Ib-cr*

* Contig ID represents chromosome (C) or plasmid (P): replicon determined via PlasmidFinder 1.3.

** Resistance genes identified using ResFinder 3.0 (≥90% sequence similarity, ≥60% minimum length) and displayed in alphabetical order. Boldface indicates a circular contig.

At least 1 megaplasmid, defined as a plasmid >100 kb, was detected in all isolates (Table 1). These commonly harboured the replicon IncA/C2 or InFIB and IncFIIK. The IncA/C2 plasmid was present in all samples except 20_GR_12. This plasmid contained up to 16 resistance genes, which conferred resistance towards aminoglycosides, β-lactams, phenicols, rifampicin, sulphonamides, tetracyclines, and trimethoprim, with the exception of 16_GR_13. Isolate 16_GR_13 lacked trimethoprim resistance on its IncA/C2 plasmid. The plasmids containing both replicons IncFIB and IncFIIK differed vastly between all 4 replicates. All contained IncFIB_pKpn3_ and IncFIIK; however, 1_GR_13 differed with IncFII_pKP91_. Additionally, a differing IncFIB replicon was detected on a separate contig in 1_GR_13 (pKPHS1) and 2_GR_12 (pQil). The only instance where another dual replicon was identified was in 1_GR_13, which harboured both IncR and IncN. This plasmid contained aminoglycoside, β-lactam, trimethoprim, macrolide, and sulphonamide resistance. 1_GR_13 also contained a 5.5-kb circular contig that was annotated as a phage genome. Various regions of these megaplasmids were unique to these isolates compared to prior sequences deposited in NCBI (Supplementary Table S5).

The ColRNAI plasmid was present in all except 1_GR_13, which encoded aminoglycoside and quinolone resistance (*aac(6')-Ib, aac(6')-Ib-cr)* (Table 1). The ColRNAI plasmid in 2_GR_12 and 20_GR_12 was 13,841 bp in size and shared 75% similarity between the 2 isolates. This plasmid differed in 16_GR_13, which contained no resistance genes and 35% the size. The same IncX3 plasmid (43,380 bp) was apparent in isolates 2_GR_12 and 20_GR_12. Unique to 16_GR_13 was the IncL/M_pOXA-48_ plasmid containing *blaOXA-48* and the 50,979 bp IncN plasmid in 20_GR_12 with resistance against 5 classes (aminoglycoside (*aph(3'')-Ib, aph(6)-Id*), β-lactam (*blaTEM-1A*), sulphonamide (*sul2*), tetracycline (*tet(A)*), trimethoprim (*dfrA14*)) of antibiotics.

Multiple copies of acquired resistance genes were apparent across plasmids in several isolates. For 1_GR_13, up to 3 copies were present of genes *aadA24, aph(3')-Ia, aph(6)-Id, dfrA1, dfrA14, strA*, and *sul1* (Table 1). In 2_GR_12, *sul1* and *blaTEM-1A* were duplicated and for 16_GR_13, only *sul1* was represented twice.

### Real-time detection emulation of resistance genes via DNA sequencing

**Figure 1:**
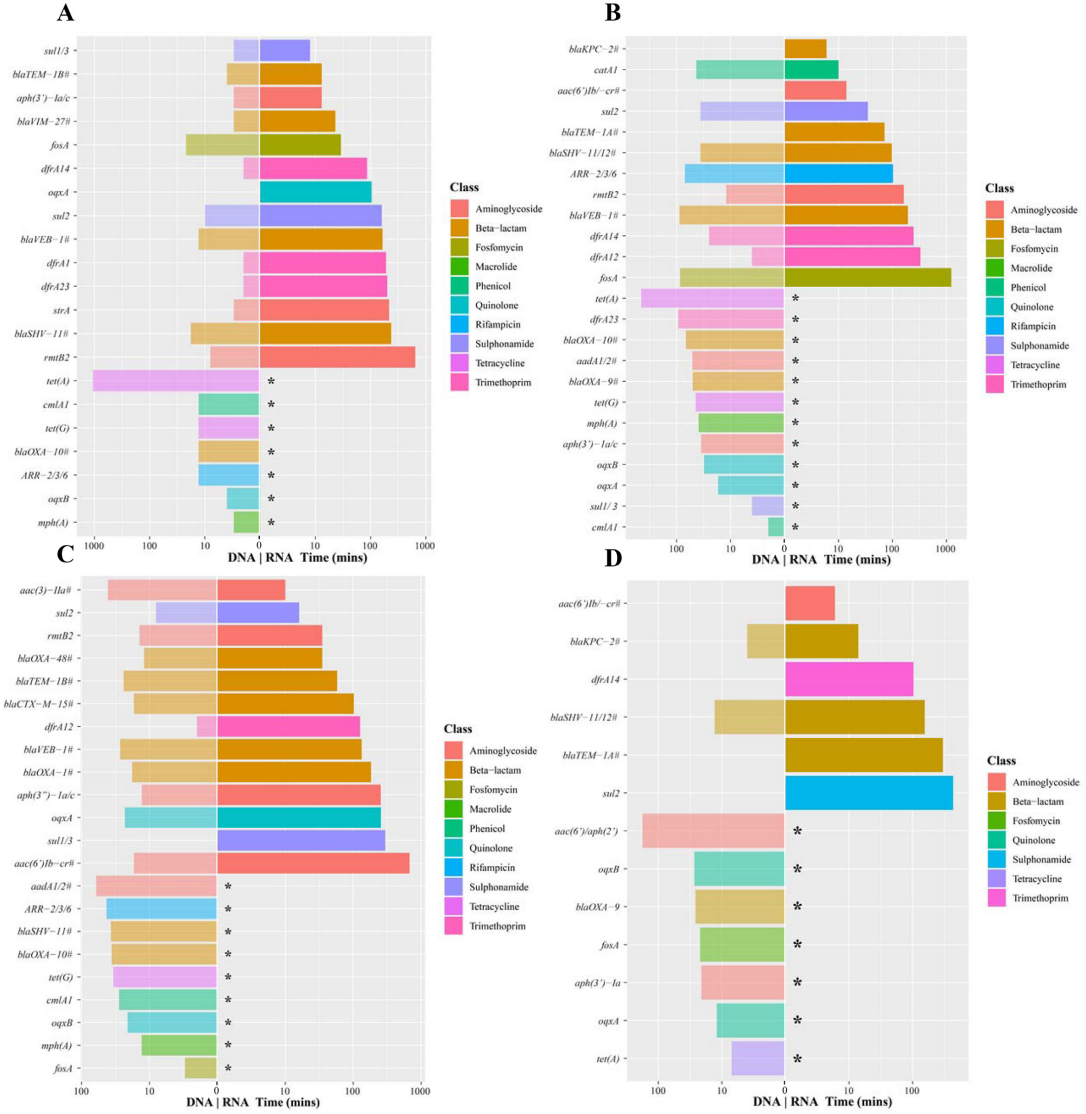
Time required to detect antibiotic resistance genes via the real-time emulation analysis using MinION native DNA and RNA sequencing. (**A**) 1_GR_13, (**B**) 2_GR_12, (**C**) 16_GR_13, and (**D**) 20_GR_12. Legend colours identify the class of antibiotic to which the gene confers resistance, a slash on the y-axis label indicates reads that detected >1 resistance gene, and the pound sign indicates a family of genes detected (>3). An asterisk indicates the inability for direct RNA sequencing to detect this gene. Albacore 2.2.7. base-called sequences were used, and all reads (pass and fail) were included in this analysis.

Most (≥70%) of resistance genes were detected via DNA sequencing within the first 2 hours (Fig. 1, Supplementary Table S5). These genes confer resistance towards aminoglycosides, β-lactams, fosfomycin, macrolides, phenicols, quinolones, rifampicin, sulphonamides, tetracyclines, and trimethoprim. 20_GR_12 lacked acquired resistance genes for macrolides, phenicols, and rifampicin; however, all other classes were detected within 2 hours. All isolates, except 2_GR_12, were sequenced for 21 hours, which was sufficient to obtain the complete genome assembly. Only a few additional genes were detected after the first 10 hours across isolates (Supplementary Table S5). For 2_GR_12, an extended run of 41 hours detected no further genes after 20 hours. Overall, the presence of these resistance genes corresponded to a resistant phenotype towards aminoglycosides, β-lactams, fosfomycin, phenicols, quinolones, sulphonamides (sulfamethoxazole), tetracyclines, and trimethoprim (Supplementary Table S1). Because macrolides and rifampicin are not routinely used to treat *K. pneumoniae* infections, no breakpoints exist according to CLSI and EUCAST guidelines; however, all isolates exhibit an MIC ≥128 µg/mL towards erythromycin (macrolide) and ≥64 µg/mL for rifampicin (Supplementary Table S1).

After 2 hours of sequencing, several genes not observed in the final assembly via ResFinder 3.0 were detected (Supplementary Table S5). These were predominantly genes attributed to aminoglycoside, β-lactam, rifampicin, and phenicol resistance. Furthermore, resistance genes to additional classes were detected including fusidic acid and vancomycin. This was evident in 2_GR_12 (*fusB*) and 16_GR_13 (*fusB, vanR*). However, these genes had <30 reads and their phred-scale mapping quality (MAPQ) scores were <10 (misplaced probability >0.1). Furthermore, the majority of genes not observed in the final assembly nor observed in Illumina data exhibited a MAPQ score of ≤10, which may indicate that a more stringent threshold is required to negate false-positive results. However, if this threshold increases, true-positive results would not be detected including *aadA1, aadA2*, and *ARR-2* in 2_GR_12 and *blaOXA-48, blaCTX-M-15*, and *ARR-2* in 16_GR_13.

Several genes found in the final assembly were not detected in the real-time emulation analysis (Supplementary Table S5). This was mainly observed for aminoglycoside resistance encoding genes. For 1_GR_13, this included *aadA1, ant(2'')-Ia, aph(6)-Id*, and *aadA24*. Similarly, 2_GR_12 and 20_GR_12 lacked *aph(3'')-Ib* and *aph(6)-Id*. 2_GR_12 additionally had the absence of *ant(2'')-Ia*. Detection of *ant(2'')-Ia, aph(3'')-Ib*, and *aph(6)-Id* was not present in 16_GR_13. 16_GR_13 further lacked *catB4* (phenicol) and *tet(A)* (tetracycline). Various phenicol resistance genes were reported in the real-time emulation; however, the incorrect gene was identified, which may represent sequencing errors accumulated over time and high similarity to other phenicol resistance genes. The tetracycline resistance gene, *tet(A)*, was interestingly not reported in this emulation with 190 reads and the majority of reads exhibiting a high mapping confidence (MAPQ = 60, equivalent to an error probability of 1 × 10^−6^). This gene was only detected after 10 hours for 1_GR_13 and 2_GR_12, and this result may be influenced by the presence of only 1 copy of *tet(A)* encoded on a low copy number megaplasmid (between 1 and 1.5; see Table 1).

### Direct RNA sequencing resistance detection

The time required to detect resistance was further interrogated using RNA sequencing. Rapid detection was possible for several resistance genes via direct RNA sequencing (Fig. 1). This was evident for genes conferring resistance to aminoglycosides, β-lactams, sulphonamides, and trimethoprim for all 4 isolates. Resistance towards these antibiotics was commonly detected within 6 hours. In some instances, quinolone, rifampicin, fosfomycin, and phenicol resistance was detected. A similar result was obtained whether all reads or passed reads alone were analysed. The most significant difference when analysing all reads was the detection of *fosA* in 1_GR_13 and *ARR-2* and *fosA* in 2_GR_12. Consistently absent from this analysis were genes attributed to macrolide (*mph(A)*) and tetracycline (*tet(A), tet(G)*) resistance; however, isolates exhibited high levels of resistance to tetracycline (>64 µg/mL) (Supplementary Table S1). Commonly no new genes were detected after 12 hours of sequencing except for *fosA* in 2_GR_12. Although *fosA* was detected when the failed reads were included, a low MAPQ score (≤10) was apparent. Similar to the DNA real-time detection, several genes not found in the final assembly were identified (Supplementary Table S5). With the exception of 20_GR_12, this included *aadB* and *strB* for all isolates. Additional genes detected included *ARR-7* in 1_GR_13, *strA* in 2_GR_12, and for 16_GR_13, *blaCTX-M-64, blaOXA-436*, and *strA*. Similar genes or gene families were identified when DNA and direct RNA sequencing were compared. Overall, genes were detected more readily via DNA sequencing; however, there were a few instances in which RNA sequencing detected resistance quicker: *aac(3’)-IIa* in 16_GR_13 and *sul2* and *catA1* in 2_GR_12. Similar results were observed when data yield rather than time was investigated, which compensates for the slower translocation speed associated with direct RNA sequencing (Supplementary Fig. S4).

### Levels of expression of resistance genes

RNA sequencing accumulated over ∼40 hours yielded between 0.9 and 1.7 million reads for these isolates (Supplementary Fig. S3). However, only a low proportion (972,436 to 1,725,702 reads [≤14.64%]) of these reads passed base-calling (sequencing quality score ≥7) using Albacore 2.2.7 (Supplementary Table S6). Aligning passed reads alone to the final assembly (ensuring the removal of the poly(A) tail and reads <75 nt), ≥98% (1_GR_13: 95,591; 2_GR_12: 138,214; 16_GR_13: 227,781; 20_GR_12: 119,425) of reads were mappable; however, ≤46% (1_GR_13: 42,654; 2_GR_12: 46,787; 16_GR_13: 79,175; 20_GR_12: 54,986) of these had a MAPQ score ≥10. When failed reads were aligned, the majority were not mappable to the reference genome (≥0.76 million reads, ≥91.50%) and commonly exhibited a low MAPQ score (≤10). Low mapping quality could be attributed to assignment of reads to multiple copies of genes in the genome. Furthermore, the ONT error rates could lead to misassignment of reads to genes. In light of this, we decided to benchmark a number of different base-callers, including Albacore 2.2.7 and Guppy 3.03, as well as Chiron v0.5, which was trained in-house (Supplementary Table S6, Fig. S5). Chiron base-called more reads compared to Albacore 2.2.7 and Guppy 3.0.3; however, fewer reads aligned to the reference genome and there was a slightly lower identity rate. Albacore 2.2.7 had the highest average accuracy across isolates (84.87%), closely followed by Guppy 3.0.3 (84.62%) and then Chiron v0.5 (78.19%) (Supplementary Table S6). However, low alignment rates could be attributed to the addition of a long artificial poly(A), which was identified to be ∼400–700 bp across isolates (Supplementary Fig. S6). Taking into consideration the Albacore 2.2.7 base-called reads, a proportion of these reads were found to map to rRNA including 1_GR_13 (18%), 2_GR_12 (37%), 16_GR_13 (24%), and 20_GR_12 (23%). Overall, ≥58% of genes (with ≥1 read mapping to the gene) were identified to be expressed across isolates 1_GR_13 (68%), 2_GR_12 (58%), 16_GR_13 (75%), and 20_GR_12 (69%).

**Figure 2:**
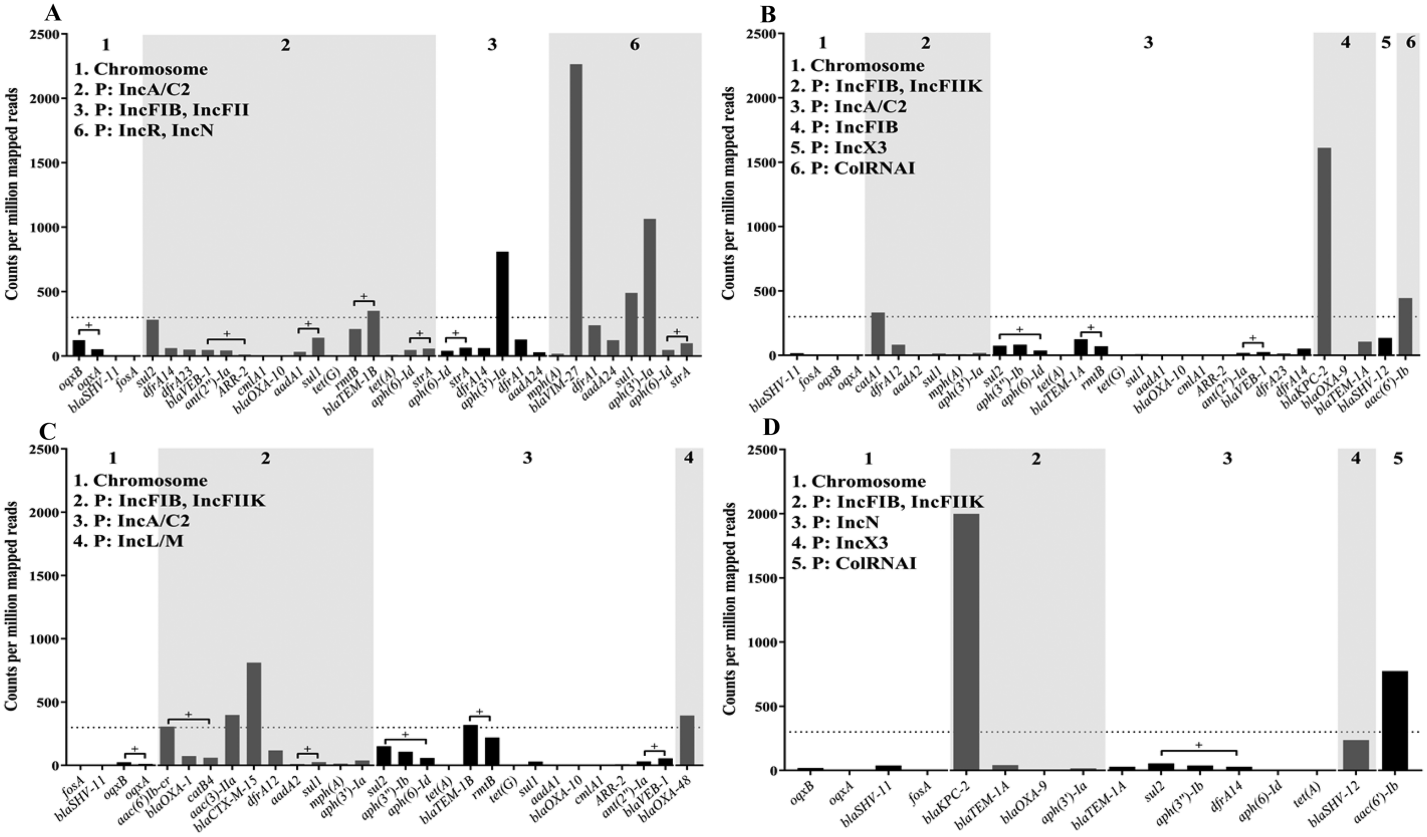
Expression of resistance genes via direct RNA sequencing when aligned to completed genomes. (**A**) 1_GR_13, (**B**) 2_GR_12, (**C**) 16_GR_13, and (**D**) 20_GR_12. The x-axis depicts the resistance genes, which are grouped on the basis of the location in the genome, where P indicates a plasmid followed by replicon identity. Albacore 2.2.7 base-called pass and fail reads were used for analysis. Values indicate counts per million mapped reads (cpm) (after removal of reads mapping to rRNA), and dotted line is set to 300 cpm. Genes are represented in order of appearance on contig, and plus sign indicates the co-expression of genes.

Amongst the 4 isolates, levels of expression for resistance genes on the chromosome (*blaSHV-11, fosA*, and *oqxAB*) were low (≤122 counts per million mapped reads) (Fig. 2). The remaining resistance genes were located on plasmids. Resistance genes exhibiting high levels of expression (300 cpm) were apparent in 1_GR_13 (*blaTEM-1B, blaVIM-27, sul1, aph(3’)-Ia*), 2_GR_12 (*aac(6’)-Ib, catA1, blaKPC-2*), 16_GR_13 (*aac(6’)Ib-cr, aac(3)-IIa, blaCTX-M-15, blaTEM-1B, blaOXA-48*), and 20_GR_12 (*blaKPC-2, aac(6’)Ib*). Counts for *aac(6’)-1b* and *aac(6’)-1b-cr* in 2_GR_12 and 20_GR_12 were grouped. The gene *aac(6’)-1b-cr* is a shortened version of *aac(6’)-1b*, and both were identified in the same genome position; hence, only *aac(6’)-1b* is displayed in Fig. 2. Expression estimates did not differ significantly when the analysis included passed reads alone or all reads. We estimated the 90% CI in cpm using a β-distribution (Supplementary Fig. S7). All highly expressed genes were detected within 6 hours as per the real-time detection emulation. As anticipated, low levels of expression were observed for fosfomycin (*fosA*), tetracycline (*tet(A), tet(B)*), and macrolide (*mph(A)*) resistance. Several resistance genes were identified to be regulated by operons, and co-expression was evident for *oqxAB* (1_GR_13, 16_GR_13), *blaVEB-1*: *ant(2”)-Ia*: *ARR-2* (1_GR_13), *aadA1*: *sul1* (1_GR_13), *rmtB*: *blaTEM-1B* (1_GR_13, 2_GR_12, 16_GR_13), *aph(6)-Id*: *strA* (1_GR_13), *sul2*: *aph(3”)-Ib*: *aph(6)-Id* (2_GR_12, 16_GR_13), *ant(2”)-Ia*: *blaVEB-1* (2_GR_12, 16_GR_13), *aac(6’)-Ib-cr*: *blaOXA-1*: *catB4* (16_GR_13), *aadA2*: *sul1* (16_GR_13), and *sul2*: *aph(3”)-Ib*: *dfrA14* (20_GR_12) (Fig. 2). Overall, various non-rRNA genes were identified to be co-expressed (≥5 reads supporting gene intersect) across isolates (1_GR_13: 428; 2_GR_12: 310; 16_GR_13: 793; 20_GR_12: 442).

**Figure 3:**
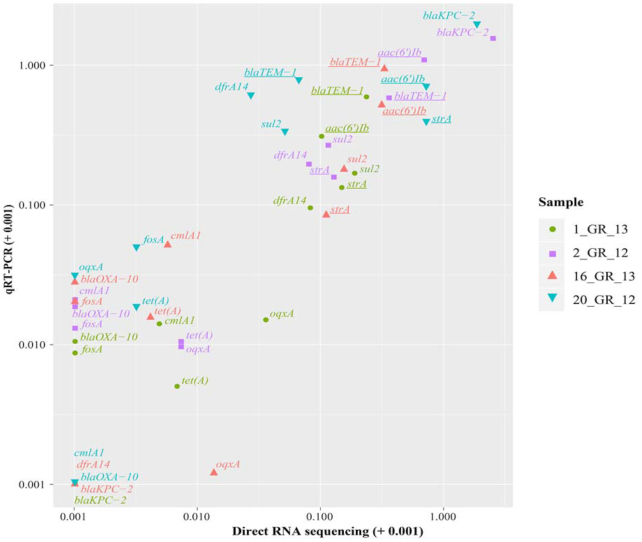
Correlation between resistance genes detected via direct RNA sequencing and validated using qRT-PCR. Relative expression was calculated via normalizing to the housekeeping gene, *rpsL*, for both direct RNA sequencing (log_2_(gene/*rpsL*)) and qRT-PCT (2^−(gene/^*^rpsL^*^)^). Owing to high similarity between certain genes, several primers recognize >1 gene (underlined). These include *aac(6’)Ib: aac(6’)Ib-cr, aadA24; strA: aph(3'')-Ib*, and *blaTEM-1: blaTEM-1A, blaTEM-1B*. Values are log_10_ transformed and shifted +0.001 to display genes with no detectable expression.

A subset of 11 resistance genes that represent resistance across various classes of antibiotics were investigated to validate gene expression in these RNA extractions via qRT-PCR (Fig. 3). These included resistance towards aminoglycosides (*aac(6’)Ib, strA*), β-lactams (*blaKPC-2, blaOXA-10, blaTEM-1*), phenicols (*cmlA1*), trimethoprim (*dfrA14*), fosfomycin (*fosA*), quinolone (*oqxA*), sulphonamides (*sul2*), and tetracyclines (*tet(A)*). A similar trend was observed between direct RNA sequencing and qRT-PCR results (Spearman rank correlation coefficient: 0.83; Pearson correlation: 0.86) (Fig. 3). The highest expression of a resistance gene was observed for *blaKPC-2*, although only 1 copy was present in a lower copy number plasmid in 2_GR_12 and 20_GR_12 (Figs 2 and 3 and Table 1). Additionally, low levels of expression for *fosA* and *tet(A)* were apparent despite exhibiting resistance towards fosfomycin and tetracycline (Fig. 3, Supplementary Table S1). Direct RNA sequencing was unable to detect low levels of expression whilst qRT-PCR could detect these genes (Fig. 3).

**Figure 4:**
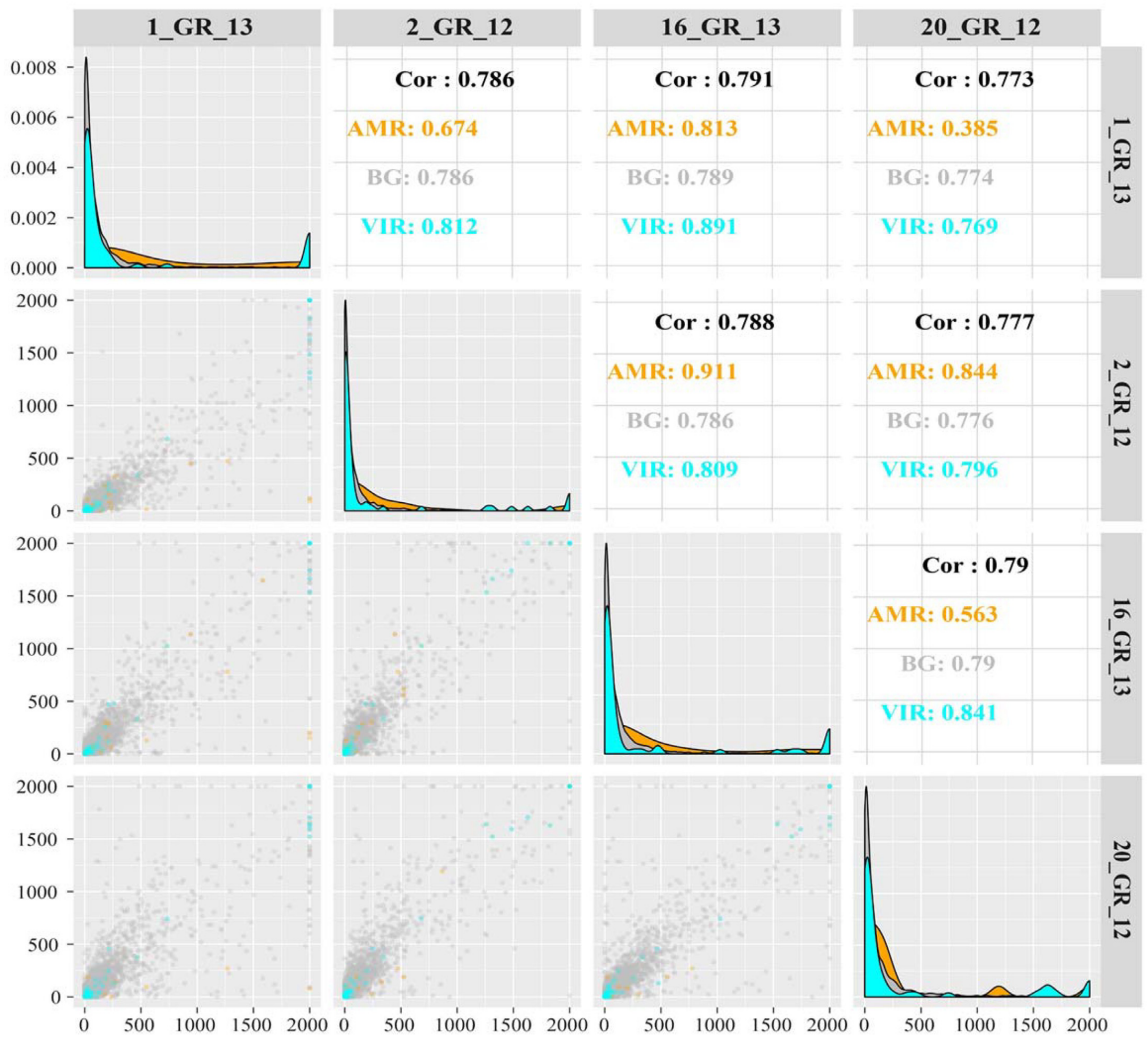
Correlation between the 4 XDR *K. pneumoniae* isolates for gene expression via direct RNA sequencing. Top panels display Spearman correlation coefficients. The diagonal panel shows the density of gene expression levels in counts per million mapped reads for each sample (after removal of rRNA mapped reads). Bottom panels depict the correlation of gene expression between isolates as a scatter plot. Colours indicate categorization of gene: antimicrobial resistance genes (AMR) as per ResFinder 3.0, virulence genes (VIR) determined via RAST, and all other genes or background genes (BG) are displayed. cpm was capped at 2,000.

Across the transcriptome, antibiotic resistance genes were identified to harbour high expression between isolates (Fig. 4). Virulence genes were comparable across these strains similar to all remaining or background genes. The top differentially expressed genes were determined (Supplementary Fig. S8), and several were associated with polymyxin resistance pathways. Heightened expression was seen in polymyxin-resistant isolates 1_GR_13, 2_GR_12, and 16_GR_13 in comparison to the single susceptible isolate (20_GR_12), in particular, genes associated with Ara4N synthesis. These genes include 4-deoxy-4-formamido-L-arabinose-phosphoundecaprenol deformylase (*arnD*), UDP-4-amino-4-deoxy-L-arabinose formyltransferase, and UDP-4-amino-4-deoxy-L-arabinose-oxoglutarate aminotransferase.

### Transcriptional biomarkers for polymyxin resistance

**Figure 5:**
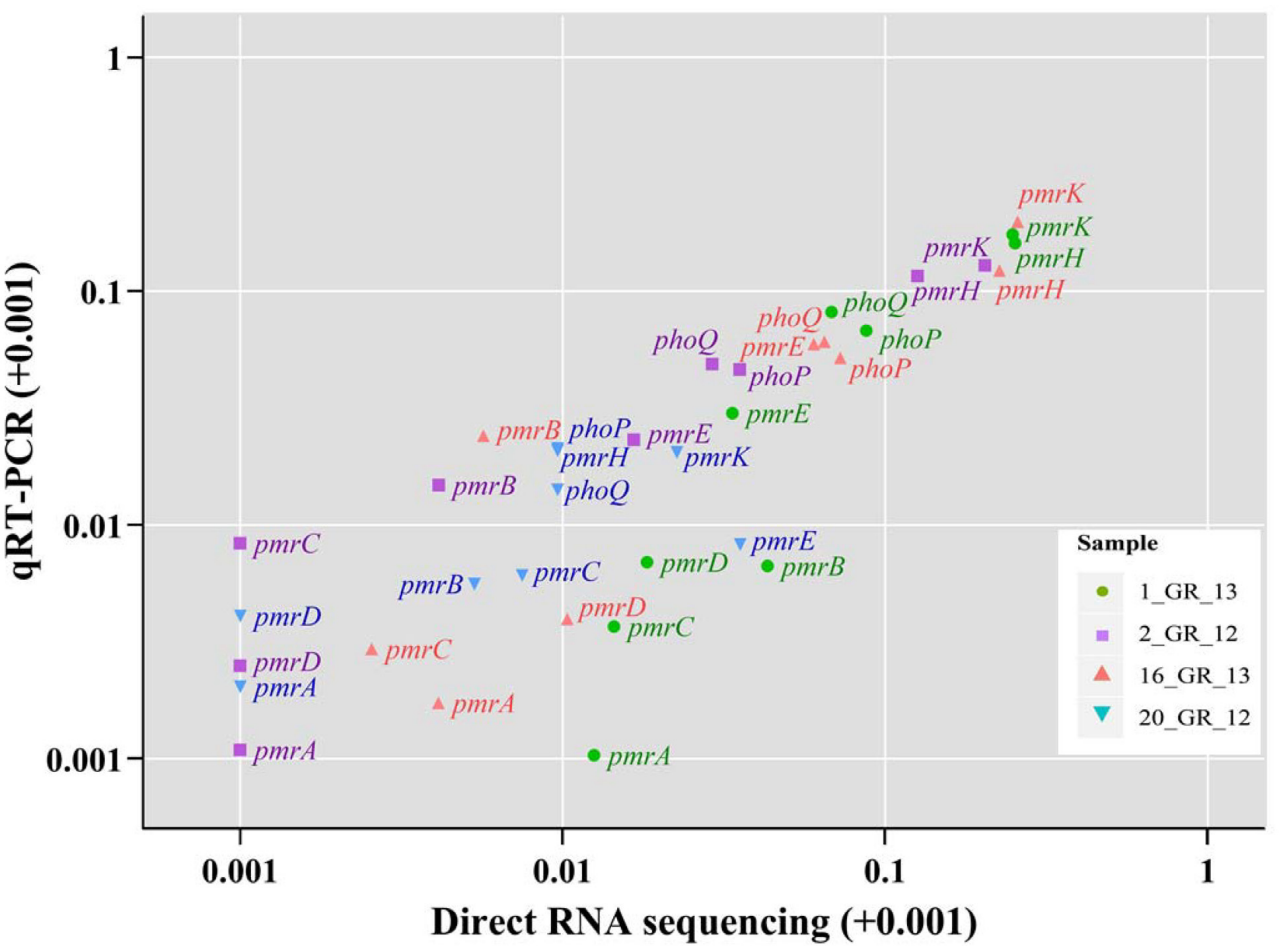
Expression of genes associated with the polymyxin resistance pathway. Comparison between direct RNA sequencing (log_2_(gene/*rpsL*)) and qRT-PCR (2*^−^*^(gene^*^/rpsL^*^)^). All isolates except 20_GR_12 harboured resistance to polymyxin (MIC: >2 µg/mL), and genes were normalized to the housekeeping gene *rpsL*. Values are log_10_ transformed and shifted +0.001 to display genes with no detectable expression.

Three of the isolates harboured resistance towards polymyxins via disruptions in *mgrB*, which included 1_GR_13, 2_GR_12, and 16_GR_13. Isolate 1_GR_13 has an insertion sequence (IS) element, IS*Kpn26*-like, at nucleotide position 75 in the same orientation as *mgrB* whilst 2_GR_12 has this IS element in the opposite orientation plus additional mutations in *phoP* (A95S) and *phoQ* (N253T). 16_GR_13 harbours an IS element, IS*1R*-like, 19 bp upstream of *mgrB*. Direct RNA sequencing revealed only low-level expression of *mgrB* (1_GR_13: 78.4 cpm; 2_GR_12: 16.3 cpm; 16_GR_13: 0 cpm; 20_GR_12: 2.3 cpm). The expression levels of various genes associated with this pathway were verified via qRT-PCR (Fig. 5). Direct RNA sequencing revealed a slight increase in transcription of *phoPQ* (≥2-fold) relative to 20_GR_12. A ≥13-fold increase in expression was observed for *pmrH* and ≥8-fold elevation for *pmrK*. Similar trends for expression were also reported using qRT-PCR (Fig. 5).

## Discussion

XDR *K. pneumoniae* infections pose a major threat to modern medicine. A rapid diagnostic would help to guide appropriate treatment options [1, 6]. The MinION sequencing technology used in this study has potential to detect antibiotic resistance in a timely manner. Three of the 4 *K. pneumoniae* isolates examined in this study harboured non-susceptibility to all antibiotics or antibiotic combinations assayed and hence would be classified as PDR according to published guidelines [52]. ONT sequencing was able to resolve both the assembly of plasmids harbouring high levels of resistance (through DNA sequencing) and the expression from the resistome in the absence of antibiotic treatment (via RNA sequencing).

The ability for ONT to sequence long fragments of DNA has significantly aided the assembly of bacterial genomes and plasmids [16–18]. In this study, multiple megaplasmids (≥100 kb) were identified that were previously unresolved via Illumina sequencing [28]. These harboured replicons IncA/C2 or a dual replicon, IncFIIK and IncFIB. The IncA/C, IncF, and IncN plasmids have been commonly associated with multidrug resistance [53]. Although several plasmids in this study revealed similarity to previously reported isolates via NCBI, various sequences deviated. In particular, the IncA/C2 plasmid exhibited multiple regions unique to these isolates. Several IncA/C2 megaplasmids have been previously described that harbour various resistance genes. However, the extent of resistance observed in our study is extreme in comparison with prior reports [54, 55]. Prior studies have shown the IncFIIK and IncFIB replicons to localize on the same plasmid and also megaplasmids with multidrug resistance [6]. The IncFIB_pQil_ plasmid in this study contained various β-lactam resistance genes (*blaKPC-2, blaOXA-9, blaTEM-1A*) that had been identified previously [56]. Similarly, *blaOXA-48* segregated with the IncL/M replicon [57, 58]; however, deviations in this plasmid were identified.

The real-time analysis capability entailed in MinION sequencing has the potential to rapidly determine antibiotic resistance profiles of pathogenic bacteria. Previously this device has been used to assemble bacterial genomes, discern species, and detect antibiotic resistance [12–15]. This study investigated the potential time required to discern resistance via a real-time emulation as previously described [14]. The majority (≥70%) of resistance genes were detected via DNA sequencing within 2 hours. Several genes not identified in the final assembly were detected after 2 hours of sequencing. This may be attributed to the high similarity (≥80%) amongst various genes, in particular, those associated with aminoglycoside, β-lactam, rifampicin, and phenicol resistance. Furthermore, the error rate associated with ONT sequencing, and the accumulation of these errors over time, may result in the false annotation of these genes. Nanopore DNA sequencing currently has an accuracy ranging from 85 to 95% (90% in our study), which limits its ability to detect genomic variations [17, 59]. Several resistance genes only differ by a few nucleotides, which significantly affects the resistance phenotype and the antibiotics that can be used to treat these infections. However, software tools such as Nanopolish [60] and Tombo [61] (similarly used to retrain Chiron v0.5 for direct RNA sequencing data) have the potential to correct these reads and would be helpful to integrate to increase the accuracy of detecting resistance genes. We used native DNA sequencing in this study, which retains epigenetic modifications such methylation that can hinder the accuracy of reads and subsequent calling of antibiotic resistance [62]. Furthermore, a small number of resistance genes were identified that were not present in the final assembly; however, these all had MAPQ values <10 and <30 mapped reads. This may be due to low-level kit contamination, while some of the false-positive results have sequence similarity to true-positive results and may be due to inaccuracies in base-calling.

We further investigated the transcriptome of these isolates to potentially elucidate the correlation between genotype and the subsequent resistant phenotype. Detection of antibiotic resistance via sequencing commonly uses DNA owing to the instability of RNA and the lengthy sample processing such as rRNA depletion [12–15, 62]. However, RNA provides additional information regarding the functionality of genes such as identifying conditions in which a resistance gene is present but not active, which gives rise to a false-positive result via DNA alone. Conversely, if expression is only induced in the presence of an antibiotic, the absence of RNA transcripts results in a false-negative result. This study grew *K. pneumoniae* strains in the absence of antibiotic, and direct RNA sequencing revealed high levels of transcription from genes associated with aminoglycoside, β-lactam, sulphonamide, and trimethoprim resistance within 6 hours of our study. In particular, the highest levels of expression were observed for the β-lactamase gene *blaKPC-2* in 2_GR_12 and 20_GR_12. Alterations in the promoter region have previously been reported to influence high levels of expression [63]. Notably, the promoter or operon (co-transcribed genes) can greatly influence expression of genes, with several resistance genes potentially identified to be regulated by operons in this study. The detection of quinolone, rifampicin, and phenicol resistance correlated to the levels of transcription within samples. All isolates exhibited low levels of expression for fosfomycin, macrolide, and tetracycline resistance, despite exhibiting phenotypic resistance to fosfomycin and tetracycline [28]. FosA, an enzyme involved in fosfomycin degradation, is commonly encoded chromosomally in *K. pneumoniae*, and a combination of expression and enzymatic activity contributes to resistance [64]. Noteably, Klontz et al. identified that chromosomally integrated FosA, similarly observed in our study, from *K. pneumoniae* harboured a higher catalytic efficiency. A higher catalytic efficiency may explain why our strains only require a low abundance of expression and still retain fosfomycin resistance. Genes *tet(A)* and *tet(G)* encode efflux pumps, which, in the absence of tetracycline, have a low level of expression, and the lack of antibiotic supplementation in this study confirms this observation [65]. Detecting inducible resistance (antibiotic exposure required for gene expression) such as tetracycline resistance highlights one of the advantages of investigating the transcriptome.

There are several other variables to consider when interpreting expression levels in bacterial RNA sequencing data. These include the extent to which prior exposure to antibiotics in the clinic alters transcription and the copy number of resistance genes and the plasmids that these are encoded on. Limitations were observed when base-calling bacterial direct RNA sequencing and may be attributed to trimming the long artificial poly(A) tail and interference of RNA modifications. This entailed an increased error rate of ≤23% across base-callers (12% identified in a prior study [21]) and a poor alignment rate ≤23%. Furthermore, the time required to detect resistance may be hindered by the slower translocation speed associated with direct RNA sequencing (70 bases/second) compared to DNA sequencing (450 bases/second) [59]. Our findings show that the slower time-to-detection of resistance genes in direct RNA sequencing was due to both the level of expression as well as the slower translocation speed, and hence using cDNA would only partially overcome this limitation.

We also investigated pathways attributed to polymyxin resistance. Three of these strains exhibited an IS element upstream or within *mgrB*, the negative regulator of PhoPQ [29]. Elevated expression was apparent for *phoPQ* and also the *pmrHFIJKLM* operon in our polymyxin-resistant isolates harbouring a disrupted *mgrB*. This has previously been witnessed for other *K. pneumoniae* isolates harbouring *mgrB* disruptions and is a potential transcriptional marker for polymyxin resistance [29, 48, 66, 67]. However, this study is limited to 4 isolates and 1 mechanism associated with polymyxin resistance. Other pathways have previously been identified including the role of other 2 component regulatory systems such as CrrAB [68]. The ability to use relative expression of key genes to detect polymyxin resistance requires further validation, including an increased sample size of resistant and non-resistant isolates. Furthermore, additional functional experiments such as complementation assays would be required in order to validate the contribution of a certain mutation to the transcriptome and subsequent resistance.

## Conclusions

This study used MinION sequencing to assemble 4 XDR *K. pneumoniae* genomes and has revealed several unique plasmids harbouring multidrug resistance. Most of this resistance was detectable within 2 hours of sequencing. Exploiting this analysis in real time would allow for a rapid diagnostic; however, the presence of a resistance gene does not necessarily indicate that resistance is conferred and requires additional phenotypic characterization. This research also established a methodology and analysis for bacterial direct RNA sequencing. The expression of resistance genes was successfully detected in addition to identifying genes potentially regulated via operons; however, native RNA sequencing incurs a slower time to detect resistance owing to translocation speed. Once base-calling algorithms have been optimized, this could allow for a whole-transcriptome interrogation of the poorly characterized bacterial RNA modifications. Overall, this study has begun to unravel the association between genotype, transcription, and subsequent resistant phenotype in these XDR/PDR *K. pneumoniae* clinical isolates, establishing the groundwork for developing a diagnostic that can rapidly determine bacterial resistance profiles.

## Availability of Supporting Data and Materials

The datasets supporting the results presented here are available in the NCBI repository BioProject PRJNA307517 (www.ncbi.nlm.nih.gov/bioproject/PRJNA307517). ONT DNA sequencing data have been deposited in the SRA (www.ncbi.nlm.nih.gov/sra/) under study SRP133040. Accession numbers are as follows: 1_GR_13 (SRR6747887), 2_GR_12 (SRR6747886), 16_GR_13 (SRR6747885), and 20_GR_12 (SRR6747884). ONT direct RNA sequencing data (pass and fail reads) have been deposited in the SRA under study SRP133040. Accession numbers are as follows: 1_GR_13 (SRR7719054), 2_GR_12 (SRR7719055), 16_GR_13 (SRR7719052), and 20_GR_12 (SRR7719053). Alignments, assemblies, and other supporting data are also available from the *GigaScience* GigaDB repository [69].

## Additional Files

**Table S1:** Minimum inhibitory concentrations of the 4 *Klebsiella pneumoniae* clinical isolates

**Table S2:** Genome assembly comparison

**Table S3:** Oligonucleotides used in this study for qRT-PCR

**Table S4:** Highest similarity observed for final assembly contigs when aligned to NCBI database

**Table S5:** Real-time emulation of time to detect resistance genes from DNA and RNA sequencing

**Table S6:** Comparison between different base-calling programs using direct RNA sequencing data

**Figure S1:** Tapestation traces of high molecular weight DNA samples, including (**A**) 1_GR_13, (**B**) 2_GR_12, (**C**) 16_GR_13, (**D**) 20_GR_12. The 100 nt marker is indicated with an M, and number indicates the most abundant length.

**Figure S2:** Bioanalzyer traces comparing mRNA enrichment and subsequent poly(A) ligation for RNA samples. Sample conditions are as follows: (**A**) 1_GR_13 rRNA depletion, (**B**) 1_GR_13 poly(A) ligation, (**C**) 2_GR_12 rRNA depletion, (**D**) 2_GR_12 poly(A) ligation, (**E**) 16_GR_13 rRNA depletion, (**F**) 16_GR_13 poly(A) ligation, (**G**) 20_GR_12 rRNA depletion, (**H**) 20_GR_12 poly(A) ligation. The 25 nt marker is indicated with an M; presence of 16S rRNA (16S) and 23S rRNA (23S) are also shown.

**Figure S3:** Size distribution of reads for sequenced samples. Isolates sequenced by the MinION platform include (**A**) 1_GR_13 DNA, (**B**) 2_GR_12 DNA, (**C**) 16_GR_13 DNA, (**D**) 20_GR_12, (**E**) 1_GR_13 RNA, (**F**) 2_GR_12 RNA, (**G**) 16_GR_13 RNA, (**H**) 20_GR_12 RNA. Reads were base-called using Albacore 2.1.1 for DNA and 2.2.7 for RNA.

**Figure S4:** Detection of resistance genes via the real-time emulation analysis using DNA or direct RNA MinION sequencing. (**A**) 1_GR_13, (**B**) 2_GR_12, (**C**) 16_GR_13, and (**D**) 20_GR_12. The y-axis displays the resistance genes, where a slash indicates reads detecting >1 gene, pound sign is a family of genes (>3), and boldface displays a gene identified in the final assembly. An asterisk on bars highlights the lack of detection in direct RNA sequencing. Albacore base-calling was used for all datasets. The x-axis shows the amount of data (Mb) required for a resistance gene to be confidently called via the emulation.

**Figure S5:** Diagram of RNA neural network underlying Chiron RNA model, consisting of 3 residual layers and 3 long short-term memory layers. The model was trained as follows: First we used Albacore 2.2.7 to base-call raw data and the nanopolish poly(A) segmentation tool to remove the signal corresponding to the poly(A) tail prior to dataset labelling. We then aligned base-called data to reference genomes with BWA-MEM and then Tombo-1.4 to re-squiggle the raw signal data to the reference genome DNA (i.e., align the signal with the underlying bases that generated the signal). Chiron was trained using a chunk length of 2,000, 80,000 training steps, and an initial learning rate of 0.004, with the following command: python chiron/chiron_rcnn_train.py -i $INPUT_DIR -f $INPUT_FILES -v 20_GR_12_validation.tfrecords -o $OUTPUT –model $MODEL_NAME –configure $WORK_DIR/Chiron/sample_config/model_rna3.json –train_cache $CACHE_DIR/2000l.hdf5 –valid_cache $CACHE_DIR/2000l_20_GR_12_valid.hdf5 -s 2000 -b 50 -t 4e-3 -x 80 000 –resample_after_epoch 1 –threads 8

**Figure S6:** Size distribution of poly(A) tails determined via direct RNA MinION sequencing. Samples include (**A**) 1_GR_13, (**B**) 2_GR_12, (**C**) 16_GR_13, and (**D**) 20_GR_12. Poly(A) length was determined using Nanopolish (https://github.com/jts/nanopolish).

**Figure S7:** Estimated 90% confidence intervals as a function of estimated cpm for direct RNA sequencing data for 4 samples. Estimates derived from a β-distribution with shape parameters α, β equal to the 0.1 + number of reads mapping to a given gene and 0.1 + number of reads mapping to all other genes.

**Figure S8:** Heat map depicting the top differentially expressed genes across the 4 *K. pneumoniae* isolates. Expression determined via ONT direct RNA sequencing. Key indicates whether these genes were over-expressed (yellow) or under-expressed (red). Grey indicates the absence of this gene in the isolate. An asterisk indicates pathways associated with polymyxin resistance, and values represent log_10_(cpm).

giaa002_GIGA-D-19-00200_Original_Submission

giaa002_GIGA-D-19-00200_Revision_1

giaa002_GIGA-D-19-00200_Revision_2

giaa002_Response_to_Reviewer_Comments_Original_Submission

giaa002_Response_to_Reviewer_Comments_Revision_1

giaa002_Reviewer_1_Report_Original_Submission Rachael Workman -- 6/25/2019 Reviewed

giaa002_Reviewer_1_Report_Revision_1 Rachael Workman -- 11/6/2019 Reviewed

giaa002_Reviewer_2_Report_Original_Submission David Eccles -- 7/1/2019 Reviewed

giaa002_Reviewer_3_Report_Original_Submission Sheng CHEN -- 7/13/2019 Reviewed

giaa002_Supplemental_Files

## Abbreviations

Ara4N: 4-amino-4-deoxy-L-arabinose; BLAST: Basic Local Alignment Search Tool; bp: base pairs; BWA: Burrows-Wheeler Aligner; cDNA: complementary DNA; CI: confidence interval; CLSI: Clinical and Laboratory Standards Institute; cpm: counts per million; EUCAST: European Committee on Antimicrobial Susceptibility Testing; FDR: false discovery rate; Gb: gigabase pairs; HMW: high molecular weight; IS: insertion sequence; kb: kilobase pairs; MAPQ: mapping quality; Mb: megabase pairs; MIC: minimum inhibitory concentration; mRNA: messenger RNA; NCBI: National Center for Biotechnology Information; ONT: Oxford Nanopore Technologies; PDR: pandrug-resistant; RAST: Rapid Annotation using Subsystem Technology; rRNA: ribosomal RNA; SPAdes: St. Petersburg genome assembler; SRA: Sequence Read Archive; ST: Sequence type; XDR: extensively drug-resistant.

## Competing Interests

The authors declare that they have no competing interests.

## Funding

L.J.M.C. is an NHMRC career development Fellow APP1103384. M.A.C. is an NHMRC Principal Research Fellow (APP1059354) and currently holds a fractional Professorial Research Fellow appointment at the University of Queensland with his remaining time as CEO of Inflazome Ltd. a company headquartered in Dublin, Ireland that is developing drugs to address clinical unmet needs in inflammatory disease by targeting the inflammasome. M.E.P. is an Australian Postgraduate Award scholar. M.A.T.B. is supported in part by a Wellcome Trust Strategic Award 104797/Z/14/Z. This work was supported by the Institute for Molecular Bioscience Centre for Superbug Solutions (610246) and an Australian Research Council Discovery Project DP170102626.

## Authors' Contributions

M.E.P., L.J.M.C., M.A.T.B., and M.A.C. conceived this study. M.E.P., S.H.N., and H.T. performed the sequencing analysis. Laboratory work was carried out by M.E.P. and T.P.S.D. M.E.P. wrote the manuscript with input from all authors.
