# Supplementary material for: Evaluating the genome and resistome of extensively drug-resistant Klebsiella pneumoniae using native DNA and RNA Nanopore sequencing
Source: Gigascience. 2020 Feb 4;9(2):giaa002. doi: 10.1093/gigascience/giaa002 (PMC6998412; doi:10.1093/gigascience/giaa002)
Supplement: giaa002_GIGA-D-19-00200_Revision_1 [file giaa002_giga-d-19-00200_revision_1.pdf]

GigaScience

Evaluating the Genome and Resistome of Extensively Drug-Resistant Klebsiella pneumoniae using Native DNA and RNA Nanopore Sequencing

--Manuscript Draft--

Manuscript Number:	GIGA-D-19-00200R1
Full Title:	Evaluating the Genome and Resistome of Extensively Drug-Resistant Klebsiella pneumoniae using Native DNA and RNA Nanopore Sequencing
Article Type:	Research
Funding Information:	Institute for Molecular Bioscience Centre for Superbug Solutions (610246) — Dr Lachlan Coin
Abstract:	Background: Klebsiella pneumoniae frequently harbours multidrug resistance and current diagnostics struggle to rapidly identify appropriate antibiotics to treat these bacterial infections. The MinION device can sequence native DNA and RNA in real-time, providing an opportunity to compare the utility of DNA and RNA for prediction of antibiotic susceptibility. However, the effectiveness of bacterial direct RNA sequencing and base-calling has not previously been investigated. This study interrogated the genome and transcriptome of four extensively drug-resistant (XDR) K. pneumoniae clinical isolates, however, further antimicrobial susceptibility testing identified three isolates as pandrug-resistant (PDR).<br>Results: The majority of acquired resistance (≥75%) resided on plasmids including several megaplasmids (≥100 kbp). DNA sequencing detected most resistance genes (≥70%) within 2 hours of sequencing. Neural-network based base-calling of direct RNA achieved up to 86% identity rate, although ≤23% of reads could be aligned. Direct RNA sequencing (with approximately 6 times slower pore translocation) was able to identify (within 10 hours) ≥35% of resistance genes, including those associated with resistance to aminoglycosides, β-lactams, trimethoprim and sulphonamide and also quinolones, rifampicin, fosfomycin and phenicol in some isolates. Polymyxin-resistant isolates showed a heightened transcription of phoPQ (≥2-fold) and the pmrHFIJKLM operon (≥8-fold). Expression levels estimated from direct RNA sequencing displayed strong correlation (Pearson: 0.86) compared to qRT-PCR across eleven resistance genes.<br>Conclusion: Overall, MinION sequencing rapidly detected the XDR/ PDR K. pneumoniae resistome and direct RNA sequencing provided accurate estimation of expression levels of these genes.
Corresponding Author:	Lachlan Coin<br><br>AUSTRALIA
Corresponding Author Secondary Information:	
Corresponding Author's Institution:	
Corresponding Author's Secondary Institution:	
First Author:	Miranda E. Pitt
First Author Secondary Information:	
Order of Authors:	Miranda E. Pitt
	Son H. Nguyen
	Tânia P.S. Duarte
	Haotian Teng
	Mark A.T. Blaskovich
	Matthew A. Cooper
	Lachlan Coin

Powered by Editorial Manager® and ProduXion Manager® from Aries Systems Corporation

**Order of Authors Secondary Information:**	
**Response to Reviewers:**	“Evaluating the Genome and Resistome of Extensively Drug-Resistant Klebsiella pneumoniae using Native DNA and RNA Nanopore Sequencing”<br>GIGA-D-19-00200<br>Response to Reviewers<br><br>Dear Dr. Scott Edmunds,<br><br>We thank the reviewers for the opportunity to revise this manuscript (GIGA-D-19-00200). Their comments have helped us significantly strengthen the work. We have now provided additional information including rationale for using direct RNA sequencing and particular analysis methodologies. Figures have also been modified to aid with the interpretation of data. To highlight the adjustments completed, we have also uploaded a mark-up version of the manuscript. Please find below a point-by-point response to the reviewers’ comments.<br><br>Reviewer reports:<br><br>Reviewer #1:<br>In the manuscript "Evaluating the Genome and Resistome of Extensively Drug-Resistant Klebsiella pneumoniae using Native DNA and RNA Nanopore Sequencing" by Pitt et al., the authors describe datasets generated from multiple sequencing modalities of antibiotic-resistant clinical isolates, and discuss the potential of this technology for rapid detection of AMR. Although these methods and sequencing characterization and analysis are of importance to the field, there are several issues which remain to be addressed.<br><br>Specific points:<br>It would be useful to better establish the rationale for why direct detection of RNA transcripts matters, and what additional information direct RNA sequencing gets you that rapid cDNA conversion and sequencing can't. Perhaps the largest issue is - "Why dRNA-seq?" There doesn't seem to be an obvious benefit, given the poor time to detection compared to just DNA sequencing. Expression levels are useful, but could be determined from Illumina sequencing. Without splicing there are no isoforms to contend with, and the error rate adds difficulty in interpretation and determination of primary protein sequence. Additionally, most clinical bacterial characterization work doesn't use RNA-seq, and addressing the problems clearly (i.e. rRNA depletion, RNA instability) should be done at the outset.<br>Response: We have now provided additional information to highlight the benefits of using direct RNA sequencing in the introduction and discussion. The time to detect antibiotic resistance using direct RNA sequencing was slower compared to DNA, however, this is only the first generation of the technology. The latest kit, SQK-RNA002, has shown advancements in data generation which unfortunately was not available during the time of this study. “Our findings show that the slower time-to-detection of resistance genes in direct RNA sequencing was due to both the level of expression as well as the slower translocation speed, and hence using cDNA would only partially overcome this limitation.” (Discussion: Line 396, also refer to Supplementary Figure S4). “Furthermore, library preparation time is halved for direct RNA sequencing due to the absence of cDNA synthesis” (Introduction: Line 57). Indeed, expression levels can be determined via Illumina sequencing, however, in the context of a diagnostic tool, Illumina platforms require the completion of the sequencing run (~48 hours) to output data and analysis to be performed. Nanopore technologies can output data as soon as it is generated to enable real-time analysis. Although bacteria lack splicing, long read sequencing has the potential to detect operon sites where several transcripts are co-expressed (refer to Line 59 and 417). Due to difficulties extracting RNA from these strains and downstream processing for sequencing, these transcripts were short and not enough data was generated to confidently detect operon sites (Supplementary Figure S3). Furthermore, native RNA sequencing has the potential to detect RNA modifications associated with antibiotic resistance which are removed when converted to cDNA and is unique to this technology (Introduction: Line 55). Although RNA is unstable and requires several additional processing steps compared to DNA, advancements on this part could be made in the future and hence, the potential for this to be used to detect antibiotic resistance was explored. We have now made note of the limitations associated with

RNA sequencing in the clinic (Discussion: Line 368). Additionally, RNA has the potential to determine the functionality of a resistance genes as the presence of these genes does not necessarily mean they confer resistance (Discussion: Line 369).

Under the "DNA extractions and HMW DNA isolation methods section", this section should be rewritten for clarity - it was confusing to determine which isolations worked and which didn't, and why. It's still important to include details of why protocol modifications were made, but if these could be incorporated into methods better that would aid in understanding.
Response: This section has now been rewritten ("High molecular weight DNA isolation", page 4). Several modifications were implemented primarily due to difficulties lysing these highly antibiotic-resistant K. pneumoniae strains potentially due to a thickened capsule wall. This resulted in capsule contamination (carbohydrate) as determined via Nanodrop (Line 96). This was very cumbersome for isolate 2_GR_12 which was noted to have an increased carbohydrate contamination potentially due to the capsule and required a further purification step (Line 97).

Under "real-time resistome detection emulation" as well as "assembly of genomes" sections, it would be helpful to include a rationale on why certain software tools were chosen over others, given you tried many options. For example, why was BWA-MEM chosen over minimap2?
Response: In light of the vast amount of software tools available, we selected the four most commonly used tools for bacterial assembly. These incorporated both hybrid assemblers (Unicycler, npScarf) and the remaining two using only Nanopore reads (Canu, Minimap2/ Miniasm/ Racon). We trialed analysis using minimap2 initially, however, a lower alignment rate was observed potentially due to the majority of reads being less than 1000 bp (Supplementary Figure S3). This has now been mentioned in the supplementary section: Supplementary Table S6 and noted in the main text (Line 148) which also notes adjusted parameters used for BWA-MEM when using ONT reads.

How were you able to distinguish multiple copies of resistance genes from duplicated misassemblies?
Response: Both the fragment distribution (Supplementary Figure S1) and the read-length distribution (Supplementary Figure S3 A-D) indicate substantial number of reads of length greater than 10kb. The vast majority of bacterial repeats are shorter than 10kb, meaning that we are able to correctly place these repeats in the assembly. Furthermore, these long reads were able to span the duplicated resistance gene regions and correctly assemble these plasmids.

Would it actually be faster to detect with cDNA sequencing, given faster motor protein translocation rate and likely higher copy number of transcripts of interest? It would be useful to include thoughts on this in the discussion.
Response: While the sequencing speed of cDNA is currently faster than direct RNA (450 bases/second vs 70 bases per second) the library preparation for direct RNA is much quicker (105 minutes vs 270 minutes). Moreover, it is anticipated that future direct RNA sequencing kits will run at the same translocation speed as cDNA. We considered the translocation speed impeding on the detection method, hence, why we included an analysis total yield required to detect resistance genes as well as time to call the resistance genes (Line 266, Supplementary Figure S4). We have now added an additional sentence in the discussion: "Our findings show that the slower time-to-detection of resistance genes in direct RNA sequencing was due to both the level of expression as well as the slower translocation speed, and hence using cDNA would only partially overcome this limitation." (Line 396).

You say "Nanopore DNA sequencing currently has an accuracy ranging from 80 to 90%, which limits its ability to detect genomic variations", but there are post-processing tools available to increase accuracy and ability to detect SNVs - this should be included in the discussion.
Response: Agreed, there are tools to improve the accuracy which we have now made note of in the discussion: "However, software tools such as Nanopolish (https://github.com/jts/nanopolish) and Tombo (https://github.com/nanoporetech/tombo) (similarly used to re-train Chiron v0.5 for direct RNA sequencing data) have the potential to correct these reads and would be helpful to integrate to increase the

accuracy of detecting resistance genes." (Line 359).

Further the detection of SNV mutations and indels is critical with respect to the detection of chromosomal mutations in these samples. Additional consideration of methylation signatures is crucial, as they can cause systematic error (PMID: 30373801) if not corrected.
Response: We have now noted the influence of DNA modifications on the accuracy of Nanopore sequencing and included this publication. "We utilised native DNA sequencing in this study which retains epigenetic modifications such methylation which can hinder the accuracy of reads and subsequent calling of antibiotic resistance [58]." (Line 362).

"All isolates exhibited low levels of expression for fosfomycin, macrolide and tetracycline resistance, despite exhibiting phenotypic resistance to fosfomycin and tetracycline", but are high levels of expression essential for phenotypic resistance? Are these low levels surprising? It would be helpful to link to papers discussing this.
Response: Additional information has now been included to identify why low expression of particular genes was observed. Limited literature is available on these specific genes in K. pneumoniae with transcriptional and antimicrobial susceptibility testing. We have included the following sentence regarding fosfomycin resistance facilitated via the fosA gene: "Noteably, Klontz et al identified that chromosomally integrated FosA, similarly observed in our study, from K. pneumoniae harboured a higher catalytic efficiency. A higher catalytic efficiency may reason why our strains only require a low abundance of expression and still retain fosfomycin resistance" (Line 382). Low levels of expression for tetracycline are not surprising as this resistance is well characterized and found to be inducible (antibiotic exposure is required for expression of genes). This has been reworded: "Genes tet(A) and tet(G) encode efflux pumps which, in the absence of tetracycline, are lowly expressed and the lack of antibiotic supplementation in this study confirms this observation [61]. Detecting inducible resistance (antibiotic exposure required for gene expression) such as tetracycline resistance highlights one of the advantages of investigating the transcriptome." (Line 384)

Figure 5 - instead of switching back and forth between panels A and B, a scatterplot comparing the two directly like Fig 3 would be more useful.
Response: This figure has now been amended with the data on a single graph.

Why do you think only 23% RNA reads aligned? Did you try to identify the unaligned reads (like sort out contamination, noise)? It would be beneficial to include at least a blast/centrifuge style analysis trying to determine the source of the unaligned reads. Additionally, a k-mer analysis of the unaligned reads could help determine their origin.
Response: We identified that various failed reads were <10 bp (Supplementary Figure S3) which were filtered before alignment with BWA-MEM (k -11, seed length of 11 bp). Preliminary BLASTn analysis of unmapped reads identified a bacterial origin. The primary issue with the direct RNA sequencing data is the base-calling. When adapting Chiron v0.5 for this data, squiggle plots (raw nanopore data) identified insufficient trimming of the artificial poly(A). Furthermore, RNA modifications in bacteria remain largely unknown and this has the potential to interfere with the raw nanopore current change and subsequent base-calling. This has now been included in the discussion: "Limitations were observed when base-calling bacterial direct RNA sequencing and may be attributed to trimming the long artificial poly(A) tail and interference of RNA modifications." (Line 391).

How much of the poor alignment is due to the method of preparation (i.e. polyA tailing, etc.)? Did the authors perform optimization of the extraction and library prep for bacterial RNA? What about using an alternative tail and RNA adaptor?
Response: We trialed phenol/ chloroform RNA extractions however, this process was lengthy and resulted in a low yield of RNA and increased impurities. The PureLink RNA Mini Kit protocol is relatively quick (<30 mins/ sample). We attempted an on-column DNase treatment during this protocol but the best DNA depletion was observed using TURBO DNase which doesn't work on column (requires 37°C incubation). Our optimized RNA extraction resulted in Bioanalyzer RNA integrity scores of ≥8.5 which has now been included in Line 116 (RIN scale 0-10, 10 is no degradation using 16S and 23S pecks as reference). We considered altering the library preparation including

using an adapter similar to Smith et al (reference 26) which recognizes the Shine-Dalgarno sequence, however, there are deviations in this sequence and multi antisense adapters would be required so all transcripts are sequenced. Hence, the poly(A) tailing kit was more feasible as it will tag all 3'transcripts which allows for only the native RNA strand to be sequenced. Unfortunately, we were unaware of the efficiency of the polymerase until post sequencing analysis was performed (Supplementary Figure S6), hence, a shorter incubation can be implemented for future studies.

Viral direct RNA seq has been done (PMID: 30765700 and 30258076 for example) - it would be good to cite these or related papers.
Response: The updated publication of PMID: 30765700 rather than the preprint has been included in the references and PMID: 30258076 was originally incorporated in the introduction as reference 24 (refer to Line 54 for references referring to viral direct RNA sequencing). To our knowledge, all the publications on direct RNA sequencing are in the references.

Some minor points:
"This research also established a methodology and analysis for bacterial direct RNA sequencing." is repeated in the conclusions.
Response: This duplicated sentence has now been removed from the conclusions section.

Figure 2 colorblocking is a little confusing - could be more straightforward to break up the figure into separate panels per strain contig, for example with a ggplot facet_grid.
Response: Figure 2 has now been modified so genes belonging to particular contigs are easier to identify. This included adjusting the transparency of the colorblocking and splitting the x-axis similar to the ggplot facet_grid format.

Reviewer #2:
This manuscript presents a rapid resistance-gene discovery experiment, using genome sequencing and assembly to identify potentially-active genes, combined with differential expression to determine drug-free resistome activity. This manuscript is differentiated from most other direct-RNA and cDNA nanopore research, in that it is the *expression* rather than the *structure* of the genes is evaluated here. Bearing in mind that I cannot comment much on the biology side of things, I consider this manuscript to be a reasonable presentation of the experimental work that has been described, and recommend that it be accepted pending minor changes to figures, and clarification of multi-mapping results. I would like to thank the authors for making their Nanopore sequence data public prior to review submission; it demonstrates a good open research ethic.
My specific comments regarding the manuscript follow:
** Text **
L133: This references a fairly old version of Canu (i.e. v1.5), which seems a bit strange given that Guppy v3.0.3 is also mentioned (L260). I note that Canu v1.8 was released before Guppy v3.0.3, and would be interested to know why this version of Canu was chosen.
Response: Genome assemblies were conducted initially in this study and the transcriptomics at a later date. As we were able to complete the assemblies adequately using the hybrid assembler Unicycler and utilize Illumina reads to correct ONT sequencing errors, we did not run analysis on the most recent version of Canu. Furthermore, Guppy was integrated later as we had multiple issues with the base-calling of direct RNA sequencing and we hoped this update in the software would ameliorate this problem.

L144: I don't have an encyclopaedic knowledge of bwa-mem command-line options. It would be helpful to explain what the options mean. I'm particularly interested in why the default options were not appropriate, and what (if any) compensations were made for multi-mapped reads.
Response: This section has now been updated: "Similar parameters to the BWA-MEM ont2d function were used but seed length was reduced (-k 14) to compensate for shorter reads: -k 11 [minimum seed length, bp] -W20 [bandwidth] -r10 [gap extension penalty] -A1 [match score] -B1 [mismatch penalty] -O1 [Gap open penalty] -E1 [Gap

extension penalty] -L0 [Clipping penalty]). Multi-mapping reads were removed via SAMtools (secondary alignment: flagged as 256)…" (Line 149).

L144: Why was minimap2 not used here? It was written by the same author as bwa-mem, but is specifically written to incorporate corrections to improve mapping for noisy Nanopore Direct RNA-seq [e.g. see https://github.com/lh3/minimap2#getting-started]
Response: Preliminary analysis using minimap2 showed fewer reads aligning to the reference (now noted in the legend of Supplementary Table S6). It has been noted by Li H (doi: 10.1093/bioinformatics/bty191) that BWA-MEM is more suited to short read data and has a slightly improved accuracy compared to minimap2. We've further noted the bias towards BWA-MEM in Line 148: "BWA-MEM was selected due to shorter transcripts being produced by bacteria (Supplementary Figure S3) and the lack of introns and alternative splicing."

L145: I notice from L198 that there are gene copies in the data, with potentially high identity. Is there a particular reason why reads were mapped to the genome, rather than to transcriptome that merges essentially-identical genes?
Response: As described in the "Real-time resistome detection emulation" section (line 127), the resistance gene detection was carried out by mapping to a database of resistance genes which was clustered based on 90% identity threshold. However, in the section "RNA alignment and expression profiling" (Line 146) we mapped reads to the genome. In this case, if a read mapped to multiple locations equally well, then BWA-MEM randomly allocates to one position (primary alignment). Several instances of multiple copy numbers of resistance genes (Line 215) occurred which will influence the quantification of expression when aligned to the genome. Interestingly, there were some slight deviations in the expression of perfectly duplicated genes with unique flanking regions (refer to strA and sul1 in Figure 2A, contig 2 and 4) which may indicate that these genes are controlled by an operon (co-transcribed genes). This is an advantage of aligning to the genome. We also took this into consideration when graphing Figure 3 and combined all reads mapping to duplicated genes, such as strA, before normalizing to a housekeeping gene (rpsL).

L153: Why was a more well-known differential expression package not used here (e.g. DESeq2 or EdgeR) for evaluating differential expression? Is there an advantage of VGAM for plasmid or small genome differential expression?
Response: The beta-binomial distribution (implemented in VGAM) was used as a statistic to identify genes with significantly fewer or greater reads mapping in one sample versus another. It was chosen because it represents the uncertainty in the proportion estimated from count data. However, we agree that EdgeR and DESeq2 are also able to adequately estimate this uncertainty and hence we have redone the analysis using EdgeR (Supplementary Figure S7, Methods: "Whole transcriptome gene expression and estimation of expression confidence intervals", Line 157). The list of differentially expressed genes is very similar to that identified using VGAM (at least 90% identical).

L198 (see also L145): How identical were these genes? Would this identity affect genome mapping? In situations with multiple copies of near-identical genes, do you have any evidence to suggest that only one copy was active?
Response: These genes are 100% similar and will impact mapping to the genome. Unless expressed by an operon and the full-length sequences are retrieved, only then could this distinguish which genes are active. This issue will still arise if transcripts are mapped to the transcriptome. The only definitive way to determine this would be to perform knock-down studies of these regions and subsequently evaluate expression.

L218: What was the MAPQ probability for these genes? If the MAPQ probability is less than 3, it means that a gene could be equally-well placed at least two different sites (-log10(0.5) *10 ~= 3), which is expected given the gene duplication in your assemblies. I don't think this would indicate that the mapping is bad, as such, although there may be other reasons for a poor mapping.
Response: Agreed, the MAPQ score was commonly ≤10 for these duplicated reads. We have made a note of low mapping quality due to multiple copies of genes: "Low mapping quality could be attributed to assignment of reads to multiple copies of genes in the genome. Furthermore, the ONT error rates could lead to misassignment of reads to genes." (Line 275).

Powered by Editorial Manager® and ProduXion Manager® from Aries Systems Corporation

L228 (see also L198): more information about the similarity between the "correct" and "incorrect" gene would be useful; I notice that L335 mentions an identity for some genes of "greater than or equal to 80%". Do you have other evidence that systematic sequencing error would lead to reads being assigned to the incorrect gene?
Response: Various resistance genes harbor ≥80% similarity when taking into consideration genes deposited on the ResFinder database. In several instances, this is only 1 nucleotide and if sequencing errors arise, have the potential for misidentification. We can determine this accumulation of sequencing errors via observing the real-time emulation for DNA sequencing in Supplementary S5. After 5 hours (300 minutes), we could witness multiple genes being detected that were not identified in the final assembly and the Illumina only SPAdes assembly.

L245 (see also L218): Were there multiple fosA transcripts in the genome? I can't see from Table 1 any indication of this, but maybe it's not clear enough for me. If not, can you suggest other reasons for the low MAPQ score? It seems like a lot of results are being thrown away because the MAPQ is low.
Response: Only one copy of fosA is encoded on the chromosome for all isolates (Line 194). All genes with multiple copies have been noted in Line 215. The mapping quality is most likely due to the low expression of this gene and difficulties with base-calling (issues removing the long artificial poly(A) tail and interference of RNA modifications (Line 393). Once base-calling tools have been optimized for bacterial direct RNA sequencing, MAPQ scores will be a better quality.

L336 (see also L228 and L198): Would 80% identity lead to a misclassification by BWA-MEM?
Response: Yes, as some genes are very similar (potentially only one nucleotide difference), this has the potential to result in misclassification of resistance genes in the real-time emulation. Especially when we identified a 10% error rate in our ONT DNA sequencing (Line 356) and ≤23% for direct RNA sequencing (Line 394).

L341: I get a bit frustrated by people discussing accuracy from previous (typically quite old) nanopore papers as if it were a fixed thing, especially in a study that has produced a lot of other nanopore data. Nanopore technology changes quickly, and basecalling accuracy has made substantial improvements in particular over the last year. I'm not convinced a paper published in January 2018 would give a good estimate for accuracy called with guppy 3.0.3 (or 3.1.5, which is the latest that I'm aware of at the time of this review). Feel free to cite it, but I'd like to know [in the same breath] what the direct RNA accuracy was in *your* reads. L260-264 briefly discuss using different base-callers; how does that accuracy change depending on the base-caller?
Response: We have now included information regarding accuracy between base-callers: “Albacore 2.2.7 had the highest average accuracy across isolates (84.87%) closely followed by Guppy 3.0.3 (84.62%) and then Chiron v0.5 (78.19%) (Supplementary Table S6).” Line 279. The abstract also notes that we could identify accuracy up to 86% for direct RNA sequencing (Line 20).

** Figures **
Figure 1:
- Would work better as a side-by-side bar plot. The split graph makes it look like one side is negative, and the other side is positive.
- Order by colour / class rather tham abundance, with brackets indicating classifications.
Response: We initially considered side-by-side bar plots however, this would result in approximately 40 bars on the y-axis which is difficult to follow. We have now split the x-axis to better delineate between DNA and RNA data. Furthermore, an overlay of this data based on yield rather than time has been included in the supplementary results (Figure S4). The main text is written in the context of time to detect a particular gene conferring resistance to an antibiotic class, hence, why we ordered this as time of detection rather than grouping the antibiotic classes.

Figure 2:
- This figure is unclear to me. If this figure is relative expression (e.g. the statistic used for the correlation plot in Figure 3), then the presented data should be relative proportions, probably in log space (e.g. log2(gene/rpsL)).

Powered by Editorial Manager® and ProduXion Manager® from Aries Systems Corporation

- Why was rpsL chosen for normalisation?
Response: Unfortunately, the wrong figure legend was included for Figure 2 and has been amended. This data is counts per million (cpm) mapped reads rather than normalized to rpsL. We didn't adjust to relative proportions for this figure (or Figure 4, which is also in cpm) as the main text mentions cpm values. However, for comparisons of direct RNA to qRT-PCR (e.g. Figure 3 and Figure 5) we did normalize relative to housekeeping gene rpsL. This housekeeping gene has been used previously in literature (reference 46). We also have data for another housekeeping gene, rpoB, which generated similar results.

Figure 3:
- Were there any sample replicates? Are you able to estimate error in any measurements?
- The colour is confusing for this graph. You could try gene name for colour, and different plot symbols for different samples.
Response: All qRT-PCR measurements were done in triplicates (Line 170). There are no sample replicates for direct RNA sequence data. This is because the primary aim of the paper is to evaluate time-to-detection of antibiotic resistance genes across multiple samples (emulating a clinical setting in which a single replicate would be sequenced for each sample, particularly in the context of not having access to direct RNA multiplexing and so running a single sample in a single flow cell). However, we can estimate variation in the proportion of reads mapping to each gene (and hence the counts-per-million) by assuming the observed read counts are generated from a binomial distribution, so we can estimate a 90% CI in the expression levels using the conjugate beta prior. We show these estimates in Supplementary Figure S7.

Regarding the colours, there are 4 samples and eleven genes, so we didn't think colouring by gene would work (too many genes). We selected to colour by sample, and indicate the gene names on the plot. We have followed the suggestion of using different symbols per isolate.

Figure 4:
- What do the bottom panels describe (e.g. gene expression level scatter plots comparing each sample with each other sample)? This is not stated in the figure legend.
Response: Yes, the bottom panels include the expression levels between differing isolates in a scatter plot. This has now been added to the legend.

Figure 5:
- I recommend changing this to a side-by-side bar plot, as the text indicates that the comparison of A vs B is important.
Response: This figure has now been amended with the data on a single graph.

Reviewer #3:
The manuscript by Pitt et al interrogated the genome and transcriptome of PDR and XDR K. pneumoniae isolates using the Oxford Nanopore MinION device. This is the very first study which adopted nanopore approaches in direct bacterial mRNA sequencing. The authors established a methodology for adding poly(A) tail onto mRNA transcripts which will benefit future bacterial sequencing and diagnosis related studies. However, authors failed to explain clearly the advantage of using Nanopore for RNA sequencing to Illumina platform. In another word, why we need to develop RNA sequencing using Nanorpore since it is not an efficient way to do it and very complicated. In addition, the manuscript indeed showed that the coverage of RNA seq is very low and the correlation is not good. In my view, if there is no specific need to do RNA seq using Nanopore platform, there is no need to develop it since the Illumina platform is very good already in this application.
Response: Please refer to our first response to Reviewer #1.

In addition, I also have the following major comments:
1. Line 169, section "Antibiotic resistance and the location of acquired resistance in the genome "The authors reported the AMR genes and their location in this section. Since this is a technical manuscript, can the authors provide some sequencing information? The volume of data generated with time, coverage of each sequenced sample, the

Powered by Editorial Manager® and ProduXion Manager® from Aries Systems Corporation

	accuracy of the sequence, and the comparison of different assembly methods could be briefly discussed.<br>Response: We've now included additional information regarding the DNA sequencing: "MinION DNA sequencing for all isolates was run for ≥20 hours which generated 1.19 GB (215X) for 1_GR_13, 0.39 GB (67X) for 2_GR_12, 0.56 GB (101X) for 16_GR_13 and 0.64 GB (115X) for 20_GR_12 (Supplementary Table S2). Across the differing assembly tools, the chromosome sequence commonly circularised as a 5.0-5.4 Mb contig including plasmids ranging between 13-193 kb with the exception of 2_GR_12. Aligning ONT reads to the final assembly revealed that this DNA sequencing had a 90% accuracy rate across isolates." (Line 184) A comparison of several assembly methods is given in Supplementary Table 2, but we don't discuss this in much detail in the paper as it is not the focus of this work.<br><br>2. Line 256, only a low proportion of these RNA sequencing reads passed base-calling. Is it also related to the sample preparation apart from the inaccuracy of the base-calling software?<br>Response: Indeed, RNA sample preparation could influence the subsequent quality of the data and we attempted several protocol optimizations. We trialed phenol/ chloroform RNA extractions however, this process was lengthy and resulted in a low yield of RNA and increased impurities. The PureLink RNA Mini Kit protocol is relatively quick (<30 mins/ sample). We attempted an on-column DNase treatment during this protocol but the best DNA depletion was observed using TURBO DNase which doesn't work on column (requires 37°C incubation). Our optimized RNA extraction resulted in Bioanalyzer RNA integrity scores of ≥8.5 which has now been included in Line 116 (RIN scale 0-10, 10 is no degradation using 16S and 23S pecks as reference). Unfortunately, we were unaware of the efficiency of the polymerase until post sequencing analysis was performed (Supplementary Figure S6), hence, a shorter incubation can be implemented for future studies. However, the majority of inaccuracy appears to be due to the base-calling software unable to accurately trim the long artificial poly(A) tail and potential interference to the raw read signal via RNA modifications (Line 391).<br><br>3. Would the authors compare the genome and transcriptome a little bit to link these data?<br>Response: We have drawn various comparisons between the genome and transcriptome to link the sequencing data. In particular, tables and figures comparing both RNA and DNA include Figure 1, Table S5, Figure S3 and Figure S4 with corresponding sections in the main text. Additional information in the discussion has been provided to highlight the pros and cons regarding interpreting antibiotic resistance using either DNA or RNA. "We further investigated the transcriptome of these isolates to potentially elucidate the correlation between genotype and the subsequent resistant phenotype. Detection of antibiotic resistance via sequencing commonly uses DNA due to the instability of RNA and the lengthy sample processing such as rRNA depletion [12-15, 58]. However, RNA provides additional information regarding the functionality of genes such as identifying conditions in which a resistance gene is present but not active which gives rise to a false positive via DNA alone. Conversely, if expression is only induced in the presence of an antibiotic, the absence of RNA transcripts results in a false negative." (Line 367). "Furthermore, the time required to detect resistance may be hindered by the slower translocation speed associated with direct RNA sequencing (70 bases/ second) compared to DNA sequencing (450 bases/ second) [57]. Although cDNA would overcome this limitation, our findings show that detection was primarily due to level of expression when evaluating data yield rather than time." (Line 394).<br><br>4. Line 381, "a number of resistance genes were identified that were not present in the final assembly. The authors were expected to discuss why this happens and how to deal with these false positive data.<br>Response: The discussion on this topic has now been extended: "Furthermore, a small number of resistance genes were identified that were not present in the final assembly, however these all had MAPQ values less than 10 and less than 30 mapped reads. Some of these may be due to low-level kit contamination, while some of the false positives have sequence similarity to true positives and may be due to inaccuracies in base-calling." (Line 363).
**Additional Information:**	

Question	Response
Are you submitting this manuscript to a special series or article collection?	No
**Experimental design and statistics**<br><br>Full details of the experimental design and statistical methods used should be given in the Methods section, as detailed in our Minimum Standards Reporting Checklist. Information essential to interpreting the data presented should be made available in the figure legends.<br><br>Have you included all the information requested in your manuscript?	Yes
**Resources**<br><br>A description of all resources used, including antibodies, cell lines, animals and software tools, with enough information to allow them to be uniquely identified, should be included in the Methods section. Authors are strongly encouraged to cite Research Resource Identifiers (RRIDs) for antibodies, model organisms and tools, where possible.<br><br>Have you included the information requested as detailed in our Minimum Standards Reporting Checklist?	Yes
**Availability of data and materials**<br><br>All datasets and code on which the conclusions of the paper rely must be either included in your submission or deposited in publicly available repositories (where available and ethically appropriate), referencing such data using a unique identifier in the references and in the "Availability of Data and Materials" section of your manuscript.	Yes

Have you have met the above requirement as detailed in our Minimum Standards Reporting Checklist?	

Manuscript
Click here to view linked References
Click here to access/download;Manuscript;###GS_Evaluating the Resistome of XDR KP###_FINAL.docx

Evaluating the Genome and Resistome of Extensively Drug-Resistant *Klebsiella pneumoniae* using Native DNA and RNA Nanopore Sequencing

Miranda E. Pitt[1], Son H. Nguyen[1], Tânia P.S. Duarte[1], Haotian Teng[1], Mark A.T. Blaskovich[1], Matthew A. Cooper[1], Lachlan J.M. Coin[1]

[1] Institute for Molecular Bioscience, The University of Queensland, Brisbane, Queensland, 4072, Australia

Corresponding authors: Miranda Pitt (miranda.pitt@imb.uq.edu.au, ORCID: 0000-0002-8255-4036) and Lachlan Coin (l.coin@imb.uq.edu.au, ORCID: 0000-0002-4300-455X)

Abstract

Background: *Klebsiella pneumoniae* frequently harbours multidrug resistance and current diagnostics struggle to rapidly identify appropriate antibiotics to treat these bacterial infections. The MinION device can sequence native DNA and RNA in real-time, providing an opportunity to compare the utility of DNA and RNA for prediction of antibiotic susceptibility. However, the effectiveness of bacterial direct RNA sequencing and base-calling has not previously been investigated. This study interrogated the genome and transcriptome of four extensively drug-resistant (XDR) *K. pneumoniae* clinical isolates, however, further antimicrobial susceptibility testing identified three isolates as pandrug-resistant (PDR).

Results: The majority of acquired resistance (≥75%) resided on plasmids including several megaplasmids (≥100 kbp). DNA sequencing detected most resistance genes (≥70%) within 2 hours of sequencing. Neural-network based base-calling of direct RNA achieved up to 86% identity rate, although ≤23% of reads could be aligned. Direct RNA sequencing (with approximately 6 times slower pore translocation) was able to identify (within 10 hours) ≥35% of resistance genes, including those associated with resistance to aminoglycosides, β-lactams, trimethoprim and sulphonamide and also quinolones, rifampicin, fosfomycin and phenicol in some isolates. Polymyxin-resistant isolates showed a heightened transcription of *phoPQ* (≥2-fold) and the *pmrHFIJKLM* operon (≥8-fold). Expression levels estimated from direct RNA sequencing displayed strong correlation (Pearson: 0.86) compared to qRT-PCR across eleven resistance genes.

Conclusion: Overall, MinION sequencing rapidly detected the XDR/ PDR *K. pneumoniae* resistome and direct RNA sequencing provided accurate estimation of expression levels of these genes.

Introduction

Klebsiella pneumoniae is one of the leading causes of nosocomial infections, with reports of mortality rates as high as 50% [1-5]. This opportunistic pathogen commonly exhibits multidrug resistance which severely limits treatment options [6]. A high abundance of resistance is frequently encoded on plasmids, accounting for the rapid global dissemination of resistance [1,6]. Common therapeutic options for multidrug-resistant infections include carbapenems, fosfomycin, tigecycline and polymyxins [7]. However, resistance is also rapidly developing against these antibiotics resulting in the emergence of extensively drug-resistant (XDR) and subsequent pandrug-resistant (PDR) strains [6-9].

One of the major contributors to the advent of antibiotic resistance is the inability for current detection methodologies to readily and accurately assess bacterial infections in particular, the resistance profile [10]. Rapid sequencing has been proposed as a way to determine antibiotic resistance, including approaches which utilise high accuracy short reads, as well as those which exploit real-time single-molecule sequencing such as Oxford Nanopore Technologies (ONT). The ONT MinION platform is a portable single-molecule sequencer which can sequence long fragments of DNA and stream the sequence data for further data processing in real-time, detecting the presence of bacterial species and acquired resistance genes [11-15]. Moreover, the long reads coupled with the ability to multiplex samples has immensely aided with the assembly of bacterial genomes [16-18]. This capability allows for the rapid determination of whether resistance is residing on the chromosome or plasmid/s. Of particular interest are high levels of resistance encoded on plasmids, as these genes can rapidly be transferred throughout the bacterial population via horizontal gene transfer. However, a limitation of DNA sequencing is accurately identifying whether the presence of an acquired resistance gene or mutation is facilitating resistance.

ONT has recently released a direct RNA sequencing capability, which sequences native transcripts. Other sequencing technologies rely on fragmentation, cDNA conversion and PCR steps that create experimental bias and hinder the accuracy of determining gene expression [19, 20]. The ability for MinION sequencing to read long fragments enables full length transcripts to be investigated. To date, only a few direct RNA sequencing publications exist which include eukaryote transcriptomes, primarily yeast (*Saccharomyces cerevisiae* [19, 21]) and recently, *Homo sapiens* [22]. This sequencing has additionally been implemented in viral transcriptomics [23-25]. Only one prior study by Smith AM *et al.* has applied this sequencing to bacterial 16S ribosomal RNA (rRNA) to detect RNA modifications [26]. Notably, resistance to certain antibiotics, such as aminoglycosides, can arise via RNA modifications which are unable to be

detected once RNA is converted to cDNA [26]. Furthermore, library preparation time is halved for direct RNA sequencing due to the absence of cDNA synthesis. Bacterial transcription differs significantly from eukaryotes in that transcription and translation occur simultaneously. As a result, bacterial mRNA transcripts lack poly(A) tails and alternative splicing, however, genes can be co-transcribed if regulated via an operon [27]. The poly(A) tail is critical for the library preparation for ONT sequencing thus, we have established a methodology for adding this component onto transcripts.

In this study, we applied MinION sequencing to interrogate both the genome and the transcriptome (via direct RNA sequencing) for XDR *K. pneumoniae* clinical isolates. Of interest was to compare the potential for RNA sequencing to provide a better correlation to the resistance phenotype than DNA sequencing. These isolates have previously undergone 'traditional' whole genome sequencing (Illumina) and antimicrobial susceptibility testing [28]. An extended panel of antibiotics was tested in this study to identify PDR isolates. Three strains were selected from this cohort which exhibited resistance to all 24 antibiotics or antibiotic combinations tested, a high abundance of antibiotic resistance genes (≥26) and differing lineages (ST11 (16_GR_13), ST147 (1_GR_13) and ST258 (2_GR_12)). Additionally, these isolates harbour polymyxin resistance which is facilitated by a disruption in or upstream of *mgrB*. Variations in the *mgrB* gene result in increased expression of the *pmrCAB* and *pmrHFIJKLM* operon, enables the addition of phosphoethanolamine and/ or 4-amino-4-deoxy-L-arabinose (Ara4N) to lipid A and subsequently facilitates polymyxin resistance [29]. These pathways associated with polymyxin resistance were further explored using direct RNA sequencing and compared against a polymyxin-susceptible XDR isolate (ST258; 20_GR_12). This research aimed to assemble these genomes, discern expression of resistance genes and ascertain the time required for detection. Furthermore, we sought to compare DNA and RNA sequencing as modalities for the rapid identification of acquired antibiotic resistance.

Methods

Bacterial strains and growth conditions

XDR *K. pneumoniae* clinical strains were sourced through the Hygeia General Hospital, Athens, Greece [28]. Antimicrobial susceptibility assays (Supplementary Table S1), sequence typing and detection of acquired resistance genes have previously been determined [28]. Strains were stored at -80°C in 20% (v/v) glycerol, the identical stock was used as per the prior study and the extended panel of antimicrobial susceptibility testing conducted similarly [28].

When required for extractions, glycerol stocks were grown on lysogeny broth (LB) agar and 6 morphologically similar colonies were selected for inoculation. The inoculum was grown in LB overnight at 37°C shaking at 220 rpm. This overnight inoculum was used for both DNA and RNA extractions.

High molecular weight DNA isolation

DNA was extracted from 10 ml of overnight culture using the DNeasy Blood and Tissue Kit (Qiagen) according to manufacturer's guidelines, with the addition of an enzymatic lysis buffer pre-treatment (60 mg/ml lysozyme). Following the DNeasy extraction, high molecular weight (HMW) DNA was isolated using the MagAttract HMW DNA Kit (Qiagen) as per manufacturer's instructions. An additional proteinase K treatment at 56°C for 10 min followed by supplementation of RNase A (1 mg) for 15 min at room temperature was included to increase DNA purity. Several direct extractions from bacterial overnight cultures using the HMW kit were performed, however, low DNA yield was observed and the initial DNeasy extraction was essential. An additional purification step following the HMW DNA extraction was critical for 2_GR_12 as carbohydrate contamination (260/230 ratio: ≤0.3) was identified potentially due to a thickened capsule. This purification included the Monarch® PCR & DNA Cleanup Kit (New England BioLabs) using the protocol to isolate fragments >2000 bp.

RNA extraction, mRNA enrichment and poly(A) addition

The overnight inoculum was sub-cultured in 10 ml of cation-adjusted Muller Hinton Broth (caMHB) to reflect the media used for minimum inhibitory concentration (MIC) assays. Cultures were grown to mid-log phase (OD_{600} = 0.5-0.6). RNA was extracted via the PureLink™ RNA Mini Kit (Thermo Fisher Scientific) as per manufacturer's protocols which included using Homogenizer columns (Thermo Fisher Scientific). To remove DNA contamination, the TURBO DNA-free™ kit was implemented. A minor adjustment was an increased concentration of TURBO DNase (4 U) incubated at 37°C for 30 min. The RNeasy Mini Kit (Qiagen) clean up protocol was used to purify and concentrate RNA samples. Ribosomal RNA was depleted via the MICROB*Express*™ Bacterial mRNA Enrichment Kit (Thermo Fisher Scientific). Minor protocol changes included adding ≥2 µg of DNA depleted RNA and the enriched mRNA was precipitated for 3 h at -20°C. Poly(A) addition was performed using the Poly(A) Polymerase Tailing Kit (Astral Scientific) as per the manufacturer's alternative protocol (4 U input of Poly(A) Polymerase). The input RNA concentration was ≥800 ng and RNA samples were incubated at 37°C for 1 h. Poly(A) ligated RNA was purified using Agencourt AmpureXP (Beckman Coulter Australia) beads (1:1 ratio).

Extraction quality control

DNA and RNA were quantitated using Qubit®2.0 (Thermo Fisher Scientific) and purity determined with a NanoDrop 1000 Spectrophotometer (Thermo Fisher Scientific). DNA fragment sizes were measured using the Genomic DNA ScreenTape & Reagents (Agilent) and sizes from 200 to >60000 bp were analyzed on a 4200 TapeStation System (Agilent) (Supplementary Figure S1). RNA fragment size was checked using an Agilent RNA 6000 Pico kit and run on a 2100 Bioanalyzer (Agilent Technologies) for the initial RNA extract (RIN: ≥8.5), post ribosomal RNA depletion and after poly(A) tailing (Supplementary Figure S2).

ONT library preparation and sequencing

RNA libraries (≥600 ng poly(A)$^+$ RNA) were prepared using the Direct RNA Sequencing kit (SQK-RNA001). The Rapid Barcoding Sequencing kit (SQK-RBK001) was used for HMW DNA samples (1_GR_13, 16_GR_13, 20_GR_12; 300 ng input each). Isolate 2_GR_12 (300 ng input) was prepared separately using the Rapid Sequencing Kit (SQK-RAD003). Libraries were sequenced with MinION R9.4 flowcells and the raw data (fast5 files) were base-called using Albacore 2.1.1 for DNA sequencing (Supplementary Figure S3). For benchmarking purposes, RNA reads were additionally base-called with Albacore 2.2.7, Guppy 3.0.3 and the Chiron v0.5 [30] RNA base-caller which was trained in-house (https://github.com/haotianteng/Chiron/releases/tag/v0.5).

Real-time resistome detection emulation

The real-time emulation was performed post sequencing and the time required to detect antibiotic resistance was determined as previously described [14]. Briefly, this pipeline aligns Albacore base-called reads via BWA-MEM [31] to an antibiotic resistance gene database. Antibiotic resistance genes were obtained from the ResFinder 3.0 database [32]. This dataset comprises of 2131 genes which were clustered based on 90% identity to form 611 groups or gene families. The detection of false positives is reduced using the multiple sequence alignment software kalign2 [33], a probabilistic Finite State Machine [34] and once the alignment score reached a threshold, the resistance gene was reported.

Assembly of genomes

To assemble genomes with both Illumina and ONT reads, SPAdes v3.10.1 [35] was utilised. Hybrid assemblers included npScarf [36] and Unicycler v0.3.1 [37]. Assemblers using only ONT reads included Canu v1.5 (excluding reads <500bp) [38] and the combination of Minimap2 v2.1-r311 and Miniasm v0.2-r168-dirty; Racon (git commit 834442) were used in both cases to polish the assemblies [39, 40]. Consensus sequences were determined using Mauve (snapshot_2015-02-13) to construct the final assembly [41]. The output from each assembly software is reported in

Supplementary Table S2. Genomes were annotated using the Rapid Annotation using Subsystem Technology (RAST) which also provided a list of virulence genes [42]. The location of acquired antibiotic resistance genes were determined using ResFinder 3.0 [32] and plasmids were identified via PlasmidFinder 1.3 [43]. To discern if plasmid sequences have previously been reported, contigs underwent a BLASTn analysis against the National Center for Biotechnology Information (NCBI) database (https://blast.ncbi.nlm.nih.gov/Blast.cgi).

RNA alignment and expression profiling

Base-called RNA reads were converted to DNA (uracil bases changed to thymine) and aligned using BWA-MEM [31] to the updated genome assemblies. BWA-MEM was selected due to shorter transcripts being produced by bacteria (Supplementary Figure S3) and the lack of introns and alternative splicing. Similar parameters to the BWA-MEM ont2d function were used but seed length was reduced (-k 14) to compensate for shorter reads: -k 11 [minimum seed length, bp] -W20 [bandwidth] -r10 [gap extension penalty] -A1 [match score] -B1 [mismatch penalty] -O1 [Gap open penalty] -E1 [Gap extension penalty] -L0 [Clipping penalty]). Multi-mapping reads were removed via SAMtools (secondary alignment: flagged as 256) [44] and BEDTools coverage [45] was used to ascertain the expression of resistance genes in counts per million (cpm) mapped reads (post removing reads mapping to rRNA). To compare against qRT-PCR results, read counts were normalised the housekeeping gene, *rpsL* [46]. Read alignments were further visualised using Integrative genomics viewer 2.3.59 [47].

Whole transcriptome gene expression and estimation of expression confidence intervals

We identified genes which were differentially expressed in one sample (versus all remaining samples) using a quasi-likelihood F-test in EdgeR [48] with a FDR threshold of 0.01. Expression levels (in cpm) were extracted for every significant gene in any one of these one versus remaining differential expression analyses in order to generate an expression heatmap. The expression heatmap is based on the log10(cpm) for each of these genes.

In order to estimate the 90% confidence intervals in cpm estimates from direct RNA sequence data, we assumed that the observed counts were generated from a binomial distribution with unobserved probability of success (p). We estimate the 5% and 95% percentiles from a beta-distribution with shape parameters equal to the number of reads mapped to a given gene (alpha) and the number of reads mapped elsewhere (beta) plus a pseudo-count of 0.1. The 90% confidence interval (CI) is calculated as the difference between the expression levels at the 5% and 95% percentile.

Quantitative real-time reverse transcriptase PCR (qRT-PCR)

First strand cDNA synthesis was performed on 1 µg of total DNA-depleted RNA using SuperScript III (Thermo Fisher Scientific). Primers used are displayed in Supplementary Table S3. Samples were prepared in triplicate via the SYBR Select Master Mix (Thermo Fisher Scientific) and expression detected using a ViiA 7 Real-time PCR system (Thermo Fisher Scientific). Cycling conditions include: Hold 50°C (2 min), 95°C (2 min) followed by 50 cycles of: 95°C (15 sec), 55°C (1 min). A melt curve was included to determine the specificity of the amplification and a no template control to detect contamination or primer dimers. Results were analysed with QuantStudioTM Real-Time PCR Software, triplicates were averaged, normalised to the housekeeping gene *rpsL* [46] and relative expression determined via the $2^{-\Delta\Delta CT}$ method [49].

Results

Antibiotic resistance and the location of acquired resistance in the genome

This study assayed nine additional antibiotics or antibiotic combinations to further characterise the phenotypic resistance of these isolates (Supplementary Table S1). Strains 1_GR_13, 2_GR_12 and 16_GR_13 were non-susceptible to all antibiotics including the 24 antibiotics tested previously [28]. 20_GR_12 was only susceptible to gentamicin and polymyxins.

MinION DNA sequencing for all isolates was run for ≥20 hours which generated 1.19 GB (215X) for 1_GR_13, 0.39 GB (67X) for 2_GR_12, 0.56 GB (101X) for 16_GR_13 and 0.64 GB (115X) for 20_GR_12 (Supplementary Table S2). Across the differing assembly tools, the chromosome sequence commonly circularised as a 5.0-5.4 Mb contig including plasmids ranging between 13-193 kb with the exception of 2_GR_12. Aligning ONT reads to the final assembly revealed that DNA sequencing had 90% accuracy across isolates.

Utilising the capacity for MinION sequencing to read long fragments of DNA, the location of antibiotic resistance genes were clearly resolved (Table 1). All genomes were circular with the exception of 2_GR_12 where 3 plasmids remained linear. This was partly due to difficulties extracting DNA, not retaining long fragments and subsequently, lower coverage of the genome (Supplementary Figure S1, Table S2). Amongst the four isolates, the chromosome size ranged between 5.1-5.5 Mb which encoded resistance genes *blaSHV-11*, *fosA* and *oqxAB*. The majority of resistance (≥75%) mapped to plasmids.

At least one megaplasmid, defined as a plasmid larger than 100 kbp, was detected in all isolates (Table 1). These commonly harboured the replicon IncA/C2 or InFIB and IncFIIK. The IncA/C2 plasmid was present in all samples

except 20_GR_12. This plasmid contained up to 16 resistance genes which conferred resistance towards aminoglycosides, β-lactams, phenicols, rifampicin, sulphonamides, tetracyclines and trimethoprim, with the exception of 16_GR_13. Isolate 16_GR_13 lacked trimethoprim resistance on its IncA/C2 plasmid. The plasmids containing both replicons IncFIB and IncFIIK differed vastly between all four replicates. All contained IncFIB$_{pKpn3}$ and IncFIIK, however, 1_GR_13 differed with IncFII$_{pKP91}$. Additionally, a differing IncFIB replicon was detected on a separate contig in 1_GR_13 (pKPHS1) and 2_GR_12 (pQil). The only instance where another dual replicon was identified was in 1_GR_13 which harboured both IncR and IncN. This plasmid contained aminoglycoside, β-lactam, trimethoprim, macrolide and sulphonamide resistance. 1_GR_13 also contained a 5.5 kb circular contig which was annotated as a phage genome. Various regions of these megaplasmids were unique to these isolates compared to prior sequences deposited on NCBI (Supplementary Table S5).

The ColRNAI plasmid was present in all except 1_GR_13 which encoded aminoglycoside and quinolone resistance (*aac(6')-Ib, aac(6')-Ib-cr)* (Table 1). The ColRNAI plasmid in 2_GR_12 and 20_GR_12 was 13841 bp in size and shared 75% similarity between the two isolates. This plasmid differed in 16_GR_13 which contained no resistance genes and 35% the size. The same IncX3 plasmid (43380 bp) was apparent in isolates 2_GR_12 and 20_GR_12. Unique to 16_GR_13 was the IncL/ M$_{pOXA-48}$ plasmid containing *blaOXA-48* and the 50979 bp IncN plasmid in 20_GR_12 with resistance against 5 classes (aminoglycoside (*aph(3'')-Ib*, *aph(6)-Id*), β-lactam (*blaTEM-1A*), sulphonamide (*sul2*), tetracycline (*tet(A)*), trimethoprim (*dfrA14*)) of antibiotics.

Multiple copies of acquired resistance genes were apparent across plasmids in several isolates. For 1_GR_13, up to three copies were present of genes *aadA24*, *aph(3')-Ia*, *aph(6)-Id, dfrA1, dfrA14, strA* and *sul1* (Table 1). In 2_GR_12, *sul1* and *blaTEM-1A* were duplicated and for 16_GR_13, only *sul1* was represented twice.

Real-time detection emulation of resistance genes via DNA sequencing

The vast majority (≥70%) of resistance genes were detected via DNA sequencing within the first 2 hours (Figure 1, Supplementary Table S5). These genes confer resistance towards aminoglycosides, β-lactams, fosfomycin, macrolides, phenicols, quinolones, rifampicin, sulphonamides, tetracyclines and trimethoprim. 20_GR_12 lacked acquired resistance genes for macrolides, phenicols and rifampicin, however, all other classes were detected within 2 hours. All isolates, except 2_GR_12, were sequenced for 21 hours which was sufficient to obtain the complete genome assembly. Only a few additional genes were detected after the first 10 hours across isolates (Supplementary Table S5). For 2_GR_12, an extended run of 41 hours detected no further genes after 20 hours. Overall, the presence of these

resistance genes corresponded to a resistant phenotype towards aminoglycosides, β-lactams, fosfomycin, phenicols, quinolones, sulphonamides (sulfamethoxazole), tetracyclines and trimethoprim (Supplementary Table S1). As macrolides and rifampicin are not routinely used to treat *K. pneumoniae* infections, no breakpoints exist according to CLSI and EUCAST guidelines, however, all isolates exhibit an MIC ≥128 μg/ml towards erythromycin (macrolide) and ≥64 μg/ml for rifampicin (Supplementary Table S1).

Post 2 hours of sequencing, several genes not observed in the final assembly via ResFinder 3.0 were detected (Supplementary Table S5). These were predominantly genes attributed to aminoglycoside, β-lactam, rifampicin and phenicol resistance. Furthermore, resistance genes to additional classes were detected including fusidic acid and vancomycin. This was evident in 2_GR_12 (*fusB*) and 16_GR_13 (*fusB*, *vanR*). However, these genes had less than 30 reads and their phred-scale mapping quality (MAPQ) scores were less than 10 (misplaced probability greater than 0.1). Furthermore, the majority of genes not observed in the final assembly nor observed in Illumina data exhibited a MAPQ score of ≤10 which may indicate that a more stringent threshold is required to negate false positives. However, if this threshold increases, true positives would not be detected including *aadA1*, *aadA2* and *ARR-2* in 2_GR_12 and *blaOXA-48*, *blaCTX-M-15* and *ARR-2* in 16_GR_13.

Several genes found in the final assembly were not detected in the real-time emulation analysis (Supplementary Table S5). This was mainly observed for aminoglycoside resistance encoding genes. For 1_GR_13, this included *aadA1, ant(2'')-Ia*, *aph(6)-Id* and *aadA24.* Similarly, 2_GR_12 and 20_GR_12 lacked *aph(3'')-Ib* and *aph(6)-Id.* 2_GR_12 additionally had the absence of *ant(2'')-Ia.* Detection of *ant(2'')-Ia*, *aph(3'')-Ib*, *aph(6)-Id* was not present in 16_GR_13. 16_GR_13 further lacked *catB4* (phenicol) and *tet(A)* (tetracycline). Various phenicol resistance genes were reported in the real-time emulation however, the incorrect gene was identified which may represent sequencing errors accumulated over time and high similarity to other phenicol resistance genes. The tetracycline resistance gene, *tet(A),* was interestingly not reported in this emulation with 190 reads and the majority of reads exhibiting a high mapping confidence (MAPQ = 60, equivalent to an error probability of $1x10^{-6}$). This gene was only detected after 10 hours for 1_GR_13 and 2_GR_12 and this result may be influenced by the presence of only 1 copy of *tet(A)* encoded on a low copy number megaplasmid (between 1 to 1.5, see Table 1).

Direct RNA sequencing resistance detection

The time required to detect resistance was further interrogated using RNA sequencing. Rapid detection was possible for several resistance genes via direct RNA sequencing (Figure 1). This was evident for genes conferring resistance

to aminoglycosides, β-lactams, sulphonamides and trimethoprim for all four isolates. Resistance towards these antibiotics was commonly detected within 6 hours. In some instances, quinolone, rifampicin, fosfomycin and phenicol resistance was detected. A similar result was obtained whether all reads or passed reads alone were analysed. The most significant difference when analysing all reads was the detection of *fosA* in 1_GR_13 and *ARR-2* and *fosA* in 2_GR_12. Consistently absent from this analysis were genes attributed to macrolide (*mph(A)*) and tetracycline (*tet(A), tet(G)*) resistance, however, isolates exhibited high levels of resistance to tetracycline (>64 μg/ml) (Supplementary Table S1). Commonly no new genes were detected after 12 hours of sequencing with the exception of *fosA* in 2_GR_12. Although *fosA* was detected when including the failed reads, a low MAPQ score (≤10) was apparent. Similar to the DNA real-time detection, several genes not found in the final assembly were identified (Supplementary Table S5). With the exception of 20_GR_12, this included *aadB* and *strB* for all isolates. Additional genes detected included *ARR-7* in 1_GR_13, *strA* in 2_GR_12 and for 16_GR_13, *blaCTX-M-64*, *blaOXA-436* and *strA.* Similar genes or gene families were identified when comparing DNA and direct RNA sequencing. Overall, genes were detected more readily via DNA sequencing however, there were a few instances where RNA sequencing detected resistance quicker: *aac(3')-IIa* in 16_GR_13 and *sul2* and *catA1* in 2_GR_12. Similar results were observed when investigating data yield rather than time which compensates for the slower translocation speed associated with direct RNA sequencing (Supplementary Figure S4).

Levels of expression of resistance genes

RNA sequencing accumulated over approximately 40 hours yielded between 0.9 and 1.7 million reads for these isolates (Supplementary Figure S3). However, only a low proportion (≤14.64%) of these reads passed base-calling using Albacore 2.2.7 (Supplementary Table S6). Aligning passed reads alone to the final assembly, ≥98% of reads were mappable, however, ≤40% of these had a MAPQ score ≥10. When all reads (pass and fail) were aligned, the majority were not mappable to the reference genome (≥76.69%) and commonly exhibited a low MAPQ score (≤10). Low mapping quality could be attributed to assignment of reads to multiple copies of genes in the genome. Furthermore, the ONT error rates could lead to misassignment of reads to genes. In light of this, we decided to benchmark a number of different base-callers, including Albacore 2.2.7, Guppy 3.03 as well as Chiron v0.5 which was trained in-house (Supplementary Table S6, Figure S5). Chiron base-called more reads compared to Albacore 2.2.7 and Guppy 3.0.3, however, fewer reads aligned to the reference genome and had a slightly lower identity rate. Albacore 2.2.7 had the highest average accuracy across isolates (84.87%) closely followed by Guppy 3.0.3 (84.62%) and then

Chiron v0.5 (78.19%) (Supplementary Table S6). These results reflect the fact that base-calling algorithms have not yet been optimised for direct RNA sequencing, and even less so for bacterial RNA sequencing. The poly(A) length was commonly found to be approximately 400 to 700 bp across isolates (Supplementary Figure S6). Taking into consideration the Albacore 2.2.7 base-called reads, a proportion of these reads were found to map to rRNA including 1_GR_13 (18%), 2_GR_12 (37%), 16_GR_13 (24%) and 20_GR_12 (23%). Overall, at least 58% of genes (with at least 1 read mapping to the gene) were identified to be expressed across isolates (1_GR_13 (68%), 2_GR_12 (58%), 16_GR_13 (75%) and 20_GR_12 (69%).

Amongst the four isolates, levels of expression for resistance genes on the chromosome (*blaSHV-11*, *fosA* and *oqxAB*) were low (≤122 counts per million mapped reads) (Figure 2). The remaining resistance genes were located on plasmids. Resistance genes exhibiting high levels of expression (300 cpm) were apparent in 1_GR_13 (*blaTEM-1B*, *blaVIM-27*, *sul1*, *aph(3')-Ia*), 2_GR_12 (*aac(6')-Ib*, *catA1*, *blaKPC-2*), 16_GR_13 (*aac(6')Ib-cr*, *aac(3)-IIa*, *blaCTX-M-15*, *blaTEM-1B*, *blaOXA-48*) and 20_GR_12 (*blaKPC-2*, *aac(6')Ib*). Counts for *aac(6')-1b* and *aac(6')-1b-cr* in 2_GR_12 and 20_GR_12 were grouped. The gene *aac(6')-1b-cr* is a shortened version of *aac(6')-1b* and both were identified in the same genome position, hence, only *aac(6')-1b* is displayed in Figure 2. Expression estimates did not differ significantly when analysing passed reads alone or all reads. We estimated the 90% confidence interval in cpm estimates using a beta-distribution (Supplementary Figure S7). All highly expressed genes were detected within 6 hours as per the real-time detection emulation. As anticipated, low levels of expression were observed for fosfomycin (*fosA*), tetracycline (*tet(A)*, *tet(B)*) and macrolide (*mph(A)*) resistance.

A subset of 11 resistance genes which represent resistance across various classes of antibiotics were investigated to validate gene expression in these RNA extractions via qRT-PCR (Figure 3). These included resistance towards aminoglycosides (*aac(6')Ib*, *strA*), β-lactams (*blaKPC-2*, *blaOXA-10*, *blaTEM-1*), phenicols (*cmlA1*), trimethoprim (*dfrA14*), fosfomycin (*fosA*), quinolone (*oqxA*), sulphonamides (*sul2*) and tetracyclines (*tet(A)*). A similar trend was observed between direct RNA sequencing and qRT-PCR results (Spearman's rank correlation coefficient: 0.83; Pearson correlation: 0.86) (Figure 3). The highest expression of a resistance gene was observed for *blaKPC-2* although only one copy was present in a lower copy number plasmid in 2_GR_12 and 20_GR_12 (Figure 2, Figure 3 and Table 1). Additionally, low levels of expression for *fosA* and *tet(A)* were apparent despite exhibiting resistance towards fosfomycin and tetracycline (Figure 3, Supplementary Table S1). Direct RNA sequencing was unable to detect low levels of expression whilst qRT-PCR could detect these genes (Figure 3).

Across the transcriptome, antibiotic resistance genes were identified to harbour high expression between isolates (Figure 4). Virulence genes were comparable across these strains similar to all remaining or background genes. The top differentially expressed genes were determined (Supplementary Figure S8) and several were associated with polymyxin resistance pathways. Heightened expression was seen in polymyxin-resistant isolates 1_GR_13, 2_GR_12, 16_GR_13 in comparison to the single susceptible isolate (20_GR_12) in particular, genes associated with Ara4N synthesis. These genes include 4-deoxy-4-formamido-L-arabinose-phosphoundecaprenol deformylase (*arnD*), UDP-4-amino-4-deoxy-L-arabinose formyltransferase and UDP-4-amino-4-deoxy-L-arabinose-oxoglutarate aminotransferase.

Transcriptional biomarkers for polymyxin resistance

Three of the isolates harboured resistance towards polymyxins via disruptions in *mgrB* which included 1_GR_13, 2_GR_12 and 16_GR_13. 1_GR_13. Isolate 1_GR_13 has an insertion sequence (IS) element, IS*Kpn26*-like, at nucleotide position 75 in the same orientation as *mgrB* whilst 2_GR_12 has this IS element in the opposite orientation plus additional mutations in *phoP* (A95S) and *phoQ* (N253T). 16_GR_13 harbours an IS element, IS*1R*-like, 19 bp upstream of *mgrB*. Direct RNA sequencing revealed only low-level expression of *mgrB* (1_GR_13 (78.4 cpm), 2_GR_12 (16.3 cpm), 16_GR_13 (0 cpm), 20_GR_12 (2.3 cpm)). The expression levels of various genes associated with this pathway were verified via qRT-PCR (Figure 5). Direct RNA sequencing revealed a slight increase in transcription of *phoPQ* (≥2-fold) relative to 20_GR_12. A ≥13-fold increase in expression was observed for *pmrH* and ≥8-fold elevation for *pmrK.* Similar trends for expression were also reported using qRT-PCR (Figure 5).

Discussion

XDR *K. pneumoniae* infections pose as a major threat to modern medicine. A rapid diagnostic would help to guide appropriate treatment options [1, 6]. The MinION sequencing technology employed in this study has potential to detect antibiotic resistance in a timely manner. Three of the four *K. pneumoniae* isolates examined in this study harboured non-susceptibility to all antibiotics or antibiotic combinations assayed, and hence would be classified as PDR according to published guidelines [50]. ONT sequencing was able to resolve both the assembly of plasmids harbouring high levels of resistance (through DNA sequencing) and the expression from the resistome in the absence of antibiotic treatment (via RNA sequencing).

The ability for ONT to sequence long fragments of DNA has significantly aided the assembly of bacterial genomes and plasmids [16-18]. In this study, multiple megaplasmids (≥100 kbp) were identified which were previously unresolved via Illumina sequencing [28]. These harboured replicons IncA/C2 or a dual replicon, IncFIIK and IncFIB. The IncA/C, IncF and IncN plasmids have been commonly associated with multidrug resistance [51]. Although several plasmids in this study revealed similarity to previously reported isolates via NCBI, various sequences deviated. In particular, the IncA/C2 plasmid exhibited multiple regions unique to these isolates. Several IncA/C2 megaplasmids have been previously described which harbour various resistance genes. However, the extent of resistance observed in our study is extreme when compared to prior reports [52, 53]. Prior studies have shown the IncFIIK and IncFIB replicons to localise on the same plasmid and also megaplasmids with multidrug resistance [6]. The $IncFIB_{pQil}$ plasmid in this study contained various β-lactam resistance genes (*blaKPC-2*, *blaOXA-9*, *blaTEM-1A*) which has been identified previously [54]. Similarly, *blaOXA-48* segregated with the IncL/M replicon [55,56], however, deviations in this plasmid were identified.

The real-time analysis capability entailed in MinION sequencing has the potential to rapidly determine antibiotic resistance profiles of pathogenic bacteria. Previously this device has been utilised to assemble bacterial genomes, discern species and detect antibiotic resistance [12-15]. This study investigated the potential time required to discern resistance via a real-time emulation as previously described [14]. The majority (≥70%) of resistance genes were detected via DNA sequencing within 2 hours. Several genes not identified in the final assembly were detected after 2 hours of sequencing. This may be attributed to the high similarity (≥80%) amongst various genes, in particular, those associated with aminoglycoside, β-lactam, rifampicin and phenicol resistance. Furthermore, the error rate associated with ONT sequencing, and the accumulation of these errors over time, may result in the false annotation of these genes. Nanopore DNA sequencing currently has an accuracy ranging from 85 to 95% (90% in our study), which limits its ability to detect genomic variations [17, 57]. Several resistance genes only differ by a few nucleotides which significantly impacts the resistance phenotype and the antibiotics which can be utilised to treat these infections. However, software tools such as Nanopolish (https://github.com/jts/nanopolish) and Tombo (https://github.com/nanoporetech/tombo) (similarly used to re-train Chiron v0.5 for direct RNA sequencing data) have the potential to correct these reads and would be helpful to integrate to increase the accuracy of detecting resistance genes. We utilised native DNA sequencing in this study which retains epigenetic modifications such methylation which can hinder the accuracy of reads and subsequent calling of antibiotic resistance [58]. Furthermore, a small

number of resistance genes were identified that were not present in the final assembly, however these all had MAPQ values less than 10 and less than 30 mapped reads. Some of these may be due to low-level kit contamination, while some of the false positives have sequence similarity to true positives and may be due to inaccuracies in base-calling.

We further investigated the transcriptome of these isolates to potentially elucidate the correlation between genotype and the subsequent resistant phenotype. Detection of antibiotic resistance via sequencing commonly uses DNA due to the instability of RNA and the lengthy sample processing such as rRNA depletion [12-15, 58]. However, RNA provides additional information regarding the functionality of genes such as identifying conditions in which a resistance gene is present but not active which gives rise to a false positive via DNA alone. Conversely, if expression is only induced in the presence of an antibiotic, the absence of RNA transcripts results in a false negative. This study grew *K. pneumoniae* strains in the absence of antibiotic and direct RNA sequencing revealed high levels of transcription from genes associated with aminoglycoside, β-lactam, sulphonamide and trimethoprim resistance within 6 hours of our study. In particular, the highest levels of expression were observed for the β-lactamase gene *blaKPC-2* in 2_GR_12 and 20_GR_12. Alterations in the promoter region have previously been reported to influence high levels of expression [59]. Notably, the promoter or operon (co-transcribed genes) can largely influence expression of genes. The detection of quinolone, rifampicin, and phenicol resistance correlated to the levels of transcription within samples. All isolates exhibited low levels of expression for fosfomycin, macrolide and tetracycline resistance, despite exhibiting phenotypic resistance to fosfomycin and tetracycline [28]. FosA, an enzyme involved in fosfomycin degradation, is commonly encoded chromosomally in *K. pneumoniae* and a combination of expression and enzymatic activity contributes to resistance [60]. Noteably, Klontz *et al* identified that chromosomally integrated FosA, similarly observed in our study, from *K. pneumoniae* harboured a higher catalytic efficiency. A higher catalytic efficiency may reason why our strains only require a low abundance of expression and still retain fosfomycin resistance. Genes *tet(A)* and *tet(G)* encode efflux pumps which, in the absence of tetracycline, are lowly expressed and the lack of antibiotic supplementation in this study confirms this observation [61]. Detecting inducible resistance (antibiotic exposure required for gene expression) such as tetracycline resistance highlights one of the advantages of investigating the transcriptome.

There are several other variables to consider when interpreting expression levels in bacterial RNA sequencing data. These include the extent prior exposure to antibiotics in the clinic alters transcription and the copy number of resistance genes and the plasmids these are encoded on. Limitations were observed when base-calling bacterial direct RNA

sequencing and may be attributed to trimming the long artificial poly(A) tail and interference of RNA modifications. This entailed an increased error rate of ≤23% across base-callers (12% identified in a prior study [21]) and a poor alignment rate ≤23%. Furthermore, the time required to detect resistance may be hindered by the slower translocation speed associated with direct RNA sequencing (70 bases/ second) compared to DNA sequencing (450 bases/ second) [57]. Our findings show that the slower time-to-detection of resistance genes in direct RNA sequencing was due to both the level of expression as well as the slower translocation speed, and hence using cDNA would only partially overcome this limitation

We also investigated pathways attributed to polymyxin resistance. Three of these strains exhibited an IS element upstream of within *mgrB*, the negative regulator of PhoPQ [29]. Elevated expression was apparent for *phoPQ* and also the *pmrHFIJKLM* operon in our polymyxin-resistant isolates harbouring a disruption in *mgrB*. This has previously been witnessed for other *K. pneumoniae* isolates harbouring *mgrB* disruptions and is a potential transcriptional marker for polymyxin resistance [29, 46, 62, 63]. However, this study is limited to four isolates and one mechanism associated with polymyxin resistance. Other pathways have previously been identified including the role of other two component regulatory systems such as CrrAB [64]. The ability to use relative expression of key genes to detect polymyxin resistance requires further validation, including an increased sample size of resistant and non-resistant isolates. Furthermore, additional functional experiments such as complementation assays would be required in order to validate the contribution of a certain mutation to the transcriptome and subsequent resistance.

Conclusions

This study has utilised MinION sequencing to assemble four XDR *K. pneumoniae* genomes and has revealed several unique plasmids harbouring multidrug resistance. The vast majority of this resistance was detectable within 2 hours of sequencing. Exploiting this analysis in real-time would allow for a rapid diagnostic, however, the presence of a resistance gene does not necessarily indicate resistance is conferred and requires additional phenotypic characterisation. This research also established a methodology and analysis for bacterial direct RNA sequencing. The expression of resistance genes were successfully detected via this technology and can be exploited for bacterial transcriptomics. Once base-calling algorithms have been optimised, this could allow for a whole transcriptome interrogation of full-length transcripts regulated by operons, where more than one gene is co-expressed in a transcript, and the evaluation of the poorly characterised RNA modifications. Overall, this study has begun to unravel the

association between genotype, transcription and subsequent resistant phenotype in these XDR/ PDR *K. pneumoniae* clinical isolates, establishing the groundwork for developing a diagnostic that can rapidly determine bacterial resistance profiles.

Availability of supporting data

The datasets supporting the results presented here are available in the National Center for Biotechnology Information repository BioProject PRJNA307517 (www.ncbi.nlm.nih.gov/bioproject/PRJNA307517). ONT DNA sequencing data has been deposited on the Sequence Read Archive (www.ncbi.nlm.nih.gov/sra/) under study SRP133040. Accession numbers are as follows: 1_GR_13 (SRR6747887), 2_GR_12 (SRR6747886), 16_GR_13 (SRR6747885) and 20_GR_12 (SRR6747884). ONT direct RNA sequencing data (pass and fail reads) have been deposited on the Sequence Read Archive (www.ncbi.nlm.nih.gov/sra/) under study SRP133040. Accession numbers are as follows: 1_GR_13 (SRR7719054), 2_GR_12 (SRR7719055), 16_GR_13 (SRR7719052) and 20_GR_12 (SRR7719053).

Abbreviations

Ara4N: 4-amino-4-deoxy-L-arabinose; caMHB: cation-adjusted Muller Hinton Broth; CLSI: Clinical & Laboratory Standards Institute; CI: Confidence interval; cpm: counts per million; EUCAST: The European Committee on Antimicrobial Susceptibility Testing; FDR: False discovery rate; HMW: High molecular weight; IS: Insertion sequence; LB: Lysogeny broth; MAPQ: Mapping quality; MIC: Minimum inhibitory concentration; NCBI: National Center for Biotechnology Information; ONT: Oxford Nanopore Technologies; PDR: Pandrug-resistant; RAST: Rapid Annotation using Subsystem Technology; rRNA: Ribosomal RNA; XDR: Extensively drug-resistant.

Competing Interests

The authors declare that there are no competing interests.

Funding

LJMC is an NHMRC career development Fellow APP1103384. MAC is an NHMRC Principal Research Fellow (APP1059354) and currently holds a fractional Professorial Research Fellow appointment at the University of Queensland with his remaining time as CEO of Inflazome Ltd. a company headquartered in Dublin, Ireland that is developing drugs to address clinical unmet needs in inflammatory disease by targeting the inflammasome. MEP is an Australian Postgraduate Award scholar. MATB is supported in part by a Wellcome Trust Strategic Award

104797/Z/14/Z. This work was supported by the Institute for Molecular Bioscience Centre for Superbug Solutions (610246).

Author Contributions

MEP, LJMC, MATB and MAC conceived this study. MEP, SHN and HT performed the sequencing analysis. Laboratory work was carried out by MEP and TPSD. MEP wrote the paper with input from all authors.

Acknowledgements

We would like to acknowledge Dr Ilias Karaiskos and Dr Helen Giamarellou for providing the bacterial strains in this study. We also acknowledge Dr Evangelos Bellos for his guidance on the RNA sequencing analysis and Dr Devika Ganesamoorthy for the initial advice on the direct RNA sequencing library preparation. We would like to acknowledge Dr Intawat Nookaew for providing yeast direct RNA sequence data.

References

1. Martin RM, Bachman MA. Colonization, Infection, and the Accessory Genome of *Klebsiella pneumoniae*. *Front Cell Infect Microbiol.* 2018;8:4.

2. Magill SS, Edwards JR, Bamberg W, et al. Multistate point-prevalence survey of health care-associated infections *N Engl J Med*. 2014;370:1198-208.

3 Kalanuria AA, Ziai W, Mirski, M. Ventilator-associated pneumonia in the ICU. *Crit Care*. 2014;18:208.

4 Talha KA, Hasan Z, Selina F, et al. Organisms associated with ventilator associated pneumonia in intensive care unit. *Mymensingh Med J*. 2009;18:S93-7.

5 Podschun R, Ullmann U. *Klebsiella spp* as nosocomial pathogens: epidemiology, taxonomy, typing methods, and pathogenicity factors. *Clin Microbiol Rev*. 1998;11:589-603.

6 Navon-Venezia S, Kondratyeva K, Carattoli A. *Klebsiella pneumoniae*: a major worldwide source and shuttle for antibiotic resistance. *FEMS Microbiol Rev*. 2017;41:252-75.

7 Karaiskos I, Giamarellou H. Multidrug-resistant and extensively drug-resistant Gram-negative pathogens: current and emerging therapeutic approaches. *Expert Opin Pharmacother*. 2014;15:1351-70.

8 Chen L, Todd R, Kiehlbauch J, et al. Notes from the Field: Pan-Resistant New Delhi Metallo-Beta-Lactamase-Producing *Klebsiella pneumoniae* - Washoe County, Nevada, 2016 *MMWR Morb Mortal Wkly Rep*. 2017;66:33.

9 Zowawi HM, Forde BM, Alfaresi M, et al. Stepwise evolution of pandrug-resistance in *Klebsiella pneumoniae*. *Sci Rep*. 2015;5:15082.

10 Sommer MOA, Munck C, Toft-Kehler RV, et al. Prediction of antibiotic resistance: time for a new preclinical paradigm? *Nat Rev Microbiol*. 2017;15:689-96.

11 Gardy JL, Loman NJ. Towards a genomics-informed, real-time, global pathogen surveillance system. *Nat Rev Genet*. 2018;19:9-20.

12 Lemon JK, Khil PP, Frank KM, et al. Rapid Nanopore Sequencing of Plasmids and Resistance Gene Detection in Clinical Isolates. *J Clin Microbiol*. 2017;55:3530-43.

13 Votintseva AA, Bradley P, Pankhurst L, et al. Same-Day Diagnostic and Surveillance Data for Tuberculosis via Whole-Genome Sequencing of Direct Respiratory Samples. *J Clin Microbiol*. 2017;55:1285-98.

14 Cao MD, Ganesamoorthy D, Elliott AG, et al. Streaming algorithms for identification of pathogens and antibiotic resistance potential from real-time MinION^{TM} sequencing. *Gigascience*. 2016;5:32.

15 Quick J, Ashton P, Calus S, et al. Rapid draft sequencing and real-time nanopore sequencing in a hospital outbreak of *Salmonella*. *Genome Biol*. 2015;16:114.

16 Wick RR, Judd LM, Gorrie CL, et al. Completing bacterial genome assemblies with multiplex MinION sequencing. *Microb Genom*. 2017;3:e000132.

17 Li R, Xie M, Dong N, et al. Efficient generation of complete sequences of MDR-encoding plasmids by rapid assembly of MinION barcoding sequencing data. *Gigascience*. 2018;7:1-9.

18 George S, Pankhurst L, Hubbard A, et al. Resolving plasmid structures in Enterobacteriaceae using the MinION nanopore sequencer: assessment of MinION and MinION/Illumina hybrid data assembly approaches. *Microb Genom*. 2017;3:e000118.

19 Garalde, DR, Snell, EA, Jachimowicz, D, et al. Highly parallel direct RNA sequencing on an array of nanopores. *Nat Methods*. 2018;15:201-6.

20 Ozsolak F, Milos PM. RNA sequencing: advances, challenges and opportunities. *Nat Rev Genet*. 2011;12: 87-98.

21 Jenjaroenpun P, Wongsurawat T, Pereira R, et al. Complete genomic and transcriptional landscape analysis using third-generation sequencing: a case study of *Saccharomyces cerevisiae* CENPK113-7D. *Nucleic Acids Res*. 2018;46:e38.

22 Workman RE, Tang A, Tang PS, et al. Nanopore native RNA sequencing of a human poly(A) transcriptome. *bioRxiv*. 2018;459529.

23 Moldovan N, Tombacz D, Szucs A, et al. Third-generation Sequencing Reveals Extensive Polycistronism and Transcriptional Overlapping in a *Baculovirus*. *Sci Rep*. 2018;8:8604.

24 Keller MW, Rambo-Martin BL, Wilson MM, et al. Direct RNA Sequencing of the Coding Complete Influenza A Virus Genome. *Sci Rep*. 2018;8:14408.

25 Depledge DP, Srinivas KP, Sadaoka T, et al. Direct RNA sequencing on nanopore arrays redefines the transcriptional complexity of a viral pathogen. *Nat Commun*. 2019;10:754.

26 Smith AM, Jain M, Mulroney L, et al. Reading canonical and modified nucleotides in 16S ribosomal RNA using nanopore direct RNA sequencing. *Plos One*. 2019;14:e0216709.

27 Sorek R, Cossart P. Prokaryotic transcriptomics: a new view on regulation, physiology and pathogenicity. *Nat Rev Genet*. 2010;11:9-16.

28 Pitt ME, Elliott AG, Cao, MD, et al. Multifactorial chromosomal variants regulate polymyxin resistance in extensively drug-resistant *Klebsiella pneumoniae*. *Microb Genom*. 2018;4:mgen1090000158.

29 Olaitan AO, Morand S, Rolain JM. Mechanisms of polymyxin resistance: acquired and intrinsic resistance in bacteria. *Front Microbiol*. 2014;5:643.

30 Teng H, Cao MD, Hall MB, et al. Chiron: translating nanopore raw signal directly into nucleotide sequence using deep learning. *GigaScience*. 2018;7:10.1093/gigascience/giy037.

31 Li H. Aligning sequence reads, clone sequences and assembly contigs with BWA-MEM. *arXiv*. 2013;13033997.

32 Zankari E, Hasman H, Cosentino S, et al. Identification of acquired antimicrobial resistance genes. *J Antimicrob Chemother*. 2012;67:2640-4.

33 Lassmann T, Frings O, Sonnhammer EL. Kalign2: high-performance multiple alignment of protein and nucleotide sequences allowing external features. *Nucleic Acids Res*. 2009;37:858-65.

34 Allison L, Wallace CS, Yee CN. When is a string like a string? In: Artificial Intelligence and Mathematics. *Ft Lauderdale FL*. 1990.

35 Bankevich A, Nurk S, Antipov D, et al. SPAdes: a new genome assembly algorithm and its applications to single-cell sequencing. *J Comput Biol*. 2012;19:455-77.

36 Cao MD, Nguyen SH, Ganesamoorthy D, et al. Scaffolding and completing genome assemblies in real-time with nanopore sequencing. *Nat Commun*. 2017;8:14515.

37 Wick RR, Judd LM, Gorrie CL, et al. Unicycler: Resolving bacterial genome assemblies from short and long sequencing reads. *PLoS Comput Biol*. 2017;13:e1005595.

38 Koren S, Walenz BP, Berlin K, et al. Canu: scalable and accurate long-read assembly via adaptive k-mer weighting and repeat separation. *Genome Res*. 2017;27:722-36.

39 Li H. Minimap and miniasm: fast mapping and *de novo* assembly for noisy long sequences. *Bioinformatics*. 2016;32:2103-10.

40 Vaser R, Sovic I, Nagarajan N, et al. Fast and accurate *de novo* genome assembly from long uncorrected reads. *Genome Res*. 2017;27:737-46.

41 Darling AE, Tritt A, Eisen JA, et al. Mauve assembly metrics. *Bioinformatics*. 2011;27:2756-7.

42 Aziz RK, Bartels D, Best AA, et al. The RAST Server: rapid annotations using subsystems technology. *BMC Genomics*. 2008;9:75.

43 Carattoli A, Zankari E, Garcia-Fernandez A, et al. *In silico* detection and typing of plasmids using PlasmidFinder and plasmid multilocus sequence typing. *Antimicrob Agents Chemother*. 2014;58:3895-903.

44. Li H, Handsaker B, Wysoker A, et al. The sequence alignment/map format and SAMtools. *Bioinformatics*. 2009;25:2078-9.

45 Quinlan AR. BEDTools: The Swiss-Army Tool for Genome Feature Analysis. *Curr Protoc Bioinformatics*. 2014;47:11.12.1-34.

46 Cannatelli A, D'Andrea MM, Giani T, et al. *In vivo* emergence of colistin resistance in *Klebsiella pneumoniae* producing KPC-type carbapenemases mediated by insertional inactivation of the PhoQ/PhoP *mgrB* regulator. *Antimicrob Agents Chemother*. 2013;57:5521-6.

47 Robinson JT, Thorvaldsdottir H, Winckler W, et al. Integrative genomics viewer. *Nat Biotechnol*. 2011;29; 24-6.

48. Robinson MD, McCarthy DJ, Smyth GK. edgeR: a Bioconductor package for differential expression analysis of digital gene expression data. *Bioinformatics*. 2010;26:139-40.

49 Livak KJ, Schmittgen TD. Analysis of relative gene expression data using real-time quantitative PCR and the 2(-Delta Delta C(T)) Method. *Methods*. 2001;25:402-8.

50 Magiorakos AP, Srinivasan A, Carey RB, et al. Multidrug-resistant, extensively drug-resistant and pandrug-resistant bacteria: an international expert proposal for interim standard definitions for acquired resistance. *Clin Microbiol Infect.* 2012;18,268–81.

51 Carattoli A. Resistance plasmid families in Enterobacteriaceae. *Antimicrob Agents Chemother*. 2009;53: 2227-38.

52 Desmet S, Nepal S, van Dijl JM, et al. Antibiotic Resistance Plasmids Cointegrated into a Megaplasmid Harboring the *blaOXA-427* Carbapenemase Gene. *Antimicrob Agents Chemother*. 2018;62:e01448-17.

53 Papagiannitsis CC, Dolejska M, Izdebski R, et al. Characterisation of IncA/C2 plasmids carrying an In416-like integron with the *blaVIM-19* gene from *Klebsiella pneumoniae* ST383 of Greek origin. *Int J Antimicrob Agents*. 2016;47:158-62.

54 Chen L, Chavda KD, Melano RG, et al. Comparative genomic analysis of KPC-encoding pKpQIL-like plasmids and their distribution in New Jersey and New York Hospitals. *Antimicrob Agents Chemother*. 2014;58:2871-7.

55 Poirel L, Bonnin RA, Nordmann P. Genetic features of the widespread plasmid coding for the carbapenemase OXA-48. *Antimicrob Agents Chemother*. 2012;56:559-62.

56 Potron A, Poirel L, Nordmann P. Derepressed transfer properties leading to the efficient spread of the plasmid encoding carbapenemase OXA-48. *Antimicrob Agents Chemother*. 2014;58:467-71.

57 Rang FJ, Kloosterman WP, de Ridder J. From squiggle to basepair: computational approaches for improving nanopore sequencing read accuracy. *Genome Biol*. 2018;19:90.

58 Tamma PD, Fan Y, Bergman Y, et al. Applying rapid whole-genome sequencing to predict phenotypic antimicrobial susceptibility testing results among carbapenem-reistant *Klebsiella pneumoniae* clinical isolates. *Antimicrob Agents Chemother*. 2018;63: pii: e01923-18.

59 Cheruvanky A, Stoesser N, Sheppard AE, et al. Enhanced *Klebsiella pneumoniae* Carbapenemase Expression from a Novel Tn4401 Deletion. *Antimicrob Agents Chemother*. 2017;61:e00025-17.

60 Klontz EH, Tomich AD, Gunther S, et al. Structure and Dynamics of FosA-Mediated Fosfomycin Resistance in *Klebsiella pneumoniae* and *Escherichia coli*. *Antimicrob Agents Chemother*. 2017;61:e01572-17.

61 Saenger W, Orth P, Kisker C, et al. The Tetracycline Repressor-A Paradigm for a Biological Switch. *Angew Chem Int Ed Engl*. 2000;39;2042-52.

62 Cheng YH, Lin TL, Pan YJ, et al. Colistin resistance mechanisms in *Klebsiella pneumoniae* strains from Taiwan. *Antimicrob Agents Chemother*. 2015;59:2909-13.

63 Haeili M, Javani A, Moradi J, et al. MgrB Alterations Mediate Colistin Resistance in *Klebsiella pneumoniae* Isolates from Iran. *Front Microbiol*. 2017;8:2470.

64 Baron S, Hadjadj L, Rolain JM, et al. Molecular mechanisms of polymyxin resistance: knowns and unknowns. *Int J Antimicrob Agents*. 2017;48:583-91.

Table and Figure Legends

Table 1: Final assembly of XDR *K pneumoniae* isolates and location of antibiotic resistance genes

Isolate	ST	Contig	Length (bp)	Coverage	Contig ID*	Resistance Genes**
1_GR_13	147	1	**5181675**	1	C	*blaSHV-11, fosA, oqxA, oqxB*
		2	**192771**	1.95	P: IncA/C2	*aadA1, ant(2'')-Ia, aph(6)-Id, ARR-2, blaOXA-10, blaTEM-1B, blaVEB-1, cmlA1, dfrA14, dfrA23, rmtB, strA, sul1, sul2, tet(A), tet(G)*
		3	**168873**	2	P: IncFIB$_{pKpn3}$, IncFII$_{pKP91}$	*aadA24, aph(3')-Ia, aph(6)-Id, dfrA1, dfrA14, strA*
		4	**108879**	1.53	P: IncFIB$_{pKPHS1}$	-
		5	**55018**	14.10	-	-
		6	**53495**	2.36	P: IncR, IncN	*aadA24, aph(3')-Ia, aph(6)-Id, blaVIM-27, dfrA1, mph(A), strA, sul1*
2_GR_12	258	1	**5466424**	1	C	*blaSHV-11, fosA, oqxA, oqxB*
		2	197872	1.3	P: IncFIB$_{pKpn3}$, IncFIIK	*aadA2, aph(3')-Ia, catA1, dfrA12, mph(A), sul1*
		3	175636	1.49	P: IncA/C2	*aadA1, ant(2'')-Ia, aph(3'')-Ib, aph(6)-Id, ARR-2, blaOXA-10, blaTEM-1A, blaVEB-1, cmlA1, dfrA14, dfrA23, rmtB, sul1, sul2, tet(A), tet(G)*
		4	95481	1.61	P: IncFIB$_{pQil}$	*blaKPC-2, blaOXA-9, blaTEM-1A*
		5	**43380**	1.91	P: IncX3	*blaSHV-12*
		6	**13841**	4	P: ColRNAI	*aac(6')-Ib, aac(6')Ib-cr*
16_GR_13	11	1	**5426917**	1	C	*blaSHV-11, fosA, oqxA, oqxB*
		2	**187670**	0.88	P: IncFIB$_{pKpn3}$; IncFIIK	*aac(3)-IIa, aac(6')Ib-cr, aadA2, aph(3')-Ia, blaCTX-M-15, blaOXA-1, catB4, dfrA12, mph(A), sul1*
		3	**155161**	0.99	P: IncA/ C2	*aadA1, ant(2'')-Ia, aph(3'')-Ib, aph(6)-Id, ARR-2, blaOXA-10, blaTEM-1B, blaVEB-1, cmlA1, rmtB, sul1, sul2, tet(A), tet(G)*
		4	**63589**	1.49	P: IncL/ M$_{pOXA-48}$	*blaOXA-48*
		5	**5234**	188.49	-	-
		6	**4940**	97.77	P: ColRNAI	-
20_GR_12	258	1	**5395894**	1	C	*blaSHV-11, fosA, oqxA, oqxB*
		2	**170467**	1.77	P: IncFIB$_{pKpn3}$; IncFIIK	*aph(3')-Ia, blaKPC-2, blaOXA-9, blaTEM-1A*
		3	**50979**	1.42	P: IncN	*aph(3'')-Ib, aph(6)-Id, blaTEM-1A, dfrA14, sul2, tet(A)*
		4	**43380**	1.78	P: IncX3	*blaSHV-12*
		5	**13841**	10.82	P: ColRNAI	*aac(6')-Ib, aac(6')Ib-cr*

*Contig ID represents chromosome (C) or plasmid (P): replicon determined via PlasmidFinder 1.3.

Resistance genes identified using ResFinder 3.0 (≥90% sequence similarity, ≥60% minimum length) and displayed in alphabetical order. **Bold indicates a circular contig.

Figure 1: Time required to detect antibiotic resistance genes via the real-time emulation analysis using MinION DNA and direct RNA sequencing. (**A**) 1_GR_13, (**B**) 2_GR_12, (**C**) 16_GR_13 and (**D**) 20_GR_12. Legend colours identify the class of antibiotic to which the gene confers resistance, / on y-axis indicates reads detected more than one resistance gene and # is a family of genes detected (>3). An asterisk (*) indicates the inability for direct RNA sequencing to detect this gene. Albacore 2.2.7. base-called sequences were used and all reads (pass and fail) were included in this analysis.

Figure 2: Direct RNA sequencing expression of resistance genes aligned to completed genomes expressed as counts per million mapped reads (post removal of reads mapping to rRNA). (**A**) 1_GR_13, (**B**) 2_GR_12, (**C**) 16_GR_13 and (**D**) 20_GR_12. X-axis depicts the resistance genes and are grouped based on the location in the genome where P indicates a plasmid followed by replicon identity. Albacore 2.2.7 base-called pass and fail reads were used for analysis. Dotted line is set to 300 cpm.

Figure 3: Correlation between resistance genes detected via direct RNA sequencing and validated using qRT-PCR. Relative expression was calculated via normalizing to the housekeeping gene, *rpsL* for both direct RNA sequencing (log2(gene/*rpsL*)) and qRT-PCT ($2^{-\Delta\Delta CT}$). Due to high similarity between certain genes, several primers recognise more than one gene. These include *aac(6')Ib: aac(6')Ib-cr, aadA24*; *strA: aph(3'')-Ib* and *blaTEM-1: blaTEM-1A, blaTEM-1B.*

Figure 4: Correlation between the four XDR *K pneumoniae* isolates for gene expression via direct RNA sequencing. Top panels display spearman correlation coefficients. The diagonal panel shows the density of gene expression levels in counts per million mapped reads for each sample (post removal of rRNA mapped reads). Bottom panels depict the correlation of gene expression between isolates as a scatter plot. Colours indicate categorization of gene: antimicrobial resistance genes (AMR) as per ResFinder 3.0, virulence genes (VIR) determined via RAST and all other genes or background genes (BG) are displayed. Cpm was capped at 2000.

Figure 5: Expression of genes associated with the polymyxin resistance pathway. Comparison between direct RNA sequencing (solid shapes without asterisk) and qRT-PCR (solid shapes with asterisk). Direct RNA sequencing data is calculated as log2(gene/ *rpsL*) and qRT-PCR as gene/ *rpsL*. All isolates except 20_GR_12 harboured resistance to polymyxin (MIC: >2 µg/mL). The bars indicate the average of qRT-PCR and direct RNA sequencing.

Manuscript_Markup

Click here to access/download;Manuscript;###GS_Evaluating the Resistome XDR KP###_MU_FINAL.docx

Click here to view linked References

Evaluating the Genome and Resistome of Extensively Drug-Resistant *Klebsiella pneumoniae* using Native DNA and RNA Nanopore Sequencing

Miranda E. Pitt[1], Son H. Nguyen[1], Tânia P.S. Duarte[1], Haotian Teng[1], Mark A.T. Blaskovich[1], Matthew A. Cooper[1], Lachlan J.M. Coin[1]

[1] Institute for Molecular Bioscience, The University of Queensland, Brisbane, Queensland, 4072, Australia

Corresponding authors: Miranda Pitt (miranda.pitt@imb.uq.edu.au, ORCID: 0000-0002-8255-4036) and Lachlan Coin (l.coin@imb.uq.edu.au, ORCID: 0000-0002-4300-455X)

Abstract

Background: *Klebsiella pneumoniae* frequently harbours multidrug resistance and current diagnostics struggle to rapidly identify appropriate antibiotics to treat these bacterial infections. The MinION device can sequence native DNA and RNA in real-time, providing an opportunity to compare the utility of DNA and RNA for prediction of antibiotic susceptibility. However, the effectiveness of bacterial direct RNA sequencing and base-calling has not previously been investigated. This study interrogated the genome and transcriptome of four extensively drug-resistant (XDR) *K. pneumoniae* clinical isolates, however, further antimicrobial susceptibility testing identified three isolates as pandrug-resistant (PDR).

Results: The majority of acquired resistance (≥75%) resided on plasmids including several megaplasmids (≥100 kbp). DNA sequencing detected most resistance genes (≥70%) within 2 hours of sequencing. Neural-network based base-calling of direct RNA achieved up to 86% identity rate, although ~~only~~ ≤23% of reads could be aligned. Direct RNA sequencing (with approximately 6 times slower pore translocation) was able to identify (within 10 hours) ≥35% of resistance genes, including those associated with resistance to aminoglycosides, β-lactams, trimethoprim and sulphonamide and also quinolones, rifampicin, fosfomycin and phenicol in some isolates. Polymyxin-resistant isolates showed a heightened transcription of *phoPQ* (≥2-fold) and the *pmrHFIJKLM* operon (≥8-fold). Expression levels estimated from direct RNA sequencing displayed strong correlation (Pearson: 0.86) compared to qRT-PCR across eleven resistance genes.

Conclusion: Overall, MinION sequencing rapidly detected the XDR/ PDR *K. pneumoniae* resistome and direct RNA sequencing provided~~revealed~~ ~~differential~~ accurate estimation of expression levels of these genes.

Introduction

Klebsiella pneumoniae is one of the leading causes of nosocomial infections, with reports of mortality rates as high as 50% [1-5]. This opportunistic pathogen commonly exhibits multidrug resistance which severely limits treatment options [6]. A high abundance of resistance is frequently~~commonly~~ encoded on plasmids, accounting for the rapid global dissemination of resistance [1,6]. Common therapeutic options for multidrug-resistant infections include carbapenems, fosfomycin, tigecycline and polymyxins [7]. However, resistance is also rapidly developing against these antibiotics resulting in the emergence of extensively drug-resistant (XDR) and subsequent pandrug-resistant (PDR) strains [6-9].

One of the major contributors to the advent of antibiotic resistance is the inability for current detection methodologies to readily and accurately assess bacterial infections in particular, the resistance profile [10]. Rapid sequencing has been proposed as a way to determine antibiotic resistance, including approaches which utilise high accuracy short reads, as well as those which exploit real-time single-molecule sequencing such as Oxford Nanopore Technologies (ONT). The ONT MinION platform is a portable single-molecule sequencer which can sequence long fragments of DNA and stream the sequence data for further data processing in real-time, detecting the presence of bacterial species and acquired resistance genes [11-15]. Moreover, the long reads coupled with the ability to multiplex samples has immensely aided with the assembly of bacterial genomes [16-18]. This capability allows for the rapid determination of whether resistance is residing on the chromosome or plasmid/s. Of particular interest are high levels of resistance encoded on plasmids, as these genes can rapidly be transferred throughout the bacterial population via horizontal gene transfer. However, a limitation of DNA sequencing is accurately identifying whether the presence of an acquired resistance gene or mutation is facilitating resistance.

ONT has recently released a direct RNA sequencing capability, which sequences native transcripts. Other sequencing technologies rely on fragmentation, cDNA conversion and PCR steps that create experimental bias and hinder the accuracy of determining gene expression [19, 20]. The ability for MinION sequencing to read long fragments enables full length transcripts to be investigated. To date, only a few direct RNA sequencing publications exist which include eukaryote transcriptomes, primarily yeast (*Saccharomyces cerevisiae* [19, 21]) and recently, *Homo sapiens* [22]. This sequencing has additionally been implemented in viral transcriptomics [23-25]. Only one prior study by Smith AM *et al*. has applied this sequencing to bacterial 16S ribosomal RNA (rRNA) to detect RNA~~epigenetic~~ modifications [26]. Notably, resistance to certain antibiotics, such as aminoglycosides, can arise via RNA modifications which are unable

to be detected once RNA is converted to cDNA [26]. Furthermore, library preparation time is halved for direct RNA sequencing due to the absence of cDNA synthesis. Bacterial transcription differs significantly from eukaryotes in that transcription and translation occur simultaneously. As a result, bacterial mRNA transcripts lack poly(A) tails and alternative splicing, however, genes can be co-transcribed if regulated via an operon [27]. The poly(A) tail is critical for the library preparation for ONT sequencing thus, we have established a methodology for adding this component onto transcripts.

Formatted: Not Highlight

In this study, we applied MinION sequencing to interrogate both the genome and the transcriptome (via direct RNA sequencing) for XDR *K. pneumoniae* clinical isolates. Of interest was to compare the potential for RNA sequencing to provide a better correlation to the resistance phenotype than DNA sequencing. These isolates have previously undergone 'traditional' whole genome sequencing (Illumina) and antimicrobial susceptibility testing [28]. An extended panel of antibiotics was tested in this study to identify PDR isolates. Three strains were selected from this cohort which exhibited resistance to all 24 antibiotics or antibiotic combinations tested, a high abundance of antibiotic resistance genes (≥26) and differing lineages (ST11 (16_GR_13), ST147 (1_GR_13) and ST258 (2_GR_12)). Additionally, these isolates harbour polymyxin resistance which is facilitated by a disruption in or upstream of *mgrB*. Variations in the *mgrB* gene result in increased expression of the *pmrCAB* and *pmrHFIJKLM* operon, enables the addition of phosphoethanolamine and/ or 4-amino-4-deoxy-L-arabinose (Ara4N) to lipid A and subsequently facilitates polymyxin resistance [29]. These pathways associated with polymyxin resistance were further explored using direct RNA sequencing and compared against a polymyxin-susceptible XDR isolate (ST258; 20_GR_12). This research aimed to assemble these genomes, discern the differential expression of resistance genes and ascertain the time required for detection. Furthermore, we sought to compare DNA and RNA sequencing as modalities for the rapid identification of acquired antibiotic resistance.

Methods

Bacterial strains and growth conditions

XDR *K. pneumoniae* clinical strains were sourced through the Hygeia General Hospital, Athens, Greece [28]. Antimicrobial susceptibility assays (Supplementary Table S1), sequence typing and detection of acquired resistance genes have previously been determined [28]. Strains were stored at -80°C in 20% (v/v) glycerol, the identical stock was used as per the prior study and the extended panel of antimicrobial susceptibility testing conducted similarly [28].

When required for extractions, glycerol stocks were grown on lysogeny broth (LB) agar~~ plates~~ and 6 morphologically similar colonies were selected for inoculation. The inoculum was grown in LB overnight at 37°C shaking at 220 rpm. This overnight inoculum was used for both DNA and RNA extractions.

H~~DNA extraction and h~~igh molecular weight DNA isolation

DNA was extracted from 10 ml of overnight culture using the DNeasy Blood and Tissue Kit (Qiagen) according to manufacturer's guidelines, with the addition of an enzymatic lysis buffer pre-treatment (60 mg/ml lysozyme). Following the DNeasy extraction, h~~H~~igh molecular weight (HMW) DNA was isolated~~from the prior extraction was selected~~ using the MagAttract HMW DNA Kit (Qiagen) as per manufacturer's instructions. ~~Subtle changes included~~An additional~~ a further~~ proteinase K treatment ~~on the DNA extracts ~~at 56°C for 10 min followed by supplementation of RNase A (1 mg) for 15 min at room temperature was included to increase DNA purity. Several direct extractions from bacterial overnight cultures using the HMW kit were performed, however, low DNA yield was observed and the initial DNeasy extraction was essential. An additional purification step following the HMW DNA extraction was critical for 2_GR_12 as ~~Several attempts at direct DNA extraction from bacterial cells were undertaken using the MagAttract HMW DNA kit, however, were unsuccessful with these isolates. Due to several issues with potential ~~carbohydrate contamination (260/230 ratio: ≤0.3) was identified potentially due to a thickened capsule. This purification included ~~, 2_GR_12 was also purified with ~~the Monarch® PCR & DNA Cleanup Kit (New England BioLabs) using the protocol to isolate fragments >2000 bp.

~~DNA and RNA contamination was quantitated using Qubit®2.0 (Thermo Fisher Scientific) and purity determined with a NanoDrop 1000 Spectrophotometer (Thermo Fisher Scientific). DNA fragment sizes were determined using the Genomic DNA ScreenTape & Reagents (Agilent) and sizes from 200 to >60000 bp were analyzed on a 4200 TapeStation System (Agilent) (Supplementary Figure S1).~~

RNA extraction, mRNA enrichment and poly(A) addition

The overnight inoculum~~culture~~ was sub-cultured in 10 ml of cation-adjusted Muller Hinton Broth (caMHB) to reflect the media~~conditions~~ used for minimum inhibitory concentration (MIC) assays. Cultures were grown to mid-log phase (OD_{600} = 0.5-0.6). RNA was extracted via the PureLink™ RNA Mini Kit (Thermo Fisher Scientific) as per manufacturer's protocols which included using Homogenizer columns (Thermo Fisher Scientific). To remove DNA contamination, the TURBO DNA-free™ kit was implemented. A minor adjustment was an increased concentration of TURBO DNase (4 U) incubated at 37°C for 30 min. The RNeasy Mini Kit (Qiagen) clean up protocol was

additionally used to purify and concentrate RNA samples. Ribosomal RNA was depleted via the MICROB*Express*™ Bacterial mRNA Enrichment Kit (Thermo Fisher Scientific). Minor protocol changes included adding ≥2 µg of DNA depleted RNA and the enriched mRNA was precipitated for 3 h at -20°C. Poly(A) addition was performed using the Poly(A) Polymerase Tailing Kit (Astral Scientific) as per the manufacturer's alternative protocol (4 U input of Poly(A) Polymerase). The input RNA concentration was ≥800 ng and RNA samples were incubated at 37°C for 1 h. Poly(A) ligated RNA was purified using Agencourt AmpureXP (Beckman Coulter Australia) beads (1:1 ratio). RNA and DNA contamination was quantitated using the Qubit®2.0 (ThermoFisher Scientific) and purity determined with a NanoDrop 1000 Spectrophotometer (Thermo Fisher Scientific). RNA fragment size was checked using an Agilent RNA 6000 Pico kit and run on a 2100 Bioanalyzer (Agilent Technologies) for the initial RNA extract, post ribosomal RNA depletion and after poly(A) ligation (Supplementary Figure S2).

Extraction quality control

DNA and RNA contamination wereas quantitated using Qubit®2.0 (Thermo Fisher Scientific) and purity determined with a NanoDrop 1000 Spectrophotometer (Thermo Fisher Scientific). DNA fragment sizes were measureddetermined using the Genomic DNA ScreenTape & Reagents (Agilent) and sizes from 200 to >60000 bp were analyzed on a 4200 TapeStation System (Agilent) (Supplementary Figure S1).

RNA and DNA contamination was quantitated using the Qubit®2.0 (ThermoFisher Scientific) and purity determined with a NanoDrop 1000 Spectrophotometer (Thermo Fisher Scientific). RNA fragment size was checked using an Agilent RNA 6000 Pico kit and run on a 2100 Bioanalyzer (Agilent Technologies) for the initial RNA extract (RIN: ≥8.5), post ribosomal RNA depletion and after poly(A) tailingligation (Supplementary Figure S2).

Formatted: Font: Not Bold, Not Italic

Formatted: Justified

ONT library preparation and sequencing

RNA libraries (≥600 ng poly(A)+ ligated RNA) were prepared using the Direct RNA Sequencing kit (SQK-RNA001). The Rapid Barcoding Sequencing kit (SQK-RBK001) was used for HMW DNA samples (1_GR_13, 16_GR_13, 20_GR_12; 300 ng input each). Isolate 2_GR_12 (300 ng input) was prepared separately using the Rapid Sequencing Kit (SQK-RAD003). Libraries were sequenced with MinION R9.4 flowcells and the raw data (fast5 files) were base-called using Albacore 2.1.1 for DNA sequencing (Supplementary Figure S3). For benchmarking purposes, RNA reads were additionally base-called with Albacore 2.2.7, Guppy 3.0.3 and the Chiron v0.5 [30] RNA base-caller which was trained in-house (https://github.com/haotianteng/Chiron/releases/tag/v0.5).

Real-time resistome detection emulation

The real-time emulation was performed post sequencing and the time required to detect antibiotic resistance was determined as previously described [14]. Briefly, this pipeline aligns Albacore base-called reads via BWA-MEM [31] to an antibiotic resistance gene database. Antibiotic resistance genes were obtained from the ResFinder 3.0 database [32]. This dataset comprises of 2131 genes which were clustered based on 90% identity to form 611 groups or gene families. The detection of false positives is reduced using the multiple sequence alignment software kalign2 [33], a probabilistic Finite State Machine [34] and once the alignment score reached a threshold, the resistance gene was reported.

Assembly of genomes

To assemble genomes with both Illumina and ONT reads, SPAdes v3.10.1 [35] was utilisedimplemented. Hybrid assemblers included npScarf [36] and Unicycler v0.3.1 [37]. Assemblers using only ONT reads included Canu v1.5 (excluding reads <500bp) [38] and the combination of Minimap2 v2.1-r311 and Miniasm v0.2-r168-dirty; Racon (git commit 834442) were used in both cases to polish the assemblies [39, 40]. Consensus sequences were determined using Mauve (snapshot_2015-02-13) to construct the final assembly [41]. The output from each assembly software is reported in Supplementary Table S2. Genomes were annotated using the Rapid Annotation using Subsystem Technology (RAST) which also provided a list of virulence genes [42]. The location of acquired antibiotic resistance genes were determined using ResFinder 3.0 [32] and plasmids were identified via PlasmidFinder 1.3 [43]. To discern if plasmid sequences have previously been reported, contigs underwent a BLASTn analysis against the National Center for Biotechnology Information (NCBI) database (https://blast.ncbi.nlm.nih.gov/Blast.cgi).

RNA alignment and expression profiling

Base-called RNA reads were converted to DNA (uracil bases changed to thymine) and aligned using BWA-MEM [31] to the updated genome assemblies. BWA-MEM was selected due to shorter transcripts being produced by bacteria (Supplementary Figure S3) and the lack of introns and alternative splicing. Similar parameters to the BWA-MEM ont2d function were used(but seed length was reduced (-k 14) to compensate for shorter reads:parameters: -k 11 [minimum seed length, bp] -W20 [bandwidth] -r10 [gap extension penalty] -A1 [match score] -B1 [mismatch penalty] -O1 [Gap open penalty] -E1 [Gap extension penalty] -L0 [Clipping penalty] -Y). to the updated genome assemblies. Multi-mapping reads were removed via SAMtools (secondary alignment: flagged as 256) [44]- and to the updated genome assemblies. Due to the lack of introns and full length transcripts being obtained, BEDTools coverage

Formatted: Not Highlight

[454] was used to ascertain the relative expression of resistance genes in counts per million (cpm) mapped reads (post removing reads mapping to rRNA). To compare against qRT-PCR results, read counts were normalised This was normalized to the number of counts obtained for the housekeeping gene, *rpsL* [465]., to compare against qRT-PCR results or counts per million mapped reads (post removing reads mapping to rRNA). Read alignments were further visualised using Integrative genomics viewer 2.3.59 [467].

Whole transcriptome differential gene expression and estimation of expression confidence intervals.

We identified genes which were differentially expressed in one sample (versus all remaining samples) using a quasi-likelihood F-test in EdgeR [48] with a FDR threshold of 0.01. -Expression levels (in cpmounts per million) were extracted for every significant gene in any one of these one versuss remaining differential expression analyses in order to generate an expression heatmap. –The expression heatmap is based on the log10(cpm) for each of these genes.

Formatted: Not Highlight

In order to estimate the 90% confidence intervals in cpmounts per million estimates from direct RNA sequence data, we assumed that the observed counts were generated from a binomial distribution with unobserved probability of success (p). -We estimate the 5% and 95% percentiles from a beta-distribution with shape parameters equal to the number of reads mapped to a given gene (alpha) and the number of reads mapped elsewhere (beta) plus a pseudo-count of 0.1. –The 90% confidence interval (CI) is calculated as the difference between the expression levels at the 5% and 95% percentile.—To identify genes which were differentially expressed between a pair of samples (x and y), we used a beta-binomial distribution to calculate the probability of observing less than or equal to x_g reads mapping to gene g in sample x, conditional on the total number of reads mapping to all genes (sum_g(x_g)), the number of reads in sample y mapping to gene g (y_g) as well as the total number of reads mapping to all genes in sample y (sum_g (y_g). This was calculated in R using the pbetabinom.ab function in the VGAM package, with q = x_g, size = sum_g'(x_g'), alpha = y_g +1; beta = sum_g'(y_g') - y(g) +1. Genes for which this probability was less than a predefined threshold were deemed to be significantly under expressed in sample x given sample y. A similar statistic was used to check for over-expression.

Quantitative real-time reverse transcriptase PCR (qRT-PCR)

First strand cDNA synthesis to generate cDNA was performed on (1 μg of total DNAase-depleted RNA) was performed using SuperScript III (Thermo Fisher Scientific).- which was also used for MinION direct RNA sequencing library preparations. Primers used are displayed in Supplementary Table S3. Samples were prepared in triplicate via the SYBR Select Master Mix (Thermo Fisher Scientific) and expression detected using a ViiA 7 Real-time PCR

system (Thermo Fisher Scientific). Cycling conditions include: Hold 50°C (2 min), 95°C (2 min) followed by 50 cycles of: 95°C (15 sec), 55°C (1 min). A melt curve was included to determine the specificity of the amplification and a no template control to detect contamination or primer dimers. Results were analysed with QuantStudioTM Real-Time PCR Software, triplicates were averaged, normalised to the housekeeping gene *rpsL* [46] and relative expression determined via the $2^{-\Delta\Delta CT}$ method [497].

Results

Antibiotic resistance and the location of acquired resistance in the genome

This study assayed nine additional antibiotics or antibiotic combinations to further characterise the phenotypic resistance of these isolates (Supplementary Table S1). Strains 1_GR_13, 2_GR_12 and 16_GR_13 were non-susceptible to all antibiotics including the 24 antibiotics tested previously [28]. 20_GR_12 was only susceptible to gentamicin and polymyxins.

MinION DNA sequencing for all isolates was run for ≥20 hours which generated 1.19 GB (215X) for 1_GR_13, 0.39 GB (67X) for 2_GR_12, 0.56 GB (101X) for 16_GR_13 and 0.64 GB (115X) for 20_GR_12 (Supplementary Table S2). Across the differing assembly tools, the chromosome sequence commonly circularised as a 5.0-5.4 Mb contig including plasmids ranging between 13-193 kb with the exception of 2_GR_12. Utilising the capacity for MinION sequencing to read long fragments of DNA, the location of antibiotic resistance genes were clearly resolved (Table 1). Aligning ONT reads to the final assembly revealed that DNA sequencing had 90% accuracy across isolates.

Utilising the capacity for MinION sequencing to read long fragments of DNA, the location of antibiotic resistance genes were clearly resolved (Table 1). All genomes were circular with the exception of 2_GR_12 where 3 plasmids remained linear. This was partly due to difficulties extracting DNA, and not retaining long fragments and subsequently, lower coverage of the genome (Supplementary Figure S1, Table S2). Amongst the four isolates, the chromosome size ranged between 5.1-5.5 Mb which encoded resistance genes *blaSHV-11*, *fosA* and *oqxAB*. The majority of resistance (≥75%) mapped to plasmids.

At least one megaplasmid, defined as a plasmid larger than 100 kbp, was detected in all isolates (Table 1). These commonly harboured the replicon IncA/C2 or InFIB and IncFIIK. The IncA/C2 plasmid was present in all samples except 20_GR_12. This plasmid contained up to 16 resistance genes which conferred resistance towards aminoglycosides, β-lactams, phenicols, rifampicin, sulphonamides, tetracyclines and trimethoprim, with the exception

of 16_GR_13. Isolate 16_GR_13 lacked trimethoprim resistance on its IncA/C2 plasmid. The plasmids containing both replicons IncFIB and IncFIIK differed vastly between all four replicates. All contained IncFIB$_{pKpn3}$ and IncFIIK, however, 1_GR_13 differed with IncFII$_{pKP91}$. Additionally, a differing IncFIB replicon was detected on a separate contig in 1_GR_13 (pKPHS1) and 2_GR_12 (pQil). The only instance where another dual replicon was identified was in 1_GR_13 which harboured both IncR and IncN. This plasmid contained aminoglycoside, β-lactam, trimethoprim, macrolide and sulphonamide resistance. 1_GR_13 also contained a 5.5 kb circular contig which was annotated as a phage genome. Various regions of these megaplasmids were unique to these isolates compared to prior sequences deposited on NCBI (Supplementary Table S5).

The ColRNAI plasmid was present in all except 1_GR_13 which encoded aminoglycoside and quinolone resistance (*aac(6')-Ib, aac(6')-Ib-cr)* (Table 1). The ColRNAI plasmid in 2_GR_12 and 20_GR_12 was 13841 bp in size and shared 75% similarity between the two isolates. This plasmid differed in 16_GR_13 which contained no resistance genes and 35% the size. The same IncX3 plasmid (43380 bp) was apparent in isolates 2_GR_12 and 20_GR_12. Unique to 16_GR_13 was the IncL/ M$_{pOXA\text{-}48}$ plasmid containing *blaOXA-48* and the 50979 bp IncN plasmid in 20_GR_12 with resistance against 5 classes (aminoglycoside (*aph(3'')-Ib*, *aph(6)-Id*), β-lactam (*blaTEM-1A*), sulphonamide (*sul2*), tetracycline (*tet(A)*), trimethoprim (*dfrA14*)) of antibiotics.

Multiple copies of acquired resistance genes were apparent across plasmids in several isolates. For 1_GR_13, up to three copies were present of genes *aadA24*, *aph(3')-Ia*, *aph(6)-Id*, *dfrA1*, *dfrA14*, *strA* and *sul1* (Table 1). In 2_GR_12, *sul1* and *blaTEM-1A* were duplicated and for 16_GR_13, only *sul1* was represented twice.

Real-time detection emulation of resistance genes via DNA sequencing

The vast majority (≥70%) of resistance genes were detected via DNA sequencing within the first 2 hours (Figure 1, Supplementary Table S5). These genes confer resistance towards aminoglycosides, β-lactams, fosfomycin, macrolides, phenicols, quinolones, rifampicin, sulphonamides, tetracyclines and trimethoprim. 20_GR_12 lacked acquired resistance genes for macrolides, phenicols and rifampicin, however, all other classes were detected within 2 hours. All isolates, except 2_GR_12, were sequenced for 21 hours which was sufficient to obtain the complete genome assembly. Only a few additional genes were detected after the first 10 hours across isolates (Supplementary Table S5). For 2_GR_12, an extended run of 41 hours detected no further genes after 20 hours. Overall, the presence of these resistance genes corresponded to a resistant phenotype towards aminoglycosides, β-lactams, fosfomycin, phenicols, quinolones, sulphonamides (sulfamethoxazole), tetracyclines and trimethoprim (Supplementary Table S1). As

macrolides and rifampicin are not routinely used to treat *K. pneumoniae* infections, no breakpoints exist according to CLSI and EUCAST guidelines, however, all isolates exhibit an MIC ≥128 µg/ml towards erythromycin (macrolide) and ≥64 µg/ml for rifampicin (Supplementary Table S1).

Post 2 hours of sequencing, several genes not observed in the final assembly via ResFinder 3.0 were detected (Supplementary Table S5). These were predominantly genes attributed to aminoglycoside, β-lactam, rifampicin and phenicol resistance. Furthermore, resistance genes to additional ~~differing~~ classes were detected including fusidic acid and vancomycin. This was evident in 2_GR_12 (*fusB*) and 16_GR_13 (*fusB*, *vanR*). However, these genes had less than 30 reads and their phred-scale mapping quality (MAPQ) scores were less than 10 (misplaced probability greater than 0.1). Furthermore, the majority of genes not observed in the final assembly nor observed in Illumina data exhibited a MAPQ score of ≤10 which may indicate that a more stringent threshold is required to negate false positives. However, if this threshold increases, true positives would not be detected including *aadA1*, *aadA2* and *ARR-2* in 2_GR_12 and *blaOXA-48*, *blaCTX-M-15* and *ARR-2* in 16_GR_13.

Several genes found in the final assembly were not detected in the real-time emulation analysis (Supplementary Table S5). This was mainly observed for aminoglycoside resistance encoding genes. For 1_GR_13, this included *aadA1*, *ant(2'')-Ia*, *aph(6)-Id* and *aadA24*. Similarly, 2_GR_12 and 20_GR_12 lacked *aph(3'')-Ib* and *aph(6)-Id*. 2_GR_12 additionally had the absence of *ant(2'')-Ia*. Detection of *ant(2'')-Ia*, *aph(3'')-Ib*, *aph(6)-Id* was not present in 16_GR_13. 16_GR_13 further lacked *catB4* (phenicol) and *tet(A)* (tetracycline). Various phenicol resistance genes were reported in the real-time emulation however, the incorrect gene was identified which may represent sequencing errors accumulated over time and high similarity to other phenicol resistance genes. The tetracycline resistance gene, *tet(A)*, was interestingly not reported in this emulation with 190 reads and the majority of reads exhibiting a high mapping confidence (MAPQ = 60, equivalent to an error probability of $1x10^{-6}$). This gene was only detected after 10 hours for 1_GR_13 and 2_GR_12 and this result may be influenced by the presence of only 1 copy of *tet(A)* encoded on a low copy number megaplasmid (between 1 to 1.5, see Table 1).

Direct RNA sequencing resistance detection

The time required to detect resistance was further interrogated using RNA sequencing. Rapid detection was possible for several resistance genes via direct RNA sequencing (Figure 1). This was evident for genes conferring resistance to aminoglycosides, β-lactams, sulphonamides and trimethoprim for all four isolates. Resistance towards these antibiotics was commonly detected within 6 hours. In some instances, quinolone, rifampicin, fosfomycin and phenicol

resistance was detected. A similar result was obtained whether all reads or passed reads alone were analysed. The most significant difference when analysing all reads was the detection of *fosA* in 1_GR_13 and *ARR-2* and *fosA* in 2_GR_12. Consistently absent from this analysis were genes attributed to macrolide (*mph(A)*) and tetracycline (*tet(A)*, *tet(G)*) resistance, however, isolates exhibited high levels of resistance to tetracycline (>64 µg/ml) (Supplementary Table S1).~~ This may indicate that isolates require antibiotic exposure to enable transcription of these genes.~~ Commonly no new genes were detected after 12 hours of sequencing with the exception of *fosA* in 2_GR_12. Although *fosA* was detected when including the failed reads, a low MAPQ score (≤10) was apparent. Similar to the DNA real-time detection, several genes not found in the final assembly were identified (Supplementary Table S5). With the exception of 20_GR_12, this included *aadB* and *strB* for all isolates. Additional genes detected included *ARR-7* in 1_GR_13, *strA* in 2_GR_12 and for 16_GR_13, *blaCTX-M-64*, *blaOXA-436* and *strA*. Similar genes or gene families were identified when comparing DNA and direct RNA sequencing. Overall, genes were detected more readily via DNA sequencing however, there were~~rather than RNA sequencing, possibly due to a lack of RNA expression in the absence of the antibiotic to which resistance is encoded. There were only~~ a few instances where RNA sequencing detected resistance quicker~~ more quickly than DNA sequencing~~: *aac(3')-IIa* in 16_GR_13 and *sul2* and *catA1* in 2_GR_12. Similar results were observed when investigating data yield rather than time which compensates for the slower translocation speed associated with direct RNA sequencing (Supplementary Figure S4).

Levels of expression of resistance genes

RNA sequencing accumulated over approximately 40 hours yielded between 0.9 and 1.7 million reads for these isolates (Supplementary Figure S3). However, only a low proportion (≤14.64%) of these reads passed base-calling using Albacore 2.2.7 (Supplementary Table S6). Aligning passed reads alone to the final assembly, ≥98% of reads were mappable, however, ≤40% of these had a MAPQ score ≥10. When all reads (pass and fail) were aligned, the majority were not mappable to the reference genome (≥76.69%) and commonly exhibited a low MAPQ score (≤10). Low mapping quality could be attributed to assignment of reads to multiple copies of genes in the genome. Furthermore, the ONT error rates could lead to misassignment of reads to genes. In light of this, we decided to benchmark a number of different base-callers, including Albacore 2.2.7, Guppy 3.03 as well as Chiron v0.5 which was trained in-house (Supplementary Table S6, Figure S5). Chiron base-called more reads compared to Albacore 2.2.7 and Guppy 3.0.3, however, fewer reads aligned to the reference genome and had a slightly lower identity rate. Albacore 2.2.7 had the highest average accuracy across isolates (84.87%) closely followed by Guppy 3.0.3 (84.62%) and then

Chiron v0.5 (78.19%) (Supplementary Table S6). These results reflect the fact that base-calling algorithms have not yet been optimised for direct RNA sequencing, and even less so for bacterial RNA sequencing. The poly(A) length was commonly found to be approximately 400 to 700 bp across isolates (Supplementary Figure S6). Taking into consideration the Albacore 2.2.7 base-called reads, a proportion of these reads were found to map to rRNA including 1_GR_13 (18%), 2_GR_12 (37%), 16_GR_13 (24%) and 20_GR_12 (23%). Overall, at least 58% of genes (with at least 1 read mapping to the gene) were identified to be expressed across isolates (1_GR_13 (68%), 2_GR_12 (58%), 16_GR_13 (75%) and 20_GR_12 (69%).

Amongst the four isolates, levels of expression for resistance genes on the chromosome (*blaSHV-11*, *fosA* and *oqxAB*) were low (≤122 counts per million mapped reads (cpm)) (Figure 2). The remaining resistance genes were located on plasmids. Resistance genes exhibiting high levels of expression (300 cpm) were apparent in 1_GR_13 (*blaTEM-1B*, *blaVIM-27*, *sul1*, *aph(3')-Ia*), 2_GR_12 (*aac(6')-Ib*, *catA1*, *blaKPC-2*), 16_GR_13 (*aac(6')Ib-cr*, *aac(3)-IIa*, *blaCTX-M-15*, *blaTEM-1B*, *blaOXA-48*) and 20_GR_12 (*blaKPC-2*, *aac(6')Ib*). Counts for *aac(6')-1b* and *aac(6')-1b-cr* in 2_GR_12 and 20_GR_12 were grouped. The gene *aac(6')-1b-cr* is a shortened version of *aac(6')-1b* and both were identified in the same genome position, hence, only *aac(6')-1b* is displayed in Figure 2. Relative expression Expression estimates did not differ significantly when analysing passed reads alone or all reads. We estimated the 90% confidence interval in cpm estimates using a beta-distribution (Supplementary Figure S7 figure …). All highly expressed genes were detected within 6 hours as per the real-time detection emulation. As anticipated, low levels of expression were observed for fosfomycin (*fosA*), tetracycline (*tet(A)*, *tet(B)*) and macrolide (*mph(A)*) resistance.

A subset of 11 resistance genes which represent resistance across various classes of antibiotics were investigated to validate differential gene expression in these RNA extractions via qRT-PCR (Figure 3). These included resistance towards aminoglycosides (*aac(6')Ib*, *strA*), β-lactams (*blaKPC-2*, *blaOXA-10*, *blaTEM-1*), phenicols (*cmlA1*), trimethoprim (*dfrA14*), fosfomycin (*fosA*), quinolone (*oqxA*), sulphonamides (*sul2*) and tetracyclines (*tet(A)*). A similar trend was observed between direct RNA sequencing and qRT-PCR results (Spearman's rank correlation coefficient: 0.83; Pearson correlation: 0.86) (Figure 3). The highest expression of a resistance gene was observed for *blaKPC-2* although only one copy was present in a lower copy number plasmid in 2_GR_12 and 20_GR_12 (Figure 2, Figure 3 and Table 1). Additionally, low levels of expression for *fosA* and *tet(A)* were apparent despite exhibiting resistance towards fosfomycin and tetracycline (Figure 3, Supplementary Table S1). Direct RNA sequencing was unable to detect low levels of expression whilst qRT-PCR could detect these genes (Figure 3).

Across the transcriptome, antibiotic resistance genes were identified to harbour high ~~differential~~ expression between isolates (Figure 4). Virulence genes were comparable across these strains similar to all remaining or background genes. The top differentially expressed genes were determined (Supplementary Figure S8~~7~~) and several were associated with polymyxin resistance pathways. Heightened expression was seen in polymyxin-resistant isolates 1_GR_13, 2_GR_12, 16_GR_13 in comparison to the single susceptible isolate (20_GR_12) in particular, genes associated with Ara4N synthesis. These genes include 4-deoxy-4-formamido-L-arabinose-phosphoundecaprenol deformylase (*arnD*), UDP-4-amino-4-deoxy-L-arabinose formyltransferase and UDP-4-amino-4-deoxy-L-arabinose-oxoglutarate aminotransferase.

Transcriptional biomarkers for polymyxin resistance

Three of the isolates harboured resistance towards polymyxins via disruptions in *mgrB* which included 1_GR_13, 2_GR_12 and 16_GR_13. 1_GR_13. Isolate 1_GR_13 has~~These isolates have~~ an insertion sequence (IS) element, IS*Kpn26*-like, at nucleotide position 75 in the same orientation as *mgrB* whilst~~.~~ 2_GR_12 has this IS element in the opposite orientation plus ~~also contained an insertion at the same position, however, in the opposite orientation and~~ additional mutations in *phoP* (A95S) and *phoQ* (N253T). 16_GR_13 harbours~~possessed~~ an IS element, IS*1R*-like, 19 bp upstream of *mgrB*. Direct RNA sequencing revealed only ~~low level~~low-level expression of *mgrB* ~~in isolates~~ (1_GR_13 (78.4 cpm), 2_GR_12 (16.3 cpm), 16_GR_13 (0 cpm), 20_GR_12 (2.3 cpm)). The expression levels of various genes associated with this pathway were verified via qRT-PCR ~~which include genes *phoP, phoQ, pmrA, pmrB, pmrC, pmrD, pmrE, pmrH* and *pmrK*~~ (Figure 5). Direct RNA sequencing revealed a slight increase in transcription of *phoPQ* (≥2-fold) relative to ~~the expression in~~ 20_GR_12. A ≥13-fold increase in expression was observed for *pmrH* and ≥8-fold elevation for *pmrK*. Similar trends for expression were also reported using qRT-PCR (Figure 5~~B~~).

Discussion

XDR *K. pneumoniae* infections pose as a major threat to modern medicine. A rapid diagnostic would help to guide appropriate treatment options [1, 6]. The MinION sequencing technology employed in this study has potential to detect antibiotic resistance in a timely manner. Three of the four *K. pneumoniae* isolates examined in this study harboured non-susceptibility to all antibiotics or antibiotic combinations assayed, and hence would be classified as PDR according to published guidelines [50~~48~~]. ONT sequencing was able to resolve both the assembly of plasmids

harbouring high levels of resistance (through DNA sequencing) and the expression from the resistome in the absence of antibiotic treatment (via RNA sequencing).

The ability for ONT to sequence long fragments of DNA has significantly aided the assembly of bacterial genomes and plasmids [16-18]. In this study, multiple megaplasmids (≥100 kbp) were identified which were previously unresolved via Illumina sequencing [28]. These harboured replicons IncA/C2 or a dual replicon, IncFIIK and IncFIB. The IncA/C, IncF and IncN plasmids have been commonly associated with multidrug resistance [51~~48~~]. Although several plasmids in this study revealed similarity to previously reported isolates via NCBI, various sequences deviated. In particular, the IncA/C2 plasmid exhibited multiple regions unique to these isolates. Several IncA/C2 megaplasmids have been previously described which harbour various resistance genes. However, the extent of resistance observed in our study is extreme when compared to prior reports [52~~0~~, 53~~1~~]. Prior studies have shown the IncFIIK and IncFIB replicons to localise on the same plasmid and also megaplasmids with multidrug resistance [6]. The $IncFIB_{pQil}$ plasmid in this study contained various β-lactam resistance genes (*blaKPC-2*, *blaOXA-9*, *blaTEM-1A*) which has been identified previously [54~~2~~]. Similarly, *blaOXA-48* segregated with the IncL/M replicon [55~~3~~,56~~4~~], however, deviations in this plasmid were identified.

The real-time analysis capability entailed in MinION sequencing has the potential to rapidly determine antibiotic resistance profiles of pathogenic bacteria. Previously this device has been utilised to assemble bacterial genomes, discern species and detect antibiotic resistance [12-15]. This study investigated the potential time required to discern resistance via a real-time emulation as previously described [14]. The majority (≥70%) of resistance genes were detected via DNA sequencing within 2 hours. Several genes not identified in the final assembly were detected after 2 hours of sequencing. This may be attributed to the high similarity (≥80%) amongst various genes, in particular, those associated with aminoglycoside, β-lactam, rifampicin and phenicol resistance. Furthermore, the error rate associated with ONT sequencing, and the accumulation of these errors over time, may result in the false annotation of these genes. Nanopore DNA sequencing currently has an accuracy ranging from 85~~0~~ to 95~~0~~% (90% in our study), which limits its ability to detect genomic variations [17, 57]. Several resistance genes only differ by a few nucleotides which significantly impacts the resistance phenotype and the antibiotics which can be utilised to treat these infections. However, software tools such as Nanopolish (https://github.com/jts/nanopolish) and Tombo (https://github.com/nanoporetech/tombo) (similarly used to re-train Chiron v0.5 for direct RNA sequencing data) have the potential to correct these reads and would be helpful to integrate to increase the accuracy of detecting resistance

genes. We utilised native DNA sequencing in this study which retains epigenetic modifications such methylation which can hinder the accuracy of reads and subsequent calling of antibiotic resistance [58]. Furthermore, a small number of resistance genes were identified that were not present in the final assembly, however these all had MAPQ values less than 10 and less than 30 mapped reads. Some of these may be due to low-level kit contamination, while some of the false positives have sequence similarity to true positives and may be due to inaccuracies in base-calling. We further investigated the transcriptome of these isolates to potentially elucidate the correlation between genotype and the subsequent resistant phenotype. Detection of antibiotic resistance via sequencing commonly uses DNA due to the instability of RNA and the lengthy sample processing such as rRNA depletion [12-15, 58]. However, RNA provides additional information regarding the functionality of genes such as identifying conditions in which a resistance gene is present but not active which gives rise to a false positive via DNA alone. Conversely, if expression is only induced in the presence of an antibiotic, the absence of RNA transcripts results in a false negative. This study grew *K. pneumoniae* strains in the absence of antibiotic and ~~Several resistance genes only differ by a few nucleotides which significantly impacts the resistance phenotype and the antibiotics which can be utilised to treat these infections. Furthermore, direct RNA sequencing has an average error rate of 12% [21]. Hence, it is essential for the technology to increase its accuracy in order to correctly and rapidly diagnose antibiotic resistance.~~

~~Investigating the transcriptome of these isolates can potentially elucidate the correlation between genotype and the subsequent resistant phenotype. One of the advantages of RNA sequencing is that it can identify conditions in which a resistance gene is present but not expressed, potentially resulting in a susceptible phenotype. However, if expression is only induced in the presence of an antibiotic, the absence of RNA transcripts may falsely suggest susceptibility.~~ d~~D~~irect RNA sequencing revealed high levels of transcription from genes associated with aminoglycoside, β-lactam, sulphonamide and trimethoprim resistance within 6 hours of our study. In particular, the highest levels of expression were observed for the β-lactamase gene *blaKPC-2* in 2_GR_12 and 20_GR_12. Alterations in the promoter region have previously been reported to influence high levels of expression [59]. Notably, the promoter or operon (co-transcribed genes) can largely influence expression of genes. The detection of quinolone, rifampicin, and phenicol resistance correlated to the levels of transcription within samples. All isolates exhibited low levels of expression for fosfomycin, macrolide and tetracycline resistance, despite exhibiting phenotypic resistance to fosfomycin and tetracycline [28]. FosA, an enzyme involved in fosfomycin degradation, is commonly encoded chromosomally in *K. pneumoniae* and a combination of expression and enzymatic activity contributes to resistance [60]. Noteably, Klontz

Formatted: Not Highlight

Formatted: Font: Italic

et al identified that chromosomally integrated FosA, similarly observed in our study, from *K. pneumoniae* harboured a higher catalytic efficiency. A higher catalytic efficiency may reason why our strains only require a low abundance of expression and still retain fosfomycin resistance. Genes *tet(A)* and *tet(G)* encode efflux pumps which, in the absence of tetracycline, are lowly expressed and the lack of antibiotic supplementation in this study confirms this observation [61]. Detecting inducible resistance (antibiotic exposure required for gene expression) such as tetracycline resistance highlights one of the advantages of investigating the transcriptome.

There are several other variables to consider when interpreting expression levels in bacterial RNA sequencing data. These include the extent prior exposure to antibiotics in the clinic alters transcription and the copy number of resistance genes and the plasmids these are encoded on. Limitations were observed when base-calling bacterial direct RNA sequencing and may be attributed to trimming the long artificial poly(A) tail and interference of RNA modifi cations. This entailed an increased error rate of ≤23% across base-callers (12% identified in a prior study [21]) and a poor alignment rate ≤23%. ~~Whether this transcription is due to prior exposure to these antibiotics in the clinic and the longevity of this expression post exposure warrants further investigation. The changes in transcription levels in response to antibiotic exposure also need to be assessed in future experiments.~~ Furthermore, the time required to detect resistance may be hindered by the slower translocation speed associated with direct RNA sequencing (70 bases/ second) compared to DNA sequencing (450 bases/ second) [57]. Our findings show that the slower time-to-detection of resistance genes in direct RNA sequencing was due to both the level of expression as well as the slower translocation speed, and hence using cDNA would only partially overcome this limitation

~~Furthermore, direct RNA sequencing has an average error rate of 12% [21]. Hence, it is essential for the technology to increase its accuracy in order to correctly and rapidly diagnose antibiotic resistance.~~

~~Furthermore, insufficient rRNA depletion and low base-calling of data could be impacting the detection of this low level expression.~~

~~Another variable to consider when evaluating differential expression is the operon or promoter which can further be explored via cloning. In particular, the highest levels of expression were observed for *blaKPC-2* in 2_GR_12 and 20_GR_12. Alterations in the promoter region have previously been reported to influence high levels of expression [55]. Furthermore, despite low levels of transcription for fosfomycin (*fosA*) and tetracycline (*tet(A)*, *tet(G)*), phenotypically these isolates consistently retain resistance [28]. FosA, an enzyme involved in the degradation of fosfomycin, is commonly encoded chromosomally in *K. pneumoniae* and a combination of expression and enzymatic~~

activity contributes to resistance [56]. Genes *tet(A)* and *tet(G)* encode efflux pumps which, in the absence of tetracycline, are lowly expressed [57]. Detecting inducible resistance such as tetracycline resistance highlights one of the advantages of investigating the transcriptome. Additionally, copy number of plasmids can further alter the levels of expression detected for these resistance genes.

WIn this study we also investigated pathways attributed to polymyxin resistance. Three of these strains exhibited an IS element upstream of within *mgrB*, the negative regulator of PhoPQ [29]. Elevated expression was apparent for *phoPQ* and also the *pmrHFIJKLM* operon in our polymyxin-resistant isolates harbouring a disruption in *mgrB*. This has previously been witnessed for other *K. pneumoniae* isolates harbouring *mgrB* disruptions and is a potential transcriptional marker for polymyxin resistance [29, 464, 6258, 6359]. However, this study is limited to four isolates and one mechanism associated with polymyxin resistance. Other pathways have previously been identified including the role of other two component regulatory systemTCSs such as CrrAB [6440]. The ability to use relative expression of key genes to detect polymyxin resistance requires further validation, including an increased sample size of resistant and non-resistant isolates. Furthermore, additional functional experiments such as complementation assays would be required in order to validate the contribution of a certain mutation to the transcriptome and subsequent resistance.

Conclusions

This study has utilised MinION sequencing to assemble four XDR *K. pneumoniae* genomes and has revealed several unique plasmids harbouring multidrug resistance. The vast majority of this resistance was detectable within 2 hours of sequencing., Athough a small number of resistance genes were identified that were not present in the final assembly, however these all had MAPQ values less than 10 and a small number of mapped reads. Some of these may be due to low-level kit contamination, while some of the false positives have sequence similarity to true positives and may be due to inaccuracies in base-calling. Exploiting this analysis in real-time would allow for a rapid diagnostic, however, the presence of a resistance gene does not necessarily indicate resistance is conferred and requires additional phenotypic characterisation. This research also established a methodology and analysis for bacterial direct RNA sequencing. The differential expression of resistance genes were successfully detected via this technology and can be exploited for bacterial transcriptomics. Once base-calling algorithms have been optimised, this could allow for a whole transcriptome interrogation of full lengthfull-length transcripts regulated by operons, where more than one gene is co-expressed in a transcript, and the evaluation of the poorly characterised RNA modificationsepitranscriptome. This

~~research established a methodology and analysis for bacterial direct RNA sequencing. The differential expression of resistance genes were successfully detected via this technology and can be exploited for bacterial transcriptomics.~~ Overall, this study has begun to unravel the association between genotype, transcription and subsequent resistant phenotype in these XDR/ PDR *K. pneumoniae* clinical isolates, establishing the groundwork for developing a diagnostic that can rapidly determine bacterial resistance profiles.

Availability of supporting data

The datasets supporting the results presented here are available in the National Center for Biotechnology Information repository BioProject PRJNA307517 (www.ncbi.nlm.nih.gov/bioproject/PRJNA307517). ONT DNA sequencing data has been deposited on the Sequence Read Archive (www.ncbi.nlm.nih.gov/sra/) under study SRP133040. Accession numbers are as follows: 1_GR_13 (SRR6747887), 2_GR_12 (SRR6747886), 16_GR_13 (SRR6747885) and 20_GR_12 (SRR6747884). ONT direct RNA sequencing data (pass and fail reads) have been deposited on the Sequence Read Archive (www.ncbi.nlm.nih.gov/sra/) under study SRP133040. Accession numbers are as follows: 1_GR_13 (SRR7719054), 2_GR_12 (SRR7719055), 16_GR_13 (SRR7719052) and 20_GR_12 (SRR7719053).

Abbreviations

Ara4N: 4-amino-4-deoxy-L-arabinose; caMHB: cation-adjusted Muller Hinton Broth; CLSI: Clinical & Laboratory Standards Institute; CI: Confidence interval; cpm: counts per million; EUCAST: The European Committee on Antimicrobial Susceptibility Testing; FDR: False discovery rate; HMW: High molecular weight; IS: Insertion sequence; LB: Lysogeny broth; MAPQ: Mapping quality; MIC: Minimum inhibitory concentration; NCBI: National Center for Biotechnology Information; ONT: Oxford Nanopore Technologies; PDR: Pandrug-resistant; RAST: Rapid Annotation using Subsystem Technology; rRNA: Ribosomal RNA; XDR: Extensively drug-resistant.

Competing Interests

The authors declare that there are no competing interests.

Funding

LJMC is an NHMRC career development Fellow APP1103384. MAC is an NHMRC Principal Research Fellow (APP1059354) and currently holds a fractional Professorial Research Fellow appointment at the University of Queensland with his remaining time as CEO of Inflazome Ltd. a company headquartered in Dublin, Ireland that is developing drugs to address clinical unmet needs in inflammatory disease by targeting the inflammasome. MEP is an

Australian Postgraduate Award scholar. MATB is supported in part by a Wellcome Trust Strategic Award 104797/Z/14/Z. This work was supported by the Institute for Molecular Bioscience Centre for Superbug Solutions (610246).

Author Contributions

MEP, LJMC, MATB and MAC conceived this study. MEP, SHN and HT performed the sequencing analysis. Laboratory work was carried out by MEP and TPSD. MEP wrote the paper with input from all authors.

Acknowledgements

We would like to acknowledge Dr Ilias Karaiskos and Dr Helen Giamarellou for providing the bacterial strains in this study. We also acknowledge Dr Evangelos Bellos for his guidance on the RNA sequencing analysis and Dr Devika Ganesamoorthy for the initial advice on the direct RNA sequencing library preparation. We would like to acknowledge Dr Intawat Nookaew for providing yeast direct RNA sequence data.

References

1. Martin RM, Bachman MA. Colonization, Infection, and the Accessory Genome of *Klebsiella pneumoniae*. *Front Cell Infect Microbiol.* 2018;8:4.

2. Magill SS, Edwards JR, Bamberg W, et al. Multistate point-prevalence survey of health care-associated infections *N Engl J Med*. 2014;370:1198-208.

3 Kalanuria AA, Ziai W, Mirski, M. Ventilator-associated pneumonia in the ICU. *Crit Care*. 2014;18:208.

4 Talha KA, Hasan Z, Selina F, et al. Organisms associated with ventilator associated pneumonia in intensive care unit. *Mymensingh Med J*. 2009;18:S93-7.

5 Podschun R, Ullmann U. *Klebsiella spp* as nosocomial pathogens: epidemiology, taxonomy, typing methods, and pathogenicity factors. *Clin Microbiol Rev*. 1998;11:589-603.

6 Navon-Venezia S, Kondratyeva K, Carattoli A.– *Klebsiella pneumoniae*: a major worldwide source and shuttle for antibiotic resistance. *FEMS Microbiol Rev*. 2017;41:252-75.

7 Karaiskos I, Giamarellou H. Multidrug-resistant and extensively drug-resistant Gram-negative pathogens: current and emerging therapeutic approaches. *Expert Opin Pharmacother*. 2014;15:1351-70.

8 Chen L, Todd R, Kiehlbauch J, et al. Notes from the Field: Pan-Resistant New Delhi Metallo-Beta-Lactamase-Producing *Klebsiella pneumoniae* - Washoe County, Nevada, 2016 *MMWR Morb Mortal Wkly Rep*. 2017;66:33.

9 Zowawi HM, Forde BM, Alfaresi M, et al. Stepwise evolution of pandrug-resistance in *Klebsiella pneumoniae*. *Sci Rep*. 2015;5:15082.

10 Sommer MOA, Munck C, Toft-Kehler RV, et al. Prediction of antibiotic resistance: time for a new preclinical paradigm? *Nat Rev Microbiol*. 2017;15:689-96.

11 Gardy JL, Loman NJ. Towards a genomics-informed, real-time, global pathogen surveillance system. *Nat Rev Genet*. 2018;19:9-20.

12 Lemon JK, Khil PP, Frank KM, et al. Rapid Nanopore Sequencing of Plasmids and Resistance Gene Detection in Clinical Isolates. *J Clin Microbiol*. 2017;55:3530-43.

13 Votintseva AA, Bradley P, Pankhurst L, et al. Same-Day Diagnostic and Surveillance Data for Tuberculosis via Whole-Genome Sequencing of Direct Respiratory Samples. *J Clin Microbiol*. 2017;55:1285-98.

14 Cao MD, Ganesamoorthy D, Elliott AG, et al. Streaming algorithms for identification of pathogens and antibiotic resistance potential from real-time MinION^{TM} sequencing. *Gigascience*. 2016;5:32.

15 Quick J, Ashton P, Calus S, et al. Rapid draft sequencing and real-time nanopore sequencing in a hospital outbreak of *Salmonella. Genome Biol*. 2015;16:114.

16 Wick RR, Judd LM, Gorrie CL, et al. Completing bacterial genome assemblies with multiplex MinION sequencing. *Microb Genom*. 2017;3:e000132.

17 Li R, Xie M, Dong N, et al. Efficient generation of complete sequences of MDR-encoding plasmids by rapid assembly of MinION barcoding sequencing data. *Gigascience*. 2018;7:1-9.

18 George S, Pankhurst L, Hubbard A, et al. Resolving plasmid structures in Enterobacteriaceae using the MinION nanopore sequencer: assessment of MinION and MinION/Illumina hybrid data assembly approaches. *Microb Genom*. 2017;3:e000118.

19 Garalde, DR, Snell, EA, Jachimowicz, D, et al. Highly parallel direct RNA sequencing on an array of nanopores. *Nat Methods*. 2018;15:201-6.

20 Ozsolak F, Milos PM. RNA sequencing: advances, challenges and opportunities. *Nat Rev Genet*. 2011;12: 87-98.

21 Jenjaroenpun P, Wongsurawat T, Pereira R, et al. Complete genomic and transcriptional landscape analysis using third-generation sequencing: a case study of *Saccharomyces cerevisiae* CENPK113-7D. *Nucleic Acids Res*. 2018;46:e38.

22 Workman RE, Tang A, Tang PS, et al. Nanopore native RNA sequencing of a human poly(A) transcriptome. *bioRxiv*. 2018;459529.

23 Moldovan N, Tombacz D, Szucs A, et al. Third-generation Sequencing Reveals Extensive Polycistronism and Transcriptional Overlapping in a *Baculovirus*. *Sci Rep*. 2018;8:8604.

24 Keller MW, Rambo-Martin BL, Wilson MM, et al. Direct RNA Sequencing of the Coding Complete Influenza A Virus Genome. *Sci Rep*. 2018;8:14408.

25 Depledge DP, Srinivas KP~~Puthankalam SK~~, Sadaoka T, et al. Direct~~Native~~ RNA sequencing on nanopore arrays redefines the transcriptional complexity of a viral pathogen. *Nat Commun.*~~*bioRxiv.*~~ 2019~~8~~;10:754~~373522~~.

26 Smith AM, Jain M, Mulroney L, et al. Reading canonical and modified nucleotides in 16S ribosomal RNA using nanopore direct RNA sequencing. *Plos One*~~*bioRxiv*~~. 2019~~7~~;14:e0216709~~132274~~.

27 Sorek R, Cossart P. Prokaryotic transcriptomics: a new view on regulation, physiology and pathogenicity. *Nat Rev Genet*. 2010;11:9-16.

28 Pitt ME, Elliott AG, Cao, MD, et al. Multifactorial chromosomal variants regulate polymyxin resistance in extensively drug-resistant *Klebsiella pneumoniae*. *Microb Genom*. 2018;4:~~:~~mgen1090000158.

29 Olaitan AO, Morand S, Rolain JM. Mechanisms of polymyxin resistance: acquired and intrinsic resistance in bacteria. *Front Microbiol*. 2014;5:643.

30 Teng H, Cao MD, Hall MB, et al. Chiron: translating nanopore raw signal directly into nucleotide sequence using deep learning. *GigaScience.* 2018;7:10.1093/gigascience/giy037.

31 Li H. Aligning sequence reads, clone sequences and assembly contigs with BWA-MEM. *arXiv*. 2013;13033997.

32 Zankari E, Hasman H, Cosentino S, et al. Identification of acquired antimicrobial resistance genes. *J Antimicrob Chemother*. 2012;67:2640-4.

33 Lassmann T, Frings O, Sonnhammer EL. Kalign2: high-performance multiple alignment of protein and nucleotide sequences allowing external features. *Nucleic Acids Res*. 2009;37:858-65.

34 Allison L, Wallace CS, Yee CN. When is a string like a string? In: Artificial Intelligence and Mathematics. *Ft Lauderdale FL*. 1990.

35 Bankevich A, Nurk S, Antipov D, et al. SPAdes: a new genome assembly algorithm and its applications to single-cell sequencing. *J Comput Biol*. 2012;19:455-77.

36 Cao MD, Nguyen SH, Ganesamoorthy D, et al. Scaffolding and completing genome assemblies in real-time with nanopore sequencing. *Nat Commun*. 2017;8:14515.

37 Wick RR, Judd LM, Gorrie CL, et al. Unicycler: Resolving bacterial genome assemblies from short and long sequencing reads. *PLoS Comput Biol*. 2017;13:e1005595.

38 Koren S, Walenz BP, Berlin K, et al. Canu: scalable and accurate long-read assembly via adaptive k-mer weighting and repeat separation. *Genome Res*. 2017;27:722-36.

39 Li H. Minimap and miniasm: fast mapping and *de novo* assembly for noisy long sequences. *Bioinformatics*. 2016;32:2103-10.

40 Vaser R, Sovic I, Nagarajan N, et al. Fast and accurate *de novo* genome assembly from long uncorrected reads. *Genome Res*. 2017;27:737-46.

41 Darling AE, Tritt A, Eisen JA, et al. Mauve assembly metrics. *Bioinformatics*. 2011;27:2756-7.

42 Aziz RK, Bartels D, Best AA, et al. The RAST Server: rapid annotations using subsystems technology. *BMC Genomics*. 2008;9:75.

43 Carattoli A, Zankari E, Garcia-Fernandez A, et al. *In silico* detection and typing of plasmids using PlasmidFinder and plasmid multilocus sequence typing. *Antimicrob Agents Chemother*. 2014;58:3895-903.

44. Li H, Handsaker B, Wysoker A, et al. The sequence alignment/map format and SAMtools. *Bioinformatics*. 2009;25:2078-9.

4~~4~~5 Quinlan AR. BEDTools: The Swiss-Army Tool for Genome Feature Analysis. *Curr Protoc Bioinformatics*. 2014;47:11.12.1-34.

46~~5~~ Cannatelli A, D'Andrea MM, Giani T, et al. *In vivo* emergence of colistin resistance in *Klebsiella pneumoniae* producing KPC-type carbapenemases mediated by insertional inactivation of the PhoQ/PhoP *mgrB* regulator. *Antimicrob Agents Chemother*. 2013;57:5521-6.

47~~6~~ Robinson JT, Thorvaldsdottir H, Winckler W, et al. Integrative genomics viewer. *Nat Biotechnol*. 2011;29; 24-6.

Formatted: Not Highlight

Formatted: Font: Italic, Not Highlight

Formatted: Not Highlight

48. Robinson MD, McCarthy DJ, Smyth GK. edgeR: a Bioconductor package for differential expression analysis of digital gene expression data. *Bioinformatics*. 2010;26:139-40.

49~~7~~ Livak KJ, Schmittgen TD. Analysis of relative gene expression data using real-time quantitative PCR and the 2(-Delta Delta C(T)) Method. *Methods*. 2001;25:402-8.

50~~48~~ Magiorakos AP, Srinivasan A, Carey RB, et al. Multidrug-resistant, extensively drug-resistant and pandrug-resistant bacteria: an international expert proposal for interim standard definitions for acquired resistance. *Clin Microbiol Infect.* 2012;18,268–81.

51~~49~~ Carattoli A. Resistance plasmid families in Enterobacteriaceae. *Antimicrob Agents Chemother*. 2009;53: 2227-38.

52~~0~~ Desmet S, Nepal S, van Dijl JM, et al. Antibiotic Resistance Plasmids Cointegrated into a Megaplasmid Harboring the *blaOXA-427* Carbapenemase Gene. *Antimicrob Agents Chemother*. 2018;62:e01448-17.

53~~1~~ Papagiannitsis CC, Dolejska M, Izdebski R, et al. Characterisation of IncA/C2 plasmids carrying an In416-like integron with the *blaVIM-19* gene from *Klebsiella pneumoniae* ST383 of Greek origin. *Int J Antimicrob Agents*. 2016;47:158-62.

54~~2~~ Chen L, Chavda KD, Melano RG, et al. Comparative genomic analysis of KPC-encoding pKpQIL-like plasmids and their distribution in New Jersey and New York Hospitals. *Antimicrob Agents Chemother*. 2014;58:2871-7.

55~~3~~ Poirel L, Bonnin RA, Nordmann P. Genetic features of the widespread plasmid coding for the carbapenemase OXA-48. *Antimicrob Agents Chemother*. 2012;56:559-62.

56~~4~~ Potron A, Poirel L, Nordmann P. Derepressed transfer properties leading to the efficient spread of the plasmid encoding carbapenemase OXA-48. *Antimicrob Agents Chemother*. 2014;58:467-71.

57 Rang FJ, Kloosterman WP, de Ridder J. From squiggle to basepair: computational approaches for improving nanopore sequencing read accuracy. *Genome Biol*. 2018;19:90.

58 Tamma PD, Fan Y, Bergman Y, et al. Applying rapid whole-genome sequencing to predict phenotypic antimicrobial susceptibility testing results among carbapenem-reistant *Klebsiella pneumoniae* clinical isolates. *Antimicrob Agents Chemother*. 2018;63: pii: e01923-18.

59~~5~~ Cheruvanky A, Stoesser N, Sheppard AE, et al. Enhanced *Klebsiella pneumoniae* Carbapenemase Expression from a Novel Tn4401 Deletion. *Antimicrob Agents Chemother*. 2017;61:e00025-17.

Formatted: Font: Italic

Formatted: Font: Italic

Formatted: Font: Italic

Formatted: Font: Italic

6056 Klontz EH, Tomich AD, Gunther S, et al. Structure and Dynamics of FosA-Mediated Fosfomycin Resistance in *Klebsiella pneumoniae* and *Escherichia coli*. *Antimicrob Agents Chemother*. 2017;61:e01572-17.

6157 Saenger W, Orth P, Kisker C, et al. The Tetracycline Repressor-A Paradigm for a Biological Switch. *Angew Chem Int Ed Engl*. 2000;39;2042-52.

6258 Cheng YH, Lin TL, Pan YJ, et al. Colistin resistance mechanisms in *Klebsiella pneumoniae* strains from Taiwan. *Antimicrob Agents Chemother*. 2015;59:2909-13.

6359 Haeili M, Javani A, Moradi J, et al. MgrB Alterations Mediate Colistin Resistance in *Klebsiella pneumoniae* Isolates from Iran. *Front Microbiol*. 2017;8:2470.

640 Baron S, Hadjadj L, Rolain JM, et al. Molecular mechanisms of polymyxin resistance: knowns and unknowns. *Int J Antimicrob Agents*. 2017;48:583-91.

Formatted: Not Highlight

Formatted: Normal, Left, Indent: Left: 0", First line: 0", Line spacing: single

Table and Figure Legends

Isolate	ST	Contig	Length (bp)	Coverage	Contig ID*	Resistance Genes**
1_GR_13	147	1	**5181675**	1	C	*blaSHV-11, fosA, oqxA, oqxB*
		2	**192771**	1.95	P: IncA/C2	*aadA1, ant(2'')-Ia, aph(6)-Id, ARR-2, blaOXA-10, blaTEM-1B, blaVEB-1, cmlA1, dfrA14, dfrA23, rmtB, strA, sul1, sul2, tet(A), tet(G)*
		3	**168873**	2	P: IncFIB$_{pKpn3}$, IncFII$_{pKP91}$	*aadA24, aph(3')-Ia, aph(6)-Id, dfrA1, dfrA14, strA*
		4	**108879**	1.53	P: IncFIB$_{pKPHS1}$	-
		5	**55018**	14.10	-	-
		6	**53495**	2.36	P: IncR, IncN	*aadA24, aph(3')-Ia, aph(6)-Id, blaVIM-27, dfrA1, mph(A), strA, sul1*
2_GR_12	258	1	**5466424**	1	C	*blaSHV-11, fosA, oqxA, oqxB*
		2	197872	1.3	P: IncFIB$_{pKpn3}$, IncFIIK	*aadA2, aph(3')-Ia, catA1, dfrA12, mph(A), sul1*
		3	175636	1.49	P: IncA/C2	*aadA1, ant(2'')-Ia, aph(3'')-Ib, aph(6)-Id, ARR-2, blaOXA-10, blaTEM-1A, blaVEB-1, cmlA1, dfrA14, dfrA23, rmtB, sul1, sul2, tet(A), tet(G)*
		4	95481	1.61	P: IncFIB$_{pQil}$	*blaKPC-2, blaOXA-9, blaTEM-1A*
		5	**43380**	1.91	P: IncX3	*blaSHV-12*
		6	**13841**	4	P: ColRNAI	*aac(6')-Ib, aac(6')Ib-cr*
16_GR_13	11	1	**5426917**	1	C	*blaSHV-11, fosA, oqxA, oqxB*
		2	**187670**	0.88	P: IncFIB$_{pKpn3}$; IncFIIK	*aac(3)-IIa, aac(6')Ib-cr, aadA2, aph(3')-Ia, blaCTX-M-15, blaOXA-1, catB4, dfrA12, mph(A), sul1*
		3	**155161**	0.99	P: IncA/ C2	*aadA1, ant(2'')-Ia, aph(3'')-Ib, aph(6)-Id, ARR-2, blaOXA-10, blaTEM-1B, blaVEB-1, cmlA1, rmtB, sul1, sul2, tet(A), tet(G)*
		4	**63589**	1.49	P: IncL/ M$_{pOXA-48}$	*blaOXA-48*
		5	**5234**	188.49	-	-
		6	**4940**	97.77	P: ColRNAI	-
20_GR_12	258	1	**5395894**	1	C	*blaSHV-11, fosA, oqxA, oqxB*
		2	**170467**	1.77	P: IncFIB$_{pKpn3}$; IncFIIK	*aph(3')-Ia, blaKPC-2, blaOXA-9, blaTEM-1A*
		3	**50979**	1.42	P: IncN	*aph(3'')-Ib, aph(6)-Id, blaTEM-1A, dfrA14, sul2, tet(A)*
		4	**43380**	1.78	P: IncX3	*blaSHV-12*
		5	**13841**	10.82	P: ColRNAI	*aac(6')-Ib, aac(6')Ib-cr*

Table 1: Final assembly of XDR *K pneumoniae* isolates and location of antibiotic resistance genes

*Contig ID represents chromosome (C) or plasmid (P): replicon determined via PlasmidFinder 1.3.

Formatted: Normal, Left, Line spacing: single

Formatted Table

Resistance genes identified using ResFinder 3.0 (≥90% sequence similarity, ≥60% minimum length) and displayed in alphabetical order. **Bold indicates a circular contig.

Figure 1: Time required to detect antibiotic resistance genes via the real-time emulation analysis using MinION DNA and direct RNA sequencing. (**A**) 1_GR_13, (**B**) 2_GR_12, (**C**) 16_GR_13 and (**D**) 20_GR_12. Legend colours identify the class of antibiotic to which the gene confers resistance, / on y-axis indicates reads detected more than one resistance gene and # is a family of genes detected (>3). An asterisk (*) indicates the inability for direct RNA sequencing to detect this gene. Albacore 2.2.7. base-called sequences were used and all reads (pass and fail) were included in this analysis.

Figure 2: Direct RNA sequencing ~~rRelative~~ expression of resistance genes aligned to completed genomes expressed as counts per million mapped reads ~~)~~ (post removal of reads mapping to rRNA) ~~normalised to housekeeping gene, *rpsL*, via direct RNA sequencing~~. ~~Strains investigated include:~~ (**A**) 1_GR_13, (**B**) 2_GR_12, (**C**) 16_GR_13 and (**D**) 20_GR_12. X-axis depicts the resistance genes and are grouped based on the location in the genome where P indicates a plasmid followed by replicon identity. Albacore 2.2.7 base-called pass and fail reads were used for analysis. ~~Quantitated as counts per million mapped reads (cpm) (post removal of reads mapping to rRNA) and D~~dotted line is set to 300 cpm.

Figure 3: Correlation between resistance genes detected via direct RNA sequencing and validated using qRT-PCR. Relative expression was calculated via normalizing to the housekeeping gene, *rpsL* for both direct RNA sequencing (log2(gene/*rpsL*)) and qRT-PCT ($2^{-\Delta\Delta CT}$). Due to high similarity between certain genes, several primers recognise more than one gene. These include *aac(6')Ib: aac(6')Ib-cr*, *aadA24*; *strA: aph(3'')-Ib* and *blaTEM-1: blaTEM-1A*, *blaTEM-1B*.

Figure 4: Correlation between the four XDR *K pneumoniae* isolates for gene expression via direct RNA sequencing. Top panels display spearman correlation coefficients. The diagonal panel shows the density of gene expression levels in counts per million mapped reads ~~(cpm)~~ for each sample (post removal of rRNA mapped reads). Bottom panels depict the correlation of gene expression between isolates as a scatter plot. Colours indicate categorization of gene: antimicrobial resistance genes (AMR) as per ResFinder 3.0, virulence genes (VIR) determined via RAST and all other genes or background genes (BG) are displayed. Cpm was capped at 2000.

Figure 5: Expression of genes associated with the polymyxin resistance pathway. Comparison between ~~(A)~~ direct RNA sequencing (solid shapes without asteris~~x~~k) and ~~(B)~~ qRT-PCR (solid shapes with asteris~~x~~k). –Direct RNA sequencing data is ~~depicted~~ calculated as log2(gene/ *rpsL*) and qRT-PCR as gene/ *rpsL*. All isolates except 20_GR_12 harboured resistance to polymyxin~~Isolates harbouring resistance to polymyxins~~ (MIC: >2 µg/mL) ~~include 1_GR_13, 2_GR_12 and 16_GR_13~~. The bars indicate the average of qRT-PCR and direct RNA sequencing. ~~An asterisks (*) indicates the qRT-PCR data point and bars represent mean.~~

Formatted: Font: Italic

Formatted: Font: Italic

Figure 1 Click here to access/download;Figure;Figure_1.docx

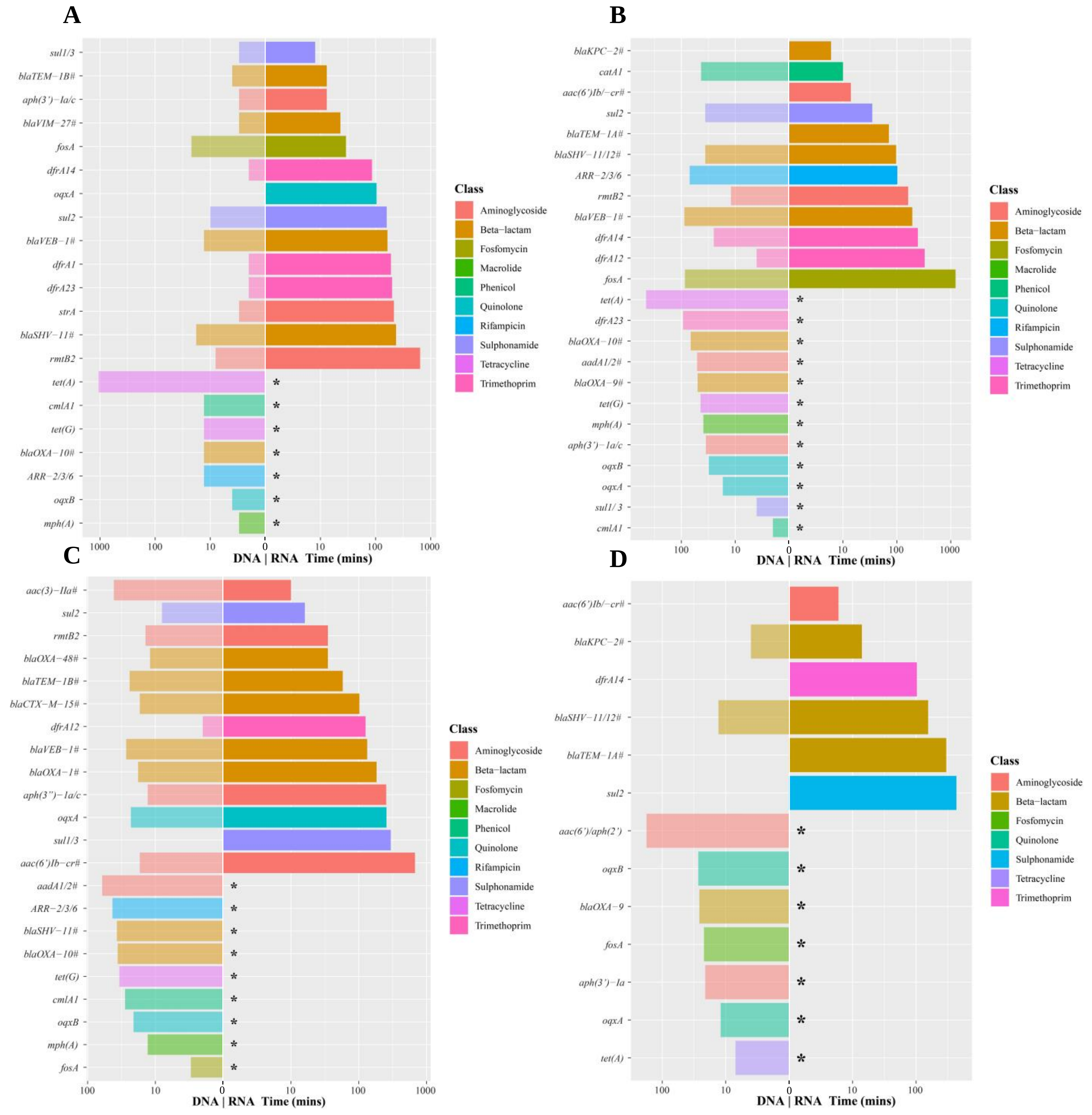

Figure 2

Click here to access/download;Figure;Figure_2.docx

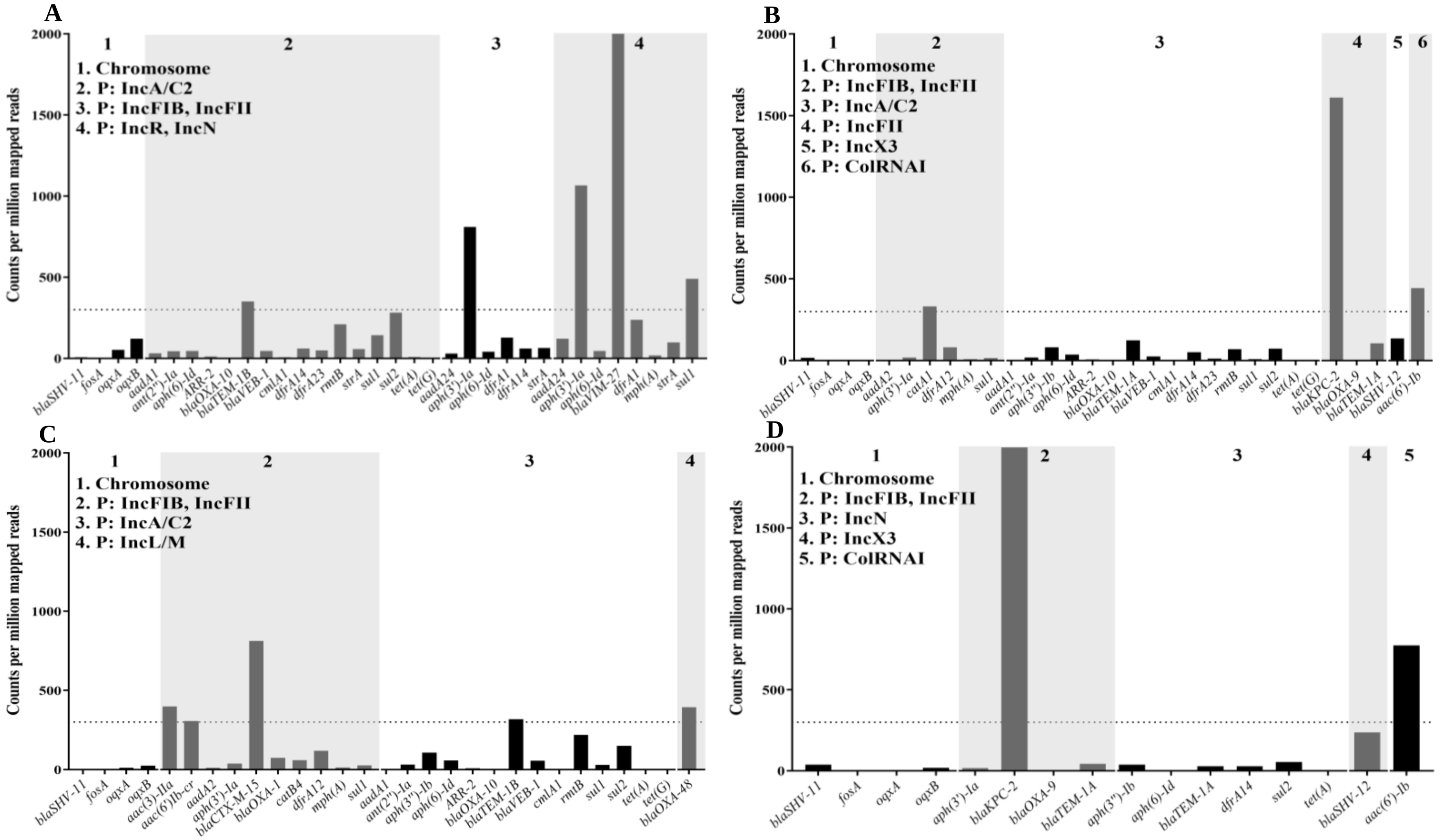

Figure 3

Click here to access/download;Figure;Figure_3.docx

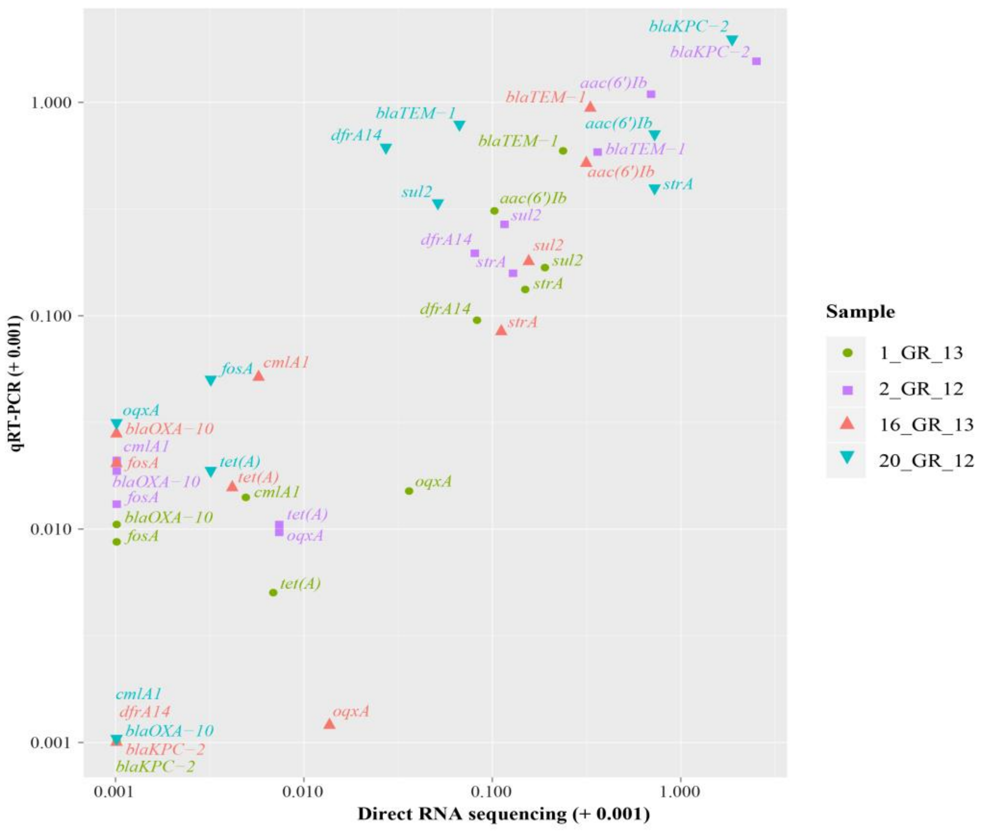

Figure 4

Click here to access/download;Figure;Figure_4.docx

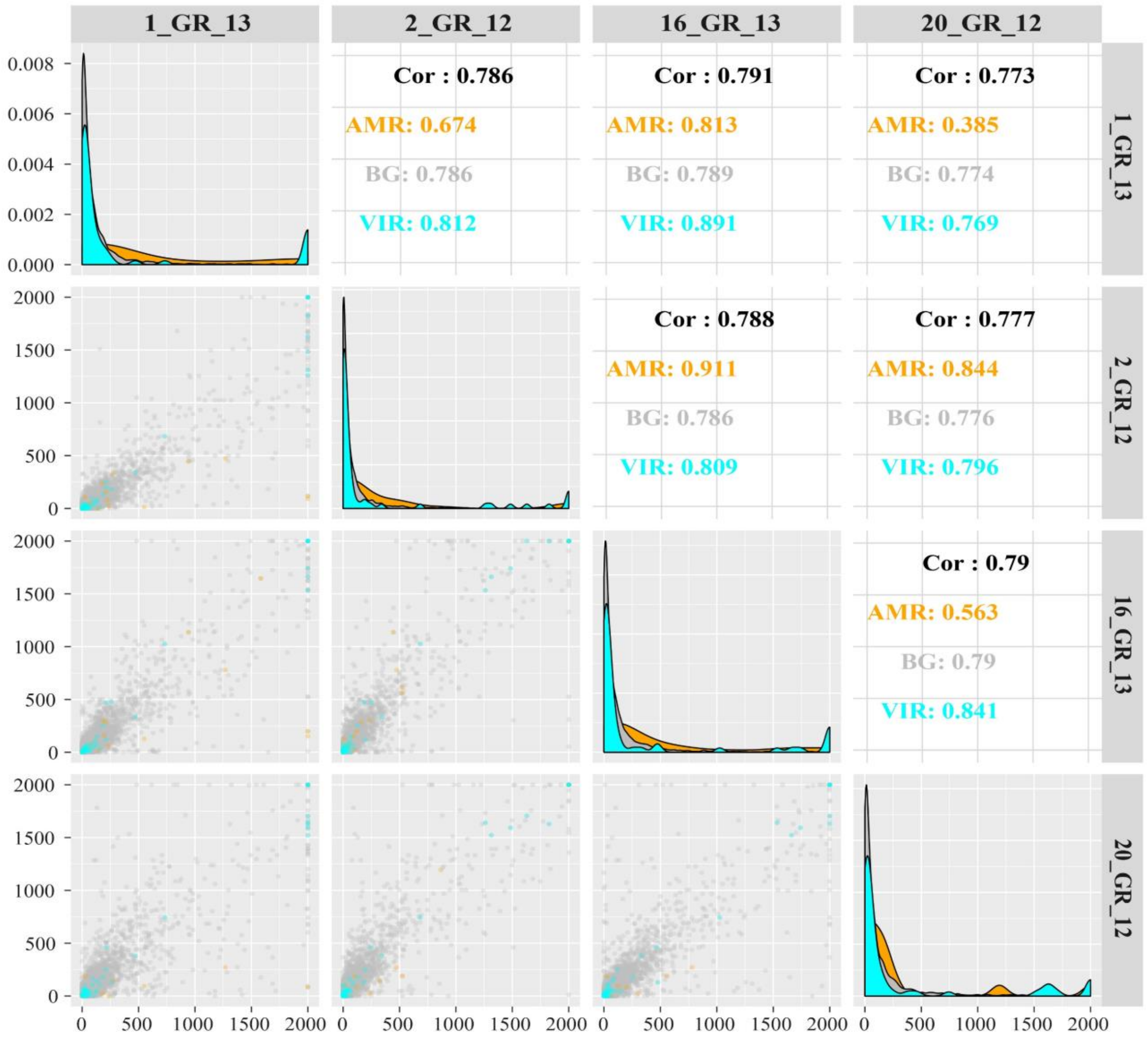

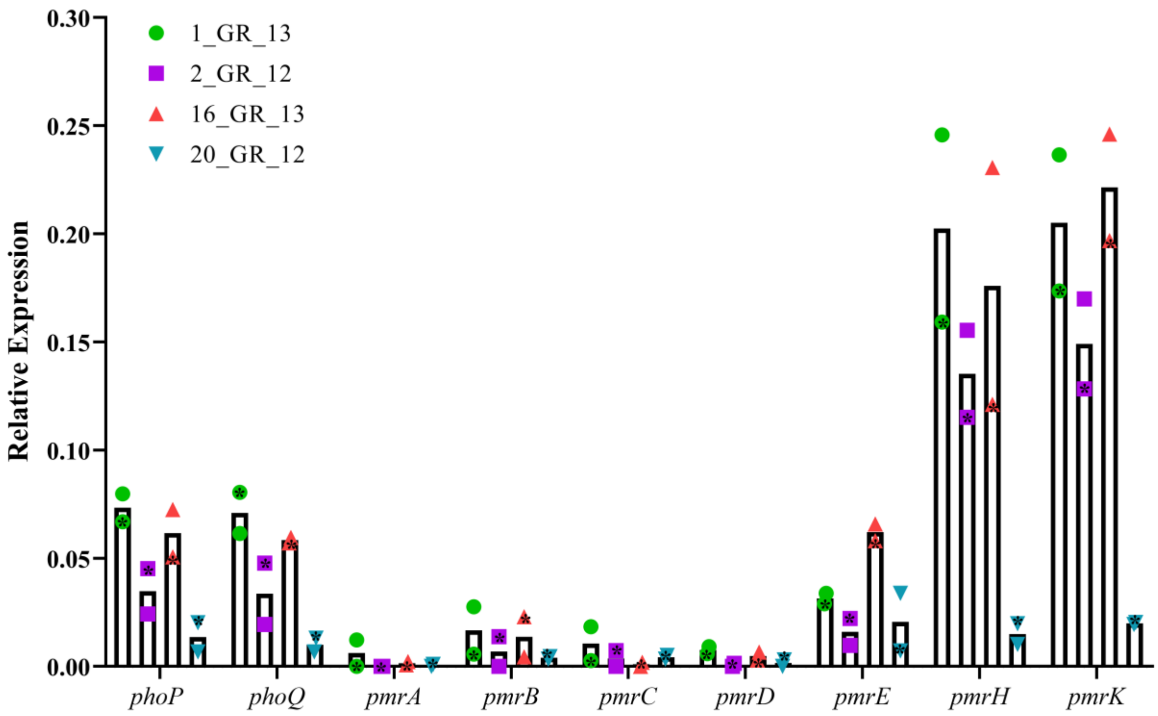
0.30
0.25
0.20
0.15
0.10
0.05
0.00
Relative Expression
1_GR_13
2_GR_12
16_GR_13
20_GR_12
phoP
phoQ
pmrA
pmrB
pmrC
pmrD
pmrE
pmrH
pmrK

Manuscript

Click here to access/download
Supplementary Material
#SI_GS_Evaluating_XDRKP#_FINAL.docx

 Click here to access/download;Personal Cover;#1_GIGA-D-19-00200_Response#_FINAL.docx

"Evaluating the Genome and Resistome of Extensively Drug-Resistant *Klebsiella pneumoniae* using Native DNA and RNA Nanopore Sequencing"
GIGA-D-19-00200
Response to Reviewers

Dear Dr. Scott Edmunds,

We thank the reviewers for the opportunity to revise this manuscript (GIGA-D-19-00200). Their comments have helped us significantly strengthen the work. We have now provided additional information including rationale for using direct RNA sequencing and particular analysis methodologies. Figures have also been modified to aid with the interpretation of data. To highlight the adjustments completed, we have also uploaded a mark-up version of the manuscript. Please find below a point-by-point response to the reviewers' comments.

Reviewer reports:

Reviewer #1:
In the manuscript "Evaluating the Genome and Resistome of Extensively Drug-Resistant *Klebsiella pneumoniae* using Native DNA and RNA Nanopore Sequencing" by Pitt et al., the authors describe datasets generated from multiple sequencing modalities of antibiotic-resistant clinical isolates, and discuss the potential of this technology for rapid detection of AMR. Although these methods and sequencing characterization and analysis are of importance to the field, there are several issues which remain to be addressed.

Specific points:
It would be useful to better establish the rationale for why direct detection of RNA transcripts matters, and what additional information direct RNA sequencing gets you that rapid cDNA conversion and sequencing can't. Perhaps the largest issue is - "Why dRNA-seq?" There doesn't seem to be an obvious benefit, given the poor time to detection compared to just DNA sequencing. Expression levels are useful, but could be determined from Illumina sequencing. Without splicing there are no isoforms to contend with, and the error rate adds difficulty in interpretation and determination of primary protein sequence. Additionally, most clinical bacterial characterization work doesn't use RNA-seq, and addressing the problems clearly (i.e. rRNA depletion, RNA instability) should be done at the outset.
Response: We have now provided additional information to highlight the benefits of using direct RNA sequencing in the introduction and discussion. The time to detect antibiotic resistance using direct RNA sequencing was slower compared to DNA, however, this is only the first generation of the technology. The latest kit, SQK-RNA002, has shown advancements in data generation which unfortunately was not available during the time of this study. "Our findings show that the slower time-to-detection of resistance genes in direct RNA sequencing was due to both the level of expression as well as the slower translocation speed, and hence using cDNA would only partially overcome this limitation." (Discussion: Line 396, also refer to Supplementary Figure S4). "Furthermore, library preparation time is halved for direct RNA sequencing due to the absence of cDNA synthesis" (Introduction: Line 57). Indeed, expression levels can be determined via Illumina sequencing, however, in the context of a diagnostic tool, Illumina platforms require the completion of the sequencing run (~48 hours) to output data and analysis to be performed.

Nanopore technologies can output data as soon as it is generated to enable real-time analysis. Although bacteria lack splicing, long read sequencing has the potential to detect operon sites where several transcripts are co-expressed (refer to Line 59 and 417). Due to difficulties extracting RNA from these strains and downstream processing for sequencing, these transcripts were short and not enough data was generated to confidently detect operon sites (Supplementary Figure S3). Furthermore, native RNA sequencing has the potential to detect RNA modifications associated with antibiotic resistance which are removed when converted to cDNA and is unique to this technology (Introduction: Line 55). Although RNA is unstable and requires several additional processing steps compared to DNA, advancements on this part could be made in the future and hence, the potential for this to be used to detect antibiotic resistance was explored. We have now made note of the limitations associated with RNA sequencing in the clinic (Discussion: Line 368). Additionally, RNA has the potential to determine the functionality of a resistance genes as the presence of these genes does not necessarily mean they confer resistance (Discussion: Line 369).

Under the "DNA extractions and HMW DNA isolation methods section", this section should be rewritten for clarity - it was confusing to determine which isolations worked and which didn't, and why. It's still important to include details of why protocol modifications were made, but if these could be incorporated into methods better that would aid in understanding.
Response: This section has now been rewritten (“High molecular weight DNA isolation”, page 4). Several modifications were implemented primarily due to difficulties lysing these highly antibiotic-resistant *K. pneumoniae* strains potentially due to a thickened capsule wall. This resulted in capsule contamination (carbohydrate) as determined via Nanodrop (Line 96). This was very cumbersome for isolate 2_GR_12 which was noted to have an increased carbohydrate contamination potentially due to the capsule and required a further purification step (Line 97).

Under "real-time resistome detection emulation" as well as "assembly of genomes" sections, it would be helpful to include a rationale on why certain software tools were chosen over others, given you tried many options. For example, why was BWA-MEM chosen over minimap2?
Response: In light of the vast amount of software tools available, we selected the four most commonly used tools for bacterial assembly. These incorporated both hybrid assemblers (Unicycler, npScarf) and the remaining two using only Nanopore reads (Canu, Minimap2/ Miniasm/ Racon). We trialed analysis using minimap2 initially, however, a lower alignment rate was observed potentially due to the majority of reads being less than 1000 bp (Supplementary Figure S3). This has now been mentioned in the supplementary section: Supplementary Table S6 and noted in the main text (Line 148) which also notes adjusted parameters used for BWA-MEM when using ONT reads.

How were you able to distinguish multiple copies of resistance genes from duplicated misassemblies?
Response: Both the fragment distribution (Supplementary Figure S1) and the read-length distribution (Supplementary Figure S3 A-D) indicate substantial number of reads of length greater than 10kb. The vast majority of bacterial repeats are shorter than 10kb, meaning that we are able to correctly place these repeats in the assembly. Furthermore, these long reads were able to span the duplicated resistance gene regions and correctly assemble these plasmids.

Would it actually be faster to detect with cDNA sequencing, given faster motor protein translocation rate and likely higher copy number of transcripts of interest? It would be useful to include thoughts on this in the discussion.
Response: While the sequencing speed of cDNA is currently faster than direct RNA (450 bases/second vs 70 bases per second) the library preparation for direct RNA is much quicker (105 minutes vs 270 minutes). Moreover, it is anticipated that future direct RNA sequencing kits will run at the same translocation speed as cDNA. We considered the translocation speed impeding on the detection method, hence, why we included an analysis total yield required to detect resistance genes as well as time to call the resistance genes (Line 266, Supplementary Figure S4). We have now added an additional sentence in the discussion: "Our findings show that the slower time-to-detection of resistance genes in direct RNA sequencing was due to both the level of expression as well as the slower translocation speed, and hence using cDNA would only partially overcome this limitation." (Line 396).

You say "Nanopore DNA sequencing currently has an accuracy ranging from 80 to 90%, which limits its ability to detect genomic variations", but there are post-processing tools available to increase accuracy and ability to detect SNVs - this should be included in the discussion.
Response: Agreed, there are tools to improve the accuracy which we have now made note of in the discussion: "However, software tools such as Nanopolish (https://github.com/jts/nanopolish) and Tombo (https://github.com/nanoporetech/tombo) (similarly used to re-train Chiron v0.5 for direct RNA sequencing data) have the potential to correct these reads and would be helpful to integrate to increase the accuracy of detecting resistance genes." (Line 359).

Further the detection of SNV mutations and indels is critical with respect to the detection of chromosomal mutations in these samples. Additional consideration of methylation signatures is crucial, as they can cause systematic error (PMID: 30373801) if not corrected.
Response: We have now noted the influence of DNA modifications on the accuracy of Nanopore sequencing and included this publication. "We utilised native DNA sequencing in this study which retains epigenetic modifications such methylation which can hinder the accuracy of reads and subsequent calling of antibiotic resistance [58]." (Line 362).

"All isolates exhibited low levels of expression for fosfomycin, macrolide and tetracycline resistance, despite exhibiting phenotypic resistance to fosfomycin and tetracycline", but are high levels of expression essential for phenotypic resistance? Are these low levels surprising? It would be helpful to link to papers discussing this.
Response: Additional information has now been included to identify why low expression of particular genes was observed. Limited literature is available on these specific genes in *K. pneumoniae* with transcriptional and antimicrobial susceptibility testing. We have included the following sentence regarding fosfomycin resistance facilitated via the *fosA* gene: "Noteably, Klontz *et al* identified that chromosomally integrated FosA, similarly observed in our study, from *K. pneumoniae* harboured a higher catalytic efficiency. A higher catalytic efficiency may reason why our strains only require a low abundance of expression and still retain fosfomycin resistance" (Line 382). Low levels of expression for tetracycline are not surprising as this resistance is well characterized and found to be inducible (antibiotic exposure is required for expression of genes). This has been reworded: "Genes *tet(A)* and *tet(G)* encode efflux pumps which, in the absence of tetracycline, are lowly expressed and the lack of antibiotic supplementation in this study confirms this

observation [61]. Detecting inducible resistance (antibiotic exposure required for gene expression) such as tetracycline resistance highlights one of the advantages of investigating the transcriptome." (Line 384)

Figure 5 - instead of switching back and forth between panels A and B, a scatterplot comparing the two directly like Fig 3 would be more useful.
Response: This figure has now been amended with the data on a single graph.

Why do you think only 23% RNA reads aligned? Did you try to identify the unaligned reads (like sort out contamination, noise)? It would be beneficial to include at least a blast/centrifuge style analysis trying to determine the source of the unaligned reads. Additionally, a k-mer analysis of the unaligned reads could help determine their origin.
Response: We identified that various failed reads were <10 bp (Supplementary Figure S3) which were filtered before alignment with BWA-MEM (k -11, seed length of 11 bp). Preliminary BLASTn analysis of unmapped reads identified a bacterial origin. The primary issue with the direct RNA sequencing data is the base-calling. When adapting Chiron v0.5 for this data, squiggle plots (raw nanopore data) identified insufficient trimming of the artificial poly(A). Furthermore, RNA modifications in bacteria remain largely unknown and this has the potential to interfere with the raw nanopore current change and subsequent base-calling. This has now been included in the discussion: "Limitations were observed when base-calling bacterial direct RNA sequencing and may be attributed to trimming the long artificial poly(A) tail and interference of RNA modifications." (Line 391).

How much of the poor alignment is due to the method of preparation (i.e. polyA tailing, etc.)? Did the authors perform optimization of the extraction and library prep for bacterial RNA? What about using an alternative tail and RNA adaptor?
Response: We trialed phenol/ chloroform RNA extractions however, this process was lengthy and resulted in a low yield of RNA and increased impurities. The PureLink RNA Mini Kit protocol is relatively quick (<30 mins/ sample). We attempted an on-column DNase treatment during this protocol but the best DNA depletion was observed using TURBO DNase which doesn't work on column (requires 37°C incubation). Our optimized RNA extraction resulted in Bioanalyzer RNA integrity scores of ≥8.5 which has now been included in Line 116 (RIN scale 0-10, 10 is no degradation using 16S and 23S pecks as reference). We considered altering the library preparation including using an adapter similar to Smith *et al* (reference 26) which recognizes the Shine-Dalgarno sequence, however, there are deviations in this sequence and multi antisense adapters would be required so all transcripts are sequenced. Hence, the poly(A) tailing kit was more feasible as it will tag all 3'transcripts which allows for only the native RNA strand to be sequenced. Unfortunately, we were unaware of the efficiency of the polymerase until post sequencing analysis was performed (Supplementary Figure S6), hence, a shorter incubation can be implemented for future studies.

Viral direct RNA seq has been done (PMID: 30765700 and 30258076 for example) - it would be good to cite these or related papers.
Response: The updated publication of PMID: 30765700 rather than the preprint has been included in the references and PMID: 30258076 was originally incorporated in the introduction as reference 24 (refer to Line 54 for references referring to viral direct RNA sequencing). To our knowledge, all the publications on direct RNA sequencing are in the references.

Some minor points:
"This research also established a methodology and analysis for bacterial direct RNA sequencing." is repeated in the conclusions.
Response: This duplicated sentence has now been removed from the conclusions section.

Figure 2 colorblocking is a little confusing - could be more straightforward to break up the figure into separate panels per strain contig, for example with a ggplot facet_grid.
Response: Figure 2 has now been modified so genes belonging to particular contigs are easier to identify. This included adjusting the transparency of the colorblocking and splitting the x-axis similar to the ggplot facet_grid format.

Reviewer #2:
This manuscript presents a rapid resistance-gene discovery experiment, using genome sequencing and assembly to identify potentially-active genes, combined with differential expression to determine drug-free resistome activity. This manuscript is differentiated from most other direct-RNA and cDNA nanopore research, in that it is the *expression* rather than the *structure* of the genes is evaluated here. Bearing in mind that I cannot comment much on the biology side of things, I consider this manuscript to be a reasonable presentation of the experimental work that has been described, and recommend that it be accepted pending minor changes to figures, and clarification of multi-mapping results. I would like to thank the authors for making their Nanopore sequence data public prior to review submission; it demonstrates a good open research ethic.
My specific comments regarding the manuscript follow:
** Text **
L133: This references a fairly old version of Canu (i.e. v1.5), which seems a bit strange given that Guppy v3.0.3 is also mentioned (L260). I note that Canu v1.8 was released before Guppy v3.0.3, and would be interested to know why this version of Canu was chosen.
Response: Genome assemblies were conducted initially in this study and the transcriptomics at a later date. As we were able to complete the assemblies adequately using the hybrid assembler Unicycler and utilize Illumina reads to correct ONT sequencing errors, we did not run analysis on the most recent version of Canu. Furthermore, Guppy was integrated later as we had multiple issues with the base-calling of direct RNA sequencing and we hoped this update in the software would ameliorate this problem.

L144: I don't have an encyclopaedic knowledge of bwa-mem command-line options. It would be helpful to explain what the options mean. I'm particularly interested in why the default options were not appropriate, and what (if any) compensations were made for multi-mapped reads.
Response: This section has now been updated: "Similar parameters to the BWA-MEM ont2d function were used but seed length was reduced (-k 14) to compensate for shorter reads: -k 11 [minimum seed length, bp] -W20 [bandwidth] -r10 [gap extension penalty] -A1 [match score] -B1 [mismatch penalty] -O1 [Gap open penalty] -E1 [Gap extension penalty] -L0 [Clipping penalty]). Multi-mapping reads were removed via SAMtools (secondary alignment: flagged as 256)…" (Line 149).

L144: Why was minimap2 not used here? It was written by the same author as bwa-mem, but is specifically written to incorporate corrections to improve mapping for noisy Nanopore Direct RNA-seq [e.g. see https://github.com/lh3/minimap2#getting-started]

Response: Preliminary analysis using minimap2 showed fewer reads aligning to the reference (now noted in the legend of Supplementary Table S6). It has been noted by Li H (doi: 10.1093/bioinformatics/bty191) that BWA-MEM is more suited to short read data and has a slightly improved accuracy compared to minimap2. We've further noted the bias towards BWA-MEM in Line 148: "BWA-MEM was selected due to shorter transcripts being produced by bacteria (Supplementary Figure S3) and the lack of introns and alternative splicing."

L145: I notice from L198 that there are gene copies in the data, with potentially high identity. Is there a particular reason why reads were mapped to the genome, rather than to transcriptome that merges essentially-identical genes?

Response: As described in the "Real-time resistome detection emulation" section (line 127), the resistance gene detection was carried out by mapping to a database of resistance genes which was clustered based on 90% identity threshold. However, in the section "RNA alignment and expression profiling" (Line 146) we mapped reads to the genome. In this case, if a read mapped to multiple locations equally well, then BWA-MEM randomly allocates to one position (primary alignment). Several instances of multiple copy numbers of resistance genes (Line 215) occurred which will influence the quantification of expression when aligned to the genome. Interestingly, there were some slight deviations in the expression of perfectly duplicated genes with unique flanking regions (refer to *strA* and *sul1* in Figure 2A, contig 2 and 4) which may indicate that these genes are controlled by an operon (co-transcribed genes). This is an advantage of aligning to the genome. We also took this into consideration when graphing Figure 3 and combined all reads mapping to duplicated genes, such as *strA*, before normalizing to a housekeeping gene (*rpsL*).

L153: Why was a more well-known differential expression package not used here (e.g. DESeq2 or EdgeR) for evaluating differential expression? Is there an advantage of VGAM for plasmid or small genome differential expression?

Response: The beta-binomial distribution (implemented in VGAM) was used as a statistic to identify genes with significantly fewer or greater reads mapping in one sample versus another. It was chosen because it represents the uncertainty in the proportion estimated from count data. However, we agree that EdgeR and DESeq2 are also able to adequately estimate this uncertainty and hence we have redone the analysis using EdgeR (Supplementary Figure S7, Methods: "Whole transcriptome gene expression and estimation of expression confidence intervals", Line 157). The list of differentially expressed genes is very similar to that identified using VGAM (at least 90% identical).

L198 (see also L145): How identical were these genes? Would this identity affect genome mapping? In situations with multiple copies of near-identical genes, do you have any evidence to suggest that only one copy was active?

Response: These genes are 100% similar and will impact mapping to the genome. Unless expressed by an operon and the full-length sequences are retrieved, only then could this distinguish which genes are active. This issue will still arise if transcripts are mapped to the transcriptome. The only definitive way to determine this would be to perform knock-down studies of these regions and subsequently evaluate expression.

L218: What was the MAPQ probability for these genes? If the MAPQ probability is less than 3, it means that a gene could be equally-well placed at least two different sites (-log10(0.5) *10 ~= 3), which is expected given the gene duplication in your assemblies. I don't think this would indicate that the mapping is bad, as such, although there may be other reasons for a poor mapping.
Response: Agreed, the MAPQ score was commonly ≤10 for these duplicated reads. We have made a note of low mapping quality due to multiple copies of genes: "Low mapping quality could be attributed to assignment of reads to multiple copies of genes in the genome. Furthermore, the ONT error rates could lead to misassignment of reads to genes." (Line 275).

L228 (see also L198): more information about the similarity between the "correct" and "incorrect" gene would be useful; I notice that L335 mentions an identity for some genes of "greater than or equal to 80%". Do you have other evidence that systematic sequencing error would lead to reads being assigned to the incorrect gene?
Response: Various resistance genes harbor ≥80% similarity when taking into consideration genes deposited on the ResFinder database. In several instances, this is only 1 nucleotide and if sequencing errors arise, have the potential for misidentification. We can determine this accumulation of sequencing errors via observing the real-time emulation for DNA sequencing in Supplementary S5. After 5 hours (300 minutes), we could witness multiple genes being detected that were not identified in the final assembly and the Illumina only SPAdes assembly.

L245 (see also L218): Were there multiple fosA transcripts in the genome? I can't see from Table 1 any indication of this, but maybe it's not clear enough for me. If not, can you suggest other reasons for the low MAPQ score? It seems like a lot of results are being thrown away because the MAPQ is low.
Response: Only one copy of *fosA* is encoded on the chromosome for all isolates (Line 194). All genes with multiple copies have been noted in Line 215. The mapping quality is most likely due to the low expression of this gene and difficulties with base-calling (issues removing the long artificial poly(A) tail and interference of RNA modifications (Line 393). Once base-calling tools have been optimized for bacterial direct RNA sequencing, MAPQ scores will be a better quality.

L336 (see also L228 and L198): Would 80% identity lead to a misclassification by BWA-MEM?
Response: Yes, as some genes are very similar (potentially only one nucleotide difference), this has the potential to result in misclassification of resistance genes in the real-time emulation. Especially when we identified a 10% error rate in our ONT DNA sequencing (Line 356) and ≤23% for direct RNA sequencing (Line 394).

L341: I get a bit frustrated by people discussing accuracy from previous (typically quite old) nanopore papers as if it were a fixed thing, especially in a study that has produced a lot of other nanopore data. Nanopore technology changes quickly, and basecalling accuracy has made substantial improvements in particular over the last year. I'm not convinced a paper published in January 2018 would give a good estimate for accuracy called with guppy 3.0.3 (or 3.1.5, which is the latest that I'm aware of at the time of this review). Feel free to cite it, but I'd like to know [in the same breath] what the direct RNA accuracy was in *your* reads. L260-264 briefly discuss using different base-callers; how does that accuracy change depending on the base-caller?

Response: We have now included information regarding accuracy between base-callers: "Albacore 2.2.7 had the highest average accuracy across isolates (84.87%) closely followed by Guppy 3.0.3 (84.62%) and then Chiron v0.5 (78.19%) (Supplementary Table S6)." Line 279. The abstract also notes that we could identify accuracy up to 86% for direct RNA sequencing (Line 20).

** Figures **
Figure 1:
- Would work better as a side-by-side bar plot. The split graph makes it look like one side is negative, and the other side is positive.
- Order by colour / class rather tham abundance, with brackets indicating classifications.
Response: We initially considered side-by-side bar plots however, this would result in approximately 40 bars on the y-axis which is difficult to follow. We have now split the x-axis to better delineate between DNA and RNA data. Furthermore, an overlay of this data based on yield rather than time has been included in the supplementary results (Figure S4). The main text is written in the context of time to detect a particular gene conferring resistance to an antibiotic class, hence, why we ordered this as time of detection rather than grouping the antibiotic classes.

Figure 2:
- This figure is unclear to me. If this figure is relative expression (e.g. the statistic used for the correlation plot in Figure 3), then the presented data should be relative proportions, probably in log space (e.g. log2(gene/rpsL)).
- Why was rpsL chosen for normalisation?
Response: Unfortunately, the wrong figure legend was included for Figure 2 and has been amended. This data is counts per million (cpm) mapped reads rather than normalized to *rpsL*. We didn't adjust to relative proportions for this figure (or Figure 4, which is also in cpm) as the main text mentions cpm values. However, for comparisons of direct RNA to qRT-PCR (e.g. Figure 3 and Figure 5) we did normalize relative to housekeeping gene *rpsL*. This housekeeping gene has been used previously in literature (reference 46). We also have data for another housekeeping gene, *rpoB*, which generated similar results.

Figure 3:
- Were there any sample replicates? Are you able to estimate error in any measurements?
- The colour is confusing for this graph. You could try gene name for colour, and different plot symbols for different samples.
Response: All qRT-PCR measurements were done in triplicates (Line 170). There are no sample replicates for direct RNA sequence data. This is because the primary aim of the paper is to evaluate time-to-detection of antibiotic resistance genes across multiple samples (emulating a clinical setting in which a single replicate would be sequenced for each sample, particularly in the context of not having access to direct RNA multiplexing and so running a single sample in a single flow cell). However, we can estimate variation in the proportion of reads mapping to each gene (and hence the counts-per-million) by assuming the observed read counts are generated from a binomial distribution, so we can estimate a 90% CI in the expression levels using the conjugate beta prior. We show these estimates in Supplementary Figure S7.

Regarding the colours, there are 4 samples and eleven genes, so we didn't think colouring by gene would work (too many genes). We selected to colour by sample, and indicate the gene names on the plot. We have followed the suggestion of using different symbols per isolate.

Figure 4:
- What do the bottom panels describe (e.g. gene expression level scatter plots comparing each sample with each other sample)? This is not stated in the figure legend.
Response: Yes, the bottom panels include the expression levels between differing isolates in a scatter plot. This has now been added to the legend.

Figure 5:
- I recommend changing this to a side-by-side bar plot, as the text indicates that the comparison of A vs B is important.
Response: This figure has now been amended with the data on a single graph.

Reviewer #3:
The manuscript by Pitt et al interrogated the genome and transcriptome of PDR and XDR *K. pneumoniae* isolates using the Oxford Nanopore MinION device. This is the very first study which adopted nanopore approaches in direct bacterial mRNA sequencing. The authors established a methodology for adding poly(A) tail onto mRNA transcripts which will benefit future bacterial sequencing and diagnosis related studies. However, authors failed to explain clearly the advantage of using Nanopore for RNA sequencing to Illumina platform. In another word, why we need to develop RNA sequencing using Nanorpore since it is not an efficient way to do it and very complicated. In addition, the manuscript indeed showed that the coverage of RNA seq is very low and the correlation is not good. In my view, if there is no specific need to do RNA seq using Nanopore platform, there is no need to develop it since the Illumina platform is very good already in this application.
Response: Please refer to our first response to Reviewer #1.

In addition, I also have the following major comments:
1. Line 169, section "Antibiotic resistance and the location of acquired resistance in the genome "The authors reported the AMR genes and their location in this section. Since this is a technical manuscript, can the authors provide some sequencing information? The volume of data generated with time, coverage of each sequenced sample, the accuracy of the sequence, and the comparison of different assembly methods could be briefly discussed.
Response: We've now included additional information regarding the DNA sequencing: "MinION DNA sequencing for all isolates was run for ≥20 hours which generated 1.19 GB (215X) for 1_GR_13, 0.39 GB (67X) for 2_GR_12, 0.56 GB (101X) for 16_GR_13 and 0.64 GB (115X) for 20_GR_12 (Supplementary Table S2). Across the differing assembly tools, the chromosome sequence commonly circularised as a 5.0-5.4 Mb contig including plasmids ranging between 13-193 kb with the exception of 2_GR_12. Aligning ONT reads to the final assembly revealed that this DNA sequencing had a 90% accuracy rate across isolates." (Line 184) A comparison of several assembly methods is given in Supplementary Table 2, but we don't discuss this in much detail in the paper as it is not the focus of this work.

2. Line 256, only a low proportion of these RNA sequencing reads passed base-calling. Is it also related to the sample preparation apart from the inaccuracy of the base-calling software?

Response: Indeed, RNA sample preparation could influence the subsequent quality of the data and we attempted several protocol optimizations. We trialed phenol/ chloroform RNA extractions however, this process was lengthy and resulted in a low yield of RNA and increased impurities. The PureLink RNA Mini Kit protocol is relatively quick (<30 mins/ sample). We attempted an on-column DNase treatment during this protocol but the best DNA depletion was observed using TURBO DNase which doesn't work on column (requires 37°C incubation). Our optimized RNA extraction resulted in Bioanalyzer RNA integrity scores of ≥8.5 which has now been included in Line 116 (RIN scale 0-10, 10 is no degradation using 16S and 23S pecks as reference). Unfortunately, we were unaware of the efficiency of the polymerase until post sequencing analysis was performed (Supplementary Figure S6), hence, a shorter incubation can be implemented for future studies. However, the majority of inaccuracy appears to be due to the base-calling software unable to accurately trim the long artificial poly(A) tail and potential interference to the raw read signal via RNA modifications (Line 391).

3. Would the authors compare the genome and transcriptome a little bit to link these data?

Response: We have drawn various comparisons between the genome and transcriptome to link the sequencing data. In particular, tables and figures comparing both RNA and DNA include Figure 1, Table S5, Figure S3 and Figure S4 with corresponding sections in the main text. Additional information in the discussion has been provided to highlight the pros and cons regarding interpreting antibiotic resistance using either DNA or RNA. "We further investigated the transcriptome of these isolates to potentially elucidate the correlation between genotype and the subsequent resistant phenotype. Detection of antibiotic resistance via sequencing commonly uses DNA due to the instability of RNA and the lengthy sample processing such as rRNA depletion [12-15, 58]. However, RNA provides additional information regarding the functionality of genes such as identifying conditions in which a resistance gene is present but not active which gives rise to a false positive via DNA alone. Conversely, if expression is only induced in the presence of an antibiotic, the absence of RNA transcripts results in a false negative." (Line 367). "Furthermore, the time required to detect resistance may be hindered by the slower translocation speed associated with direct RNA sequencing (70 bases/ second) compared to DNA sequencing (450 bases/ second) [57]. Although cDNA would overcome this limitation, our findings show that detection was primarily due to level of expression when evaluating data yield rather than time." (Line 394).

4. Line 381, "a number of resistance genes were identified that were not present in the final assembly. The authors were expected to discuss why this happens and how to deal with these false positive data.

Response: The discussion on this topic has now been extended: "Furthermore, a small number of resistance genes were identified that were not present in the final assembly, however these all had MAPQ values less than 10 and less than 30 mapped reads. Some of these may be due to low-level kit contamination, while some of the false positives have sequence similarity to true positives and may be due to inaccuracies in base-calling." (Line 363).
