# Supplementary material for: Evaluating the genome and resistome of extensively drug-resistant Klebsiella pneumoniae using native DNA and RNA Nanopore sequencing
Source: Gigascience. 2020 Feb 4;9(2):giaa002. doi: 10.1093/gigascience/giaa002 (PMC6998412; doi:10.1093/gigascience/giaa002)
Supplement: giaa002_GIGA-D-19-00200_Revision_2 [file giaa002_giga-d-19-00200_revision_2.pdf]

GigaScience

Evaluating the Genome and Resistome of Extensively Drug-Resistant Klebsiella pneumoniae using Native DNA and RNA Nanopore Sequencing

--Manuscript Draft--

**Manuscript Number:**	GIGA-D-19-00200R2
**Full Title:**	Evaluating the Genome and Resistome of Extensively Drug-Resistant Klebsiella pneumoniae using Native DNA and RNA Nanopore Sequencing
**Article Type:**	Research
**Funding Information:**	Institute for Molecular Bioscience Centre for Superbug Solutions (610246) — Prof Lachlan J.M. Coin
**Abstract:**	Background : *Klebsiella pneumoniae* frequently harbours multidrug resistance and current diagnostics struggle to rapidly identify appropriate antibiotics to treat these bacterial infections. The MinION device can sequence native DNA and RNA in real-time, providing an opportunity to compare the utility of DNA and RNA for prediction of antibiotic susceptibility. However, the effectiveness of bacterial direct RNA sequencing and base-calling has not previously been investigated. This study interrogated the genome and transcriptome of four extensively drug-resistant (XDR) *K. pneumoniae* clinical isolates, however, further antimicrobial susceptibility testing identified three isolates as pandrug-resistant (PDR).<br>Results: The majority of acquired resistance (≥75%) resided on plasmids including several megaplasmids (≥100 kbp). DNA sequencing detected most resistance genes (≥70%) within 2 hours of sequencing. Neural-network based base-calling of direct RNA achieved up to 86% identity rate, although ≤23% of reads could be aligned. Direct RNA sequencing (with approximately 6 times slower pore translocation) was able to identify (within 10 hours) ≥35% of resistance genes, including those associated with resistance to aminoglycosides, β-lactams, trimethoprim and sulphonamide and also quinolones, rifampicin, fosfomycin and phenicol in some isolates. Direct RNA sequencing also identified the presence of operons containing up to 3 resistance genes. Polymyxin-resistant isolates showed a heightened transcription of *phoPQ* (≥2-fold) and the *pmrHFIJKLM* operon (≥8-fold). Expression levels estimated from direct RNA sequencing displayed strong correlation (Pearson: 0.86) compared to qRT-PCR across eleven resistance genes.<br>Conclusion: Overall, MinION sequencing rapidly detected the XDR/ PDR *K. pneumoniae* resistome and direct RNA sequencing provided accurate estimation of expression levels of these genes.
**Corresponding Author:**	Lachlan Coin<br><br>AUSTRALIA
**Corresponding Author Secondary Information:**	
**Corresponding Author's Institution:**	
**Corresponding Author's Secondary Institution:**	
**First Author:**	Miranda E. Pitt
**First Author Secondary Information:**	
**Order of Authors:**	Miranda E. Pitt
	Son H. Nguyen
	Tânia P.S. Duarte
	Haotian Teng
	Mark A.T. Blaskovich
	Matthew A. Cooper

Powered by Editorial Manager® and ProduXion Manager® from Aries Systems Corporation

	Lachlan J.M. Coin
**Order of Authors Secondary Information:**	
**Response to Reviewers:**	"Evaluating the Genome and Resistome of Extensively Drug-Resistant Klebsiella pneumoniae using Native DNA and RNA Nanopore Sequencing"<br>GIGA-D-19-00200_R2<br>Response to Reviewers<br><br>Dear Dr. Scott Edmunds,<br><br>We thank the reviewers for the opportunity to implement additional revisions in our manuscript (GIGA-D-19-00200) which has enabled us to further clarify and strengthen this research via amending figures and conducting further analysis. Please find below a point-by-point response to the reviewer comments.<br><br>Reviewer reports:<br><br>Reviewer reports:<br>Reviewer #1: The authors' response to our comments and suggestions has improved this manuscript. We are generally satisfied with the manuscript edits and improvements; however, there are a few more modifications, particularly in the presentation of results, that we feel are required to make this paper acceptable for publishing.<br><br>1. The response to pt 1 - (why dRNA) is still largely unsubstantiated. The library prep time being cut in half is a slight advantage, but with cDNA we can start from less material (i.e. perform PCR). Multioperon sequencing could be shown from cDNA as easily as from dRNA - and the authors still haven't shown it. And the authors suggest RNA modifications could be detected, arguably the most unique advantage to dRNA seq in prokaryotes - but showed none of this. We recommend rewording rationale to avoid discouraging cDNA sequencing, especially since it is more practical for most applications at this point - and since the promised improvements for dRNA have yet failed to materialize from ONT, and cDNA yield is currently substantially better.<br>Response: We agree and have run additional analysis to detect operons (co-expression of genes) using BEDTools intersect which can be found in the results section: "Several resistance genes were identified to be regulated by operons and co-expression was evident for oqxAB (1_GR_13, 16_GR_13), blaVEB-1:ant(2")-Ia:ARR-2 (1_GR_13), aadA1:sul1 (1_GR_13), rmtB:blaTEM-1B (1_GR_13, 2_GR_12, 16_GR_13), aph(6)-Id:strA (1_GR_13), sul2:aph(3")-Ib:aph(6)-Id (2_GR_12, 16_GR_13), ant(2")-Ia:blaVEB-1 (2_GR_12, 16_GR_13), aac(6')-Ib-cr:blaOXA-1:catB4 (16_GR_13), aadA2:sul1 (16_GR_13) and sul2:aph(3")-Ib:dfrA14 (20_GR_12) (Figure 2). Overall, various non rRNA genes were identified to be co-expressed (≥5 reads supporting gene intersect) across isolates (1_GR_13: 428; 2_GR_12: 310; 16_GR_13: 793; 20_GR_12: 442)." (Line 301-307). We have also uploaded the complete list of operons (including rRNA genes): "GIGA-D-19-00200_operons_+rRNA.xlsx".<br>Unfortunately, there is currently no robust approach to detect RNA modifications using ONT direct RNA sequencing. The prior study by Garalde et al (2018) only interrogated one gene with a known modification and could compare to the same sequence lacking this modification (unmodified). The m6A and A-to-I editing modification can potentially be detected in recent studies (Workman RA et al (2019) https://doi.org/10.1038/s41592-019-0617-2. Liu H et al (2019) https://doi.org/10.1038/s41467-019-11713-9), however, these studies also had an unmodified dataset for comparison. Generation of the unmodified dataset usually requires in vitro transcribed RNA or modification sites can be determined using immunoprecipitation sequencing. These approaches can be costly and time consuming to generate on a whole transcriptome scale. As this dataset has not been generated on our isolates, we are unable to accurately call RNA modifications and hence, why we have not included this in our study. We have reworded the conclusion to highlight some shortcomings of ONT native RNA sequencing: "The expression of resistance genes was successfully detected in addition to identifying genes potentially regulated via operons, however, native RNA sequencing incurs a slower time to detect resistance due to translocation speed. Once base-calling algorithms have been optimised, this could allow for a whole transcriptome interrogation of the poorly characterised bacterial RNA modifications." Line 423-427.

	2. In the "levels of expression of resistance genes" section, lines 270-274, it would be useful to include read counts alongside percentages aligned to increase transparency for counts of reads included in the analysis.<br>Response: The read counts have now been included in this section. In some instances, a read range was used rather than all the individual values however, the full list of read counts can be found in the Supplementary material (Supplementary Table S6). Line 273-277.<br><br>3. In line 281 the authors state "These results reflect the fact that base-calling algorithms have not 282 yet been optimised for direct RNA sequencing, and even less so for bacterial RNA sequencing". However, the accuracies reported in the line above are not atypical of single molecule sequencing, and low alignment % seems largely driven by 400-700nt poly-A tails added- recommend amending this statement.<br>Response: This line has now been modified: "However, low alignment rates could be attributed to the addition of a long artificial poly(A) which was identified to be approximately 400 to 700 bp across isolates (Supplementary Figure S6)" Line 285-287.<br><br>4. More importantly, the alignment % is still alarmingly low - even with current RNA basecallers you should be seeing 80-90% alignment. We recommend filtering reads before alignment by only using "pass" reads, trimming poly-A tails off reads, and removing small reads less than 75nt (which are more likely to be noise), then reporting this alignment percentage.<br>Response: We now report the alignment rate on pass reads only after poly(A) trimming and removing the small reads less than 75nt, and find that the percentage is actually quite reasonable (≥98% alignment rate). These results have been included in the main text: "Aligning passed reads alone to the final assembly (ensuring trimming of poly(A) tail and removing reads <75 nt), ≥98% (1_GR_13: 95591; 2_GR_12: 138214; 16_GR_13: 227781; 20_GR_12: 119425) of reads were mappable, however, ≤46% (1_GR_13: 42654; 2_GR_12: 46787; 16_GR_13: 79175; 20_GR_12: 54986) of these had a MAPQ score ≥10." Line 274-277.<br><br>5. Figure 3- Please denote on the figure itself which primers recognize more than one gene, maybe with underlining? Also, what does the +0.001 notation on axes mean?<br>Response: Primers which recognize more than one gene have now been underlined in Figure 3. The data in this Figure has been log10 transformed hence, to show genes with no detectable expression, the data was shifted by +0.001. This has now been noted in the legend of Figure 3.<br><br>6. Figure 5 is overly complicated with the shapes and colors and asterisks - why not just plot the data in the same way as Figure 3, and you can facet by sample?<br>Response: Figure 5 has now been graphed similar to Figure 3.<br><br>Small things:<br>Spell out XDR first (line 329)<br>Response: Line 329 has now been amended. (Line 338).<br>Please parse paragraph lines 348-388 into DNA and RNA sections<br>Response: This section has now been modified to separate DNA and RNA. (Line 375).
**Additional Information:**	
**Question**	**Response**
Are you submitting this manuscript to a special series or article collection?	No
**Experimental design and statistics**<br><br>Full details of the experimental design and statistical methods used should be given in the Methods section, as detailed in our Minimum Standards Reporting Checklist. Information essential to interpreting the	Yes

data presented should be made available in the figure legends.<br><br>Have you included all the information requested in your manuscript?	
**Resources**<br><br>A description of all resources used, including antibodies, cell lines, animals and software tools, with enough information to allow them to be uniquely identified, should be included in the Methods section. Authors are strongly encouraged to cite Research Resource Identifiers (RRIDs) for antibodies, model organisms and tools, where possible.<br><br>Have you included the information requested as detailed in our Minimum Standards Reporting Checklist?	Yes
**Availability of data and materials**<br><br>All datasets and code on which the conclusions of the paper rely must be either included in your submission or deposited in publicly available repositories (where available and ethically appropriate), referencing such data using a unique identifier in the references and in the “Availability of Data and Materials” section of your manuscript.<br><br>Have you have met the above requirement as detailed in our Minimum Standards Reporting Checklist?	Yes

Manuscript
Click here to access/download;Manuscript;###GS_Evaluating the Resistome of XDR KP###_R2.docx

Evaluating the Genome and Resistome of Extensively Drug-Resistant *Klebsiella pneumoniae* using Native DNA and RNA Nanopore Sequencing

Miranda E. Pitt[1], Son H. Nguyen[1], Tânia P.S. Duarte[1], Haotian Teng[1], Mark A.T. Blaskovich[1], Matthew A. Cooper[1], Lachlan J.M. Coin[1]

[1] Institute for Molecular Bioscience, The University of Queensland, Brisbane, Queensland, 4072, Australia

Corresponding authors: Miranda Pitt (miranda.pitt@imb.uq.edu.au, ORCID: 0000-0002-8255-4036) and Lachlan Coin (l.coin@imb.uq.edu.au, ORCID: 0000-0002-4300-455X)

Other ORCID IDs:

Haotian Teng: 0000-0001-8711-1587; Mark A.T. Blaskovich: 0000-0001-9447-2292; Matthew A. Cooper: 0000-0003-3147-3460

Abstract

Background: *Klebsiella pneumoniae* frequently harbours multidrug resistance and current diagnostics struggle to rapidly identify appropriate antibiotics to treat these bacterial infections. The MinION device can sequence native DNA and RNA in real-time, providing an opportunity to compare the utility of DNA and RNA for prediction of antibiotic susceptibility. However, the effectiveness of bacterial direct RNA sequencing and base-calling has not previously been investigated. This study interrogated the genome and transcriptome of four extensively drug-resistant (XDR) *K. pneumoniae* clinical isolates, however, further antimicrobial susceptibility testing identified three isolates as pandrug-resistant (PDR).

Results: The majority of acquired resistance (≥75%) resided on plasmids including several megaplasmids (≥100 kbp). DNA sequencing detected most resistance genes (≥70%) within 2 hours

of sequencing. Neural-network based base-calling of direct RNA achieved up to 86% identity rate, although ≤23% of reads could be aligned. Direct RNA sequencing (with approximately 6 times slower pore translocation) was able to identify (within 10 hours) ≥35% of resistance genes, including those associated with resistance to aminoglycosides, β-lactams, trimethoprim and sulphonamide and also quinolones, rifampicin, fosfomycin and phenicol in some isolates. Direct RNA sequencing also identified the presence of operons containing up to 3 resistance genes. Polymyxin-resistant isolates showed a heightened transcription of *phoPQ* (≥2-fold) and the *pmrHFIJKLM* operon (≥8-fold). Expression levels estimated from direct RNA sequencing displayed strong correlation (Pearson: 0.86) compared to qRT-PCR across eleven resistance genes.

Conclusion: Overall, MinION sequencing rapidly detected the XDR/ PDR *K. pneumoniae* resistome and direct RNA sequencing provided accurate estimation of expression levels of these genes.

Introduction

Klebsiella pneumoniae is one of the leading causes of nosocomial infections, with reports of mortality rates as high as 50% [1-5]. This opportunistic pathogen commonly exhibits multidrug resistance which severely limits treatment options [6]. A high abundance of resistance is frequently encoded on plasmids, accounting for the rapid global dissemination of resistance [1,6]. Common therapeutic options for multidrug-resistant infections include carbapenems, fosfomycin, tigecycline and polymyxins [7]. However, resistance is also rapidly developing against these antibiotics resulting in the emergence of extensively drug-resistant (XDR) and subsequent pandrug-resistant (PDR) strains [6-9].

One of the major contributors to the advent of antibiotic resistance is the inability for current detection methodologies to readily and accurately assess bacterial infections in particular, the

resistance profile [10]. Rapid sequencing has been proposed as a way to determine antibiotic resistance, including approaches which utilise high accuracy short reads, as well as those which exploit real-time single-molecule sequencing such as Oxford Nanopore Technologies (ONT). The ONT MinION platform is a portable single-molecule sequencer which can sequence long fragments of DNA and stream the sequence data for further data processing in real-time, detecting the presence of bacterial species and acquired resistance genes [11-15]. Moreover, the long reads coupled with the ability to multiplex samples has immensely aided with the assembly of bacterial genomes [16-18]. This capability allows for the rapid determination of whether resistance is residing on the chromosome or plasmid/s. Of particular interest are high levels of resistance encoded on plasmids, as these genes can rapidly be transferred throughout the bacterial population via horizontal gene transfer. However, a limitation of DNA sequencing is accurately identifying whether the presence of an acquired resistance gene or mutation is facilitating resistance.

ONT has recently released a direct RNA sequencing capability, which sequences native transcripts. Other sequencing technologies rely on fragmentation, cDNA conversion and PCR steps that create experimental bias and hinder the accuracy of determining gene expression [19, 20]. The ability for MinION sequencing to read long fragments enables full length transcripts to be investigated. To date, only a few direct RNA sequencing publications exist which include eukaryote transcriptomes, primarily yeast (*Saccharomyces cerevisiae* [19, 21]) and recently, *Homo sapiens* [22]. This sequencing has additionally been implemented in viral transcriptomics [23-25]. Only one prior study by Smith AM *et al.* has applied this sequencing to bacterial 16S ribosomal RNA (rRNA) to detect RNA modifications [26]. Notably, resistance to certain antibiotics, such as aminoglycosides, can arise via RNA modifications which are unable to be detected once RNA is converted to cDNA [26]. Furthermore, library preparation time is halved for direct RNA

sequencing due to the absence of cDNA synthesis. Bacterial transcription differs significantly from eukaryotes in that transcription and translation occur simultaneously. As a result, bacterial mRNA transcripts lack poly(A) tails and alternative splicing, however, genes can be co-transcribed if regulated via an operon [27]. The poly(A) tail is critical for the library preparation for ONT sequencing thus, we have established a methodology for adding this component onto transcripts.

In this study, we applied MinION sequencing to interrogate both the genome and the transcriptome (via direct RNA sequencing) for XDR *K. pneumoniae* clinical isolates. Of interest was to compare the potential for RNA sequencing to provide a better correlation to the resistance phenotype than DNA sequencing. These isolates have previously undergone ‘traditional’ whole genome sequencing (Illumina) and antimicrobial susceptibility testing [28]. An extended panel of antibiotics was tested in this study to identify PDR isolates. Three strains were selected from this cohort which exhibited resistance to all 24 antibiotics or antibiotic combinations tested, a high abundance of antibiotic resistance genes (≥26) and differing lineages (ST11 (16_GR_13), ST147 (1_GR_13) and ST258 (2_GR_12)). Additionally, these isolates harbour polymyxin resistance which is facilitated by a disruption in or upstream of *mgrB*. Variations in the *mgrB* gene result in increased expression of the *pmrCAB* and *pmrHFIJKLM* operon, enables the addition of phosphoethanolamine and/ or 4-amino-4-deoxy-L-arabinose (Ara4N) to lipid A and subsequently facilitates polymyxin resistance [29]. These pathways associated with polymyxin resistance were further explored using direct RNA sequencing and compared against a polymyxin-susceptible XDR isolate (ST258; 20_GR_12). This research aimed to assemble these genomes, discern expression of resistance genes and ascertain the time required for detection. Furthermore, we sought to compare DNA and RNA sequencing as modalities for the rapid identification of acquired antibiotic resistance.

Methods

Bacterial strains and growth conditions

XDR *K. pneumoniae* clinical strains were sourced through the Hygeia General Hospital, Athens, Greece [28]. Antimicrobial susceptibility assays (Supplementary Table S1), sequence typing and detection of acquired resistance genes have previously been determined [28]. Strains were stored at -80°C in 20% (v/v) glycerol, the identical stock was used as per the prior study and the extended panel of antimicrobial susceptibility testing conducted similarly [28]. When required for extractions, glycerol stocks were grown on lysogeny broth (LB) agar and 6 morphologically similar colonies were selected for inoculation. The inoculum was grown in LB overnight at 37°C shaking at 220 rpm. This overnight inoculum was used for both DNA and RNA extractions.

High molecular weight DNA isolation

DNA was extracted from 10 ml of overnight culture using the DNeasy Blood and Tissue Kit (Qiagen) according to manufacturer's guidelines, with the addition of an enzymatic lysis buffer pre-treatment (60 mg/ml lysozyme). Following the DNeasy extraction, high molecular weight (HMW) DNA was isolated using the MagAttract HMW DNA Kit (Qiagen) as per manufacturer's instructions. An additional proteinase K treatment at 56°C for 10 min followed by supplementation of RNase A (1 mg) for 15 min at room temperature was included to increase DNA purity. Several direct extractions from bacterial overnight cultures using the HMW kit were performed, however, low DNA yield was observed and the initial DNeasy extraction was essential. An additional purification step following the HMW DNA extraction was critical for 2_GR_12 as carbohydrate contamination (260/230 ratio: ≤0.3) was identified potentially due to a thickened capsule. This

purification included the Monarch® PCR & DNA Cleanup Kit (New England BioLabs) using the protocol to isolate fragments >2000 bp.

RNA extraction, mRNA enrichment and poly(A) addition

The overnight inoculum was sub-cultured in 10 ml of cation-adjusted Muller Hinton Broth (caMHB) to reflect the media used for minimum inhibitory concentration (MIC) assays. Cultures were grown to mid-log phase (OD_{600} = 0.5-0.6). RNA was extracted via the PureLink™ RNA Mini Kit (Thermo Fisher Scientific) as per manufacturer's protocols which included using Homogenizer columns (Thermo Fisher Scientific). To remove DNA contamination, the TURBO DNA-free™ kit was implemented. A minor adjustment was an increased concentration of TURBO DNase (4 U) incubated at 37°C for 30 min. The RNeasy Mini Kit (Qiagen) clean up protocol was used to purify and concentrate RNA samples. Ribosomal RNA was depleted via the MICROB*Express*™ Bacterial mRNA Enrichment Kit (Thermo Fisher Scientific). Minor protocol changes included adding ≥2 µg of DNA depleted RNA and the enriched mRNA was precipitated for 3 h at -20°C. Poly(A) addition was performed using the Poly(A) Polymerase Tailing Kit (Astral Scientific) as per the manufacturer's alternative protocol (4 U input of Poly(A) Polymerase). The input RNA concentration was ≥800 ng and RNA samples were incubated at 37°C for 1 h. Poly(A) ligated RNA was purified using Agencourt AmpureXP (Beckman Coulter Australia) beads (1:1 ratio).

Extraction quality control

DNA and RNA were quantitated using Qubit®2.0 (Thermo Fisher Scientific) and purity determined with a NanoDrop 1000 Spectrophotometer (Thermo Fisher Scientific). DNA fragment sizes were measured using the Genomic DNA ScreenTape & Reagents (Agilent) and sizes from 200 to >60000 bp were analyzed on a 4200 TapeStation System (Agilent) (Supplementary Figure

S1). RNA fragment size was checked using an Agilent RNA 6000 Pico kit and run on a 2100 Bioanalyzer (Agilent Technologies) for the initial RNA extract (RIN: ≥8.5), post ribosomal RNA depletion and after poly(A) tailing (Supplementary Figure S2).

ONT library preparation and sequencing

RNA libraries (≥600 ng poly(A)$^+$ RNA) were prepared using the Direct RNA Sequencing kit (SQK-RNA001). The Rapid Barcoding Sequencing kit (SQK-RBK001) was used for HMW DNA samples (1_GR_13, 16_GR_13, 20_GR_12; 300 ng input each). Isolate 2_GR_12 (300 ng input) was prepared separately using the Rapid Sequencing Kit (SQK-RAD003). Libraries were sequenced with MinION R9.4 flowcells and the raw data (fast5 files) were base-called using Albacore 2.1.1 for DNA sequencing (Supplementary Figure S3). For benchmarking purposes, RNA reads were additionally base-called with Albacore 2.2.7, Guppy 3.0.3 and the Chiron v0.5 [30] RNA base-caller which was trained in-house (https://github.com/haotianteng/Chiron/releases/tag/v0.5).

Real-time resistome detection emulation

The real-time emulation was performed post sequencing and the time required to detect antibiotic resistance was determined as previously described [14]. Briefly, this pipeline aligns Albacore base-called reads via BWA-MEM (BWA , RRID:SCR_010910)[31] to an antibiotic resistance gene database. Antibiotic resistance genes were obtained from the ResFinder 3.0 database [32]. This dataset comprises of 2131 genes which were clustered based on 90% identity to form 611 groups or gene families. The detection of false positives is reduced using the multiple sequence alignment software kalign2 [33], a probabilistic Finite State Machine [34] and once the alignment score reached a threshold, the resistance gene was reported.

Assembly of genomes

To assemble genomes with both Illumina and ONT reads, SPAdes v3.10.1 (SPAdes , RRID:SCR_000131)[35] was utilised. Hybrid assemblers included npScarf [36] and Unicycler v0.3.1 [37]. Assemblers using only ONT reads included Canu v1.5 (excluding reads <500bp) (Canu, RRID:SCR_015880)[38] and the combination of Minimap2 v2.1-r311 and Miniasm v0.2-r168-dirty; Racon (git commit 834442) were used in both cases to polish the assemblies [39, 40]. Consensus sequences were determined using Mauve (snapshot_2015-02-13) to construct the final assembly (Mauve, RRID:SCR_012852)[41]. The output from each assembly software is reported in Supplementary Table S2. Genomes were annotated using the Rapid Annotation using Subsystem Technology (RAST) which also provided a list of virulence genes [42]. The location of acquired antibiotic resistance genes were determined using ResFinder 3.0 [32] and plasmids were identified via PlasmidFinder 1.3 [43]. To discern if plasmid sequences have previously been reported, contigs underwent a BLASTn analysis against the National Center for Biotechnology Information (NCBI) database (https://blast.ncbi.nlm.nih.gov/Blast.cgi)(BLASTN, RRID:SCR_001598).

RNA alignment and expression profiling

Base-called RNA reads were converted to DNA (uracil bases changed to thymine) and aligned using BWA-MEM [31] to the updated genome assemblies. BWA-MEM was selected due to shorter transcripts being produced by bacteria (Supplementary Figure S3) and the lack of introns and alternative splicing. Similar parameters to the BWA-MEM ont2d function were used but seed length was reduced (-k 14) to compensate for shorter reads: -k 11 [minimum seed length, bp] -W20 [bandwidth] -r10 [gap extension penalty] -A1 [match score] -B1 [mismatch penalty] -O1 [Gap open penalty] -E1 [Gap extension penalty] -L0 [Clipping penalty]). Multi-mapping reads were removed via SAMtools (secondary alignment: flagged as 256) [44] and BEDTools coverage

(BEDTools , RRID:SCR_006646)[45] was used to ascertain the expression of resistance genes in counts per million (cpm) mapped reads (post removing reads mapping to rRNA). BEDTools intersect [45] was utilised to identify potential operons and co-expression of genes. To compare against qRT-PCR results, read counts were normalised the housekeeping gene, *rpsL* [46]. Read alignments were further visualised using Integrative genomics viewer 2.3.59 [47].

Whole transcriptome gene expression and estimation of expression confidence intervals

We identified genes which were differentially expressed in one sample (versus all remaining samples) using a quasi-likelihood F-test in EdgeR (edgeR, RRID:SCR_012802)[48] with a FDR threshold of 0.01. Expression levels (in cpm) were extracted for every significant gene in any one of these one versus remaining differential expression analyses in order to generate an expression heatmap. The expression heatmap is based on the log10(cpm) for each of these genes. In order to estimate the 90% confidence intervals in cpm estimates from direct RNA sequence data, we assumed that the observed counts were generated from a binomial distribution with unobserved probability of success (p). We estimate the 5% and 95% percentiles from a beta-distribution with shape parameters equal to the number of reads mapped to a given gene (alpha) and the number of reads mapped elsewhere (beta) plus a pseudo-count of 0.1. The 90% confidence interval (CI) is calculated as the difference between the expression levels at the 5% and 95% percentile.

Quantitative real-time reverse transcriptase PCR (qRT-PCR)

First strand cDNA synthesis was performed on 1 µg of total DNA-depleted RNA using SuperScript III (Thermo Fisher Scientific). Primers used are displayed in Supplementary Table S3. Samples were prepared in triplicate via the SYBR Select Master Mix (Thermo Fisher Scientific) and expression detected using a ViiA 7 Real-time PCR system (Thermo Fisher Scientific). Cycling conditions include: Hold 50°C (2 min), 95°C (2 min) followed by 50 cycles of: 95°C (15 sec),

55°C (1 min). A melt curve was included to determine the specificity of the amplification and a no template control to detect contamination or primer dimers. Results were analysed with QuantStudioTM Real-Time PCR Software, triplicates were averaged, normalised to the housekeeping gene *rpsL* [46] and relative expression determined via the $2^{-\Delta\Delta CT}$ method [49].

Results

Antibiotic resistance and the location of acquired resistance in the genome

This study assayed nine additional antibiotics or antibiotic combinations to further characterise the phenotypic resistance of these isolates (Supplementary Table S1). Strains 1_GR_13, 2_GR_12 and 16_GR_13 were non-susceptible to all antibiotics including the 24 antibiotics tested previously [28]. 20_GR_12 was only susceptible to gentamicin and polymyxins.

MinION DNA sequencing for all isolates was run for ≥20 hours which generated 1.19 GB (215X) for 1_GR_13, 0.39 GB (67X) for 2_GR_12, 0.56 GB (101X) for 16_GR_13 and 0.64 GB (115X) for 20_GR_12 (Supplementary Table S2). Across the differing assembly tools, the chromosome sequence commonly circularised as a 5.0-5.4 Mb contig including plasmids ranging between 13-193 kb with the exception of 2_GR_12. Aligning ONT reads to the final assembly revealed that DNA sequencing had 90% accuracy across isolates.

Utilising the capacity for MinION sequencing to read long fragments of DNA, the location of antibiotic resistance genes were clearly resolved (Table 1). All genomes were circular except for 2_GR_12 where 3 plasmids remained linear. This was partly due to difficulties extracting DNA, not retaining long fragments and subsequently, lower coverage of the genome (Supplementary Figure S1, Table S2). Amongst the four isolates, the chromosome size ranged between 5.1-5.5 Mb

which encoded resistance genes *blaSHV-11*, *fosA* and *oqxAB*. The majority of resistance (≥75%) mapped to plasmids.

At least one megaplasmid, defined as a plasmid larger than 100 kbp, was detected in all isolates (Table 1). These commonly harboured the replicon IncA/C2 or InFIB and IncFIIK. The IncA/C2 plasmid was present in all samples except 20_GR_12. This plasmid contained up to 16 resistance genes which conferred resistance towards aminoglycosides, β-lactams, phenicols, rifampicin, sulphonamides, tetracyclines and trimethoprim, with the exception of 16_GR_13. Isolate 16_GR_13 lacked trimethoprim resistance on its IncA/C2 plasmid. The plasmids containing both replicons IncFIB and IncFIIK differed vastly between all four replicates. All contained IncFIB$_{pKpn3}$ and IncFIIK, however, 1_GR_13 differed with IncFII$_{pKP91}$. Additionally, a differing IncFIB replicon was detected on a separate contig in 1_GR_13 (pKPHS1) and 2_GR_12 (pQil). The only instance where another dual replicon was identified was in 1_GR_13 which harboured both IncR and IncN. This plasmid contained aminoglycoside, β-lactam, trimethoprim, macrolide and sulphonamide resistance. 1_GR_13 also contained a 5.5 kb circular contig which was annotated as a phage genome. Various regions of these megaplasmids were unique to these isolates compared to prior sequences deposited on NCBI (Supplementary Table S5).

The ColRNAI plasmid was present in all except 1_GR_13 which encoded aminoglycoside and quinolone resistance (*aac(6')-Ib, aac(6')-Ib-cr)* (Table 1). The ColRNAI plasmid in 2_GR_12 and 20_GR_12 was 13841 bp in size and shared 75% similarity between the two isolates. This plasmid differed in 16_GR_13 which contained no resistance genes and 35% the size. The same IncX3 plasmid (43380 bp) was apparent in isolates 2_GR_12 and 20_GR_12. Unique to 16_GR_13 was the IncL/ M$_{pOXA-48}$ plasmid containing *blaOXA-48* and the 50979 bp IncN plasmid in 20_GR_12

with resistance against 5 classes (aminoglycoside (*aph(3'')-Ib*, *aph(6)-Id*), β-lactam (*blaTEM-1A*), sulphonamide (*sul2*), tetracycline (*tet(A)*), trimethoprim (*dfrA14*)) of antibiotics.

Multiple copies of acquired resistance genes were apparent across plasmids in several isolates. For 1_GR_13, up to three copies were present of genes *aadA24, aph(3')-Ia, aph(6)-Id, dfrA1, dfrA14, strA* and *sul1* (Table 1). In 2_GR_12, *sul1* and *blaTEM-1A* were duplicated and for 16_GR_13, only *sul1* was represented twice.

Real-time detection emulation of resistance genes via DNA sequencing

The vast majority (≥70%) of resistance genes were detected via DNA sequencing within the first 2 hours (Figure 1, Supplementary Table S5). These genes confer resistance towards aminoglycosides, β-lactams, fosfomycin, macrolides, phenicols, quinolones, rifampicin, sulphonamides, tetracyclines and trimethoprim. 20_GR_12 lacked acquired resistance genes for macrolides, phenicols and rifampicin, however, all other classes were detected within 2 hours. All isolates, except 2_GR_12, were sequenced for 21 hours which was sufficient to obtain the complete genome assembly. Only a few additional genes were detected after the first 10 hours across isolates (Supplementary Table S5). For 2_GR_12, an extended run of 41 hours detected no further genes after 20 hours. Overall, the presence of these resistance genes corresponded to a resistant phenotype towards aminoglycosides, β-lactams, fosfomycin, phenicols, quinolones, sulphonamides (sulfamethoxazole), tetracyclines and trimethoprim (Supplementary Table S1). As macrolides and rifampicin are not routinely used to treat *K. pneumoniae* infections, no breakpoints exist according to CLSI and EUCAST guidelines, however, all isolates exhibit an MIC ≥128 μg/ml towards erythromycin (macrolide) and ≥64 μg/ml for rifampicin (Supplementary Table S1).

Post 2 hours of sequencing, several genes not observed in the final assembly via ResFinder 3.0 were detected (Supplementary Table S5). These were predominantly genes attributed to

aminoglycoside, β-lactam, rifampicin and phenicol resistance. Furthermore, resistance genes to additional classes were detected including fusidic acid and vancomycin. This was evident in 2_GR_12 (*fusB*) and 16_GR_13 (*fusB*, *vanR*). However, these genes had less than 30 reads and their phred-scale mapping quality (MAPQ) scores were less than 10 (misplaced probability greater than 0.1). Furthermore, the majority of genes not observed in the final assembly nor observed in Illumina data exhibited a MAPQ score of ≤10 which may indicate that a more stringent threshold is required to negate false positives. However, if this threshold increases, true positives would not be detected including *aadA1*, *aadA2* and *ARR-2* in 2_GR_12 and *blaOXA-48*, *blaCTX-M-15* and *ARR-2* in 16_GR_13.

Several genes found in the final assembly were not detected in the real-time emulation analysis (Supplementary Table S5). This was mainly observed for aminoglycoside resistance encoding genes. For 1_GR_13, this included *aadA1*, *ant(2")-Ia*, *aph(6)-Id* and *aadA24*. Similarly, 2_GR_12 and 20_GR_12 lacked *aph(3")-Ib* and *aph(6)-Id*. 2_GR_12 additionally had the absence of *ant(2")-Ia*. Detection of *ant(2")-Ia*, *aph(3")-Ib*, *aph(6)-Id* was not present in 16_GR_13. 16_GR_13 further lacked *catB4* (phenicol) and *tet(A)* (tetracycline). Various phenicol resistance genes were reported in the real-time emulation however, the incorrect gene was identified which may represent sequencing errors accumulated over time and high similarity to other phenicol resistance genes. The tetracycline resistance gene, *tet(A)*, was interestingly not reported in this emulation with 190 reads and the majority of reads exhibiting a high mapping confidence (MAPQ = 60, equivalent to an error probability of $1x10^{-6}$). This gene was only detected after 10 hours for 1_GR_13 and 2_GR_12 and this result may be influenced by the presence of only 1 copy of *tet(A)* encoded on a low copy number megaplasmid (between 1 to 1.5, see Table 1).

Direct RNA sequencing resistance detection

The time required to detect resistance was further interrogated using RNA sequencing. Rapid detection was possible for several resistance genes via direct RNA sequencing (Figure 1). This was evident for genes conferring resistance to aminoglycosides, β-lactams, sulphonamides and trimethoprim for all four isolates. Resistance towards these antibiotics was commonly detected within 6 hours. In some instances, quinolone, rifampicin, fosfomycin and phenicol resistance was detected. A similar result was obtained whether all reads or passed reads alone were analysed. The most significant difference when analysing all reads was the detection of *fosA* in 1_GR_13 and *ARR-2* and *fosA* in 2_GR_12. Consistently absent from this analysis were genes attributed to macrolide (*mph(A)*) and tetracycline (*tet(A)*, *tet(G)*) resistance, however, isolates exhibited high levels of resistance to tetracycline (>64 µg/ml) (Supplementary Table S1). Commonly no new genes were detected after 12 hours of sequencing except for *fosA* in 2_GR_12. Although *fosA* was detected when including the failed reads, a low MAPQ score (≤10) was apparent. Similar to the DNA real-time detection, several genes not found in the final assembly were identified (Supplementary Table S5). With the exception of 20_GR_12, this included *aadB* and *strB* for all isolates. Additional genes detected included *ARR-7* in 1_GR_13, *strA* in 2_GR_12 and for 16_GR_13, *blaCTX-M-64*, *blaOXA-436* and *strA.* Similar genes or gene families were identified when comparing DNA and direct RNA sequencing. Overall, genes were detected more readily via DNA sequencing however, there were a few instances where RNA sequencing detected resistance quicker: *aac(3')-IIa* in 16_GR_13 and *sul2* and *catA1* in 2_GR_12. Similar results were observed when investigating data yield rather than time which compensates for the slower translocation speed associated with direct RNA sequencing (Supplementary Figure S4).

Levels of expression of resistance genes

RNA sequencing accumulated over approximately 40 hours yielded between 0.9 and 1.7 million reads for these isolates (Supplementary Figure S3). However, only a low proportion (972436 to 1725702 reads: ≤14.64%) of these reads passed base-calling using Albacore 2.2.7 (Supplementary Table S6). Aligning passed reads alone to the final assembly (ensuring the removal of the poly(A) tail and reads <75 nt), ≥98% (1_GR_13: 95591; 2_GR_12: 138214; 16_GR_13: 227781; 20_GR_12: 119425) of reads were mappable, however, ≤46% (1_GR_13: 42654; 2_GR_12: 46787; 16_GR_13: 79175; 20_GR_12: 54986) of these had a MAPQ score ≥10. When failed reads were aligned, the majority were not mappable to the reference genome (≥0.76 million reads, ≥91.50%) and commonly exhibited a low MAPQ score (≤10). Low mapping quality could be attributed to assignment of reads to multiple copies of genes in the genome. Furthermore, the ONT error rates could lead to misassignment of reads to genes. In light of this, we decided to benchmark a number of different base-callers, including Albacore 2.2.7, Guppy 3.03 as well as Chiron v0.5 which was trained in-house (Supplementary Table S6, Figure S5). Chiron base-called more reads compared to Albacore 2.2.7 and Guppy 3.0.3, however, fewer reads aligned to the reference genome and had a slightly lower identity rate. Albacore 2.2.7 had the highest average accuracy across isolates (84.87%) closely followed by Guppy 3.0.3 (84.62%) and then Chiron v0.5 (78.19%) (Supplementary Table S6). However, low alignment rates could be attributed to the addition of a long artificial poly(A) which was identified to be approximately 400 to 700 bp across isolates (Supplementary Figure S6). Taking into consideration the Albacore 2.2.7 base-called reads, a proportion of these reads were found to map to rRNA including 1_GR_13 (18%), 2_GR_12 (37%), 16_GR_13 (24%) and 20_GR_12 (23%). Overall, at least 58% of genes (with at least 1 read mapping to the gene) were identified to be expressed across isolates (1_GR_13 (68%), 2_GR_12 (58%), 16_GR_13 (75%) and 20_GR_12 (69%).

Amongst the four isolates, levels of expression for resistance genes on the chromosome (*blaSHV-11, fosA* and *oqxAB*) were low (≤122 counts per million mapped reads) (Figure 2). The remaining resistance genes were located on plasmids. Resistance genes exhibiting high levels of expression (300 cpm) were apparent in 1_GR_13 (*blaTEM-1B, blaVIM-27, sul1, aph(3')-Ia*), 2_GR_12 (*aac(6')-Ib, catA1, blaKPC-2*), 16_GR_13 (*aac(6')Ib-cr, aac(3)-IIa, blaCTX-M-15, blaTEM-1B, blaOXA-48*) and 20_GR_12 (*blaKPC-2*, *aac(6')Ib*). Counts for *aac(6')-1b* and *aac(6')-1b-cr* in 2_GR_12 and 20_GR_12 were grouped. The gene *aac(6')-1b-cr* is a shortened version of *aac(6')-1b* and both were identified in the same genome position, hence, only *aac(6')-1b* is displayed in Figure 2. Expression estimates did not differ significantly when analysing passed reads alone or all reads. We estimated the 90% confidence interval in cpm estimates using a beta-distribution (Supplementary Figure S7). All highly expressed genes were detected within 6 hours as per the real-time detection emulation. As anticipated, low levels of expression were observed for fosfomycin (*fosA*), tetracycline (*tet(A)*, *tet(B)*) and macrolide (*mph(A)*) resistance. Several resistance genes were identified to be regulated by operons and co-expression was evident for *oqxAB* (1_GR_13, 16_GR_13), *blaVEB-1*:*ant(2")-Ia*:*ARR-2* (1_GR_13), *aadA1*:*sul1* (1_GR_13), *rmtB*:*blaTEM-1B* (1_GR_13, 2_GR_12, 16_GR_13), *aph(6)-Id*:*strA* (1_GR_13), *sul2*:*aph(3")-Ib*:*aph(6)-Id* (2_GR_12, 16_GR_13), *ant(2")-Ia*:*blaVEB-1* (2_GR_12, 16_GR_13), *aac(6')-Ib-cr*:*blaOXA-1*:*catB4* (16_GR_13), *aadA2*:*sul1* (16_GR_13) and *sul2*:*aph(3")-Ib*:*dfrA14* (20_GR_12) (Figure 2). Overall, various non rRNA genes were identified to be co-expressed (≥5 reads supporting gene intersect) across isolates (1_GR_13: 428; 2_GR_12: 310; 16_GR_13: 793; 20_GR_12: 442).

A subset of 11 resistance genes which represent resistance across various classes of antibiotics were investigated to validate gene expression in these RNA extractions via qRT-PCR (Figure 3).

These included resistance towards aminoglycosides (*aac(6')Ib*, *strA*), β-lactams (*blaKPC-2*, *blaOXA-10*, *blaTEM-1*), phenicols (*cmlA1*), trimethoprim (*dfrA14*), fosfomycin (*fosA*), quinolone (*oqxA*), sulphonamides (*sul2*) and tetracyclines (*tet(A)*). A similar trend was observed between direct RNA sequencing and qRT-PCR results (Spearman's rank correlation coefficient: 0.83; Pearson correlation: 0.86) (Figure 3). The highest expression of a resistance gene was observed for *blaKPC-2* although only one copy was present in a lower copy number plasmid in 2_GR_12 and 20_GR_12 (Figure 2, Figure 3 and Table 1). Additionally, low levels of expression for *fosA* and *tet(A)* were apparent despite exhibiting resistance towards fosfomycin and tetracycline (Figure 3, Supplementary Table S1). Direct RNA sequencing was unable to detect low levels of expression whilst qRT-PCR could detect these genes (Figure 3).

Across the transcriptome, antibiotic resistance genes were identified to harbour high expression between isolates (Figure 4). Virulence genes were comparable across these strains similar to all remaining or background genes. The top differentially expressed genes were determined (Supplementary Figure S8) and several were associated with polymyxin resistance pathways. Heightened expression was seen in polymyxin-resistant isolates 1_GR_13, 2_GR_12, 16_GR_13 in comparison to the single susceptible isolate (20_GR_12) in particular, genes associated with Ara4N synthesis. These genes include 4-deoxy-4-formamido-L-arabinose-phosphoundecaprenol deformylase (*arnD*), UDP-4-amino-4-deoxy-L-arabinose formyltransferase and UDP-4-amino-4-deoxy-L-arabinose-oxoglutarate aminotransferase.

Transcriptional biomarkers for polymyxin resistance

Three of the isolates harboured resistance towards polymyxins via disruptions in *mgrB* which included 1_GR_13, 2_GR_12 and 16_GR_13. 1_GR_13. Isolate 1_GR_13 has an insertion sequence (IS) element, IS*Kpn26*-like, at nucleotide position 75 in the same orientation as *mgrB*

whilst 2_GR_12 has this IS element in the opposite orientation plus additional mutations in *phoP* (A95S) and *phoQ* (N253T). 16_GR_13 harbours an IS element, IS*1R*-like, 19 bp upstream of *mgrB*. Direct RNA sequencing revealed only low-level expression of *mgrB* (1_GR_13 (78.4 cpm), 2_GR_12 (16.3 cpm), 16_GR_13 (0 cpm), 20_GR_12 (2.3 cpm)). The expression levels of various genes associated with this pathway were verified via qRT-PCR (Figure 5). Direct RNA sequencing revealed a slight increase in transcription of *phoPQ* (≥2-fold) relative to 20_GR_12. A ≥13-fold increase in expression was observed for *pmrH* and ≥8-fold elevation for *pmrK*. Similar trends for expression were also reported using qRT-PCR (Figure 5).

Discussion

Extensively drug-resistant *K. pneumoniae* infections pose as a major threat to modern medicine. A rapid diagnostic would help to guide appropriate treatment options [1, 6]. The MinION sequencing technology employed in this study has potential to detect antibiotic resistance in a timely manner. Three of the four *K. pneumoniae* isolates examined in this study harboured non-susceptibility to all antibiotics or antibiotic combinations assayed, and hence would be classified as PDR according to published guidelines [50]. ONT sequencing was able to resolve both the assembly of plasmids harbouring high levels of resistance (through DNA sequencing) and the expression from the resistome in the absence of antibiotic treatment (via RNA sequencing).

The ability for ONT to sequence long fragments of DNA has significantly aided the assembly of bacterial genomes and plasmids [16-18]. In this study, multiple megaplasmids (≥100 kbp) were identified which were previously unresolved via Illumina sequencing [28]. These harboured replicons IncA/C2 or a dual replicon, IncFIIK and IncFIB. The IncA/C, IncF and IncN plasmids have been commonly associated with multidrug resistance [51]. Although several plasmids in this

study revealed similarity to previously reported isolates via NCBI, various sequences deviated. In particular, the IncA/C2 plasmid exhibited multiple regions unique to these isolates. Several IncA/C2 megaplasmids have been previously described which harbour various resistance genes. However, the extent of resistance observed in our study is extreme when compared to prior reports [52, 53]. Prior studies have shown the IncFIIK and IncFIB replicons to localise on the same plasmid and also megaplasmids with multidrug resistance [6]. The $IncFIB_{pQil}$ plasmid in this study contained various β-lactam resistance genes (*blaKPC-2*, *blaOXA-9*, *blaTEM-1A*) which has been identified previously [54]. Similarly, *blaOXA-48* segregated with the IncL/M replicon [55,56], however, deviations in this plasmid were identified.

The real-time analysis capability entailed in MinION sequencing has the potential to rapidly determine antibiotic resistance profiles of pathogenic bacteria. Previously this device has been utilised to assemble bacterial genomes, discern species and detect antibiotic resistance [12-15]. This study investigated the potential time required to discern resistance via a real-time emulation as previously described [14]. The majority (≥70%) of resistance genes were detected via DNA sequencing within 2 hours. Several genes not identified in the final assembly were detected after 2 hours of sequencing. This may be attributed to the high similarity (≥80%) amongst various genes, in particular, those associated with aminoglycoside, β-lactam, rifampicin and phenicol resistance. Furthermore, the error rate associated with ONT sequencing, and the accumulation of these errors over time, may result in the false annotation of these genes. Nanopore DNA sequencing currently has an accuracy ranging from 85 to 95% (90% in our study), which limits its ability to detect genomic variations [17, 57]. Several resistance genes only differ by a few nucleotides which significantly impacts the resistance phenotype and the antibiotics which can be utilised to treat these infections. However, software tools such as Nanopolish (https://github.com/jts/nanopolish)

and Tombo (https://github.com/nanoporetech/tombo) (similarly used to re-train Chiron v0.5 for direct RNA sequencing data) have the potential to correct these reads and would be helpful to integrate to increase the accuracy of detecting resistance genes. We utilised native DNA sequencing in this study which retains epigenetic modifications such methylation which can hinder the accuracy of reads and subsequent calling of antibiotic resistance [58]. Furthermore, a small number of resistance genes were identified that were not present in the final assembly, however these all had MAPQ values less than 10 and less than 30 mapped reads. This may be due to low-level kit contamination, while some of the false positives have sequence similarity to true positives and may be due to inaccuracies in base-calling.

We further investigated the transcriptome of these isolates to potentially elucidate the correlation between genotype and the subsequent resistant phenotype. Detection of antibiotic resistance via sequencing commonly uses DNA due to the instability of RNA and the lengthy sample processing such as rRNA depletion [12-15, 58]. However, RNA provides additional information regarding the functionality of genes such as identifying conditions in which a resistance gene is present but not active which gives rise to a false positive via DNA alone. Conversely, if expression is only induced in the presence of an antibiotic, the absence of RNA transcripts results in a false negative. This study grew *K. pneumoniae* strains in the absence of antibiotic and direct RNA sequencing revealed high levels of transcription from genes associated with aminoglycoside, β-lactam, sulphonamide and trimethoprim resistance within 6 hours of our study. In particular, the highest levels of expression were observed for the β-lactamase gene *blaKPC-2* in 2_GR_12 and 20_GR_12. Alterations in the promoter region have previously been reported to influence high levels of expression [59]. Notably, the promoter or operon (co-transcribed genes) can largely influence expression of genes with several resistance genes potentially identified to be regulated

by operons in this study. The detection of quinolone, rifampicin, and phenicol resistance correlated to the levels of transcription within samples. All isolates exhibited low levels of expression for fosfomycin, macrolide and tetracycline resistance, despite exhibiting phenotypic resistance to fosfomycin and tetracycline [28]. FosA, an enzyme involved in fosfomycin degradation, is commonly encoded chromosomally in *K. pneumoniae* and a combination of expression and enzymatic activity contributes to resistance [60]. Noteably, Klontz *et al* identified that chromosomally integrated FosA, similarly observed in our study, from *K. pneumoniae* harboured a higher catalytic efficiency. A higher catalytic efficiency may reason why our strains only require a low abundance of expression and still retain fosfomycin resistance. Genes *tet(A)* and *tet(G)* encode efflux pumps which, in the absence of tetracycline, are lowly expressed and the lack of antibiotic supplementation in this study confirms this observation [61]. Detecting inducible resistance (antibiotic exposure required for gene expression) such as tetracycline resistance highlights one of the advantages of investigating the transcriptome.

There are several other variables to consider when interpreting expression levels in bacterial RNA sequencing data. These include the extent prior exposure to antibiotics in the clinic alters transcription and the copy number of resistance genes and the plasmids these are encoded on. Limitations were observed when base-calling bacterial direct RNA sequencing and may be attributed to trimming the long artificial poly(A) tail and interference of RNA modifications. This entailed an increased error rate of $\leq$23% across base-callers (12% identified in a prior study [21]) and a poor alignment rate $\leq$23%. Furthermore, the time required to detect resistance may be hindered by the slower translocation speed associated with direct RNA sequencing (70 bases/ second) compared to DNA sequencing (450 bases/ second) [57]. Our findings show that the slower time-to-detection of resistance genes in direct RNA sequencing was due to both the level of

expression as well as the slower translocation speed, and hence using cDNA would only partially overcome this limitation.

We also investigated pathways attributed to polymyxin resistance. Three of these strains exhibited an IS element upstream of within *mgrB*, the negative regulator of PhoPQ [29]. Elevated expression was apparent for *phoPQ* and also the *pmrHFIJKLM* operon in our polymyxin-resistant isolates harbouring a disruption in *mgrB*. This has previously been witnessed for other *K. pneumoniae* isolates harbouring *mgrB* disruptions and is a potential transcriptional marker for polymyxin resistance [29, 46, 62, 63]. However, this study is limited to four isolates and one mechanism associated with polymyxin resistance. Other pathways have previously been identified including the role of other two component regulatory systems such as CrrAB [64]. The ability to use relative expression of key genes to detect polymyxin resistance requires further validation, including an increased sample size of resistant and non-resistant isolates. Furthermore, additional functional experiments such as complementation assays would be required in order to validate the contribution of a certain mutation to the transcriptome and subsequent resistance.

Conclusions

This study has utilised MinION sequencing to assemble four XDR *K. pneumoniae* genomes and has revealed several unique plasmids harbouring multidrug resistance. The vast majority of this resistance was detectable within 2 hours of sequencing. Exploiting this analysis in real-time would allow for a rapid diagnostic, however, the presence of a resistance gene does not necessarily indicate resistance is conferred and requires additional phenotypic characterisation. This research also established a methodology and analysis for bacterial direct RNA sequencing. The expression of resistance genes was successfully detected in addition to identifying genes potentially regulated via operons, however, native RNA sequencing incurs a slower time to detect resistance due to

translocation speed. Once base-calling algorithms have been optimised, this could allow for a whole transcriptome interrogation of the poorly characterised bacterial RNA modifications. Overall, this study has begun to unravel the association between genotype, transcription and subsequent resistant phenotype in these XDR/ PDR *K. pneumoniae* clinical isolates, establishing the groundwork for developing a diagnostic that can rapidly determine bacterial resistance profiles.

Availability of supporting data

The datasets supporting the results presented here are available in the NCBI repository BioProject PRJNA307517 (www.ncbi.nlm.nih.gov/bioproject/PRJNA307517). ONT DNA sequencing data has been deposited on the Sequence Read Archive (www.ncbi.nlm.nih.gov/sra/) under study SRP133040. Accession numbers are as follows: 1_GR_13 (SRR6747887), 2_GR_12 (SRR6747886), 16_GR_13 (SRR6747885) and 20_GR_12 (SRR6747884). ONT direct RNA sequencing data (pass and fail reads) have been deposited on the Sequence Read Archive (www.ncbi.nlm.nih.gov/sra/) under study SRP133040. Accession numbers are as follows: 1_GR_13 (SRR7719054), 2_GR_12 (SRR7719055), 16_GR_13 (SRR7719052) and 20_GR_12 (SRR7719053). Alignments, assemblies and other supporting data are also available from the *GigaScience* GigaDB repository [65].

Abbreviations

Ara4N: 4-amino-4-deoxy-L-arabinose; caMHB: cation-adjusted Muller Hinton Broth; CLSI: Clinical & Laboratory Standards Institute; CI: Confidence interval; cpm: counts per million; EUCAST: The European Committee on Antimicrobial Susceptibility Testing; FDR: False discovery rate; HMW: High molecular weight; IS: Insertion sequence; LB: Lysogeny broth; MAPQ: Mapping quality; MIC: Minimum inhibitory concentration; NCBI: National Center for

Biotechnology Information; ONT: Oxford Nanopore Technologies; PDR: Pandrug-resistant; RAST: Rapid Annotation using Subsystem Technology; rRNA: Ribosomal RNA; XDR: Extensively drug-resistant.

Competing Interests

The authors declare that there are no competing interests.

Funding

LJMC is an NHMRC career development Fellow APP1103384. MAC is an NHMRC Principal Research Fellow (APP1059354) and currently holds a fractional Professorial Research Fellow appointment at the University of Queensland with his remaining time as CEO of Inflazome Ltd. a company headquartered in Dublin, Ireland that is developing drugs to address clinical unmet needs in inflammatory disease by targeting the inflammasome. MEP is an Australian Postgraduate Award scholar. MATB is supported in part by a Wellcome Trust Strategic Award 104797/Z/14/Z. This work was supported by the Institute for Molecular Bioscience Centre for Superbug Solutions (610246).

Author Contributions

MEP, LJMC, MATB and MAC conceived this study. MEP, SHN and HT performed the sequencing analysis. Laboratory work was carried out by MEP and TPSD. MEP wrote the paper with input from all authors.

Acknowledgements

We would like to acknowledge Dr Ilias Karaiskos and Dr Helen Giamarellou for providing the bacterial strains in this study. We also acknowledge Dr Evangelos Bellos for his guidance on the RNA sequencing analysis and Dr Devika Ganesamoorthy for the initial advice on the direct RNA

sequencing library preparation. We would like to acknowledge Dr Intawat Nookaew for providing yeast direct RNA sequence data and Josh Zhang for his help with the operon detection.

References

1. Martin RM, Bachman MA. Colonization, Infection, and the Accessory Genome of *Klebsiella pneumoniae*. *Front Cell Infect Microbiol.* 2018;8:4.
2. Magill SS, Edwards JR, Bamberg W, et al. Multistate point-prevalence survey of health care-associated infections *N Engl J Med*. 2014;370:1198-208.

3 Kalanuria AA, Ziai W, Mirski, M. Ventilator-associated pneumonia in the ICU. *Crit Care*. 2014;18:208.

4 Talha KA, Hasan Z, Selina F, et al. Organisms associated with ventilator associated pneumonia in intensive care unit. *Mymensingh Med J*. 2009;18:S93-7.

5 Podschun R, Ullmann U. *Klebsiella spp* as nosocomial pathogens: epidemiology, taxonomy, typing methods, and pathogenicity factors. *Clin Microbiol Rev*. 1998;11:589-603.

6 Navon-Venezia S, Kondratyeva K, Carattoli A. *Klebsiella pneumoniae*: a major worldwide source and shuttle for antibiotic resistance. *FEMS Microbiol Rev*. 2017;41:252-75.

7 Karaiskos I, Giamarellou H. Multidrug-resistant and extensively drug-resistant Gram-negative pathogens: current and emerging therapeutic approaches. *Expert Opin Pharmacother*. 2014;15:1351-70.

8 Chen L, Todd R, Kiehlbauch J, et al. Notes from the Field: Pan-Resistant New Delhi Metallo-Beta-Lactamase-Producing *Klebsiella pneumoniae* - Washoe County, Nevada, 2016 *MMWR Morb Mortal Wkly Rep*. 2017;66:33.

9 Zowawi HM, Forde BM, Alfaresi M, et al. Stepwise evolution of pandrug-resistance in *Klebsiella pneumoniae*. *Sci Rep*. 2015;5:15082.

10 Sommer MOA, Munck C, Toft-Kehler RV, et al. Prediction of antibiotic resistance: time for a new preclinical paradigm? *Nat Rev Microbiol*. 2017;15:689-96.

11 Gardy JL, Loman NJ. Towards a genomics-informed, real-time, global pathogen surveillance system. *Nat Rev Genet*. 2018;19:9-20.

12 Lemon JK, Khil PP, Frank KM, et al. Rapid Nanopore Sequencing of Plasmids and Resistance Gene Detection in Clinical Isolates. *J Clin Microbiol*. 2017;55:3530-43.

13 Votintseva AA, Bradley P, Pankhurst L, et al. Same-Day Diagnostic and Surveillance Data for Tuberculosis via Whole-Genome Sequencing of Direct Respiratory Samples. *J Clin Microbiol*. 2017;55:1285-98.

14 Cao MD, Ganesamoorthy D, Elliott AG, et al. Streaming algorithms for identification of pathogens and antibiotic resistance potential from real-time MinION^{TM} sequencing. *Gigascience*. 2016;5:32.

15 Quick J, Ashton P, Calus S, et al. Rapid draft sequencing and real-time nanopore sequencing in a hospital outbreak of *Salmonella*. *Genome Biol*. 2015;16:114.

16 Wick RR, Judd LM, Gorrie CL, et al. Completing bacterial genome assemblies with multiplex MinION sequencing. *Microb Genom*. 2017;3:e000132.

17 Li R, Xie M, Dong N, et al. Efficient generation of complete sequences of MDR-encoding plasmids by rapid assembly of MinION barcoding sequencing data. *Gigascience*. 2018;7:1-9.

18 George S, Pankhurst L, Hubbard A, et al. Resolving plasmid structures in Enterobacteriaceae using the MinION nanopore sequencer: assessment of MinION and MinION/Illumina hybrid data assembly approaches. *Microb Genom*. 2017;3:e000118.

19 Garalde, DR, Snell, EA, Jachimowicz, D, et al. Highly parallel direct RNA sequencing on an array of nanopores. *Nat Methods*. 2018;15:201-6.

20 Ozsolak F, Milos PM. RNA sequencing: advances, challenges and opportunities. *Nat Rev Genet*. 2011;12: 87-98.

21 Jenjaroenpun P, Wongsurawat T, Pereira R, et al. Complete genomic and transcriptional landscape analysis using third-generation sequencing: a case study of *Saccharomyces cerevisiae* CENPK113-7D. *Nucleic Acids Res*. 2018;46:e38.

22 Workman RE, Tang A, Tang PS, et al. Nanopore native RNA sequencing of a human poly(A) transcriptome. *bioRxiv*. 2018;459529.

23 Moldovan N, Tombacz D, Szucs A, et al. Third-generation Sequencing Reveals Extensive Polycistronism and Transcriptional Overlapping in a *Baculovirus*. *Sci Rep*. 2018;8:8604.

24 Keller MW, Rambo-Martin BL, Wilson MM, et al. Direct RNA Sequencing of the Coding Complete Influenza A Virus Genome. *Sci Rep*. 2018;8:14408.

25 Depledge DP, Srinivas KP, Sadaoka T, et al. Direct RNA sequencing on nanopore arrays redefines the transcriptional complexity of a viral pathogen. *Nat Commun*. 2019;10:754.

26 Smith AM, Jain M, Mulroney L, et al. Reading canonical and modified nucleotides in 16S ribosomal RNA using nanopore direct RNA sequencing. *Plos One*. 2019;14:e0216709.

27 Sorek R, Cossart P. Prokaryotic transcriptomics: a new view on regulation, physiology and pathogenicity. *Nat Rev Genet*. 2010;11:9-16.

28 Pitt ME, Elliott AG, Cao, MD, et al. Multifactorial chromosomal variants regulate polymyxin resistance in extensively drug-resistant *Klebsiella pneumoniae*. *Microb Genom*. 2018;4:mgen1090000158.

29 Olaitan AO, Morand S, Rolain JM. Mechanisms of polymyxin resistance: acquired and intrinsic resistance in bacteria. *Front Microbiol*. 2014;5:643.

30 Teng H, Cao MD, Hall MB, et al. Chiron: translating nanopore raw signal directly into nucleotide sequence using deep learning. *GigaScience*. 2018;7:10.1093/gigascience/giy037.

31 Li H. Aligning sequence reads, clone sequences and assembly contigs with BWA-MEM. *arXiv*. 2013;13033997.

32 Zankari E, Hasman H, Cosentino S, et al. Identification of acquired antimicrobial resistance genes. *J Antimicrob Chemother*. 2012;67:2640-4.

33 Lassmann T, Frings O, Sonnhammer EL. Kalign2: high-performance multiple alignment of protein and nucleotide sequences allowing external features. *Nucleic Acids Res*. 2009;37:858-65.

34 Allison L, Wallace CS, Yee CN. When is a string like a string? In: Artificial Intelligence and Mathematics. *Ft Lauderdale FL*. 1990.

35 Bankevich A, Nurk S, Antipov D, et al. SPAdes: a new genome assembly algorithm and its applications to single-cell sequencing. *J Comput Biol*. 2012;19:455-77.

36 Cao MD, Nguyen SH, Ganesamoorthy D, et al. Scaffolding and completing genome assemblies in real-time with nanopore sequencing. *Nat Commun*. 2017;8:14515.

37 Wick RR, Judd LM, Gorrie CL, et al. Unicycler: Resolving bacterial genome assemblies from short and long sequencing reads. *PLoS Comput Biol*. 2017;13:e1005595.

38 Koren S, Walenz BP, Berlin K, et al. Canu: scalable and accurate long-read assembly via adaptive k-mer weighting and repeat separation. *Genome Res*. 2017;27:722-36.

39 Li H. Minimap and miniasm: fast mapping and *de novo* assembly for noisy long sequences. *Bioinformatics*. 2016;32:2103-10.

40 Vaser R, Sovic I, Nagarajan N, et al. Fast and accurate *de novo* genome assembly from long uncorrected reads. *Genome Res*. 2017;27:737-46.

41 Darling AE, Tritt A, Eisen JA, et al. Mauve assembly metrics. *Bioinformatics*. 2011;27:2756-7.

42 Aziz RK, Bartels D, Best AA, et al. The RAST Server: rapid annotations using subsystems technology. *BMC Genomics*. 2008;9:75.

43 Carattoli A, Zankari E, Garcia-Fernandez A, et al. *In silico* detection and typing of plasmids using PlasmidFinder and plasmid multilocus sequence typing. *Antimicrob Agents Chemother*. 2014;58:3895-903.

44. Li H, Handsaker B, Wysoker A, et al. The sequence alignment/map format and SAMtools. *Bioinformatics*. 2009;25:2078-9.

45 Quinlan AR. BEDTools: The Swiss-Army Tool for Genome Feature Analysis. *Curr Protoc Bioinformatics*. 2014;47:11.12.1-34.

46 Cannatelli A, D'Andrea MM, Giani T, et al. *In vivo* emergence of colistin resistance in *Klebsiella pneumoniae* producing KPC-type carbapenemases mediated by insertional inactivation of the PhoQ/PhoP *mgrB* regulator. *Antimicrob Agents Chemother*. 2013;57:5521-6.

47 Robinson JT, Thorvaldsdottir H, Winckler W, et al. Integrative genomics viewer. *Nat Biotechnol*. 2011;29; 24-6.

48. Robinson MD, McCarthy DJ, Smyth GK. edgeR: a Bioconductor package for differential expression analysis of digital gene expression data. *Bioinformatics*. 2010;26:139-40.

49 Livak KJ, Schmittgen TD. Analysis of relative gene expression data using real-time quantitative PCR and the 2(-Delta Delta C(T)) Method. *Methods*. 2001;25:402-8.

50 Magiorakos AP, Srinivasan A, Carey RB, et al. Multidrug-resistant, extensively drug-resistant and pandrug-resistant bacteria: an international expert proposal for interim standard definitions for acquired resistance. *Clin Microbiol Infect.* 2012;18,268–81.

51 Carattoli A. Resistance plasmid families in Enterobacteriaceae. *Antimicrob Agents Chemother*. 2009;53: 2227-38.

52 Desmet S, Nepal S, van Dijl JM, et al. Antibiotic Resistance Plasmids Cointegrated into a Megaplasmid Harboring the *blaOXA-427* Carbapenemase Gene. *Antimicrob Agents Chemother*. 2018;62:e01448-17.

53 Papagiannitsis CC, Dolejska M, Izdebski R, et al. Characterisation of IncA/C2 plasmids carrying an In416-like integron with the *blaVIM-19* gene from *Klebsiella pneumoniae* ST383 of Greek origin. *Int J Antimicrob Agents*. 2016;47:158-62.

54 Chen L, Chavda KD, Melano RG, et al. Comparative genomic analysis of KPC-encoding pKpQIL-like plasmids and their distribution in New Jersey and New York Hospitals. *Antimicrob Agents Chemother*. 2014;58:2871-7.

55 Poirel L, Bonnin RA, Nordmann P. Genetic features of the widespread plasmid coding for the carbapenemase OXA-48. *Antimicrob Agents Chemother*. 2012;56:559-62.

56 Potron A, Poirel L, Nordmann P. Derepressed transfer properties leading to the efficient spread of the plasmid encoding carbapenemase OXA-48. *Antimicrob Agents Chemother*. 2014;58:467-71.

57 Rang FJ, Kloosterman WP, de Ridder J. From squiggle to basepair: computational approaches for improving nanopore sequencing read accuracy. *Genome Biol*. 2018;19:90.

58 Tamma PD, Fan Y, Bergman Y, et al. Applying rapid whole-genome sequencing to predict phenotypic antimicrobial susceptibility testing results among carbapenem-reistant *Klebsiella pneumoniae* clinical isolates. *Antimicrob Agents Chemother*. 2018;63: pii: e01923-18.

59 Cheruvanky A, Stoesser N, Sheppard AE, et al. Enhanced *Klebsiella pneumoniae* Carbapenemase Expression from a Novel Tn4401 Deletion. *Antimicrob Agents Chemother*. 2017;61:e00025-17.

60 Klontz EH, Tomich AD, Gunther S, et al. Structure and Dynamics of FosA-Mediated Fosfomycin Resistance in *Klebsiella pneumoniae* and *Escherichia coli*. *Antimicrob Agents Chemother*. 2017;61:e01572-17.

61 Saenger W, Orth P, Kisker C, et al. The Tetracycline Repressor-A Paradigm for a Biological Switch. *Angew Chem Int Ed Engl*. 2000;39;2042-52.

62 Cheng YH, Lin TL, Pan YJ, et al. Colistin resistance mechanisms in *Klebsiella pneumoniae* strains from Taiwan. *Antimicrob Agents Chemother*. 2015;59:2909-13.

63 Haeili M, Javani A, Moradi J, et al. MgrB Alterations Mediate Colistin Resistance in *Klebsiella pneumoniae* Isolates from Iran. *Front Microbiol*. 2017;8:2470.

64 Baron S, Hadjadj L, Rolain JM, et al. Molecular mechanisms of polymyxin resistance: knowns and unknowns. *Int J Antimicrob Agents*. 2017;48:583-91.

65. Pitt ME; Nguyen SH; Duarte TPS; Teng H; Blaskovich MAT; Cooper MA; Coin LJM (2020): Supporting data for "Evaluating the Genome and Resistome of Extensively Drug-Resistant Klebsiella pneumoniae using Native DNA and RNA Nanopore Sequencing" GigaScience Database. http://dx.doi.org/10.5524/100695

Table and Figure Legends

Table 1: Final assembly of XDR *K pneumoniae* isolates and location of antibiotic resistance genes

Isolate	ST	Contig	Length (bp)	Coverage	Contig ID*	Resistance Genes**
1_GR_13	147	1	**5181675**	1	C	*blaSHV-11*, *fosA*, *oqxA*, *oqxB*
		2	**192771**	1.95	P: IncA/C2	*aadA1*, *ant(2'')-Ia*, *aph(6)-Id*, *ARR-2*, *blaOXA-10*, *blaTEM-1B*, *blaVEB-1*, *cmlA1*, *dfrA14*, *dfrA23*, *rmtB*, *strA*, *sul1*, *sul2*, *tet(A)*, *tet(G)*
		3	**168873**	2	P: IncFIB$_{pKpn3}$, IncFII$_{pKP91}$	*aadA24*, *aph(3')-Ia*, *aph(6)-Id*, *dfrA1*, *dfrA14*, *strA*
		4	**108879**	1.53	P: IncFIB$_{pKPHS1}$	-
		5	**55018**	14.10	-	-
		6	**53495**	2.36	P: IncR, IncN	*aadA24*, *aph(3')-Ia*, *aph(6)-Id*, *blaVIM-27*, *dfrA1*, *mph(A)*, *strA*, *sul1*
2_GR_12	258	1	**5466424**	1	C	*blaSHV-11*, *fosA*, *oqxA*, *oqxB*
		2	197872	1.3	P: IncFIB$_{pKpn3}$, IncFIIK	*aadA2*, *aph(3')-Ia*, *catA1*, *dfrA12*, *mph(A)*, *sul1*
		3	175636	1.49	P: IncA/C2	*aadA1*, *ant(2'')-Ia*, *aph(3'')-Ib*, *aph(6)-Id*, *ARR-2*, *blaOXA-10*, *blaTEM-1A*, *blaVEB-1*, *cmlA1*, *dfrA14*, *dfrA23*, *rmtB*, *sul1*, *sul2*, *tet(A)*, *tet(G)*
		4	95481	1.61	P: IncFIB$_{pQil}$	*blaKPC-2*, *blaOXA-9*, *blaTEM-1A*
		5	**43380**	1.91	P: IncX3	*blaSHV-12*
		6	**13841**	4	P: ColRNAI	*aac(6')-Ib*, *aac(6')Ib-cr*
16_GR_13	11	1	**5426917**	1	C	*blaSHV-11*, *fosA*, *oqxA*, *oqxB*
		2	**187670**	0.88	P: IncFIB$_{pKpn3}$; IncFIIK	*aac(3)-IIa*, *aac(6')Ib-cr*, *aadA2*, *aph(3')-Ia*, *blaCTX-M-15*, *blaOXA-1*, *catB4*, *dfrA12*, *mph(A)*, *sul1*
		3	**155161**	0.99	P: IncA/ C2	*aadA1*, *ant(2'')-Ia*, *aph(3'')-Ib*, *aph(6)-Id*, *ARR-2*, *blaOXA-10*, *blaTEM-1B*, *blaVEB-1*, *cmlA1*, *rmtB*, *sul1*, *sul2*, *tet(A)*, *tet(G)*
		4	**63589**	1.49	P: IncL/ M$_{pOXA-48}$	*blaOXA-48*
		5	**5234**	188.49	-	-
		6	**4940**	97.77	P: ColRNAI	-

20_GR_12	258	1	**5395894**	1	C	*blaSHV-11, fosA, oqxA, oqxB*
		2	**170467**	1.77	P: $IncFIB_{pKpn3}$; IncFIIK	*aph(3')-Ia, blaKPC-2, blaOXA-9, blaTEM-1A*
		3	**50979**	1.42	P: IncN	*aph(3'')-Ib, aph(6)-Id, blaTEM-1A, dfrA14, sul2, tet(A)*
		4	**43380**	1.78	P: IncX3	*blaSHV-12*
		5	**13841**	10.82	P: ColRNAI	*aac(6')-Ib, aac(6')Ib-cr*

*Contig ID represents chromosome (C) or plasmid (P): replicon determined via PlasmidFinder 1.3.

Resistance genes identified using ResFinder 3.0 (≥90% sequence similarity, ≥60% minimum length) and displayed in alphabetical order. **Bold indicates a circular contig.

Figure 1: Time required to detect antibiotic resistance genes via the real-time emulation analysis using MinION native DNA and RNA sequencing. (**A**) 1_GR_13, (**B**) 2_GR_12, (**C**) 16_GR_13 and (**D**) 20_GR_12. Legend colours identify the class of antibiotic to which the gene confers resistance, / on y-axis indicates reads detected more than one resistance gene and # is a family of genes detected (>3). An asterisk (*) indicates the inability for direct RNA sequencing to detect this gene. Albacore 2.2.7. base-called sequences were used and all reads (pass and fail) were included in this analysis.

Figure 2: Expression of resistance genes via direct RNA sequencing when aligned to completed genomes. (**A**) 1_GR_13, (**B**) 2_GR_12, (**C**) 16_GR_13 and (**D**) 20_GR_12. X-axis depicts the resistance genes and are grouped based on the location in the genome where P indicates a plasmid followed by replicon identity. Albacore 2.2.7 base-called pass and fail reads were used for analysis. Values indicate counts per million mapped reads (post removal of reads mapping to rRNA) and dotted line is set to 300 cpm. Genes represented in order of appearance on contig and (+) indicates the co-expression of genes.

Figure 3: Correlation between resistance genes detected via direct RNA sequencing and validated using qRT-PCR. Relative expression was calculated via normalizing to the housekeeping gene, *rpsL* for both direct RNA sequencing (log2(gene/*rpsL*)) and qRT-PCT ($2^{-(\text{gene}/rpsL)}$). Due to high similarity between certain genes, several primers recognise more than one gene (underlined). These include *aac(6')Ib: aac(6')Ib-cr, aadA24*; *strA: aph(3'')-Ib* and *blaTEM-1: blaTEM-1A, blaTEM-1B.* Values are log10 transformed and shifted +0.001 to display genes with no detectable expression.

Figure 4: Correlation between the four XDR *K pneumoniae* isolates for gene expression via direct RNA sequencing. Top panels display spearman correlation coefficients. The diagonal panel shows the density of gene expression levels in counts per million mapped reads for each sample (post removal of rRNA mapped reads). Bottom panels depict the correlation of gene expression between isolates as a scatter plot. Colours indicate categorization of gene: antimicrobial resistance genes (AMR) as per ResFinder 3.0, virulence genes (VIR) determined via RAST and all other genes or background genes (BG) are displayed. Cpm was capped at 2000.

Figure 5: Expression of genes associated with the polymyxin resistance pathway. Comparison between direct RNA sequencing ((log2(gene/*rpsL*)) and qRT-PCR ($2^{-(\text{gene}/rpsL)}$) All isolates except 20_GR_12 harboured resistance to polymyxin (MIC: >2 µg/mL) and genes were normalised to the housekeeping gene *rpsL*. Values are log10 transformed and shifted +0.001 to display genes with no detectable expression.

Figure 1 Click here to access/download;Figure;Figure_1.docx

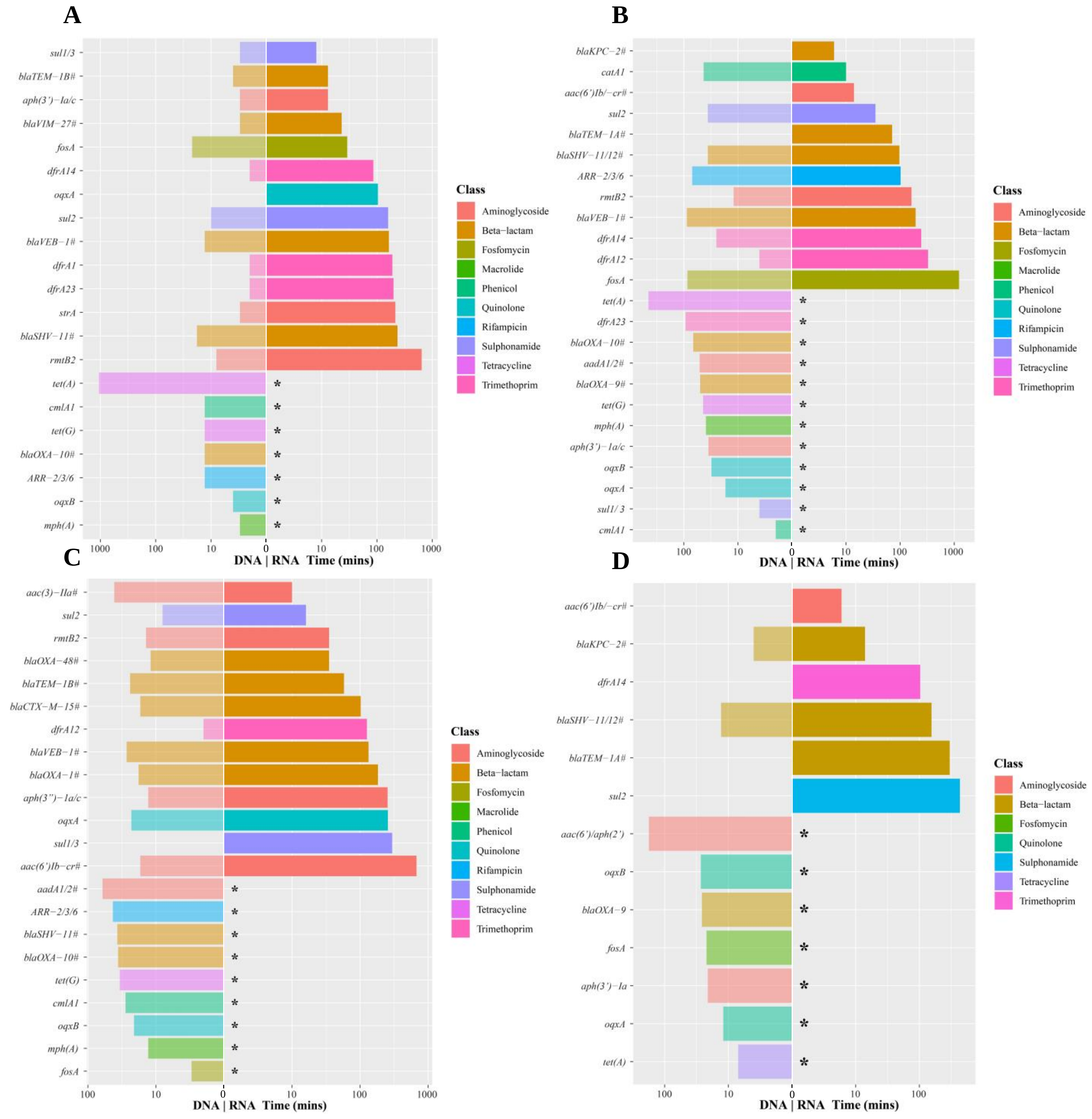

Figure 2

Click here to access/download;Figure;Figure_2.docx

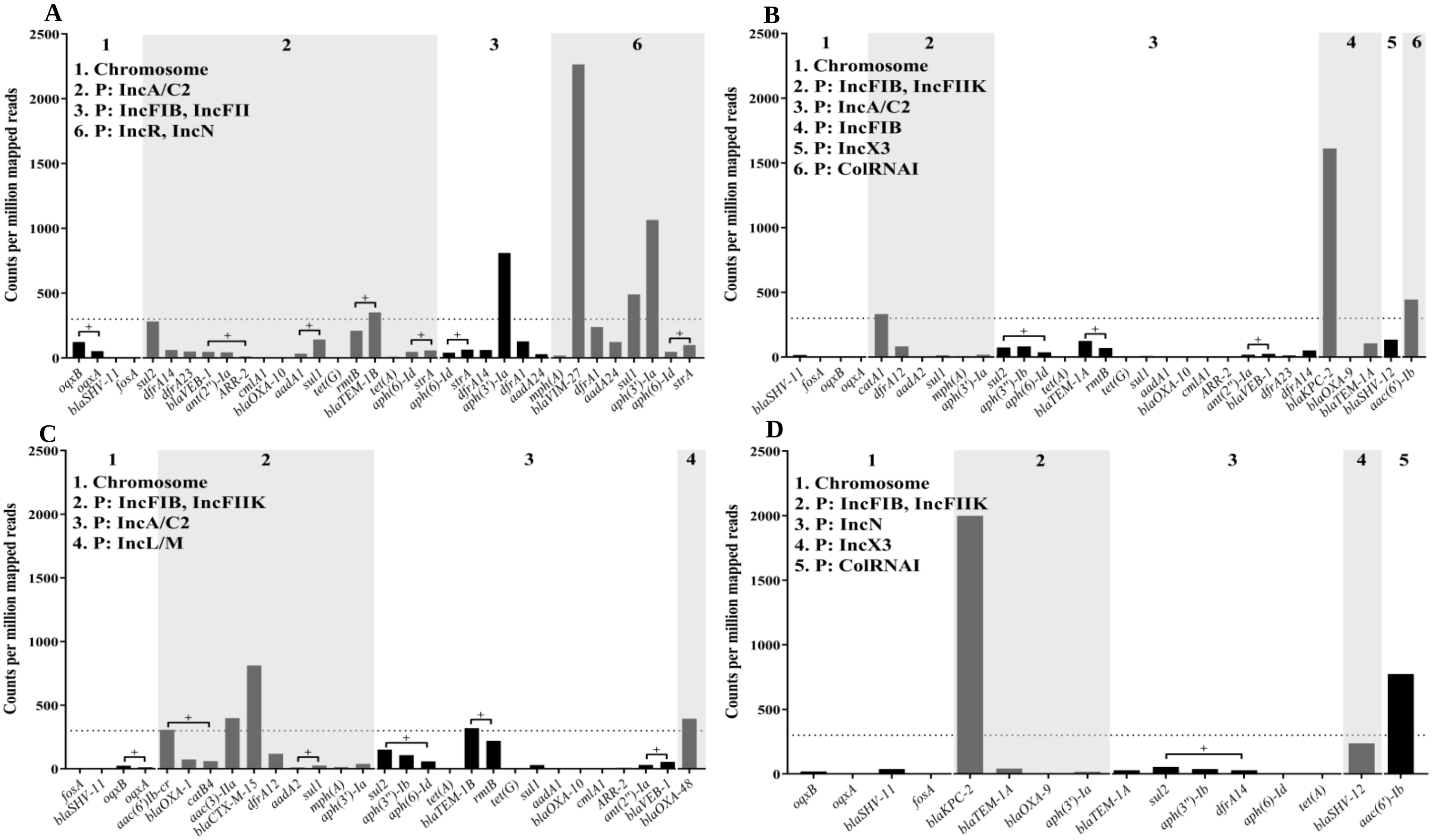

Figure 3 Click here to access/download;Figure;Figure_3.docx

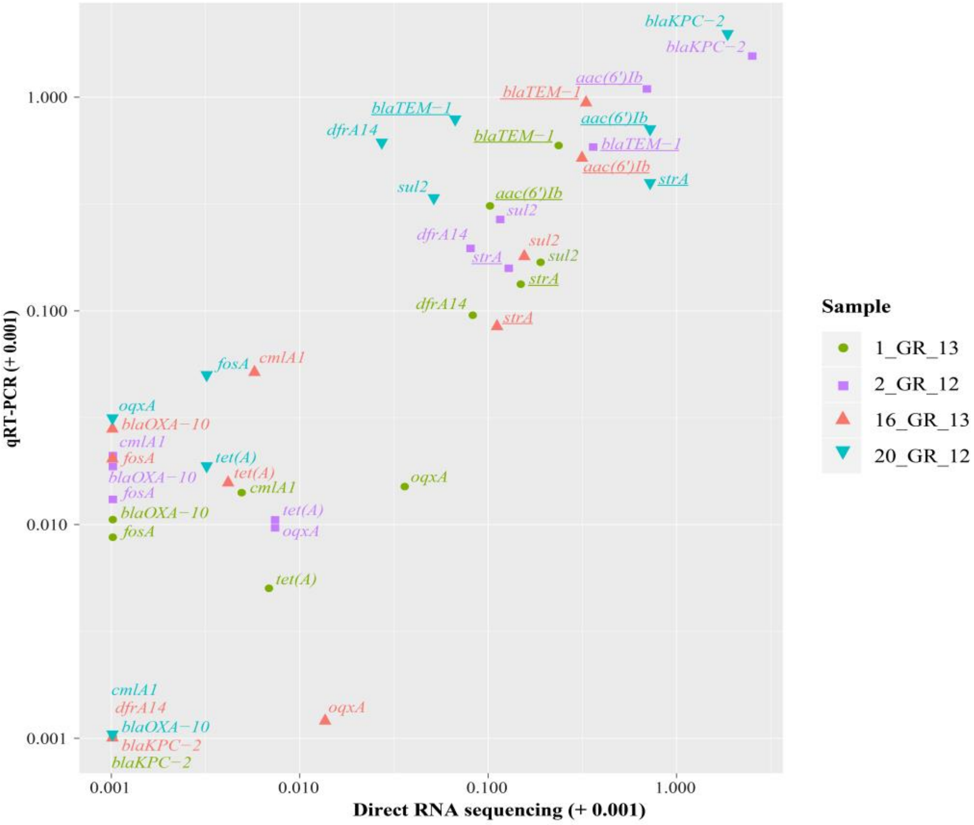

Figure 2

Click here to access/download;Figure;Figure_4.docx

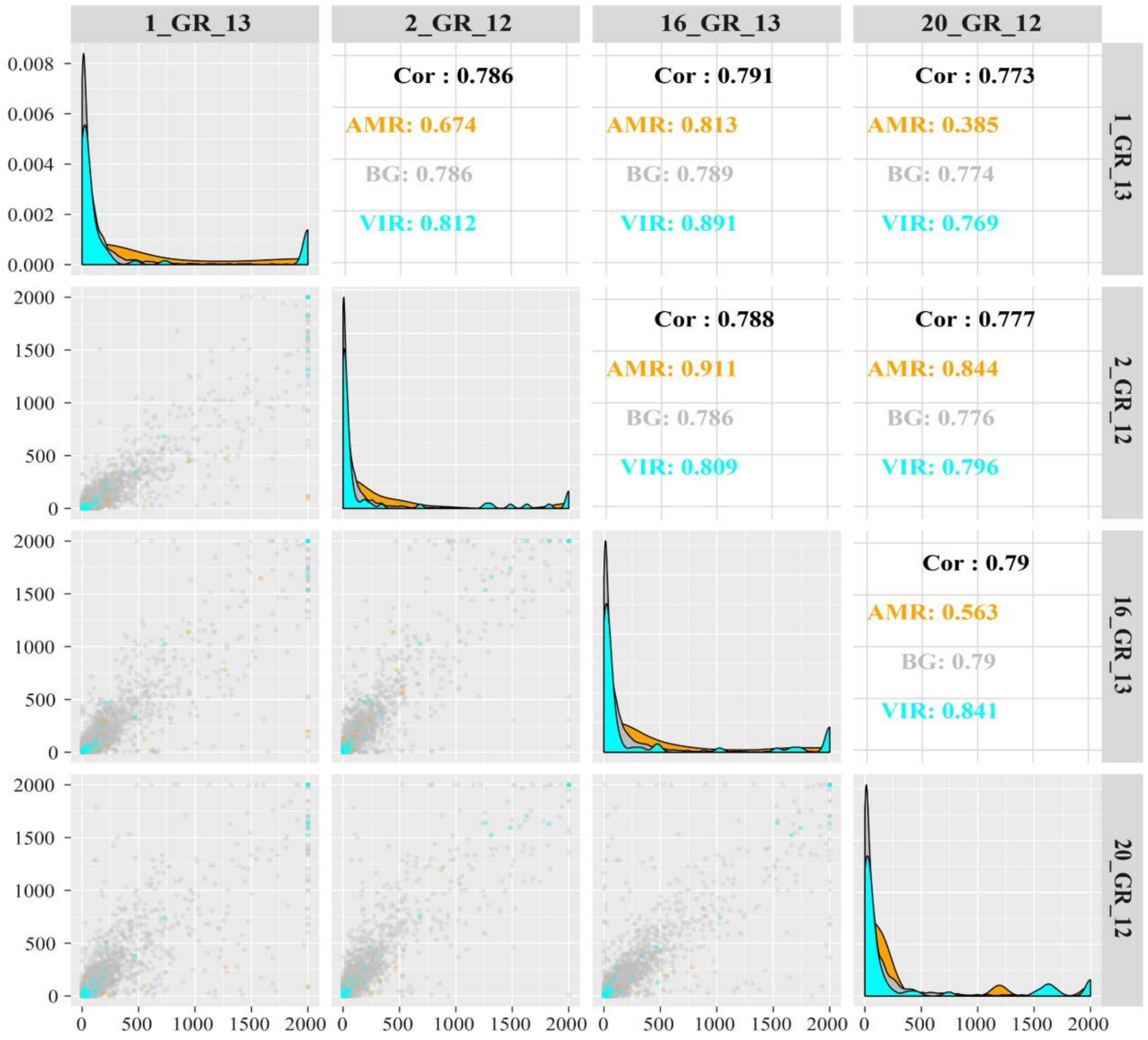

Figure 5

Click here to access/download;Figure;Figure_5.docx

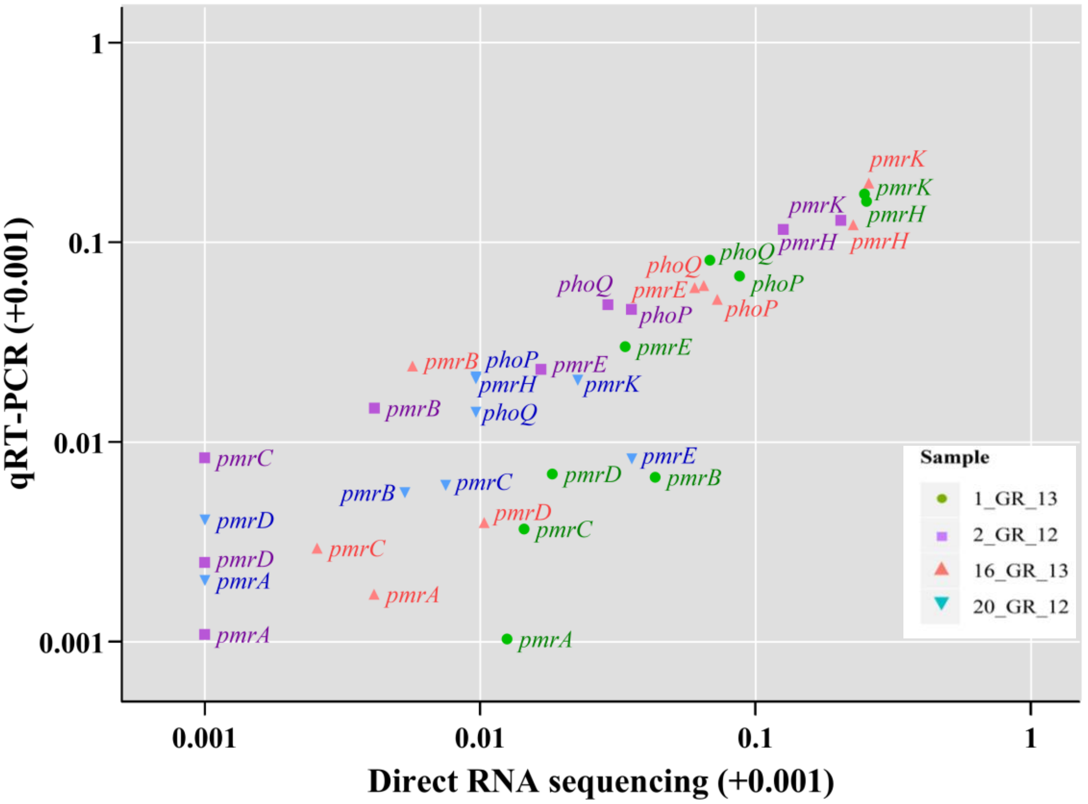

Click here to access/download
Supplementary Material
#SI_GS_Evaluating_XDRKP#_R2.docx

Supplementary Material: Operons

Click here to access/download
Supplementary Material
GIGA-D-19-00200_operons_+rRNA.xlsx

Manuscript

Click here to access/download;Personal Cover;GIGA-D-19-00200_ResponseReviewers_R2.docx

"Evaluating the Genome and Resistome of Extensively Drug-Resistant *Klebsiella pneumoniae* using Native DNA and RNA Nanopore Sequencing"
GIGA-D-19-00200_R2
Response to Reviewers

Dear Dr. Scott Edmunds,

We thank the reviewers for the opportunity to implement additional revisions in our manuscript (GIGA-D-19-00200) which has enabled us to further clarify and strengthen this research via amending figures and conducting further analysis. Please find below a point-by-point response to the reviewer comments.

Reviewer reports:

Reviewer reports:
Reviewer #1: The authors' response to our comments and suggestions has improved this manuscript. We are generally satisfied with the manuscript edits and improvements; however, there are a few more modifications, particularly in the presentation of results, that we feel are required to make this paper acceptable for publishing.

1. The response to pt 1 - (why dRNA) is still largely unsubstantiated. The library prep time being cut in half is a slight advantage, but with cDNA we can start from less material (i.e. perform PCR). Multioperon sequencing could be shown from cDNA as easily as from dRNA - and the authors still haven't shown it. And the authors suggest RNA modifications could be detected, arguably the most unique advantage to dRNA seq in prokaryotes - but showed none of this. We recommend rewording rationale to avoid discouraging cDNA sequencing, especially since it is more practical for most applications at this point - and since the promised improvements for dRNA have yet failed to materialize from ONT, and cDNA yield is currently substantially better.
Response: We agree and have run additional analysis to detect operons (co-expression of genes) using BEDTools intersect which can be found in the results section: "Several resistance genes were identified to be regulated by operons and co-expression was evident for *oqxAB* (1_GR_13, 16_GR_13), *blaVEB-1*:*ant(2")-Ia*:*ARR-2* (1_GR_13), *aadA1*:*sul1* (1_GR_13), *rmtB*:*blaTEM-1B* (1_GR_13, 2_GR_12, 16_GR_13), *aph(6)-Id*:*strA* (1_GR_13), *sul2*:*aph(3")-Ib*:*aph(6)-Id* (2_GR_12, 16_GR_13), *ant(2")-Ia*:*blaVEB-1* (2_GR_12, 16_GR_13), *aac(6')-Ib-cr*:*blaOXA-1*:*catB4* (16_GR_13), *aadA2*:*sul1* (16_GR_13) and *sul2*:*aph(3")-Ib*:*dfrA14* (20_GR_12) (Figure 2). Overall, various non rRNA genes were identified to be co-expressed (≥5 reads supporting gene intersect) across isolates (1_GR_13: 428; 2_GR_12: 310; 16_GR_13: 793; 20_GR_12: 442)." (Line 301-307). We have also uploaded the complete list of operons (including rRNA genes): "GIGA-D-19-00200_operons_+rRNA.xlsx".
Unfortunately, there is currently no robust approach to detect RNA modifications using ONT direct RNA sequencing. The prior study by Garalde *et al* (2018) only interrogated one gene with a known modification and could compare to the same sequence lacking this modification (unmodified). The m6A and A-to-I editing modification can potentially be detected in recent studies (Workman RA *et al* (2019) https://doi.org/10.1038/s41592-019-0617-2. Liu H *et al* (2019) https://doi.org/10.1038/s41467-019-11713-9), however, these studies also had an unmodified dataset for comparison. Generation of the unmodified dataset usually requires *in vitro* transcribed RNA or modification sites can be determined using immunoprecipitation sequencing. These approaches can be costly and time consuming to generate on a whole transcriptome scale. As this dataset has not been generated on our isolates, we are unable to accurately call RNA modifications and hence, why we have not included this in our study. We have reworded the conclusion to highlight some shortcomings of ONT native RNA sequencing: "The expression of resistance genes was successfully detected in addition to identifying genes potentially regulated via

operons, however, native RNA sequencing incurs a slower time to detect resistance due to translocation speed. Once base-calling algorithms have been optimised, this could allow for a whole transcriptome interrogation of the poorly characterised bacterial RNA modifications." Line 423-427.

2. In the "levels of expression of resistance genes" section, lines 270-274, it would be useful to include read counts alongside percentages aligned to increase transparency for counts of reads included in the analysis.
Response: The read counts have now been included in this section. In some instances, a read range was used rather than all the individual values however, the full list of read counts can be found in the Supplementary material (Supplementary Table S6). Line 273-277.

3. In line 281 the authors state "These results reflect the fact that base-calling algorithms have not 282 yet been optimised for direct RNA sequencing, and even less so for bacterial RNA sequencing". However, the accuracies reported in the line above are not atypical of single molecule sequencing, and low alignment % seems largely driven by 400-700nt poly-A tails added- recommend amending this statement.
Response: This line has now been modified: "However, low alignment rates could be attributed to the addition of a long artificial poly(A) which was identified to be approximately 400 to 700 bp across isolates (Supplementary Figure S6)" Line 285-287.

4. More importantly, the alignment % is still alarmingly low - even with current RNA basecallers you should be seeing 80-90% alignment. We recommend filtering reads before alignment by only using "pass" reads, trimming poly-A tails off reads, and removing small reads less than 75nt (which are more likely to be noise), then reporting this alignment percentage.
Response: We now report the alignment rate on pass reads only after poly(A) trimming and removing the small reads less than 75nt, and find that the percentage is actually quite reasonable (≥98% alignment rate). These results have been included in the main text: "Aligning passed reads alone to the final assembly (ensuring trimming of poly(A) tail and removing reads <75 nt), ≥98% (1_GR_13: 95591; 2_GR_12: 138214; 16_GR_13: 227781; 20_GR_12: 119425) of reads were mappable, however, ≤46% (1_GR_13: 42654; 2_GR_12: 46787; 16_GR_13: 79175; 20_GR_12: 54986) of these had a MAPQ score ≥10." Line 274-277.

5. Figure 3- Please denote on the figure itself which primers recognize more than one gene, maybe with underlining? Also, what does the +0.001 notation on axes mean?
Response: Primers which recognize more than one gene have now been underlined in Figure 3. The data in this Figure has been log_{10} transformed hence, to show genes with no detectable expression, the data was shifted by +0.001. This has now been noted in the legend of Figure 3.

6. Figure 5 is overly complicated with the shapes and colors and asterisks - why not just plot the data in the same way as Figure 3, and you can facet by sample?
Response: Figure 5 has now been graphed similar to Figure 3.

Small things:
Spell out XDR first (line 329)
Response: Line 329 has now been amended. (Line 338).
Please parse paragraph lines 348-388 into DNA and RNA sections
Response: This section has now been modified to separate DNA and RNA. (Line 375).
