# Supplementary material for: Evaluating the genome and resistome of extensively drug-resistant Klebsiella pneumoniae using native DNA and RNA Nanopore sequencing
Source: Gigascience. 2020 Feb 4;9(2):giaa002. doi: 10.1093/gigascience/giaa002 (PMC6998412; doi:10.1093/gigascience/giaa002)
Supplement: giaa002_Supplemental_Files [file giaa002_supplemental_files.zip › #SI_GS_Evaluating_XDRKP#_R2.docx]

**Evaluating the Genome and Resistome of Extensively Drug-Resistant *Klebsiella pneumoniae* using Native DNA and RNA Nanopore Sequencing**

Miranda E. Pitt^1,^*, Son H. Nguyen^1^, Tânia P.S. Duarte^1^, Haotian Teng^1^, Mark A.T. Blaskovich^1^, Matthew A. Cooper^1^, Lachlan J.M. Coin^1,^*

^1^Institute for Molecular Bioscience, The University of Queensland, Brisbane, Queensland, 4072, Australia

* To whom correspondence should be addressed. Email: miranda.pitt@imb.uq.edu.au, l.coin@imb.uq.edu.au

**SUPPLEMENTAL MATERIAL**

This pdf contains:

Table S1-6

Figures S1-8

References

**Table S1:** Minimum inhibitory concentrations of the four *Klebsiella pneumoniae* clinical isolates

|  | | **Antibiotic*** | | **1_GR_13** | | **2_GR_12** | | **16_GR_13** | | **20_GR_12** | |
| --- | --- | --- | --- | --- | --- | --- | --- | --- | --- | --- | --- |
| **1** | | AMK | | >64^R^ | | >64^R^ | | >64^R^ | | 64^R^ | |
|  |  | GEN | | >64^R^ | | >64^R^ | | >64^R^ | | 1^S^ | |
|  |  | NET | | >64^R^ | | >64^R^ | | >64^R^ | | ≥64^R^ | |
|  |  | TOB | | >64^R^ | | >64^R^ | | >64^R^ | | 64^R^ | |
| **2** | | CPT | | >32^R^ | | >32^R^ | | >32^R^ | | >32^R^ | |
| **3** | | TZP | | >128/4^R^ | | >128/4^R^ | | >128/4^R^ | | >128/4^R^ | |
|  |  | TIM | | >128/2^R^ | | >128/2^R^ | | >128/2^R^ | | >128/2 | |
| **4** | | DOR | | >64^R^ | | >64^R^ | | 16^I^ | | ≥8^I^ | |
|  |  | ETP | | >64^R^ | | >64^R^ | | 64^R^ | | 64^R^ | |
|  |  | IPM | | ≥64^R^ | | ≥64^R^ | | ≥8^R^ | | ≥8^R^ | |
|  |  | MEM | | >32^R^ | | >32^R^ | | ≥16^R^ | | ≥8^R^ | |
| **5** | | CFZ | | >64^R^ | | >64^R^ | | >64^R^ | | >64^R^ | |
|  |  | CXM | | >64^R^ | | >64^R^ | | >64^R^ | | >64^R^ | |
| **6** | | FEP | | >64^R^ | | >64^R^ | | >64^R^ | | ≥16^R^ | |
|  |  | CTX | | >32^R^ | | >32^R^ | | >32^R^ | | >32^R^ | |
|  |  | CAZ | | >64^R^ | | >64^R^ | | >64^R^ | | >64^R^ | |
|  |  | CRO | | >64^R^ | | >64^R^ | | >64^R^ | | >64^R^ | |
| **7** | CTT | | >64^R^ | | >64^R^ | | >64^R^ | | >64^R^ | |  |
|  | FOX | | >64^R^ | | >64^R^ | | 64^R^ | | 64^R^ | |  |
| **8** | | CIP | | >16^R^ | | >16^R^ | | >16^R^ | | >16^R^ | |
| **9** | | SXT | | >6/>121^R^ | | >6/>121^R^ | | >6/>121^R^ | | >6/>121^R^ | |
| **10** | | TGC | | ≥2^R^ | | 2^I^ | | ≥2^R^ | | ≥2^R^ | |
| **11** | | ATM | | >64^R^ | | >64^R^ | | >64^R^ | | >64^R^ | |
| **12** | | AMP | | >64^R^ | | >64^R^ | | >64^R^ | | >64^R^ | |
| **13** | | SAM | | >64/4^R^ | | >64/4^R^ | | >64/4^R^ | | >64/4^R^ | |
|  |  | AMC | | >64/2^R^ | | >64/2^R^ | | >64/2^R^ | | >64/2^R^ | |
| **14** | | CHL | | 64^R^ | | >64^R^ | | 32^R^ | | ≥32^R^ | |
| **15** | | FOF | | ≤19^R^ | | ≤20^R^ | | ≤19^R^ | | ≤22^R^ | |
| **16** | | CST | | 16^R^ | | 16^R^ | | 64^R^ | | 0.125^S^ | |
|  |  | PMB | | ≥8^R^ | | 8^R^ | | 64^R^ | | ≤0.25^S^ | |
| **17** | | DOX | | 32^R^ | | 32^R^ | | 32^R^ | | 32^R^ | |
|  |  | MIN | | ≥16^R^ | | 16^R^ | | ≤8^I^ | | 16^R^ | |
|  |  | TET | | >64^R^ | | >64^R^ | | >64^R^ | | >64^R^ | |
| **18** | | ERY | | >128^N^ | | >128^N^ | | >128^N^ | | 128^N^ | |
| **19** | | RIF | | >128^N^ | | >128^N^ | | 128^N^ | | 64^N^ | |

*Antibiotic resistance as determined by broth microdilution according to CLSI guidelines (1) except fosfomycin (disk diffusion) and tigecycline which followed EUCAST breakpoints (2). Antibiotic classes tested include **1**, Aminoglycosides (Amikacin, AMK; Gentamicin, GEN; Netilmicin, NET; Tobramycin, TOB); **2**, Anti-MRSA cephalosporins (Ceftaroline, CPT); **3**, Antipseudomonal penicillins + β-lactamase inhibitors (Piperacillin-tazobactam, TZP; Ticarcillin-clavulanic acid, TIM); **4**, Carbapenems (Doripenem, DOR; Ertapenem, ETP; Imipenem, IPM; Meropenem, MEM); **5**, Non-extended spectrum cephalosporins (1^st^ and 2^nd^ generation) (Cefazolin, CFZ; Cefuroxime, CXM); **6**, Extended-spectrum cephalosporins (3^rd^ and 4^th^ generation) (Cefepime, FEP; Cefotaxime, CTX, Ceftazidime, CAZ; Ceftriaxone, CRO); **7**, Cephamycins (Cefotetan, CTT; Cefoxitin, FOX); **8**, Fluoroquinolones (Ciprofloxacin, CIP); **9**, Folate pathway inhibitors (Trimethoprim-sulfamethoxazole, SXT); **10**, Glycylcyclines (Tigecycline, TGC); **11**, Monobactams (Aztreonam, ATM); **12**, Penicillins (Ampicillin, AMP); **13**, Penicillins + β-lactamase inhibitors (Ampicillin-sulbactam, SAM; Amoxicillin-clavulanic acid, AMC); **14**, Phenicols (Chloramphenicol, CHL); **15**, Phosphonic acids (Fosfomycin, FOF); **16**, Polymyxins (Colistin, CST; Polymyxin B, PMB); **17**, Tetracyclines (Doxycycline, DOX; Minocycline, MIN; Tetracycline, TET); **18**, Macrolides (Erythromycin, ERY); **19**, Macrolactams (Rifampicin, RIF). Antibiotic combinations (TZP, TIM, SXT, SAM, AMC) were assayed in accordance to EUCAST ratios or fixed concentrations (2). Shading represents: R, Resistant; I, Intermediate; S, Susceptible; Not Available, N. Values indicate MIC in µg/mL except disk diffusion values for fosfomycin in millimeters.

**Table S2:** Genome assembly comparison

| **Strain** | **Illumina coverage (X)** | **N50** | **Nanopore coverage (X)** | **N50** | **Assembly Method*** | | | |
| --- | --- | --- | --- | --- | --- | --- | --- | --- |
|  |  |  |  |  | **SPAdes/ npScarf** | **SPAdes/ Unicycler** | **Canu** | **Minimap2/ Miniasm/ Racon** |
| 1_GR_13 | 63 | 242938 | 215 | 8712 | **5289533** (IncFIB), **193063** (IncA/C2), **168359** (IncFIB; IncFII), 61039, 55020, **53489** (IncR; IncN), **6457** | **5181675**, **192771** (IncA/C2), 159172 (IncFIB; IncFII), **108879** (IncFIB), **55018**, **53495** (IncR; IncN) | **5073727**, **226704** (IncA/C2), **184820** (IncFIB; IncFII), **136154** (IncFIB), 100186, **82924** (IncR; IncN), 54495 | **5167584**, **192237** (IncA/C2), **168292** (IncFIB; IncFII), **108582** (IncFIB), **53325** (IncR; IncN) |
| 2_GR_12 | 158 | 196706 | 67 | 5251 | 5485776, 457649 (IncFIB; IncA/C2; IncFII), 60365 (IncFII), 42041 (IncX3), 32458, 21300, **13841** (ColRNAI), 12226 | 3743268, 1694231, 175636 (IncA/C2), 152644 (IncFIB; IncFII), 95481 (IncFIB), **43380** (IncX3), 28913, 26127, 16781 (IncFII), 16315, **13841** (ColRNAI) | 5440093, 392198 (IncFIB; IncA/C2; IncFII), 122463 (IncFIB; IncFII), 57534 (IncX3), 26891 (ColRNAI) | 3769033, 1696038, 204124 (IncA/C2), 179356 (IncFIB; IncFII), 158561 (IncFIB), **43085** (IncX3), 2250, 13400 (ColRNAI) |
| 16_GR_13 | 123 | 203529 | 101 | 5012 | **5426917**, **186908** (IncFIB; IncFII), **154971** (IncA/C2), **63588** (IncL/M), 37608, 35578, **5225**, 4426 (ColRNAI), **3703** | **5426765**, **187670** (IncFIB; IncFII), **155161** (IncA/C2), **63589** (IncL/M), **5234**, **4940** (ColRNAI), 2156 (ColRNAI) | **5400611**, **199904** (IncFIB; IncFII), **180542** (IncA/C2), **83467** (IncL/M), 14853, 11623, 9308, 9243, 7568, 7089 | **5410256**, **186950** (IncFIB; IncFII), **154635** (IncA/C2), **63299** (IncL/M), 5100, 4900 |
| 20_GR_12 | 262 | 256217 | 115 | 10151 | **5391578**, **163468** (IncFIB; IncFII), **50940** (IncN), 50856 (IncX3), 12578 (ColRNAI) | **5395894**, **170467** (IncFIB; IncFII), **50979** (IncN), **43380** (IncX3), **13841** (ColRNAI), 4645 | 5342491, **192947** (IncFIB; IncFII), 74844 (IncX3), 72588 (IncN), 25624 (ColRNAI) | **5380057**, **169880** (IncFIB; IncFII), **50636** (IncN), **43157** (IncX3), 13600 (ColRNAI) |

*Output of genome assembly identifies circular sequences (**bold**) and in brackets, plasmid replicons present on these contigs.

**Table S3:** Oligonucleotides used in this study for qRT-PCR

| **Assay** | **Gene** | **Forward sequence (5’ to 3’)** | **Reverse sequence (5’ to 3’)** | **Reference*** |
| --- | --- | --- | --- | --- |
| Acquired resistance | | |  |  |
|  | *aac(6’)Ib^#^* | TTG CAA TGC TGA ATG GAG AG | TGG TCT ATT CCG CGT ACT CC | TS |
|  | *blaKPC-2* | TGG CTA AAG GGA AAC ACG AC | TAG TCA TTT GCC GTG CCA TA | TS |
|  | *blaOXA-10* | GGT GGG TTG AGA AGG AGA CA | ATG ATT TTG GTG GGA ATG GA | TS |
|  | *blaTEM-1^#^* | AAG CCA TAC CAA ACG ACG AG | TTG CCG GGA AGC TAG AGT AA | TS |
|  | *cmlA1* | AAT GGG ATG CCT GAT AGC TG | ACC CAC TAG CCA CAT TGG AG | TS |
|  | *dfrA14* | TTT GAA TCT ATG GGC GCA CT | ATG GCC TCT TCG ATT GAC TG | TS |
|  | *fosA* | CGT GGC GTT TTA TCA GCA G | ACA GGC ACA GCC ACA AAT C | TS |
|  | *oqxA* | GCG ATG ATG CTC TCC TTT CT | GAT CGA CTT CAC CAG CAC CT | TS |
|  | *strA^#^* | ACT CTT CAA TGC ACG GGT CT | CCA GTT CTC TTC GGC GTT AG | TS |
|  | *sul2* | GAT ATT CGC GGT TTT CCA GA | GTC TTG CAC CGA ATG CAT AA | TS |
|  | *tetA* | TTG GCA TTC TGC ATT CAC TC | GAA GGC AAG CAG GAT GTA GC | TS |
|  |  |  |  |  |
| Polymyxin resistance | | |  |  |
|  | *phoP* | ATT GAA GAG GTT GCC GCC CGC | GCT TGA TCG GCT GGT CAT TCA CC | TS |
|  | *phoQ* | GCA TAT CTT CCC GCT GTC AT | GCT AAC GCT ATA GCC CAC CA | TS |
|  | *pmrA* | GAT GAA GAC GGG CTG CAT TT | ACC GCT AAT GCG ATC CTC AA | 3 |
|  | *pmrB* | TGC CAG CTG ATA AGC GTC TT | TTC TGG TTG TTG TGC CCT TC | 3 |
|  | *pmrC* | GCG TGA TGA ATA TCC TCA CCA | CAC GCC AAA GTT CCA GAT GA | 3 |
|  | *pmrD* | GAT CGC AGA GAT TGA AGC CT | GCG TTG CGA ATC TTC AAA GT | 3 |
|  |  |  | GCG TTG CGG ATC TTC AAA GT^1, 16^ | TS |
|  | *pmrE* | GGG TTG ATC TCT GTG ACA TC | GCC TAC CGT AAT GCC GAC TA | 3 |
|  |  | GGG TTA ATC TCC GCA ACA TC^16^ | TGC ATA TCG CAA TGC TGA CTA^16^ | TS |
|  |  |  | GCA TAC CGT AAT GCC GAC TA^1^ | TS |
|  | *pmrH* | CCG CAT CCG TAG CCT GAA | CGT GGG TCT GGC GAT CAT | 4 |
|  | *pmrK* | AGT ATC GGT CAG TGG CTG TT | CCG CTT ATC ACG AAA GAT CC | 3 |
|  | *rpsL* | CCG TGG CGG TCG TGT TAA AGA | GCC GTA CTT GGA GCG AGC CTG | 5 |

*A (TS) indicates primers were designed in this study. Superscript after a primer designates alternative coding for: ^1^, 1_GR_13 (ST147) and ^16^, 16_GR_13 (ST11). ^#^ Primer recognises multiple genes, *aac(6’)Ib: aac(6’)Ib-cr, aadA24*, *strA: aph(3'')-Ib* and *blaTEM-1: blaTEM-1A, blaTEM-1B.*

**Table S4:** Highest similarity observed for final assembly contigs when aligned to NCBI database

| **Isolate** | **ST** | **Contig** | **Length (bp)** | **Contig ID** | **Accession number** | **Query Coverage** | **Identity** |
| --- | --- | --- | --- | --- | --- | --- | --- |
| **1_GR_13** | 147 | 1 | **5181675** | C | CP018719.1^*^ | 99 | 99 |
|  |  | 2 | **192771** | P: IncA/C2 | CP008824.1^*^ | 76 | 99 |
|  |  | 3 | **168873** | P: IncFIB_pKpn3_, IncFII_pKP91_ | CP006657.1^*^ | 89 | 99 |
|  |  | 4 | **108879** | P: IncFIB_pKPHS1_ | CP023929.1^*^ | 92 | 99 |
|  |  | 5 | **55018** | - | CP023927.1^*^ | 100 | 99 |
|  |  | 6 | **53495** | P: IncR, IncN | CP023926.1^*^ | 92 | 99 |
| **2_GR_12** | 258 | 1 | **5466424** | C | LT216436.1^*^ | 99 | 99 |
|  |  | 2 | 197872 | P: IncFIB_pKpn3_, IncFIIK | CP021713.1^*^ | 97 | 99 |
|  |  | 3 | 175636 | P: IncA/C2 | LT882698.1^*^ | 80 | 99 |
|  |  | 4 | 95481 | P: IncFIB_pQil_ | CP023928.1 | 100 | 99 |
|  |  | 5 | **43380** | P: IncX3 | CP009776.1^*^ | 100 | 99 |
|  |  | 6 | **13841** | P: ColRNAI | CP010362.1^*^ | 99 | 99 |
| **16_GR_13** | 11 | 1 | **5426917** | C | CP018438.1^#^ | 97 | 99 |
|  |  | 2 | **187670** | P: IncFIB_pKpn3_; IncFIIK | CP018693.1^#^ | 90 | 100 |
|  |  | 3 | **155161** | P: IncA/ C2 | KX029331.1^#^ | 84 | 99 |
|  |  | 4 | **63589** | P: IncL/ M_pOXA-48_ | CP018723.1^#^ | 100 | 99 |
|  |  | 5 | **5234** | - | CP025818.1^*^ | 99 | 99 |
|  |  | 6 | **4940** | P: ColRNAI | CP024579.1 | 84 | 99 |
| **20_GR_12** | 258 | 1 | **5395894** | C | CP027160.1^#^ | 96 | 99 |
|  |  | 2 | **170467** | P: IncFIB_pKpn3_; IncFIIK | CP022574.1^#^ | 99 | 99 |
|  |  | 3 | **50979** | P: IncN | CP026053.1^#^ | 93 | 99 |
|  |  | 4 | **43380** | P: IncX3 | CP009776.1^*^ | 100 | 99 |
|  |  | 5 | **13841** | P: ColRNAI | CP010362.1^*^ | 100 | 100 |

Query coverage indicates percentage (%) of sequence from NCBI database aligning to contig and identity (%) represents sequence similarity.

^#^Indicates the full length of the previously reported sequence is ≥90% the length of the contig and ^*^ is a sequence longer than the contig.

| **Isolate** | **Time (mins)** | **DNA** | **RNA** |
| --- | --- | --- | --- |
|  |  | **Resistance gene/s detected*** | |
| **1_GR_13** | 10 | ***oqxA*** (Q), ***dfrA1*** (Tr), ***dfrA14*** (Tr), ***dfrA23*** (Tr), ***blaVIM-27^#^*** (B)**, *sul1****/3* (S), ***strA*** (A), ***aph(3’)-Ia****/c* (A), *strB* (A), *aadB* (A), ***mph(A)*** (M), ***blaTEM-1B^#^*** (B), ***oqxB*** (Q), ***rmtB2*** (A) | ***sul1****/ 3* (S) |
|  | 30 | ***sul2*** (S), ***ARR-2****/3/6* (R), ***blaOXA-10^#^*** (B), ***blaVEB-1^#^*** (B), ***tet(G)*** (T), ***cmlA1*** (P), *floR* (P), ***blaSHV-11^#^*** (B), ***fosA*** (F) | ***aph(3')-Ia/c*** (A)*,* ***blaTEM-1B^#^***(B)*,* ***blaVIM-27^#^*** (B)*,* ***fosA*** (F) |
|  | 60 | *ARR-3* (R), *aac(6’)Ib^#^* (A), *ARR-7* (R), *aac(2’)* (A), *cml* (P) | *ARR-7* (R) |
|  | 120 | *aac(3’)-IIIc* (A) | ***dfrA14*** (Tr)*,* ***oqxA*** (Q) |
|  | 300 | *aac(3’)-IIIb* (A), *aac(6’)-Ic* (A), | *strB* (A)*,* ***sul2*** (S)*,* ***blaVEB-1^#^*** (B**)** *,* ***dfrA1*** (Tr)*,* ***dfrA23*** (Tr)*,* ***strA*** (A)*,* ***blaSHV-11^#^*** (B) |
|  | 600 | *blaPAO* (B), *aph(3’)-IIb* (A)*, aph(6)-Ic* (A) | *aadB* (A) |
|  | 900 | *catpC233* (P), *blaOKP* (B) | ***rmtB2*** (A) |
|  | 1200 | ***tet(A)*** (T) | - |
| **2_GR_12** | 10 | ***blaKPC-2^#^*** (B), ***blaTEM*-*1A*^#^**(B), ***aac6Ib/-cr^#^*** (A), ***cmlA1* (**P), ***dfrA12*** (Tr), ***sul1****/ 3* (S) | ***blaKPC-2^#^*** (B)*,* ***catA1*** (P) |
|  | 30 | ***rmtB2*** (A), ***oqxA*** (Q), ***dfrA14*** (Tr), *blaPAO* (B) | ***aac6Ib/-cr^#^*** (A) |
|  | 60 | ***oqxB*** (Q), *strA* (A), *strB* (A), ***aph(3’)-1a****/c* (A), ***sul2*** (S), ***blaSHV-11/12^#^*** (B), ***mph(A)*** (M), ***catA1*** (P), ***tet(G)*** (T), ***blaOXA-9^#^*** (B), ***aadA1/2^#^*** (A), *fusB* (Fu), ***aac(6’)****/aph(2”)* (A) | ***sul2*** (S) |
|  | 120 | ***blaOXA-10^#^*** (B), *floR* (P), ***ARR-2****/3/6* (R), *aadB* (A), ***fosA*** (F), ***blaVEB-1^#^*** (B), *cml* (P), ***dfrA23*** (Tr) | ***blaTEM-1A^#^*** (B)*,* ***blaSHV-11/12^#^***(B) *,* ***ARR-2^#^***(R)*,* *aadB* (A) |
|  | 300 | *aac(3’)-IIIc* (A), *catpC233* (P), *aac(3’)-IIIb* (A), *ARR-3* (R) | ***rmtB2*** (A)*,* ***blaVEB-1^#^***(B)*,* *strB* (A)*, strA* (A)*,* ***dfrA14*** (Tr) |
|  | 600 | ***tet(A)*** (T) | ***dfrA12*** (Tr) |
|  | 900 | *aph(6)-Ic* (A) | - |
|  | 1200 | - | ***fosA*** (F) |
| **16_GR_13** | 10 | *blaOXA***-***436* (B), ***sul1****/3* (S), ***dfrA12*** (Tr), ***fosA*** (F), *aadB* (A), *strA* (A), ***sul2*** (S), *strB* (A), *blaCTX-M-64* (B) | - |
|  | 30 | ***blaOXA*-*48^#^*** (B), ***aph(3’)-1a****/c* (A), ***mph(A)*** (M), ***rmtB2*** (A), *floR* (P), ***blaCTX-M-15****^#^* (B), ***aac(6’)Ib-cr^#^*** (A), ***blaOXA-1^#^*** (B), ***oqxB*** (Q), ***oqxA*** (Q), ***blaTEM-1B^#^*** (B), ***blaVEB-1***^#^ (B), ***cmlA1*** (P) | ***aac(3')-IIa^#^*** (A)*,* ***sul2*** (S)*,* *blaOXA-436* (B) |
|  | 60 | ***tet(G)*** (T), ***blaOXA-10^#^*** (B), ***blaSHV-11^#^*** (B), ***aac(3’)-IIa****^#^* (A), ***ARR-2****/3/6* (R) | ***rmtB2*** (A)*,* ***blaOXA-48^#^*** (B)*,* *strA* (A)*,* ***blaTEM-1B^#^*** (B) |
|  | 120 | ***aadA1/2^#^*** (A), *fusB* (Fu), *ermT* (M), *str* (A), *rmtG* (A), ***aac(6’)****/aph(2”)* (A) | *blaCTX-M-64* (B)*,* ***blaCTX-M-15*** (B) |
|  | 300 | *ARR-3* (R), *catpC233* (P), *cml* (P), *aph(6)-Ic* (A), *aac(3’)-IIIb* (A) | ***dfrA12*** (Tr)*,* *strB* (A)*,* ***blaVEB-1^#^*** (B)*,* ***blaOXA-1^#^*** (B)*,* ***aph(3')-Ia/****c* (A)*,* ***oqxA*** (Q)*,* ***sul1/*** *3* (S) |
|  | 600 | *vanR* (V), *dfrA14* (Tr) | - |
|  | 900 | *blaPAO* (B) | *aadB* (A)*,* ***aac(6')Ib-cr^#^*** (A) |
|  | 1200 | *aph(3’)-IIb* (A) | - |
| **20_GR_12** | 10 | ***aac(6’)Ib/-cr^#^*** (A), ***blaTEM-1A^#^*** (B), ***dfrA14*** (Tr), ***sul2*** (S), *strB* (A), ***blaKPC-2^#^*** (B), ***tet(A)*** (T) | ***aac(6')Ib/-cr^#^*** (A) |
|  | 30 | ***oqxA*** (Q), ***blaSHV-11/12^#^*** (B), ***aph(3’)-Ia*** (A), ***fosA*** (F), ***blaOXA-9*** (B), ***oqxB* (**Q), *aph(6)-Ic* (A) | ***blaKPC-2^#^*** (B) |
|  | 60 | - | - |
|  | 120 | *aac(2’)* (A), *catpC233* (P), *aac(3’)-IIIb* (A) | ***dfrA14*** (Tr) |
|  | 300 | *ermT* (M), *blaPAO* (B), ***aac(6’)****/aph(2”)* (A), *catpC221* (P) | ***blaSHV-11/12^#^*** (B)*,* ***blaTEM-1A^#^*** (B) |
|  | 600 | *rmtf* (A), *ermG* (M) | ***sul2*** (S) |
|  | 900 | *aac(3’)-IIIc* (A), *aac(6’)-Ic* (A) | - |
|  | 1200 | *vatB* (M), *ARR-2/3/6* (R), *aadB* (A) | - |

**Table S5:** Real-time emulation of time to detect resistance genes from DNA and RNA sequencing

*Resistance genes detected as per the real-time emulation. **Bold** represents gene or gene family detected in final assembly and ^#^ indicates more than three genes grouped within this family. Genes displayed in order of time detected. An underline identifies genes with a low mapping quality (MAPQ: ≤10). Brackets represent class of antibiotic this gene confers resistance which includes: A, aminoglycoside; B, β-lactam; F; fosfomycin; Fu; fusidic acid, M, macrolide; P, phenicol; Q, quinolone; R, rifampicin; S, sulphonamide; T, tetracycline;

Tr, trimethoprim; V, vancomycin. Albacore base-calling used for both datasets.

**Table S6:** Comparison between different base-calling programs using direct RNA sequencing data

|  |  | **1_GR_13** | **2_GR_12** | **16_GR_13** | **20_GR_12** |
| --- | --- | --- | --- | --- | --- |
| **Albacore 2.2.7** | Total reads (Pass reads:%) | 974841 (97327:9.98%) | 972436 (142371:14.64%) | 1725702 (233610:13.54%) | 1175902 (123689:10.52%) |
|  | Aligned reads (%) | 130982 (13.40%) | 208784 (21.47%) | 294271 (17.05%) | 175738 (14.95%) |
|  | Identity rate | 0.8449 | 0.8409 | 0.8520 | 0.8569 |
|  | MAPQ (≥10) | 50669 | 45736 | 91145 | 61892 |
| **Chiron v0.5** | Total reads | 979830 | 1229380 | 1744007 | 2250487 |
|  | Aligned reads | 76572 (7.81%) | 190967 (15.53%) | 273772 (15.70%) | 113568 (5.05%) |
|  | Identity rate | 0.7596 | 0.8028 | 0.7895 | 0.7758 |
|  | MAPQ (≥10) | 26490 | 42906 | 72613 | 38151 |
| **Guppy 3.0.3** | Total reads (Pass reads:%) | 974841 (13201:1.35%) | 813661 (94941:11.67%) | 1727272 (75172:4.35%) | 1175902 (44283:3.77%) |
|  | Aligned reads (%) | 44107 (4.52%) | 189640 (23.31%) | 162223 (9.39%) | 133251 (11.33%) |
|  | Identity rate | 0.8164 | 0.8665 | 0.8516 | 0.8503 |
|  | MAPQ (≥10) | 15109 | 39225 | 42999 | 36888 |

All reads (pass (quality score ≥7) and fail) aligned to updated completed assemblies using BWA-MEM (parameters: -k 11 -W20 -r10 -A1 -B1 -O1 -E1 -L0).

Identity rate takes into consideration deletions, insertions and mismatches when comparing against the reference genome. Preliminary analysis using minimap2 [6] revealed a lower alignment rate potentially due to the majority of reads being <1000 bp (Figure S3).


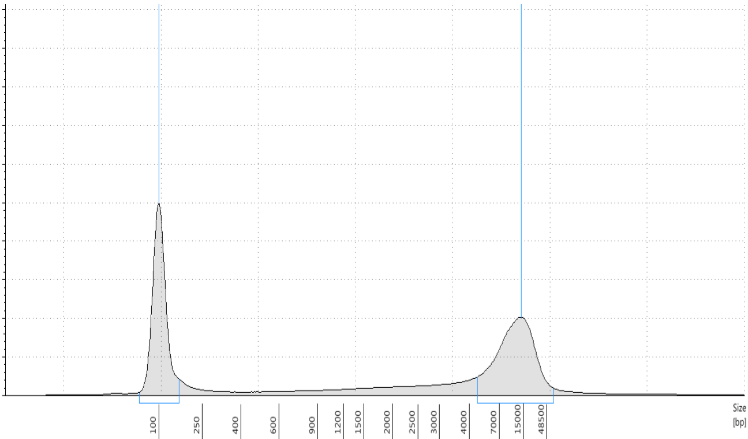


**M**

**13739**


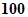

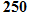

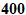

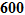

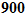

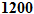

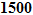

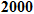

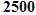

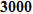

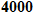

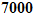

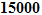

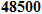


**0**

**100**

**200**

**300**

**400**

**500**

**600**

**700**

**800**

**900**

**1000**

**Length (bp)**


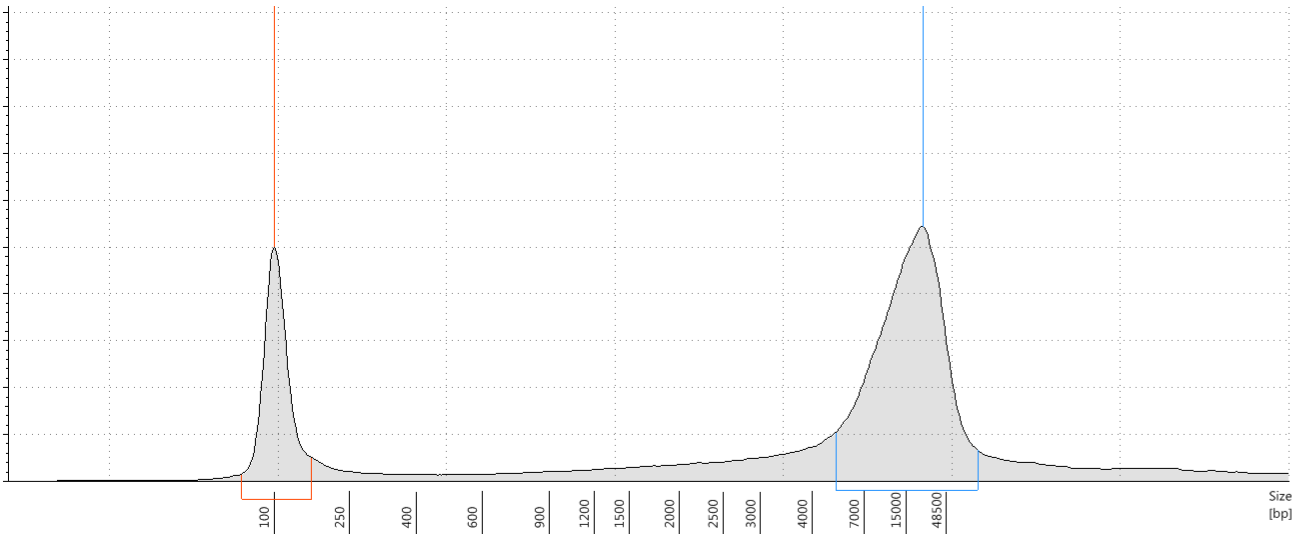


**M**

**20253**

**0**

**100**

**200**

**300**

**400**

**500**

**600**

**700**

**800**

**900**

**1000**


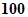

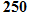

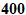

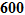

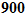

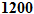

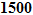

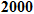

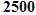

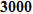

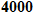

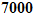

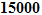

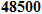


**Length (bp)**


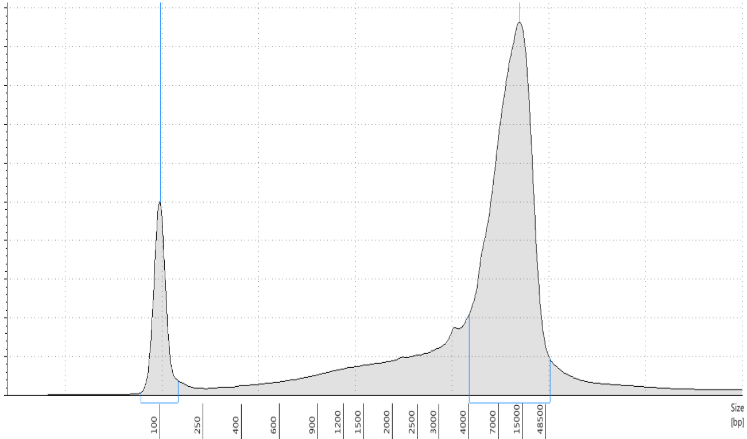

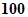

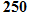

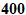

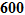

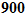

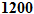

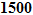

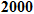

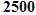

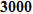

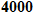

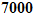

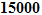

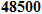


**0**

**100**

**200**

**300**

**400**

**500**

**600**

**700**

**800**

**900**

**1000**

**M**

**13582**

**Length (bp)**


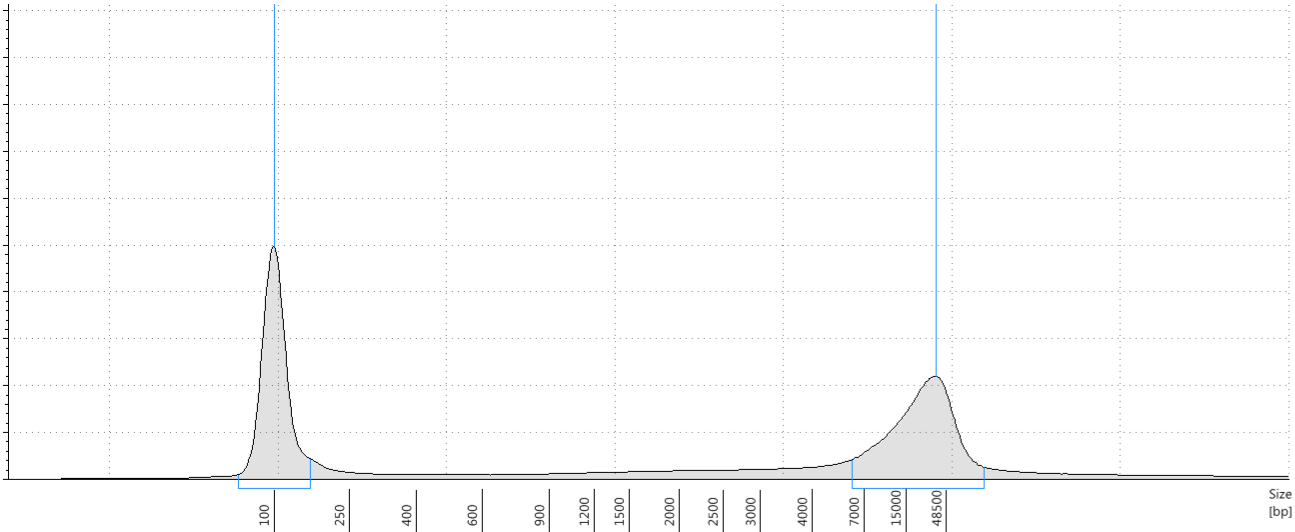

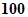

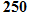

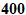

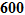

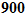

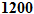

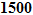

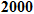

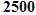

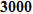

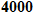

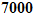

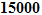

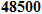


**0**

**100**

**200**

**300**

**400**

**500**

**600**

**700**

**800**

**900**

**1000**

**M**

**26364**

**Length (bp)**

**A**

**B**

**C**

**D**


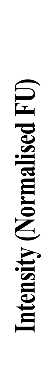

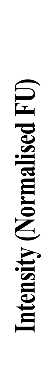

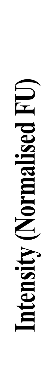

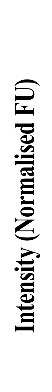


**Figure S1:** Tapestation traces of high molecular weight DNA samples including, (**A**) 1_GR_13, (**B**) 2_GR_12, (**C**) 16_GR_13, (**D**) 20_GR_12. The 100 nt marker is indicated with an M and number indicates the most abundant length.

**Nucleotide length (bp)**


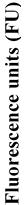


**A**

**M**

**16S**

**23S**


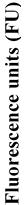


**Nucleotide length (bp)**

**M**

**B**


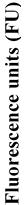


**Nucleotide length (bp)**

**C**

**M**

**16S**

**23S**


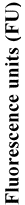


**Nucleotide length (bp)**

**M**

**D**


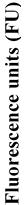


**Nucleotide length (bp)**

**M**

**16S**

**23S**

**E**


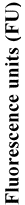


**Nucleotide length (bp)**

**F**

**M**


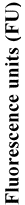


**Nucleotide length (bp)**

**M**

**16S**

**23S**

**G**


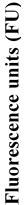


**Nucleotide length (bp)**

**M**

**H**

**Figure S2:** Bioanalzyer traces comparing mRNA enrichment and subsequent poly(A) ligation for RNA samples. Sample conditions are as follows: (**A**) 1_GR_13 rRNA depletion, (**B**) 1_GR_13 poly(A) ligation, (**C**) 2_GR_12 rRNA depletion, (**D**) 2_GR_12 poly(A) ligation, (**E**) 16_GR_13 rRNA depletion, (**F**) 16_GR_13 poly(A) ligation, (**G**) 20_GR_12 rRNA depletion, (**H**) 20_GR_12 poly(A) ligation. The 25 nt marker is indicated with an M, presence of 16S rRNA (16S) and 23S rRNA (23S) are also shown.

**A**

**B**

**C**

**D**

**E**

**F**

**G**

**H**


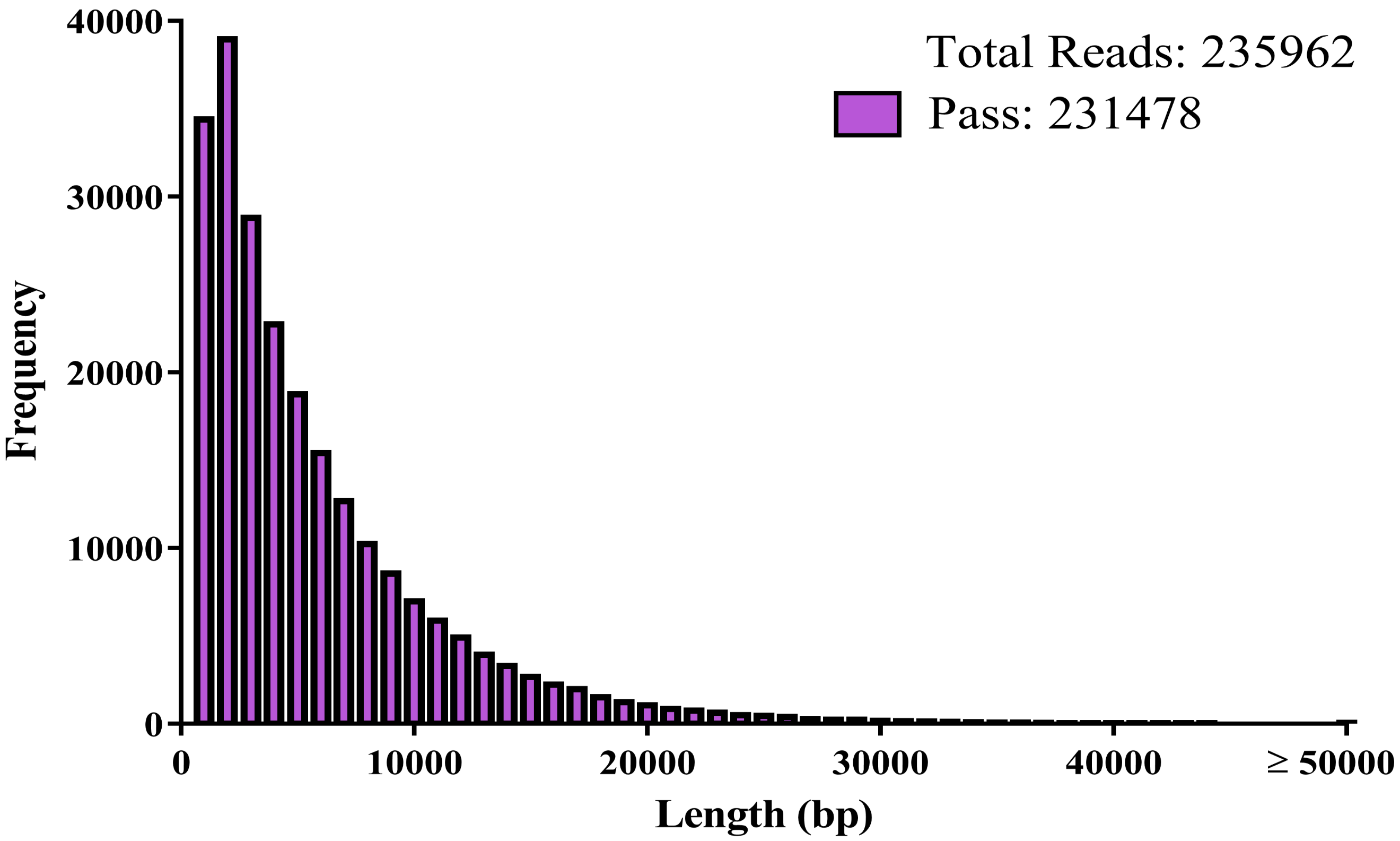

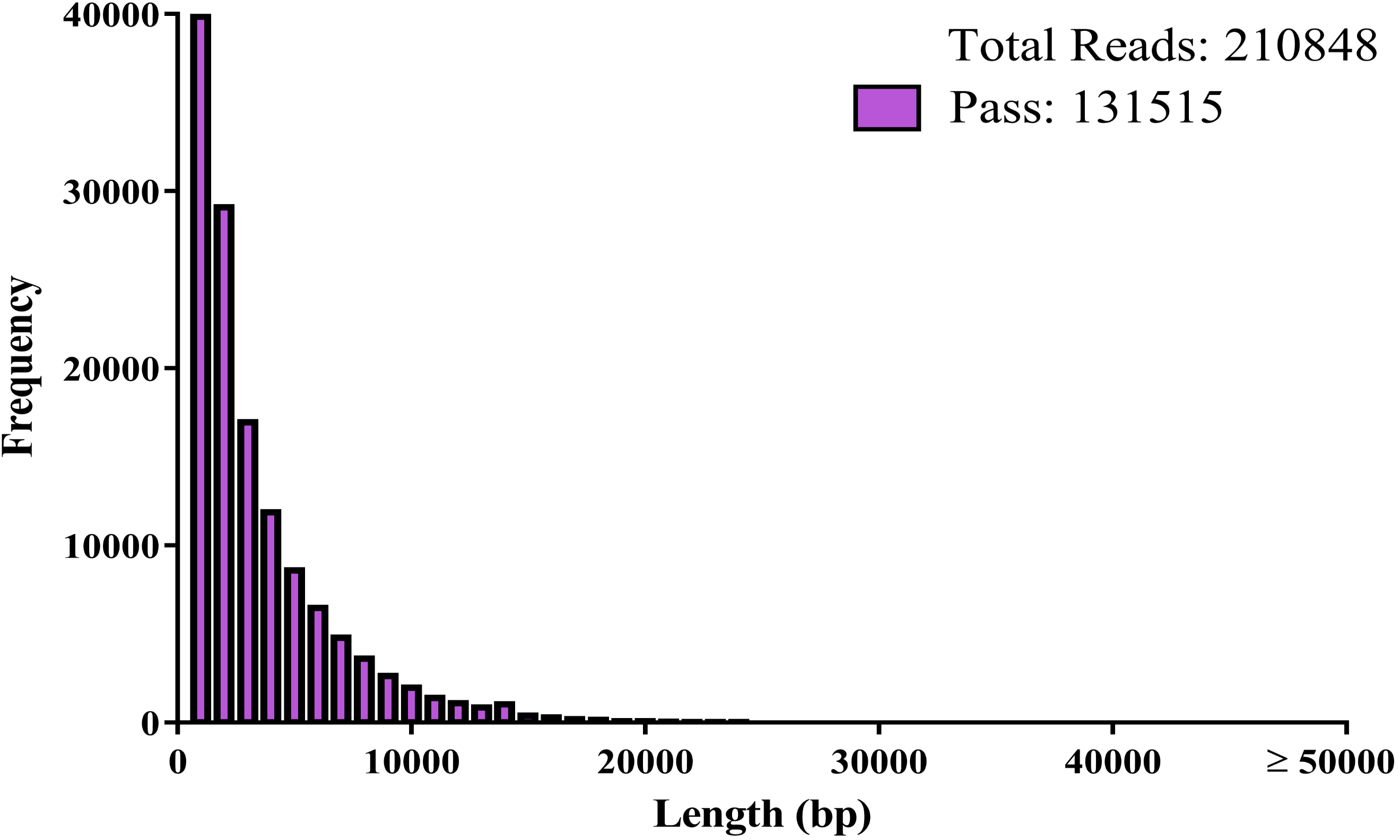

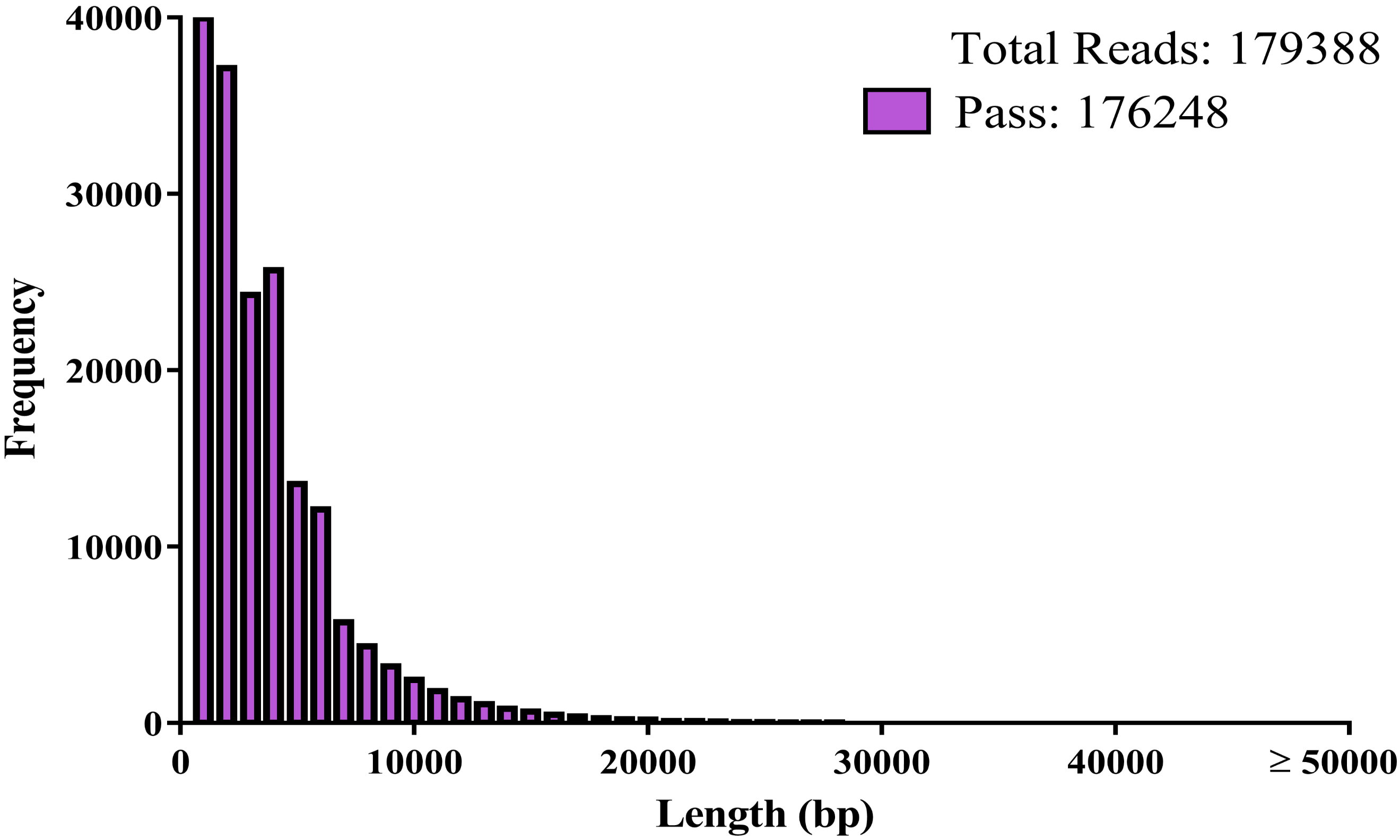

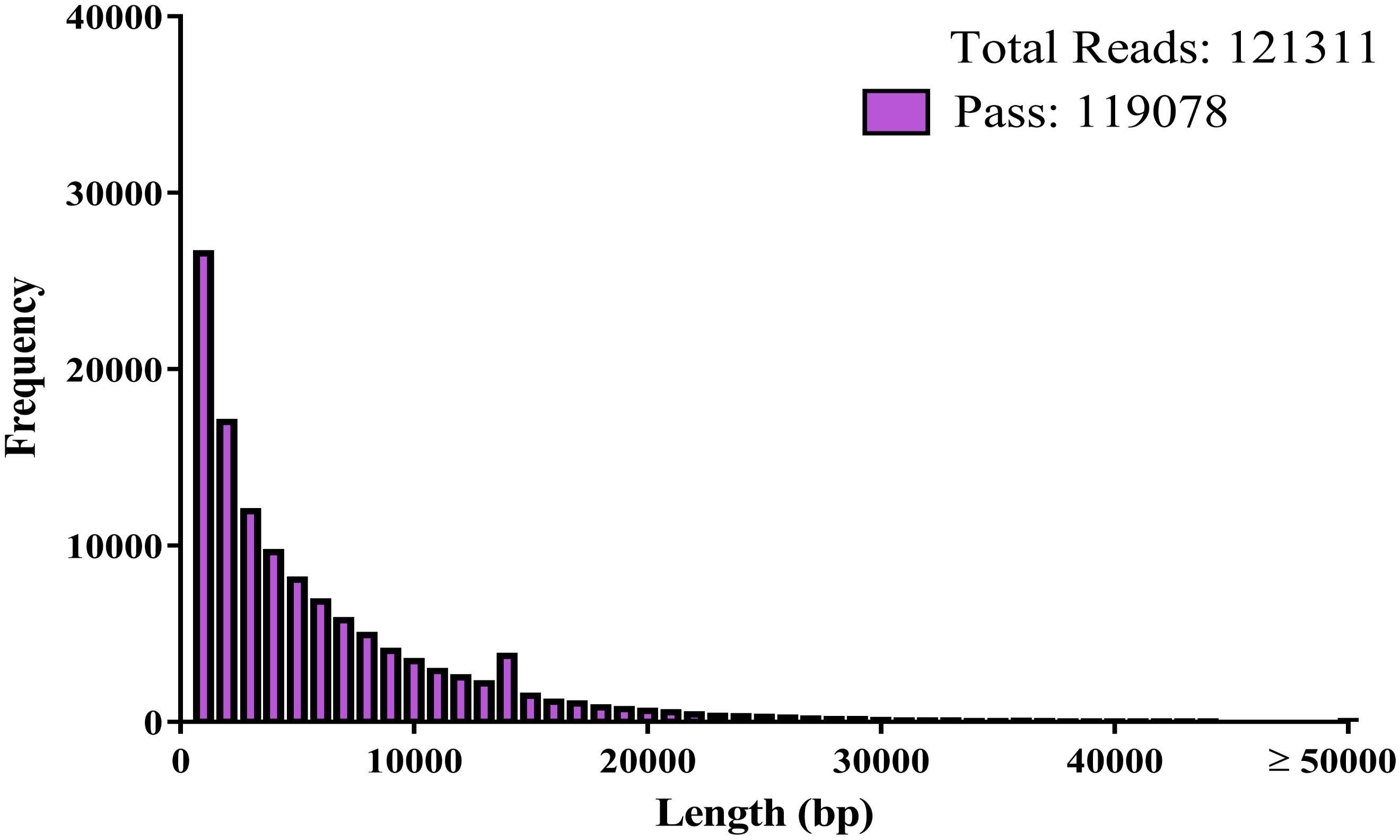

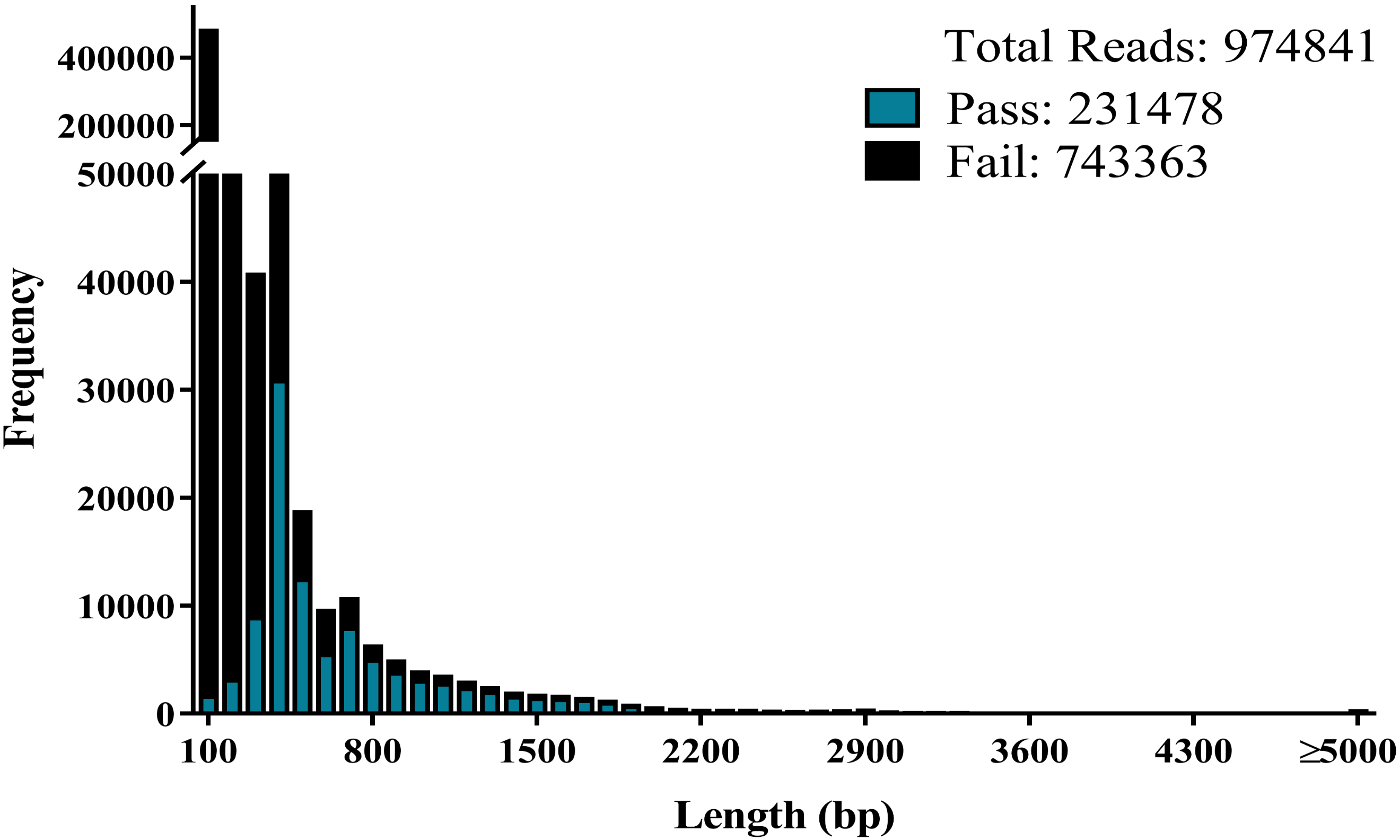

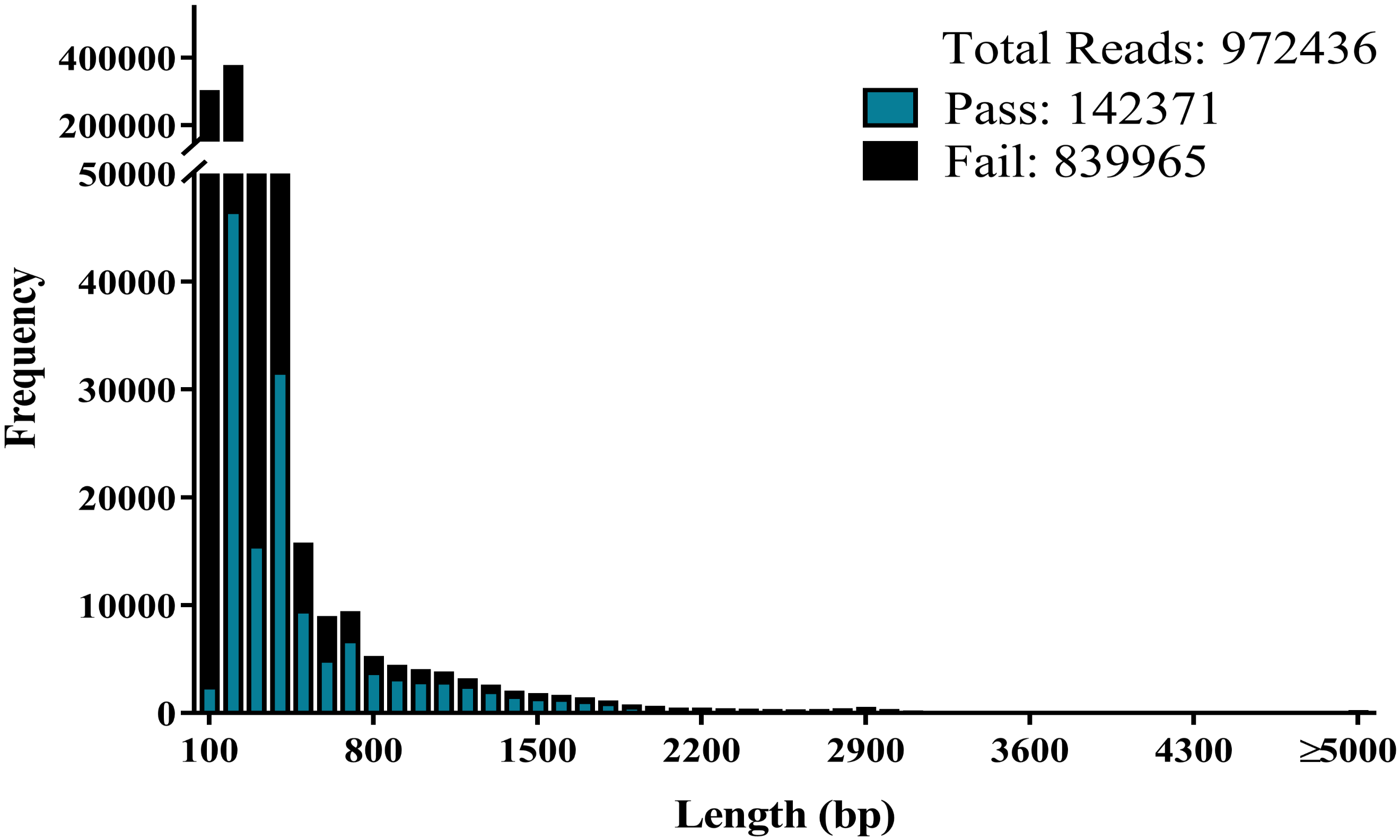

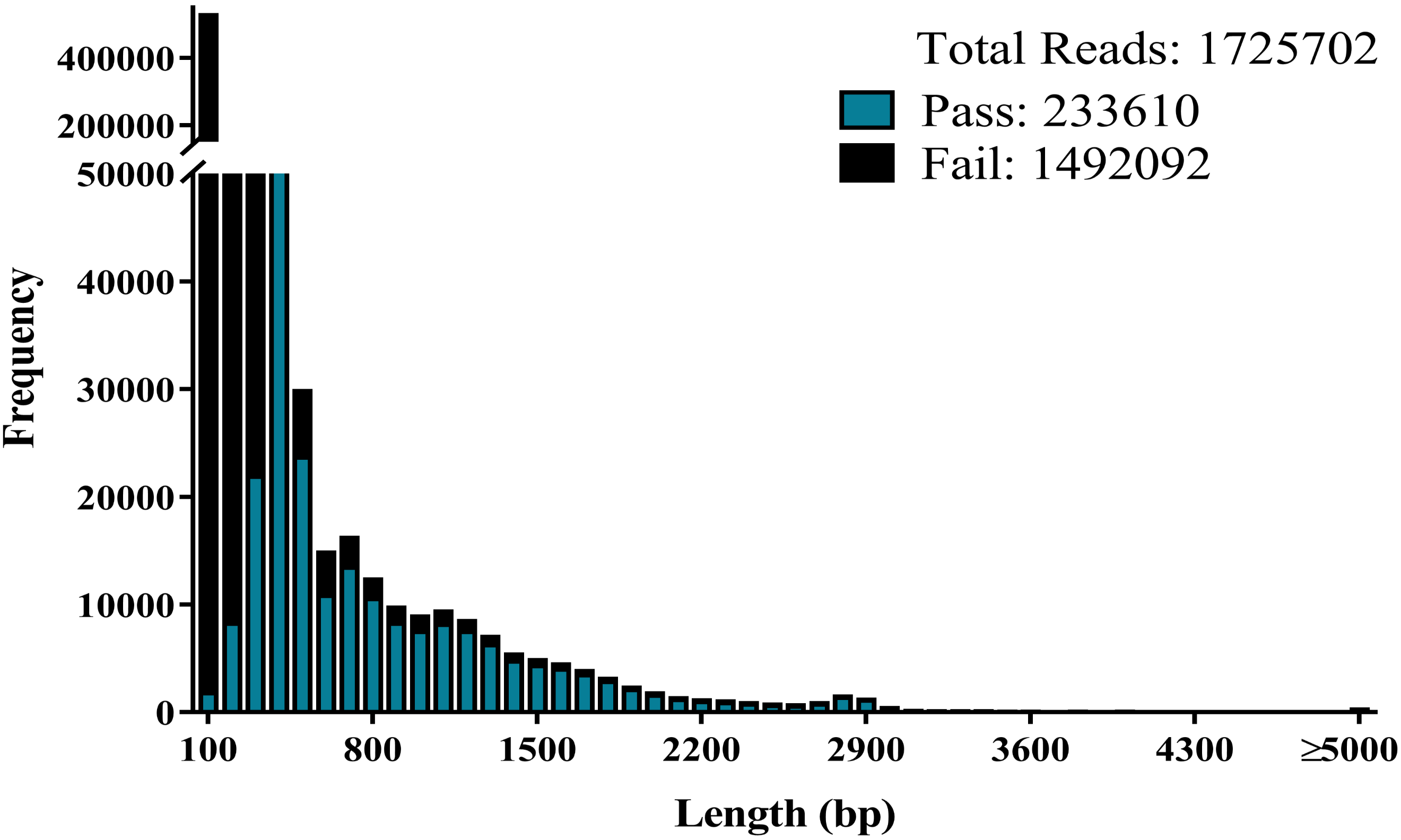

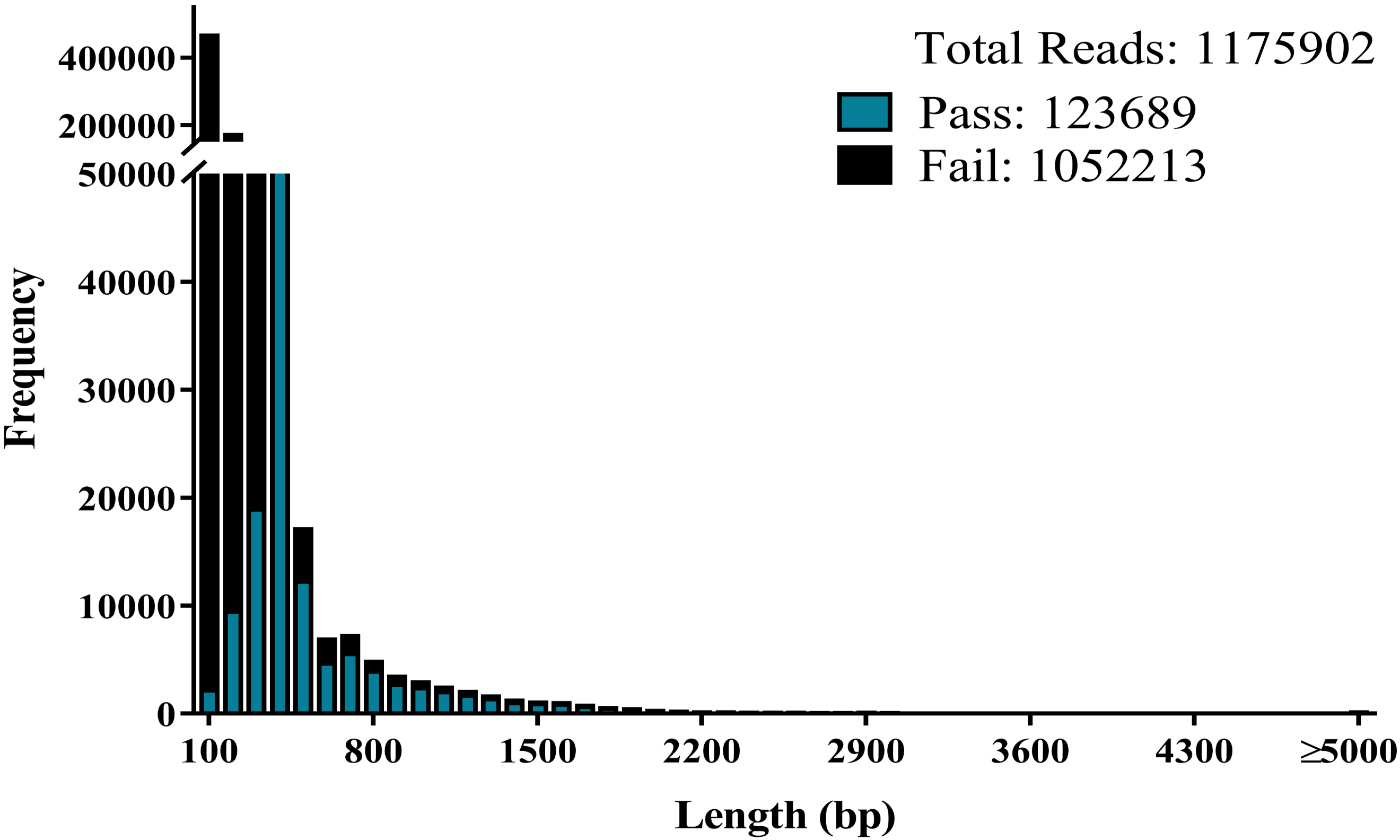


**Figure S3:** Size distribution of reads for sequenced samples. Isolates sequenced by the MinION platform include: (**A**) 1_GR_13 DNA, (**B**) 2_GR_12 DNA, (**C**) 16_GR_13 DNA, (**D**) 20_GR_12, (**E**) 1_GR_13 RNA, (**F**) 2_GR_12 RNA, (**G**) 16_GR_13 RNA, (**H**) 20_GR_12 RNA. Reads were base-called using Albacore 2.1.1 for DNA and 2.2.7 for RNA.

**Figure S4:** Detection of resistance genes via the real-time emulation analysis using DNA or direct RNA MinION sequencing. (**A**) 1_GR_13, (**B**) 2_GR_12, (**C**) 16_GR_13 and (**D**) 20_GR_12. The y-axis displays the resistance genes where an (/) indicates reads detecting more than one gene, (^#^) is a family of genes (>3) and **bold** displays a gene identified in the final assembly. An asterisk (*) on bars highlights the lack of detection in direct RNA sequencing. Albacore base-calling was used for all datasets. X-axis shows the amount of data (Mb) required for a resistance gene to be confidently called via the emulation.


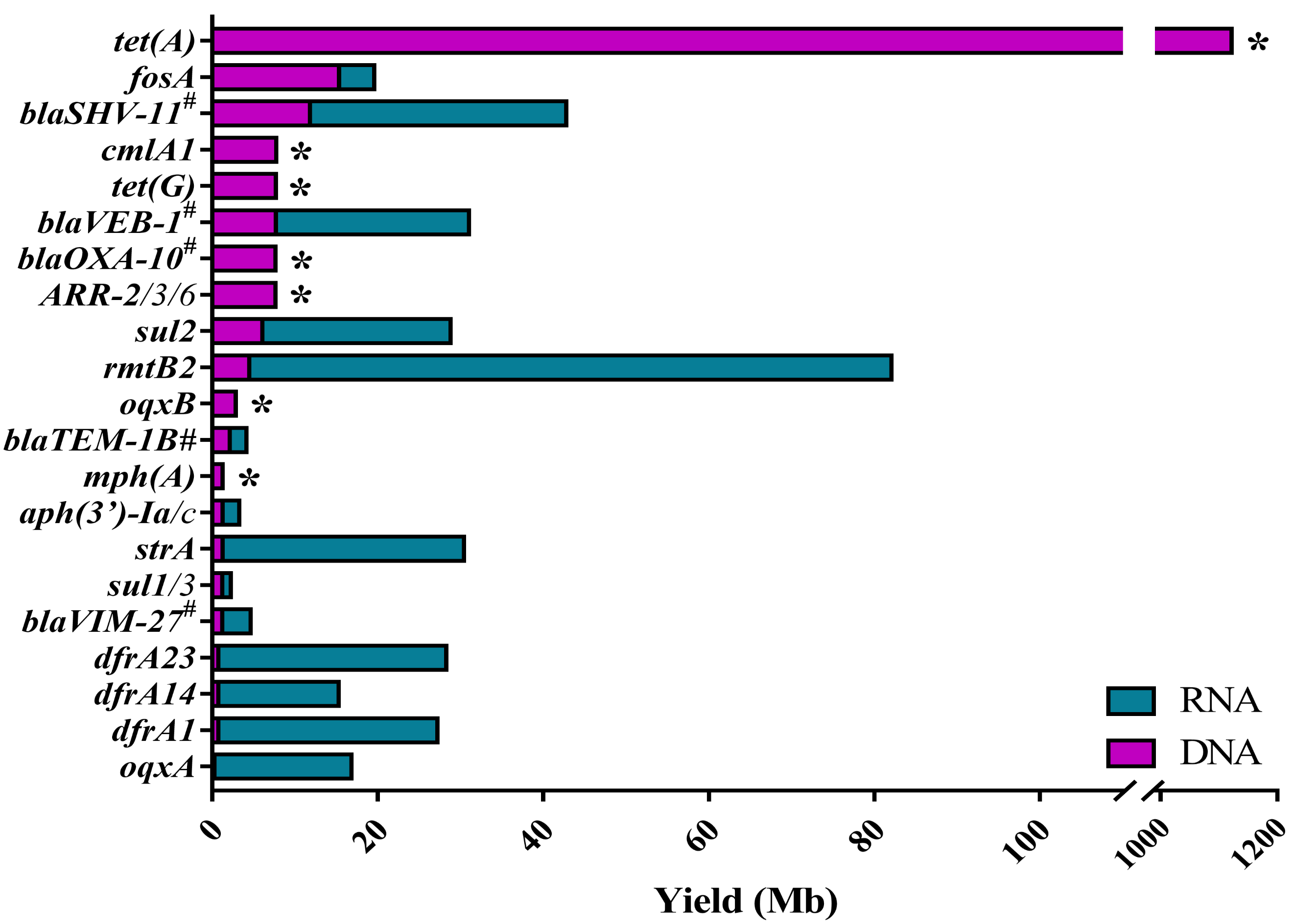

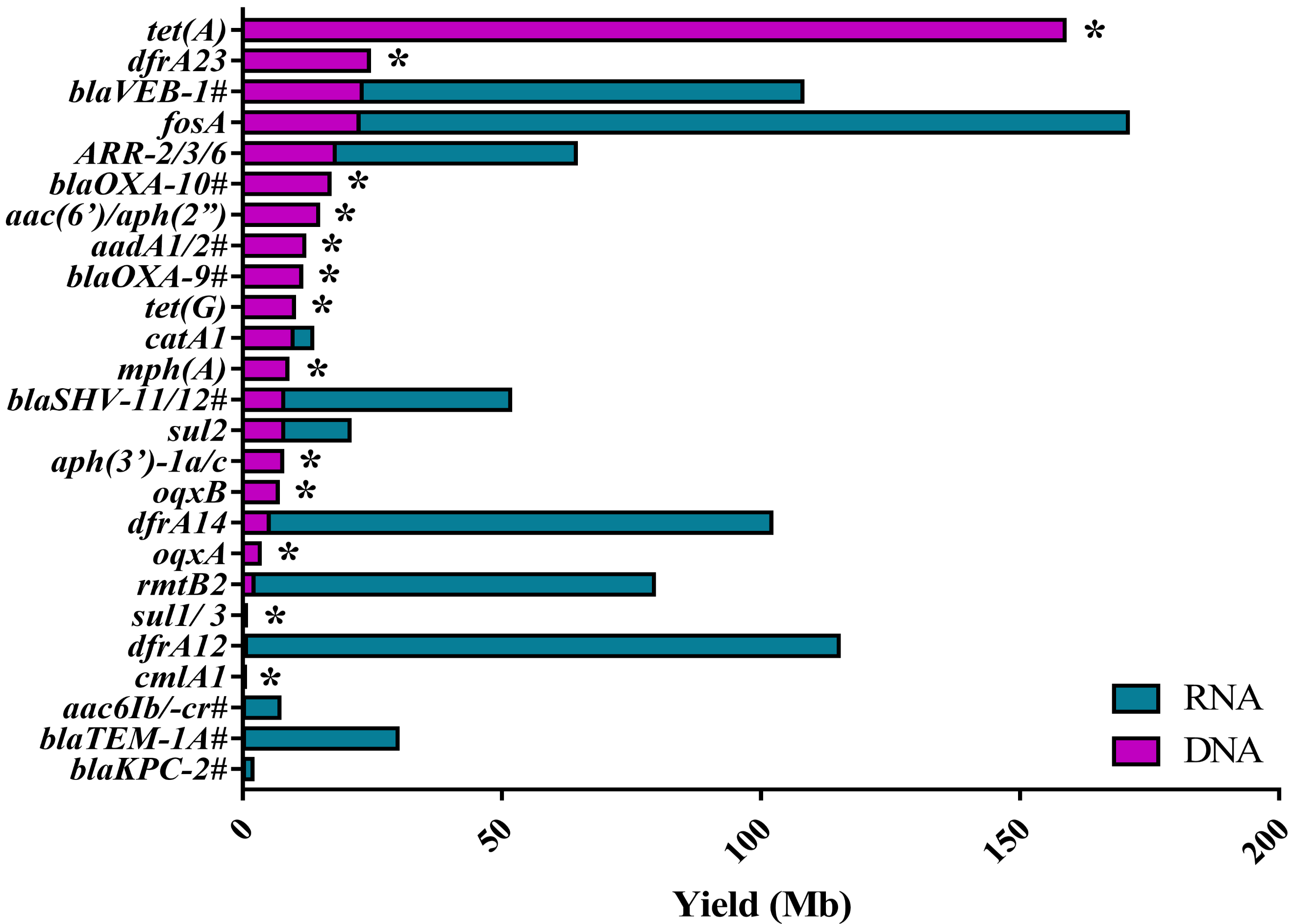

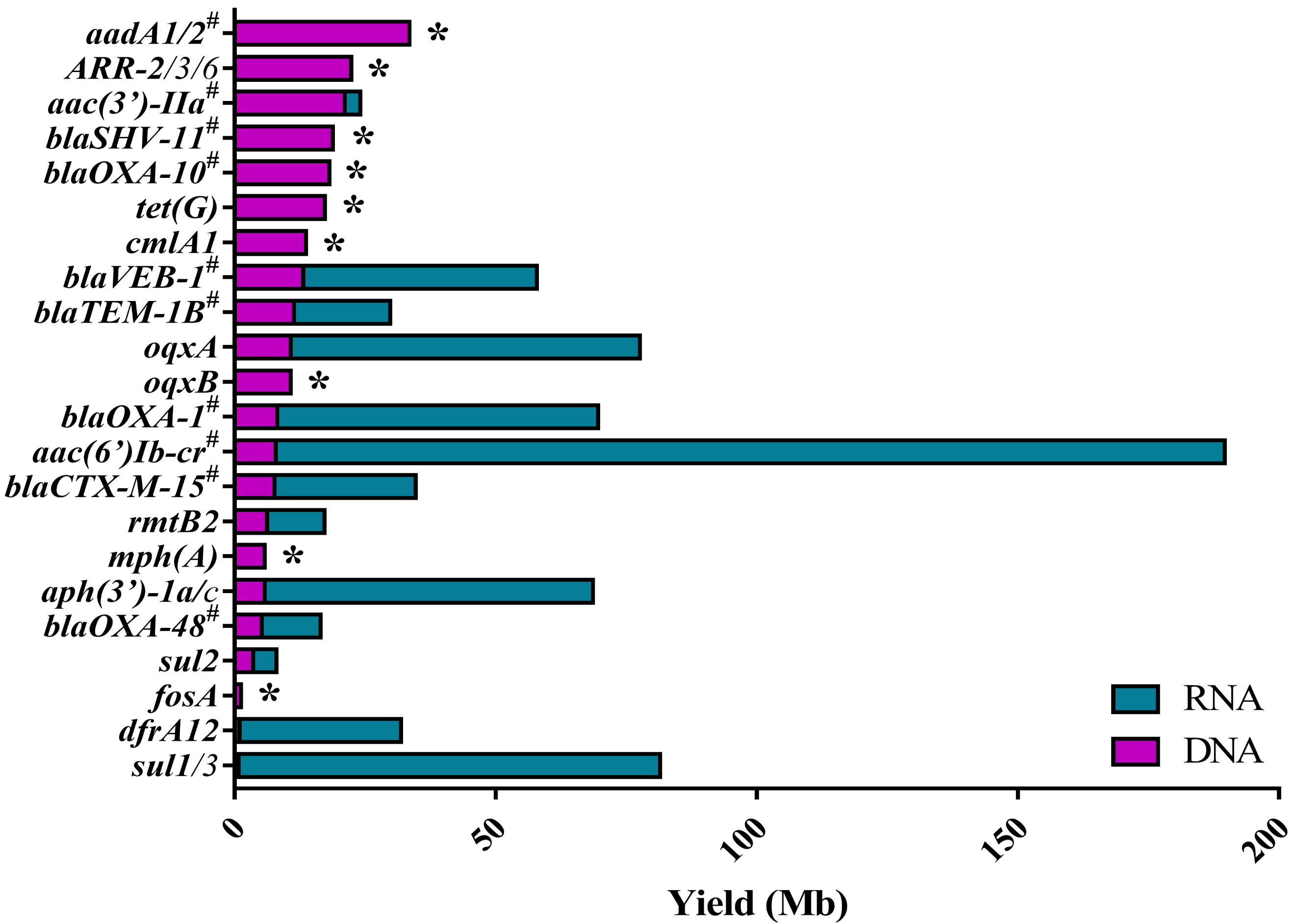

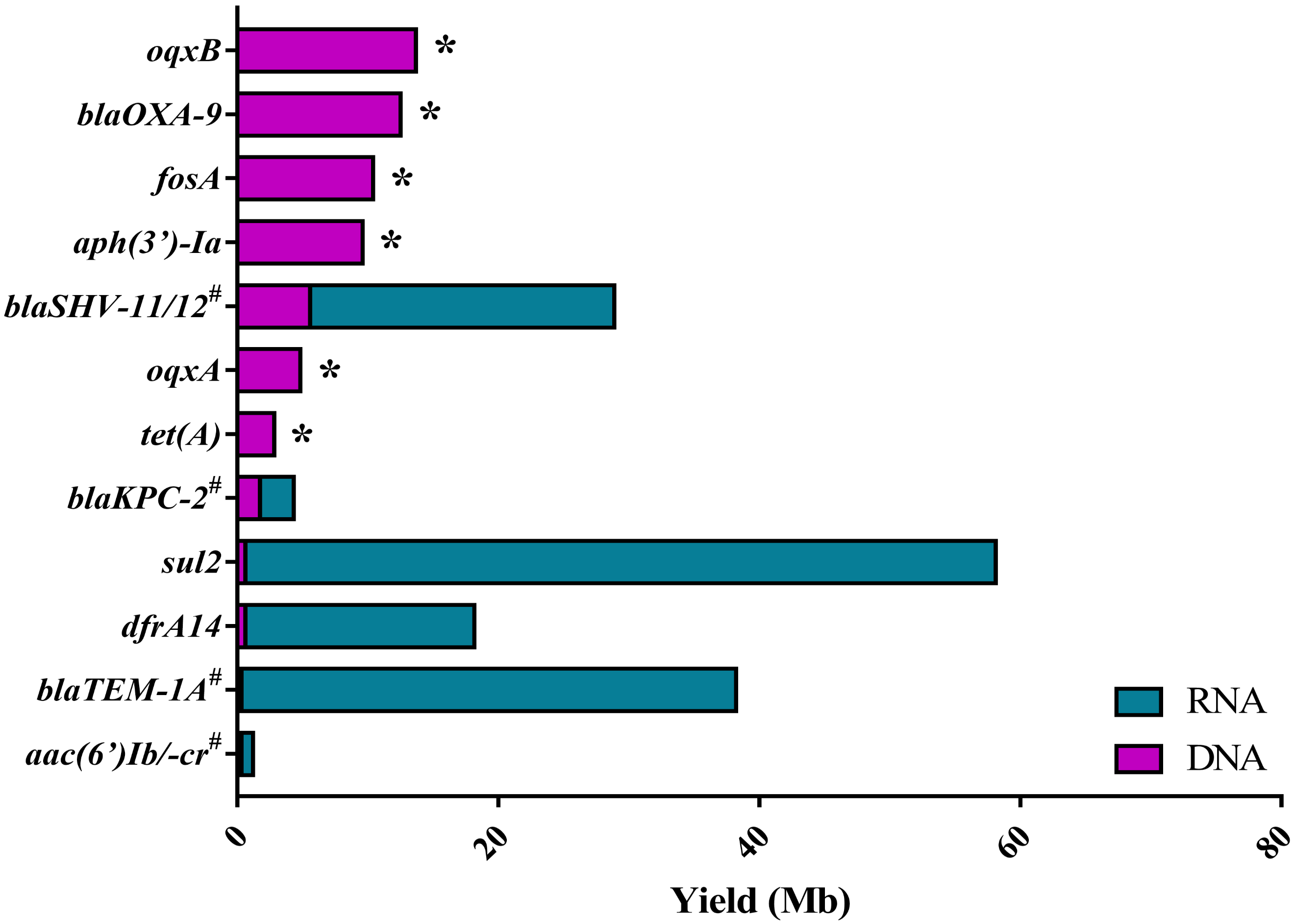


**A**

**B**

**C**

**D**

**
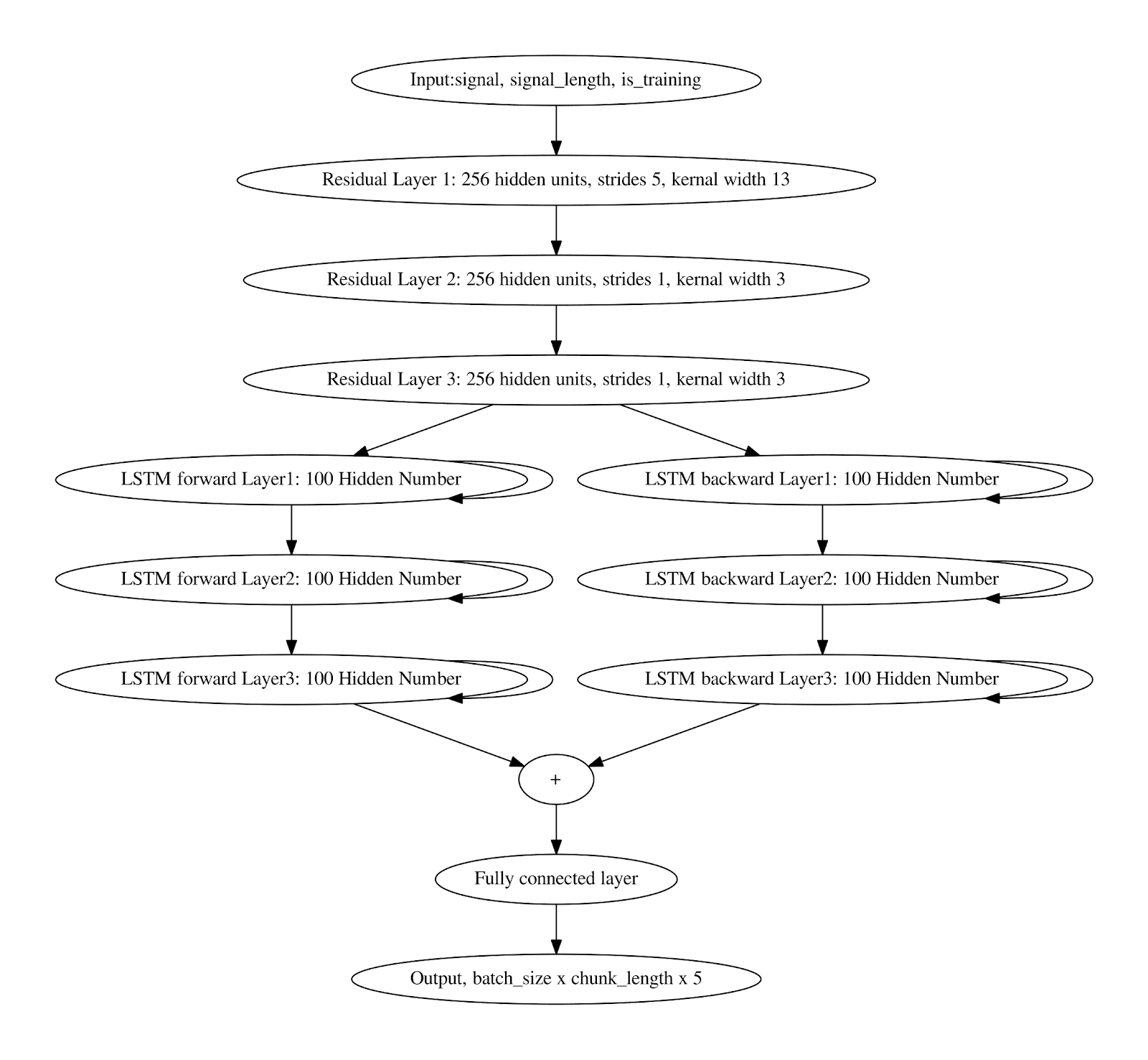
**

**Figure S5:** Diagram of RNA neural network underlying Chiron RNA model, consisting of 3 residual layers, and 3 LSTM layers. The model was trained as follows: First we used Albacore 2.2.7 to base-call raw data and the nanopolish poly(A) segmentation tool to remove the signal corresponding to the poly(A) tail prior to dataset labelling. We then aligned base-called data to reference genomes with BWA-MEM and then Tombo-1.4 to re-squiggle the raw signal data to the reference genome DNA (i.e. align the signal with the underlying bases which generated the signal). Chiron was trained using a chunk length of 2000, 80000 training steps, and an initial learning rate of 0.004, with the following command:

python chiron/chiron_rcnn_train.py -i $INPUT_DIR -f $INPUT_FILES -v 20_GR_12_validation.tfrecords -o $OUTPUT --model $MODEL_NAME --configure $WORK_DIR/Chiron/sample_config/model_rna3.json --train_cache $CACHE_DIR/2000l.hdf5 --valid_cache $CACHE_DIR/2000l_20_GR_12_valid.hdf5 -s 2000 -b 50 -t 4e-3 -x 80000 --resample_after_epoch 1 --threads 8


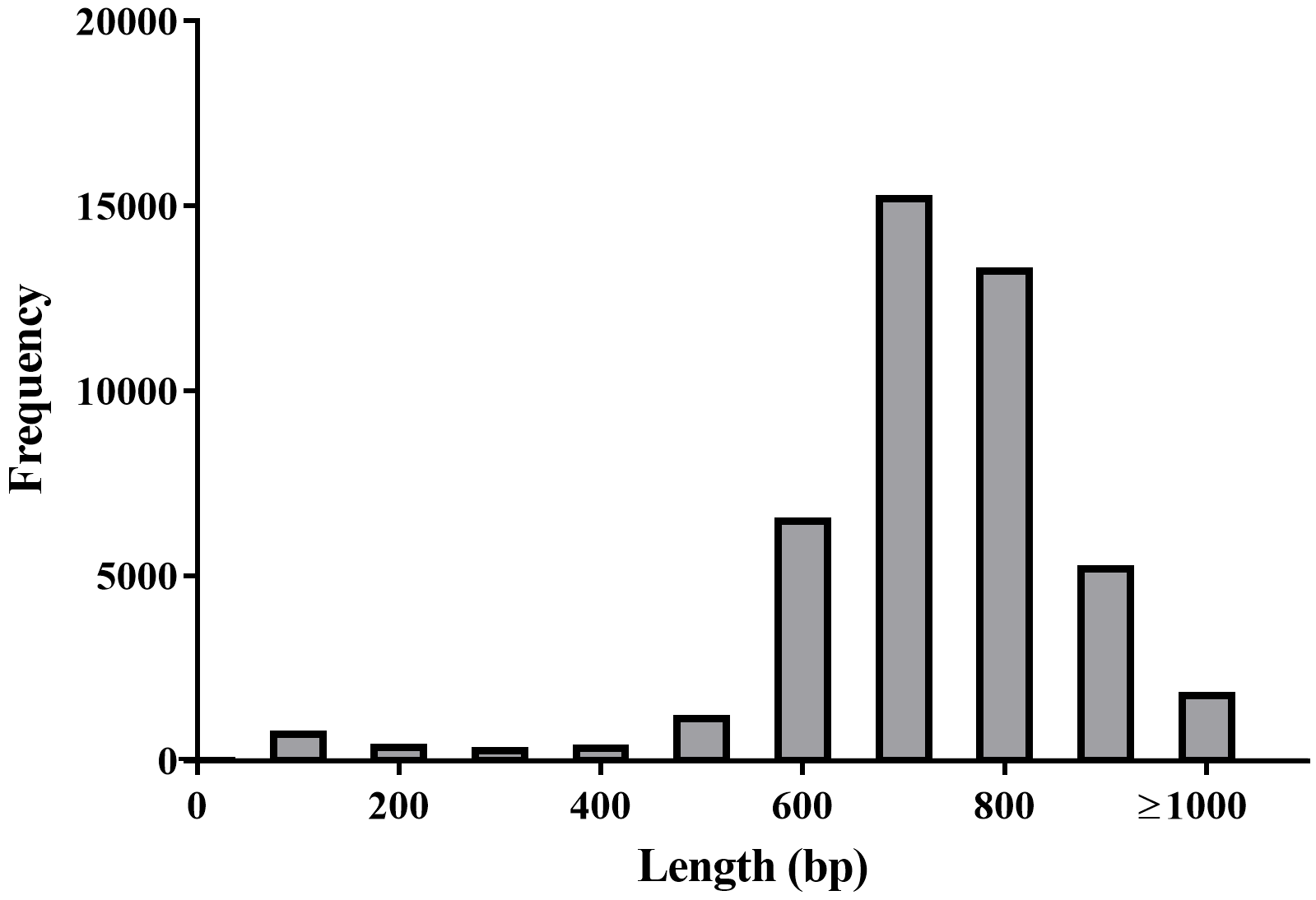


**A**


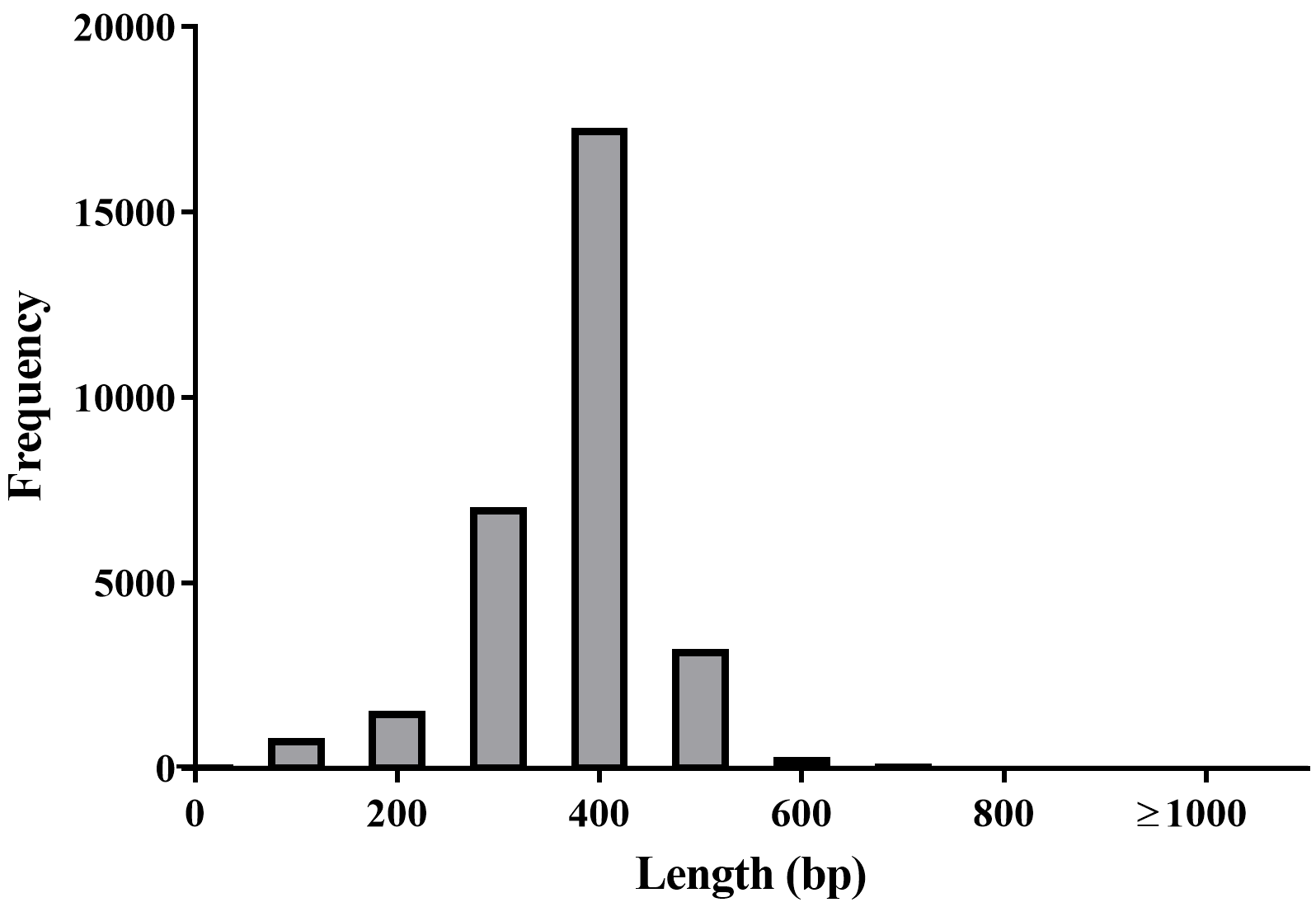


**B**


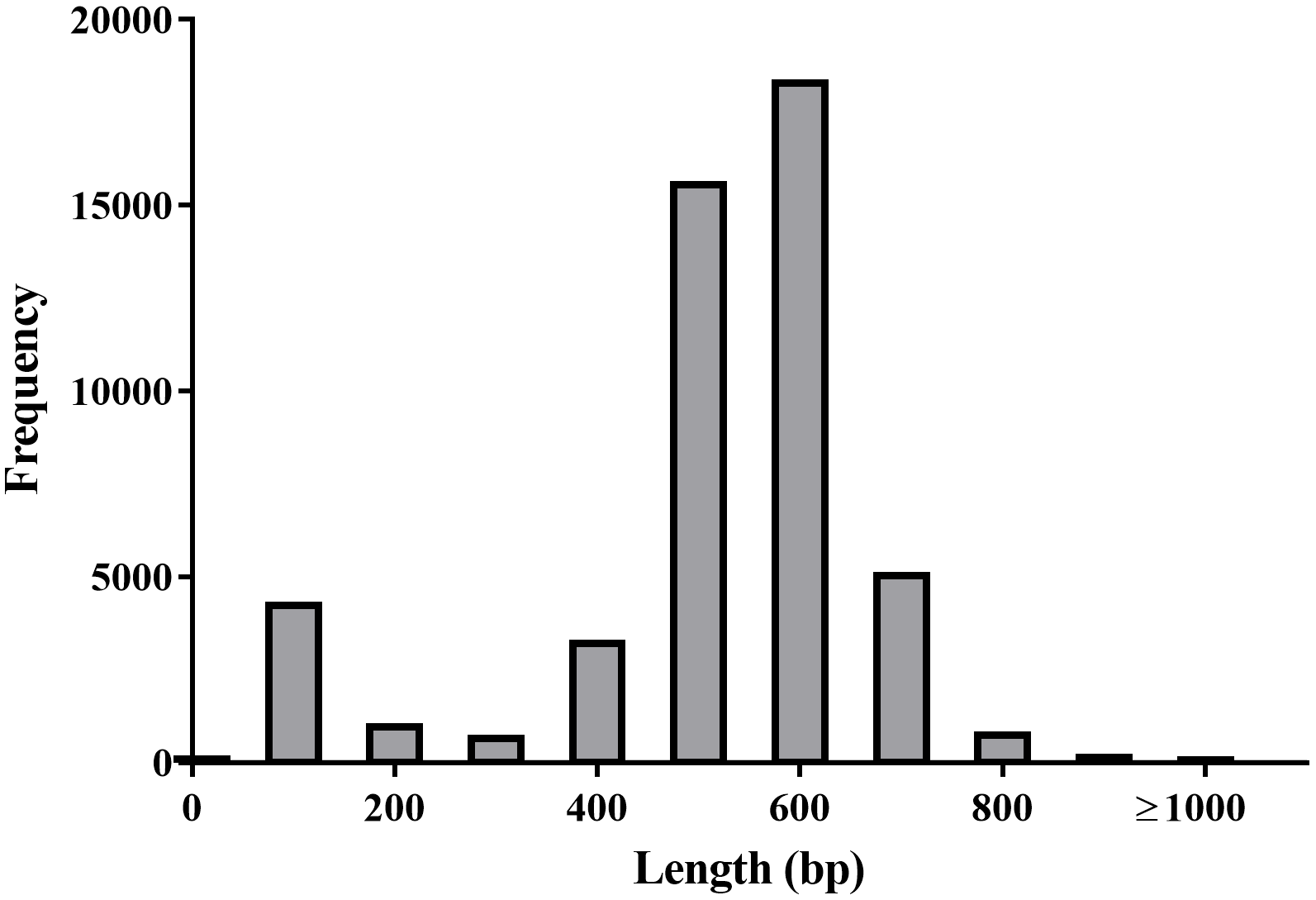


**C**


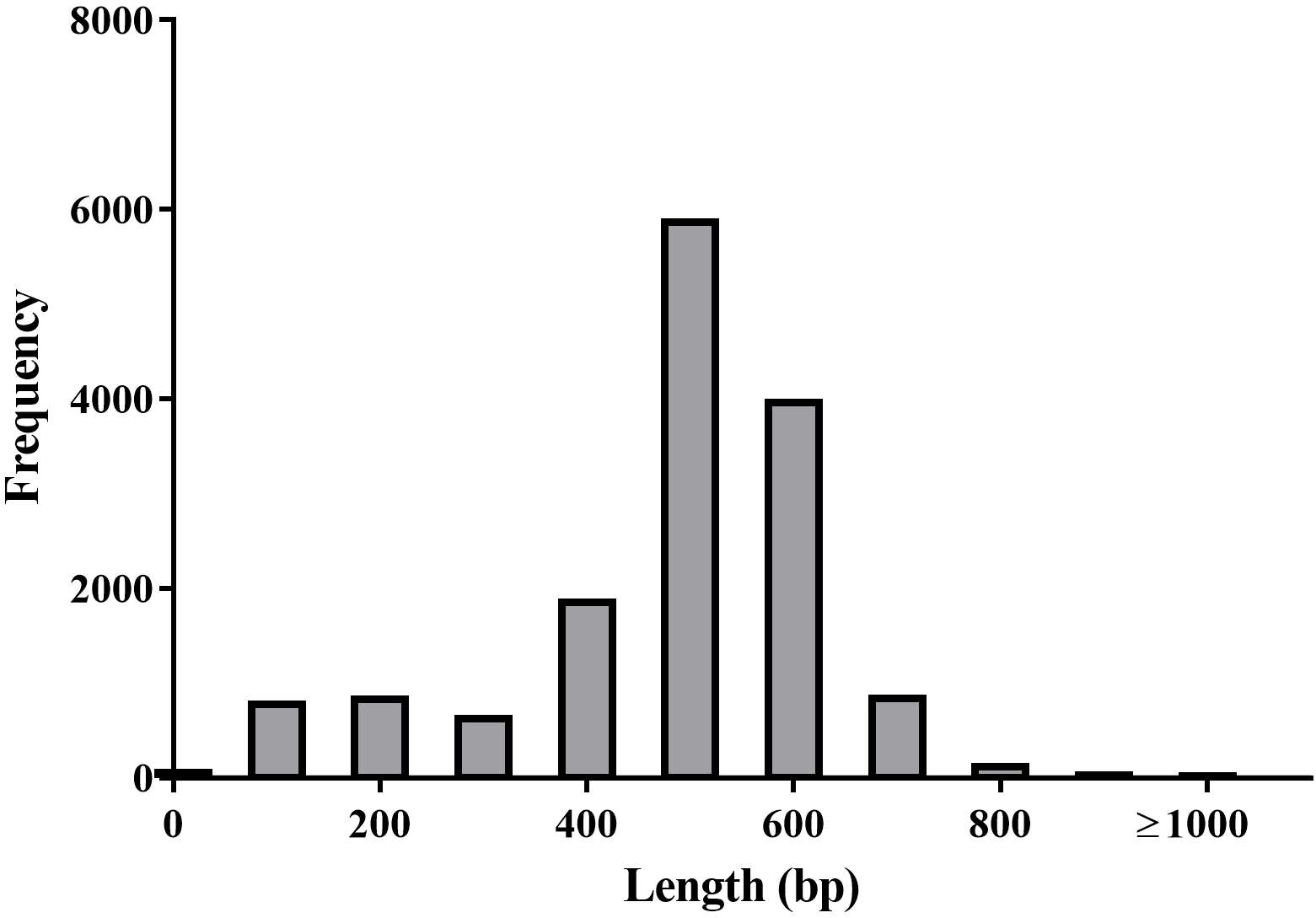


**D**

**Figure S6:** Size distribution of poly(A) tails determined via direct RNA MinION sequencing. Samples include: 1_GR_13 (**A**), 2_GR_12 (**B**), 16_GR_13 (**C**) and 20_GR_12 (**D**). Poly(A) length was determined using Nanopolish (https://github.com/jts/nanopolish).


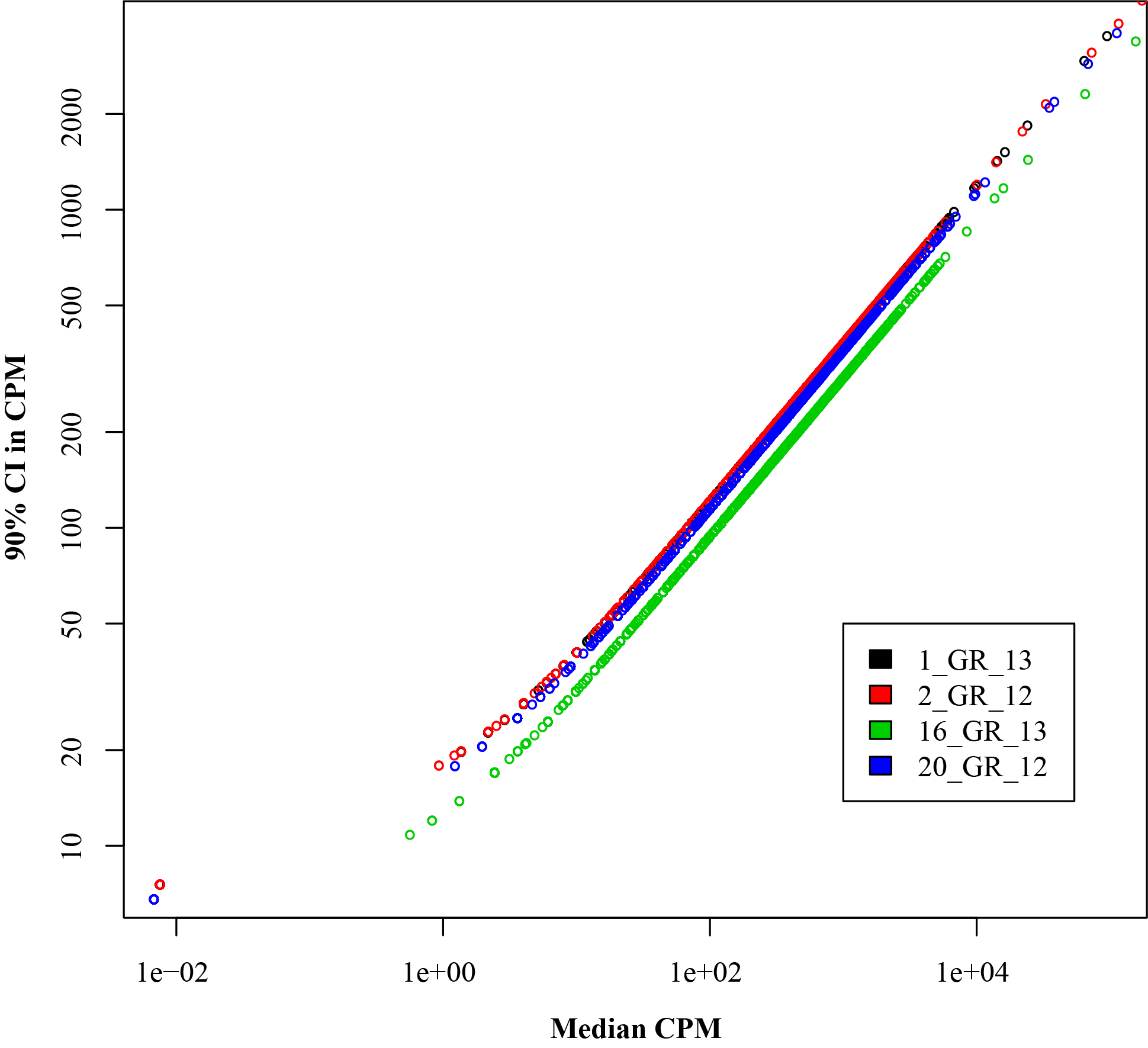


**Figure S7:** Estimated 90% confidence intervals as a function of estimated CPM for direct RNA sequencing data for 4 samples. Estimates derived from a beta-distribution with shape parameters alpha, beta equal to the 0.1 + number of reads mapping to a given gene and 0.1 + number of reads mapping to all other genes.


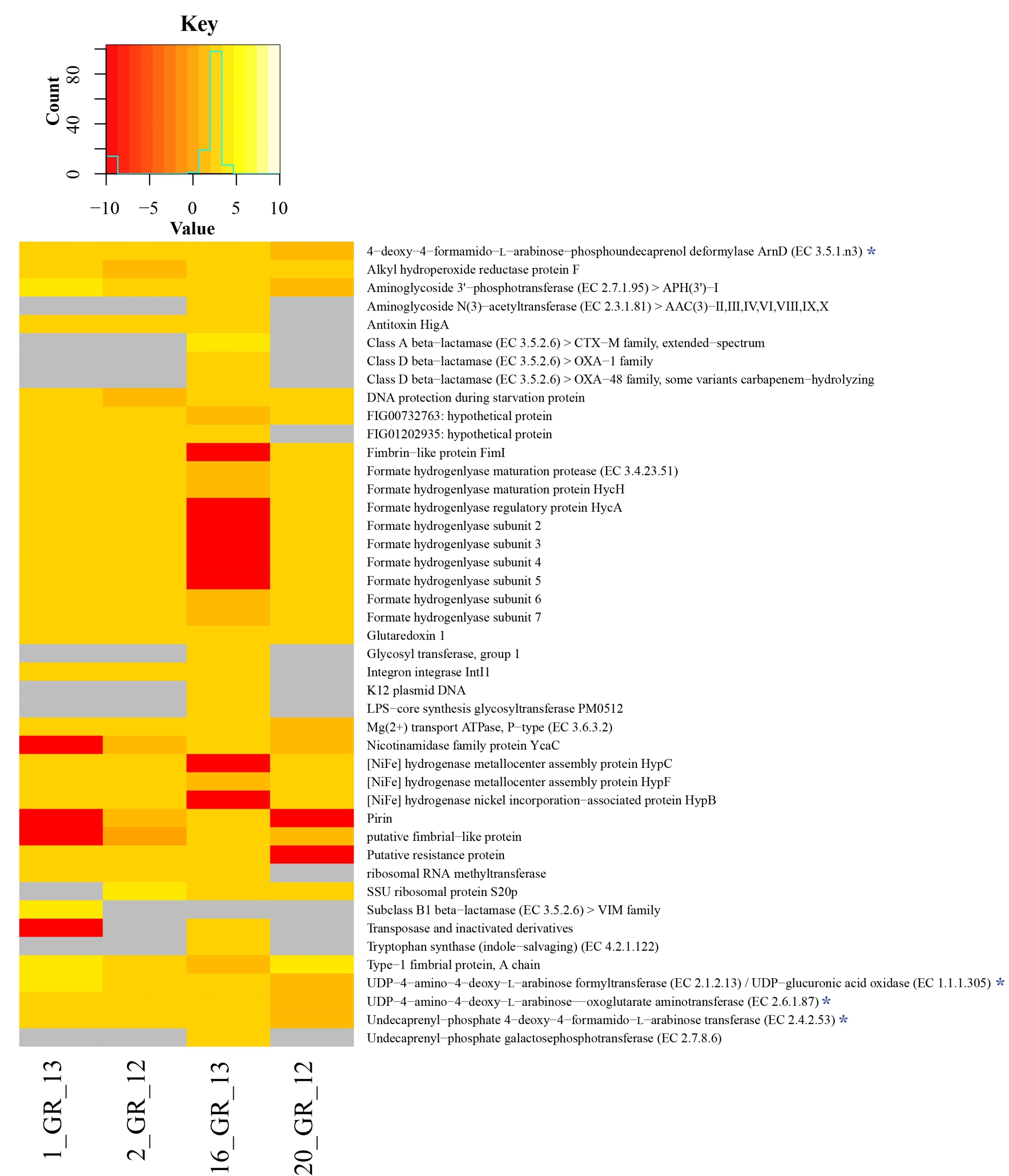


**Figure S8:** Heatmap depicting the top differentially expressed genes across the four *K. pneumoniae* isolates. Expression determined via ONT direct RNA sequencing. Key indicates whether these genes were over-expressed (yellow) or under-expressed (red). Grey indicates the absence of this gene in the isolate. An asterisks (*) indicates pathways associated with polymyxin resistance and values represent log10(cpm).

**References**

1. Clinical and Laboratory Standards Institute. Performance Standards for Antimicrobial Susceptibility Testing - Twenty-Eighth Edition: M100. 2018, CLSI, Wayne, PA, USA.
2. The European Committee on Antimicrobial Susceptibility Testing. Breakpoint tables for interpretation of MICs and zone diameters. Version 8.1. 2018, http://www.eucast.org.
3. Jayol A, Poirel L, Brink A, et al. Resistance to colistin associated with a single amino acid change in protein *pmrB* among *Klebsiella pneumoniae* isolates of worldwide origin. *Antimicrob Agents Chemother.* 2014;58:4762-6.
4. Cheng YH, Lin TL, Pan YJ, et al. Colistin resistance mechanisms in *Klebsiella pneumoniae* strains from Taiwan. *Antimicrob Agents Chemother.* 2015;59:2909-13.
5. Cannatelli A, D’Andrea MM, Giani T, et al. *In vivo* emergence of colistin resistance in *Klebsiella pneumoniae* producing KPC-type carbapenemase mediated by insertional inactivation of the PhoQ/PhoP *mgrB* regulator. *Antimicrob Agents Chemother.* 2013;57:5521-6.
6. Li H. Minimap2: pairwise alignment for nucleotide sequences. *Bioinformatics*. 2018;34:3094-100.
