# Supplementary material for: Evaluating the genome and resistome of extensively drug-resistant Klebsiella pneumoniae using native DNA and RNA Nanopore sequencing
Source: Gigascience. 2020 Feb 4;9(2):giaa002. doi: 10.1093/gigascience/giaa002 (PMC6998412; doi:10.1093/gigascience/giaa002)
Supplement: giaa002_Response_to_Reviewer_Comments_Original_Submission [file giaa002_response_to_reviewer_comments_original_submission.pdf]

# Author's Response To Reviewer Comments

Close

“Evaluating the Genome and Resistome of Extensively Drug-Resistant Klebsiella pneumoniae using Native DNA and RNA Nanopore Sequencing”
GIGA-D-19-00200
Response to Reviewers

Dear Dr. Scott Edmunds,

We thank the reviewers for the opportunity to revise this manuscript (GIGA-D-19-00200). Their comments have helped us significantly strengthen the work. We have now provided additional information including rationale for using direct RNA sequencing and particular analysis methodologies. Figures have also been modified to aid with the interpretation of data. To highlight the adjustments completed, we have also uploaded a mark-up version of the manuscript. Please find below a point-by-point response to the reviewers’ comments.

Reviewer reports:

Reviewer #1:
In the manuscript "Evaluating the Genome and Resistome of Extensively Drug-Resistant Klebsiella pneumoniae using Native DNA and RNA Nanopore Sequencing" by Pitt et al., the authors describe datasets generated from multiple sequencing modalities of antibiotic-resistant clinical isolates, and discuss the potential of this technology for rapid detection of AMR. Although these methods and sequencing characterization and analysis are of importance to the field, there are several issues which remain to be addressed.

Specific points:
It would be useful to better establish the rationale for why direct detection of RNA transcripts matters, and what additional information direct RNA sequencing gets you that rapid cDNA conversion and sequencing can't. Perhaps the largest issue is - "Why dRNA-seq?" There doesn't seem to be an obvious benefit, given the poor time to detection compared to just DNA sequencing. Expression levels are useful, but could be determined from Illumina sequencing. Without splicing there are no isoforms to contend with, and the error rate adds difficulty in interpretation and determination of primary protein sequence. Additionally, most clinical bacterial characterization work doesn't use RNA-seq, and addressing the problems clearly (i.e. rRNA depletion, RNA instability) should be done at the outset.
Response: We have now provided additional information to highlight the benefits of using direct RNA sequencing in the introduction and discussion. The time to detect antibiotic resistance using direct RNA sequencing was slower compared to DNA, however, this is only the first generation of the technology. The latest kit, SQK-RNA002, has shown advancements in data generation which unfortunately was not available during the time of this study. “Our findings show that the slower time-to-detection of resistance genes in direct RNA sequencing was due to both the level of expression as well as the slower translocation speed, and hence using cDNA would only partially overcome this limitation.” (Discussion: Line 396, also refer to Supplementary Figure S4). “Furthermore, library preparation time is halved for direct RNA sequencing due to the absence of cDNA synthesis” (Introduction: Line 57). Indeed, expression levels can be determined via Illumina sequencing, however, in the context of a diagnostic tool, Illumina platforms require the completion of the sequencing run (~48 hours) to output data and analysis to be performed. Nanopore technologies can output data as soon as it is generated to enable real-time analysis. Although bacteria lack splicing, long read sequencing has the potential to detect operon sites where several transcripts are co-expressed (refer to Line 59 and 417). Due to difficulties extracting RNA from these strains and downstream processing for sequencing, these transcripts were short and not enough data was generated to confidently detect operon sites (Supplementary Figure S3). Furthermore, native RNA sequencing has the potential to detect RNA modifications associated with antibiotic resistance which are removed when converted to cDNA and is unique to this technology (Introduction: Line 55). Although RNA is unstable and requires several additional processing steps compared to DNA, advancements on this part could be made in the future and hence, the potential for

this to be used to detect antibiotic resistance was explored. We have now made note of the limitations associated with RNA sequencing in the clinic (Discussion: Line 368). Additionally, RNA has the potential to determine the functionality of a resistance genes as the presence of these genes does not necessarily mean they confer resistance (Discussion: Line 369).

Under the "DNA extractions and HMW DNA isolation methods section", this section should be rewritten for clarity - it was confusing to determine which isolations worked and which didn't, and why. It's still important to include details of why protocol modifications were made, but if these could be incorporated into methods better that would aid in understanding.
Response: This section has now been rewritten ("High molecular weight DNA isolation", page 4). Several modifications were implemented primarily due to difficulties lysing these highly antibiotic-resistant K. pneumoniae strains potentially due to a thickened capsule wall. This resulted in capsule contamination (carbohydrate) as determined via Nanodrop (Line 96). This was very cumbersome for isolate 2_GR_12 which was noted to have an increased carbohydrate contamination potentially due to the capsule and required a further purification step (Line 97).

Under "real-time resistome detection emulation" as well as "assembly of genomes" sections, it would be helpful to include a rationale on why certain software tools were chosen over others, given you tried many options. For example, why was BWA-MEM chosen over minimap2?
Response: In light of the vast amount of software tools available, we selected the four most commonly used tools for bacterial assembly. These incorporated both hybrid assemblers (Unicycler, npScarf) and the remaining two using only Nanopore reads (Canu, Minimap2/ Miniasm/ Racon). We trialed analysis using minimap2 initially, however, a lower alignment rate was observed potentially due to the majority of reads being less than 1000 bp (Supplementary Figure S3). This has now been mentioned in the supplementary section: Supplementary Table S6 and noted in the main text (Line 148) which also notes adjusted parameters used for BWA-MEM when using ONT reads.

How were you able to distinguish multiple copies of resistance genes from duplicated misassemblies?
Response: Both the fragment distribution (Supplementary Figure S1) and the read-length distribution (Supplementary Figure S3 A-D) indicate substantial number of reads of length greater than 10kb. The vast majority of bacterial repeats are shorter than 10kb, meaning that we are able to correctly place these repeats in the assembly. Furthermore, these long reads were able to span the duplicated resistance gene regions and correctly assemble these plasmids.

Would it actually be faster to detect with cDNA sequencing, given faster motor protein translocation rate and likely higher copy number of transcripts of interest? It would be useful to include thoughts on this in the discussion.
Response: While the sequencing speed of cDNA is currently faster than direct RNA (450 bases/second vs 70 bases per second) the library preparation for direct RNA is much quicker (105 minutes vs 270 minutes). Moreover, it is anticipated that future direct RNA sequencing kits will run at the same translocation speed as cDNA. We considered the translocation speed impeding on the detection method, hence, why we included an analysis total yield required to detect resistance genes as well as time to call the resistance genes (Line 266, Supplementary Figure S4). We have now added an additional sentence in the discussion: "Our findings show that the slower time-to-detection of resistance genes in direct RNA sequencing was due to both the level of expression as well as the slower translocation speed, and hence using cDNA would only partially overcome this limitation." (Line 396).

You say "Nanopore DNA sequencing currently has an accuracy ranging from 80 to 90%, which limits its ability to detect genomic variations", but there are post-processing tools available to increase accuracy and ability to detect SNVs - this should be included in the discussion.
Response: Agreed, there are tools to improve the accuracy which we have now made note of in the discussion: "However, software tools such as Nanopolish (https://github.com/jts/nanopolish) and Tombo (https://github.com/nanoporetech/tombo) (similarly used to re-train Chiron v0.5 for direct RNA sequencing data) have the potential to correct these reads and would be helpful to integrate to increase the accuracy of detecting resistance genes." (Line 359).

Further the detection of SNV mutations and indels is critical with respect to the detection of chromosomal mutations in these samples. Additional consideration of methylation signatures is crucial, as they can cause systematic error (PMID: 30373801) if not corrected.
Response: We have now noted the influence of DNA modifications on the accuracy of Nanopore sequencing and included this publication. "We utilised native DNA sequencing in this study which retains epigenetic modifications such methylation which can hinder the accuracy of reads and subsequent calling

of antibiotic resistance [58].” (Line 362).

"All isolates exhibited low levels of expression for fosfomycin, macrolide and tetracycline resistance, despite exhibiting phenotypic resistance to fosfomycin and tetracycline", but are high levels of expression essential for phenotypic resistance? Are these low levels surprising? It would be helpful to link to papers discussing this.
Response: Additional information has now been included to identify why low expression of particular genes was observed. Limited literature is available on these specific genes in K. pneumoniae with transcriptional and antimicrobial susceptibility testing. We have included the following sentence regarding fosfomycin resistance facilitated via the fosA gene: “Noteably, Klontz et al identified that chromosomally integrated FosA, similarly observed in our study, from K. pneumoniae harboured a higher catalytic efficiency. A higher catalytic efficiency may reason why our strains only require a low abundance of expression and still retain fosfomycin resistance” (Line 382). Low levels of expression for tetracycline are not surprising as this resistance is well characterized and found to be inducible (antibiotic exposure is required for expression of genes). This has been reworded: “Genes tet(A) and tet(G) encode efflux pumps which, in the absence of tetracycline, are lowly expressed and the lack of antibiotic supplementation in this study confirms this observation [61]. Detecting inducible resistance (antibiotic exposure required for gene expression) such as tetracycline resistance highlights one of the advantages of investigating the transcriptome.” (Line 384)

Figure 5 - instead of switching back and forth between panels A and B, a scatterplot comparing the two directly like Fig 3 would be more useful.
Response: This figure has now been amended with the data on a single graph.

Why do you think only 23% RNA reads aligned? Did you try to identify the unaligned reads (like sort out contamination, noise)? It would be beneficial to include at least a blast/centrifuge style analysis trying to determine the source of the unaligned reads. Additionally, a k-mer analysis of the unaligned reads could help determine their origin.
Response: We identified that various failed reads were <10 bp (Supplementary Figure S3) which were filtered before alignment with BWA-MEM (k -11, seed length of 11 bp). Preliminary BLASTn analysis of unmapped reads identified a bacterial origin. The primary issue with the direct RNA sequencing data is the base-calling. When adapting Chiron v0.5 for this data, squiggle plots (raw nanopore data) identified insufficient trimming of the artificial poly(A). Furthermore, RNA modifications in bacteria remain largely unknown and this has the potential to interfere with the raw nanopore current change and subsequent base-calling. This has now been included in the discussion: “Limitations were observed when base-calling bacterial direct RNA sequencing and may be attributed to trimming the long artificial poly(A) tail and interference of RNA modifications.” (Line 391).

How much of the poor alignment is due to the method of preparation (i.e. polyA tailing, etc.)? Did the authors perform optimization of the extraction and library prep for bacterial RNA? What about using an alternative tail and RNA adaptor?
Response: We trialed phenol/ chloroform RNA extractions however, this process was lengthy and resulted in a low yield of RNA and increased impurities. The PureLink RNA Mini Kit protocol is relatively quick (<30 mins/ sample). We attempted an on-column DNase treatment during this protocol but the best DNA depletion was observed using TURBO DNase which doesn’t work on column (requires 37°C incubation). Our optimized RNA extraction resulted in Bioanalyzer RNA integrity scores of ≥8.5 which has now been included in Line 116 (RIN scale 0-10, 10 is no degradation using 16S and 23S pecks as reference). We considered altering the library preparation including using an adapter similar to Smith et al (reference 26) which recognizes the Shine-Dalgarno sequence, however, there are deviations in this sequence and multi antisense adapters would be required so all transcripts are sequenced. Hence, the poly(A) tailing kit was more feasible as it will tag all 3’transcripts which allows for only the native RNA strand to be sequenced. Unfortunately, we were unaware of the efficiency of the polymerase until post sequencing analysis was performed (Supplementary Figure S6), hence, a shorter incubation can be implemented for future studies.

Viral direct RNA seq has been done (PMID: 30765700 and 30258076 for example) - it would be good to cite these or related papers.
Response: The updated publication of PMID: 30765700 rather than the preprint has been included in the references and PMID: 30258076 was originally incorporated in the introduction as reference 24 (refer to Line 54 for references referring to viral direct RNA sequencing). To our knowledge, all the publications on direct RNA sequencing are in the references.

Some minor points:
"This research also established a methodology and analysis for bacterial direct RNA sequencing." is repeated in the conclusions.
Response: This duplicated sentence has now been removed from the conclusions section.

Figure 2 colorblocking is a little confusing - could be more straightforward to break up the figure into separate panels per strain contig, for example with a ggplot facet_grid.
Response: Figure 2 has now been modified so genes belonging to particular contigs are easier to identify. This included adjusting the transparency of the colorblocking and splitting the x-axis similar to the ggplot facet_grid format.

Reviewer #2:
This manuscript presents a rapid resistance-gene discovery experiment, using genome sequencing and assembly to identify potentially-active genes, combined with differential expression to determine drug-free resistome activity. This manuscript is differentiated from most other direct-RNA and cDNA nanopore research, in that it is the *expression* rather than the *structure* of the genes is evaluated here. Bearing in mind that I cannot comment much on the biology side of things, I consider this manuscript to be a reasonable presentation of the experimental work that has been described, and recommend that it be accepted pending minor changes to figures, and clarification of multi-mapping results. I would like to thank the authors for making their Nanopore sequence data public prior to review submission; it demonstrates a good open research ethic.
My specific comments regarding the manuscript follow:
** Text **
L133: This references a fairly old version of Canu (i.e. v1.5), which seems a bit strange given that Guppy v3.0.3 is also mentioned (L260). I note that Canu v1.8 was released before Guppy v3.0.3, and would be interested to know why this version of Canu was chosen.
Response: Genome assemblies were conducted initially in this study and the transcriptomics at a later date. As we were able to complete the assemblies adequately using the hybrid assembler Unicycler and utilize Illumina reads to correct ONT sequencing errors, we did not run analysis on the most recent version of Canu. Furthermore, Guppy was integrated later as we had multiple issues with the base-calling of direct RNA sequencing and we hoped this update in the software would ameliorate this problem.

L144: I don't have an encyclopaedic knowledge of bwa-mem command-line options. It would be helpful to explain what the options mean. I'm particularly interested in why the default options were not appropriate, and what (if any) compensations were made for multi-mapped reads.
Response: This section has now been updated: "Similar parameters to the BWA-MEM ont2d function were used but seed length was reduced (-k 14) to compensate for shorter reads: -k 11 [minimum seed length, bp] -W20 [bandwidth] -r10 [gap extension penalty] -A1 [match score] -B1 [mismatch penalty] -O1 [Gap open penalty] -E1 [Gap extension penalty] -L0 [Clipping penalty]). Multi-mapping reads were removed via SAMtools (secondary alignment: flagged as 256)…" (Line 149).

L144: Why was minimap2 not used here? It was written by the same author as bwa-mem, but is specifically written to incorporate corrections to improve mapping for noisy Nanopore Direct RNA-seq [e.g. see https://github.com/lh3/minimap2#getting-started]
Response: Preliminary analysis using minimap2 showed fewer reads aligning to the reference (now noted in the legend of Supplementary Table S6). It has been noted by Li H (doi: 10.1093/bioinformatics/bty191) that BWA-MEM is more suited to short read data and has a slightly improved accuracy compared to minimap2. We've further noted the bias towards BWA-MEM in Line 148: "BWA-MEM was selected due to shorter transcripts being produced by bacteria (Supplementary Figure S3) and the lack of introns and alternative splicing."

L145: I notice from L198 that there are gene copies in the data, with potentially high identity. Is there a particular reason why reads were mapped to the genome, rather than to transcriptome that merges essentially-identical genes?
Response: As described in the "Real-time resistome detection emulation" section (line 127), the resistance gene detection was carried out by mapping to a database of resistance genes which was clustered based on 90% identity threshold. However, in the section "RNA alignment and expression profiling" (Line 146) we mapped reads to the genome. In this case, if a read mapped to multiple locations equally well, then BWA-MEM randomly allocates to one position (primary alignment). Several instances of multiple copy numbers of resistance genes (Line 215) occurred which will influence the

quantification of expression when aligned to the genome. Interestingly, there were some slight deviations in the expression of perfectly duplicated genes with unique flanking regions (refer to strA and sul1 in Figure 2A, contig 2 and 4) which may indicate that these genes are controlled by an operon (co-transcribed genes). This is an advantage of aligning to the genome. We also took this into consideration when graphing Figure 3 and combined all reads mapping to duplicated genes, such as strA, before normalizing to a housekeeping gene (rpsL).

L153: Why was a more well-known differential expression package not used here (e.g. DESeq2 or EdgeR) for evaluating differential expression? Is there an advantage of VGAM for plasmid or small genome differential expression?
Response: The beta-binomial distribution (implemented in VGAM) was used as a statistic to identify genes with significantly fewer or greater reads mapping in one sample versus another. It was chosen because it represents the uncertainty in the proportion estimated from count data. However, we agree that EdgeR and DESeq2 are also able to adequately estimate this uncertainty and hence we have redone the analysis using EdgeR (Supplementary Figure S7, Methods: "Whole transcriptome gene expression and estimation of expression confidence intervals", Line 157). The list of differentially expressed genes is very similar to that identified using VGAM (at least 90% identical).

L198 (see also L145): How identical were these genes? Would this identity affect genome mapping? In situations with multiple copies of near-identical genes, do you have any evidence to suggest that only one copy was active?
Response: These genes are 100% similar and will impact mapping to the genome. Unless expressed by an operon and the full-length sequences are retrieved, only then could this distinguish which genes are active. This issue will still arise if transcripts are mapped to the transcriptome. The only definitive way to determine this would be to perform knock-down studies of these regions and subsequently evaluate expression.

L218: What was the MAPQ probability for these genes? If the MAPQ probability is less than 3, it means that a gene could be equally-well placed at least two different sites (-log10(0.5) *10 ~= 3), which is expected given the gene duplication in your assemblies. I don't think this would indicate that the mapping is bad, as such, although there may be other reasons for a poor mapping.
Response: Agreed, the MAPQ score was commonly ≤10 for these duplicated reads. We have made a note of low mapping quality due to multiple copies of genes: "Low mapping quality could be attributed to assignment of reads to multiple copies of genes in the genome. Furthermore, the ONT error rates could lead to misassignment of reads to genes." (Line 275).

L228 (see also L198): more information about the similarity between the "correct" and "incorrect" gene would be useful; I notice that L335 mentions an identity for some genes of "greater than or equal to 80%". Do you have other evidence that systematic sequencing error would lead to reads being assigned to the incorrect gene?
Response: Various resistance genes harbor ≥80% similarity when taking into consideration genes deposited on the ResFinder database. In several instances, this is only 1 nucleotide and if sequencing errors arise, have the potential for misidentification. We can determine this accumulation of sequencing errors via observing the real-time emulation for DNA sequencing in Supplementary S5. After 5 hours (300 minutes), we could witness multiple genes being detected that were not identified in the final assembly and the Illumina only SPAdes assembly.

L245 (see also L218): Were there multiple fosA transcripts in the genome? I can't see from Table 1 any indication of this, but maybe it's not clear enough for me. If not, can you suggest other reasons for the low MAPQ score? It seems like a lot of results are being thrown away because the MAPQ is low.
Response: Only one copy of fosA is encoded on the chromosome for all isolates (Line 194). All genes with multiple copies have been noted in Line 215. The mapping quality is most likely due to the low expression of this gene and difficulties with base-calling (issues removing the long artificial poly(A) tail and interference of RNA modifications (Line 393). Once base-calling tools have been optimized for bacterial direct RNA sequencing, MAPQ scores will be a better quality.

L336 (see also L228 and L198): Would 80% identity lead to a misclassification by BWA-MEM?
Response: Yes, as some genes are very similar (potentially only one nucleotide difference), this has the potential to result in misclassification of resistance genes in the real-time emulation. Especially when we identified a 10% error rate in our ONT DNA sequencing (Line 356) and ≤23% for direct RNA sequencing (Line 394).

L341: I get a bit frustrated by people discussing accuracy from previous (typically quite old) nanopore papers as if it were a fixed thing, especially in a study that has produced a lot of other nanopore data. Nanopore technology changes quickly, and basecalling accuracy has made substantial improvements in particular over the last year. I'm not convinced a paper published in January 2018 would give a good estimate for accuracy called with guppy 3.0.3 (or 3.1.5, which is the latest that I'm aware of at the time of this review). Feel free to cite it, but I'd like to know [in the same breath] what the direct RNA accuracy was in *your* reads. L260-264 briefly discuss using different base-callers; how does that accuracy change depending on the base-caller?
Response: We have now included information regarding accuracy between base-callers: "Albacore 2.2.7 had the highest average accuracy across isolates (84.87%) closely followed by Guppy 3.0.3 (84.62%) and then Chiron v0.5 (78.19%) (Supplementary Table S6)." Line 279. The abstract also notes that we could identify accuracy up to 86% for direct RNA sequencing (Line 20).

** Figures **
Figure 1:
- Would work better as a side-by-side bar plot. The split graph makes it look like one side is negative, and the other side is positive.
- Order by colour / class rather tham abundance, with brackets indicating classifications.
Response: We initially considered side-by-side bar plots however, this would result in approximately 40 bars on the y-axis which is difficult to follow. We have now split the x-axis to better delineate between DNA and RNA data. Furthermore, an overlay of this data based on yield rather than time has been included in the supplementary results (Figure S4). The main text is written in the context of time to detect a particular gene conferring resistance to an antibiotic class, hence, why we ordered this as time of detection rather than grouping the antibiotic classes.

Figure 2:
- This figure is unclear to me. If this figure is relative expression (e.g. the statistic used for the correlation plot in Figure 3), then the presented data should be relative proportions, probably in log space (e.g. log2(gene/rpsL)).
- Why was rpsL chosen for normalisation?
Response: Unfortunately, the wrong figure legend was included for Figure 2 and has been amended. This data is counts per million (cpm) mapped reads rather than normalized to rpsL. We didn't adjust to relative proportions for this figure (or Figure 4, which is also in cpm) as the main text mentions cpm values. However, for comparisons of direct RNA to qRT-PCR (e.g. Figure 3 and Figure 5) we did normalize relative to housekeeping gene rpsL. This housekeeping gene has been used previously in literature (reference 46). We also have data for another housekeeping gene, rpoB, which generated similar results.

Figure 3:
- Were there any sample replicates? Are you able to estimate error in any measurements?
- The colour is confusing for this graph. You could try gene name for colour, and different plot symbols for different samples.
Response: All qRT-PCR measurements were done in triplicates (Line 170). There are no sample replicates for direct RNA sequence data. This is because the primary aim of the paper is to evaluate time-to-detection of antibiotic resistance genes across multiple samples (emulating a clinical setting in which a single replicate would be sequenced for each sample, particularly in the context of not having access to direct RNA multiplexing and so running a single sample in a single flow cell). However, we can estimate variation in the proportion of reads mapping to each gene (and hence the counts-per-million) by assuming the observed read counts are generated from a binomial distribution, so we can estimate a 90% CI in the expression levels using the conjugate beta prior. We show these estimates in Supplementary Figure S7.

Regarding the colours, there are 4 samples and eleven genes, so we didn't think colouring by gene would work (too many genes). We selected to colour by sample, and indicate the gene names on the plot. We have followed the suggestion of using different symbols per isolate.

Figure 4:
- What do the bottom panels describe (e.g. gene expression level scatter plots comparing each sample with each other sample)? This is not stated in the figure legend.
Response: Yes, the bottom panels include the expression levels between differing isolates in a scatter plot. This has now been added to the legend.

Figure 5:
- I recommend changing this to a side-by-side bar plot, as the text indicates that the comparison of A vs B is important.
Response: This figure has now been amended with the data on a single graph.

Reviewer #3:
The manuscript by Pitt et al interrogated the genome and transcriptome of PDR and XDR K. pneumoniae isolates using the Oxford Nanopore MinION device. This is the very first study which adopted nanopore approaches in direct bacterial mRNA sequencing. The authors established a methodology for adding poly(A) tail onto mRNA transcripts which will benefit future bacterial sequencing and diagnosis related studies. However, authors failed to explain clearly the advantage of using Nanopore for RNA sequencing to Illumina platform. In another word, why we need to develop RNA sequencing using Nanorpore since it is not an efficient way to do it and very complicated. In addition, the manuscript indeed showed that the coverage of RNA seq is very low and the correlation is not good. In my view, if there is no specific need to do RNA seq using Nanopore platform, there is no need to develop it since the Illumina platform is very good already in this application.
Response: Please refer to our first response to Reviewer #1.

In addition, I also have the following major comments:
1. Line 169, section "Antibiotic resistance and the location of acquired resistance in the genome "The authors reported the AMR genes and their location in this section. Since this is a technical manuscript, can the authors provide some sequencing information? The volume of data generated with time, coverage of each sequenced sample, the accuracy of the sequence, and the comparison of different assembly methods could be briefly discussed.
Response: We've now included additional information regarding the DNA sequencing: "MinION DNA sequencing for all isolates was run for ≥20 hours which generated 1.19 GB (215X) for 1_GR_13, 0.39 GB (67X) for 2_GR_12, 0.56 GB (101X) for 16_GR_13 and 0.64 GB (115X) for 20_GR_12 (Supplementary Table S2). Across the differing assembly tools, the chromosome sequence commonly circularised as a 5.0-5.4 Mb contig including plasmids ranging between 13-193 kb with the exception of 2_GR_12. Aligning ONT reads to the final assembly revealed that this DNA sequencing had a 90% accuracy rate across isolates." (Line 184) A comparison of several assembly methods is given in Supplementary Table 2, but we don't discuss this in much detail in the paper as it is not the focus of this work.

2. Line 256, only a low proportion of these RNA sequencing reads passed base-calling. Is it also related to the sample preparation apart from the inaccuracy of the base-calling software?
Response: Indeed, RNA sample preparation could influence the subsequent quality of the data and we attempted several protocol optimizations. We trialed phenol/ chloroform RNA extractions however, this process was lengthy and resulted in a low yield of RNA and increased impurities. The PureLink RNA Mini Kit protocol is relatively quick (<30 mins/ sample). We attempted an on-column DNase treatment during this protocol but the best DNA depletion was observed using TURBO DNase which doesn't work on column (requires 37°C incubation). Our optimized RNA extraction resulted in Bioanalyzer RNA integrity scores of ≥8.5 which has now been included in Line 116 (RIN scale 0-10, 10 is no degradation using 16S and 23S pecks as reference). Unfortunately, we were unaware of the efficiency of the polymerase until post sequencing analysis was performed (Supplementary Figure S6), hence, a shorter incubation can be implemented for future studies. However, the majority of inaccuracy appears to be due to the base-calling software unable to accurately trim the long artificial poly(A) tail and potential interference to the raw read signal via RNA modifications (Line 391).

3. Would the authors compare the genome and transcriptome a little bit to link these data?
Response: We have drawn various comparisons between the genome and transcriptome to link the sequencing data. In particular, tables and figures comparing both RNA and DNA include Figure 1, Table S5, Figure S3 and Figure S4 with corresponding sections in the main text. Additional information in the discussion has been provided to highlight the pros and cons regarding interpreting antibiotic resistance using either DNA or RNA. "We further investigated the transcriptome of these isolates to potentially elucidate the correlation between genotype and the subsequent resistant phenotype. Detection of antibiotic resistance via sequencing commonly uses DNA due to the instability of RNA and the lengthy sample processing such as rRNA depletion [12-15, 58]. However, RNA provides additional information regarding the functionality of genes such as identifying conditions in which a resistance gene is present but not active which gives rise to a false positive via DNA alone. Conversely, if expression is only induced in the presence of an antibiotic, the absence of RNA transcripts results in a false negative." (Line

367). “Furthermore, the time required to detect resistance may be hindered by the slower translocation speed associated with direct RNA sequencing (70 bases/ second) compared to DNA sequencing (450 bases/ second) [57]. Although cDNA would overcome this limitation, our findings show that detection was primarily due to level of expression when evaluating data yield rather than time.” (Line 394).

4. Line 381, "a number of resistance genes were identified that were not present in the final assembly. The authors were expected to discuss why this happens and how to deal with these false positive data.
Response: The discussion on this topic has now been extended: “Furthermore, a small number of resistance genes were identified that were not present in the final assembly, however these all had MAPQ values less than 10 and less than 30 mapped reads. Some of these may be due to low-level kit contamination, while some of the false positives have sequence similarity to true positives and may be due to inaccuracies in base-calling.” (Line 363).

Close
