# Supplementary material for: Evaluating the genome and resistome of extensively drug-resistant Klebsiella pneumoniae using native DNA and RNA Nanopore sequencing
Source: Gigascience. 2020 Feb 4;9(2):giaa002. doi: 10.1093/gigascience/giaa002 (PMC6998412; doi:10.1093/gigascience/giaa002)
Supplement: giaa002_Response_to_Reviewer_Comments_Revision_1 [file giaa002_response_to_reviewer_comments_revision_1.pdf]

Author's Response To Reviewer Comments

Close

“Evaluating the Genome and Resistome of Extensively Drug-Resistant Klebsiella pneumoniae using Native DNA and RNA Nanopore Sequencing”
GIGA-D-19-00200_R2
Response to Reviewers

Dear Dr. Scott Edmunds,

We thank the reviewers for the opportunity to implement additional revisions in our manuscript (GIGA-D-19-00200) which has enabled us to further clarify and strengthen this research via amending figures and conducting further analysis. Please find below a point-by-point response to the reviewer comments.

Reviewer reports:

Reviewer reports:
Reviewer #1: The authors' response to our comments and suggestions has improved this manuscript. We are generally satisfied with the manuscript edits and improvements; however, there are a few more modifications, particularly in the presentation of results, that we feel are required to make this paper acceptable for publishing.

1. The response to pt 1 - (why dRNA) is still largely unsubstantiated. The library prep time being cut in half is a slight advantage, but with cDNA we can start from less material (i.e. perform PCR). Multioperon sequencing could be shown from cDNA as easily as from dRNA - and the authors still haven't shown it. And the authors suggest RNA modifications could be detected, arguably the most unique advantage to dRNA seq in prokaryotes - but showed none of this. We recommend rewording rationale to avoid discouraging cDNA sequencing, especially since it is more practical for most applications at this point - and since the promised improvements for dRNA have yet failed to materialize from ONT, and cDNA yield is currently substantially better.
Response: We agree and have run additional analysis to detect operons (co-expression of genes) using BEDTools intersect which can be found in the results section: “Several resistance genes were identified to be regulated by operons and co-expression was evident for oqxAB (1_GR_13, 16_GR_13), blaVEB-1:ant(2”)-Ia:ARR-2 (1_GR_13), aadA1:sul1 (1_GR_13), rmtB:blaTEM-1B (1_GR_13, 2_GR_12, 16_GR_13), aph(6)-Id:strA (1_GR_13), sul2:aph(3”)-Ib:aph(6)-Id (2_GR_12, 16_GR_13), ant(2”)-Ia:blaVEB-1 (2_GR_12, 16_GR_13), aac(6’)-Ib-cr:blaOXA-1:catB4 (16_GR_13), aadA2:sul1 (16_GR_13) and sul2:aph(3”)-Ib:dfrA14 (20_GR_12) (Figure 2). Overall, various non rRNA genes were identified to be co-expressed (≥5 reads supporting gene intersect) across isolates (1_GR_13: 428; 2_GR_12: 310; 16_GR_13: 793; 20_GR_12: 442).” (Line 301-307). We have also uploaded the complete list of operons (including rRNA genes): “GIGA-D-19-00200_operons_+rRNA.xlsx”.
Unfortunately, there is currently no robust approach to detect RNA modifications using ONT direct RNA sequencing. The prior study by Garalde et al (2018) only interrogated one gene with a known modification and could compare to the same sequence lacking this modification (unmodified). The m6A and A-to-I editing modification can potentially be detected in recent studies (Workman RA et al (2019) https://doi.org/10.1038/s41592-019-0617-2. Liu H et al (2019) https://doi.org/10.1038/s41467-019-11713-9), however, these studies also had an unmodified dataset for comparison. Generation of the unmodified dataset usually requires in vitro transcribed RNA or modification sites can be determined using immunoprecipitation sequencing. These approaches can be costly and time consuming to generate on a whole transcriptome scale. As this dataset has not been generated on our isolates, we are unable to accurately call RNA modifications and hence, why we have not included this in our study. We have reworded the conclusion to highlight some shortcomings of ONT native RNA sequencing: “The expression of resistance genes was successfully detected in addition to identifying genes potentially regulated via operons, however, native RNA sequencing incurs a slower time to detect resistance due to translocation speed. Once base-calling algorithms have been optimised, this could allow for a whole transcriptome interrogation of the poorly characterised bacterial RNA modifications.” Line 423-427.

2. In the "levels of expression of resistance genes" section, lines 270-274, it would be useful to include read counts alongside percentages aligned to increase transparency for counts of reads included in the analysis.
Response: The read counts have now been included in this section. In some instances, a read range was used rather than all the individual values however, the full list of read counts can be found in the Supplementary material (Supplementary Table S6). Line 273-277.

3. In line 281 the authors state "These results reflect the fact that base-calling algorithms have not 282 yet been optimised for direct RNA sequencing, and even less so for bacterial RNA sequencing". However, the accuracies reported in the line above are not atypical of single molecule sequencing, and low alignment % seems largely driven by 400-700nt poly-A tails added- recommend amending this statement.
Response: This line has now been modified: "However, low alignment rates could be attributed to the addition of a long artificial poly(A) which was identified to be approximately 400 to 700 bp across isolates (Supplementary Figure S6)" Line 285-287.

4. More importantly, the alignment % is still alarmingly low - even with current RNA basecallers you should be seeing 80-90% alignment. We recommend filtering reads before alignment by only using "pass" reads, trimming poly-A tails off reads, and removing small reads less than 75nt (which are more likely to be noise), then reporting this alignment percentage.
Response: We now report the alignment rate on pass reads only after poly(A) trimming and removing the small reads less than 75nt, and find that the percentage is actually quite reasonable (≥98% alignment rate). These results have been included in the main text: "Aligning passed reads alone to the final assembly (ensuring trimming of poly(A) tail and removing reads <75 nt), ≥98% (1_GR_13: 95591; 2_GR_12: 138214; 16_GR_13: 227781; 20_GR_12: 119425) of reads were mappable, however, ≤46% (1_GR_13: 42654; 2_GR_12: 46787; 16_GR_13: 79175; 20_GR_12: 54986) of these had a MAPQ score ≥10." Line 274-277.

5. Figure 3- Please denote on the figure itself which primers recognize more than one gene, maybe with underlining? Also, what does the +0.001 notation on axes mean?
Response: Primers which recognize more than one gene have now been underlined in Figure 3. The data in this Figure has been log10 transformed hence, to show genes with no detectable expression, the data was shifted by +0.001. This has now been noted in the legend of Figure 3.

6. Figure 5 is overly complicated with the shapes and colors and asterisks - why not just plot the data in the same way as Figure 3, and you can facet by sample?
Response: Figure 5 has now been graphed similar to Figure 3.

Small things:
Spell out XDR first (line 329)
Response: Line 329 has now been amended. (Line 338).
Please parse paragraph lines 348-388 into DNA and RNA sections
Response: This section has now been modified to separate DNA and RNA. (Line 375).

Close
