# Supplementary material for: Evaluating the genome and resistome of extensively drug-resistant Klebsiella pneumoniae using native DNA and RNA Nanopore sequencing
Source: Gigascience. 2020 Feb 4;9(2):giaa002. doi: 10.1093/gigascience/giaa002 (PMC6998412; doi:10.1093/gigascience/giaa002)
Supplement: giaa002_Reviewer_1_Report_Original_Submission — Rachael Workman -- 6/25/2019 Reviewed [file giaa002_reviewer_1_report_original_submission.pdf]

Reviewer Report

Title: Evaluating the Genome and Resistome of Extensively Drug-Resistant Klebsiella pneumoniae using Native DNA and RNA Nanopore Sequencing

Version: Original Submission **Date:** 6/25/2019

Reviewer name: Rachael Workman

Reviewer Comments to Author:

In the manuscript "Evaluating the Genome and Resistome of Extensively Drug-Resistant Klebsiella pneumoniae using Native DNA and RNA Nanopore Sequencing" by Pitt et al., the authors describe datasets generated from multiple sequencing modalities of antibiotic-resistant clinical isolates, and discuss the potential of this technology for rapid detection of AMR. Although these methods and sequencing characterization and analysis are of importance to the field, there are several issues which remain to be addressed.
Specific points:
It would be useful to better establish the rationale for why direct detection of RNA transcripts matters, and what additional information direct RNA sequencing gets you that rapid cDNA conversion and sequencing can't. Perhaps the largest issue is - "Why dRNA-seq?" There doesn't seem to be an obvious benefit, given the poor time to detection compared to just DNA sequencing. Expression levels are useful, but could be determined from Illumina sequencing. Without splicing there are no isoforms to contend with, and the error rate adds difficulty in interpretation and determination of primary protein sequence.
Additionally, most clinical bacterial characterization work doesn't use RNA-seq, and addressing the problems clearly (i.e. rRNA depletion, RNA instability) should be done at the outset.
Under the "DNA extractions and HMW DNA isolation methods section", this section should be rewritten for clarity - it was confusing to determine which isolations worked and which didn't, and why. It's still important to include details of why protocol modifications were made, but if these could be incorporated into methods better that would aid in understanding.
Under "real-time resistome detection emulation" as well as "assembly of genomes" sections, it would be helpful to include a rationale on why certain software tools were chosen over others, given you tried many options. For example, why was BWA-MEM chosen over minimap2?
How were you able to distinguish multiple copies of resistance genes from duplicated misassemblies?
Would it actually be faster to detect with cDNA sequencing, given faster motor protein translocation rate and likely higher copy number of transcripts of interest? It would be useful to include thoughts on this in the discussion.
You say "Nanopore DNA sequencing currently has an accuracy ranging from 80 to 90%, which limits its ability to detect genomic variations", but there are post-processing tools available to increase accuracy and ability to detect SNVs - this should be included in the discussion.
Further the detection of SNV mutations and indels is critical with respect to the detection of chromosomal mutations in these samples. Additional consideration of methylation signatures is crucial,

as they can cause systematic error (PMID: 30373801) if not corrected.
"All isolates exhibited low levels of expression for fosfomycin, macrolide and tetracycline resistance, despite exhibiting phenotypic resistance to fosfomycin and tetracycline", but are high levels of expression essential for phenotypic resistance? Are these low levels surprising? It would be helpful to link to papers discussing this.
Figure 5 - instead of switching back and forth between panels A and B, a scatterplot comparing the two directly like Fig 3 would be more useful.
Why do you think only 23% RNA reads aligned? Did you try to identify the unaligned reads (like sort out contamination, noise)? It would be beneficial to include at least a blast/centrifuge style analysis trying to determine the source of the unaligned reads. Additionally, a k-mer analysis of the unaligned reads could help determine their origin.
How much of the poor alignment is due to the method of preparation (i.e. polyA tailing, etc.)? Did the authors perform optimization of the extraction and library prep for bacterial RNA? What about using an alternative tail and RNA adaptor?
Viral direct RNA seq has been done (PMID: 30765700 and 30258076 for example) - it would be good to cite these or related papers.
Some minor points:
"This research also established a methodology and analysis for bacterial direct RNA sequencing." is repeated in the conclusions.
Figure 2 colorblocking is a little confusing - could be more straightforward to break up the figure into separate panels per strain contig, for example with a ggplot facet_grid.

Methods

Are the methods appropriate to the aims of the study, are they well described, and are necessary controls included? Choose an item.

Conclusions

Are the conclusions adequately supported by the data shown? Choose an item.

Reporting Standards

Does the manuscript adhere to the journal's guidelines on minimum standards of reporting? Choose an item.

Choose an item.

Statistics

Are you able to assess all statistics in the manuscript, including the appropriateness of statistical tests used? Choose an item.

Quality of Written English

Please indicate the quality of language in the manuscript: Choose an item.

Declaration of Competing Interests

Please complete a declaration of competing interests, considering the following questions:

- Have you in the past five years received reimbursements, fees, funding, or salary from an organisation that may in any way gain or lose financially from the publication of this manuscript, either now or in the future?
- Do you hold any stocks or shares in an organisation that may in any way gain or lose financially from the publication of this manuscript, either now or in the future?
- Do you hold or are you currently applying for any patents relating to the content of the manuscript?
- Have you received reimbursements, fees, funding, or salary from an organization that holds or has applied for patents relating to the content of the manuscript?
- Do you have any other financial competing interests?
- Do you have any non-financial competing interests in relation to this paper?

If you can answer no to all of the above, write 'I declare that I have no competing interests' below. If your reply is yes to any, please give details below.

Rachael Workman and Winston Timp have received reimbursements for travel and speaking from Oxford Nanopore. Dr. Timp holds 2 patents (US2011/0226623 A1 and US2012/0040343 A1) which have been licensed by Oxford Nanopore Technologies.

I agree to the open peer review policy of the journal. I understand that my name will be included on my report to the authors and, if the manuscript is accepted for publication, my named report including any attachments I upload will be posted on the website along with the authors' responses. I agree for my report to be made available under an Open Access Creative Commons CC-BY license (http://creativecommons.org/licenses/by/4.0/). I understand that any comments which I do not wish to be included in my named report can be included as confidential comments to the editors, which will not be published.

Choose an item.

To further support our reviewers, we have joined with Publons, where you can gain additional credit to further highlight your hard work (see: https://publons.com/journal/530/gigascience). On publication of this paper, your review will be automatically added to Publons, you can then choose whether or not to claim your Publons credit. I understand this statement.

Yes Choose an item.
