# Supplementary material for: Evaluating the genome and resistome of extensively drug-resistant Klebsiella pneumoniae using native DNA and RNA Nanopore sequencing
Source: Gigascience. 2020 Feb 4;9(2):giaa002. doi: 10.1093/gigascience/giaa002 (PMC6998412; doi:10.1093/gigascience/giaa002)
Supplement: giaa002_Reviewer_1_Report_Revision_1 — Rachael Workman -- 11/6/2019 Reviewed [file giaa002_reviewer_1_report_revision_1.pdf]

Reviewer Report

Title: Evaluating the Genome and Resistome of Extensively Drug-Resistant Klebsiella pneumoniae using Native DNA and RNA Nanopore Sequencing

Version: Revision 1 **Date:** 11/6/2019

Reviewer name: Rachael Workman

Reviewer Comments to Author:

The authors' response to our comments and suggestions has improved this manuscript. We are generally satisfied with the manuscript edits and improvements; however, there are a few more modifications, particularly in the presentation of results, that we feel are required to make this paper acceptable for publishing.

1. The response to pt 1 - (why dRNA) is still largely unsubstantiated. The library prep time being cut in half is a slight advantage, but with cDNA we can start from less material (i.e. perform PCR). Multioperon sequencing could be shown from cDNA as easily as from dRNA - and the authors still haven't shown it. And the authors suggest RNA modifications could be detected, arguably the most unique advantage to dRNA seq in prokaryotes - but showed none of this. We recommend rewording rationale to avoid discouraging cDNA sequencing, especially since it is more practical for most applications at this point - and since the promised improvements for dRNA have yet failed to materialize from ONT, and cDNA yield is currently _substantially_ better.
2. In the "levels of expression of resistance genes" section, lines 270-274, it would be useful to include read counts alongside percentages aligned to increase transparency for counts of reads included in the analysis.
3. In line 281 the authors state "These results reflect the fact that base-calling algorithms have not 282 yet been optimised for direct RNA sequencing, and even less so for bacterial RNA sequencing". However, the accuracies reported in the line above are not atypical of single molecule sequencing, and low alignment % seems largely driven by 400-700nt poly-A tails added- recommend amending this statement.
4. More importantly, the alignment % is still alarmingly low - even with current RNA basecallers you should be seeing 80-90% alignment. We recommend filtering reads before alignment by only using "pass" reads, trimming poly-A tails off reads, and removing small reads less than 75nt (which are more likely to be noise), then reporting this alignment percentage.
5. Figure 3- Please denote on the figure itself which primers recognize more than one gene, maybe with underlining? Also, what does the +0.001 notation on axes mean?
6. Figure 5 is overly complicated with the shapes and colors and asterisks - why not just plot the data in the same way as Figure 3, and you can facet by sample?

Small things:

Spell out XDR first (line 329)

Please parse paragraph lines 348-388 into DNA and RNA sections

Methods

Are the methods appropriate to the aims of the study, are they well described, and are necessary controls included? Choose an item.

Conclusions

Are the conclusions adequately supported by the data shown? Choose an item.

Reporting Standards

Does the manuscript adhere to the journal's guidelines on minimum standards of reporting? Choose an item.

Choose an item.

Statistics

Are you able to assess all statistics in the manuscript, including the appropriateness of statistical tests used? Choose an item.

Quality of Written English

Please indicate the quality of language in the manuscript: Choose an item.

Declaration of Competing Interests

Please complete a declaration of competing interests, considering the following questions:

- Have you in the past five years received reimbursements, fees, funding, or salary from an organisation that may in any way gain or lose financially from the publication of this manuscript, either now or in the future?
- Do you hold any stocks or shares in an organisation that may in any way gain or lose financially from the publication of this manuscript, either now or in the future?
- Do you hold or are you currently applying for any patents relating to the content of the manuscript?
- Have you received reimbursements, fees, funding, or salary from an organization that holds or has applied for patents relating to the content of the manuscript?
- Do you have any other financial competing interests?
- Do you have any non-financial competing interests in relation to this paper?

If you can answer no to all of the above, write 'I declare that I have no competing interests' below. If your reply is yes to any, please give details below.

R.E.W. and W.T. received reimbursements for travel, accommodation and conference fees to speak at events organised by Oxford Nanopore. W.T. has two patents (8,748,091 and 8,394,584) licensed to ONT

I agree to the open peer review policy of the journal. I understand that my name will be included on my report to the authors and, if the manuscript is accepted for publication, my named report including any attachments I upload will be posted on the website along with the authors' responses. I agree for my report to be made available under an Open Access Creative Commons CC-BY license (http://creativecommons.org/licenses/by/4.0/). I understand that any comments which I do not wish to be included in my named report can be included as confidential comments to the editors, which will not be published.

Choose an item.

To further support our reviewers, we have joined with Publons, where you can gain additional credit to further highlight your hard work (see: https://publons.com/journal/530/gigascience). On publication of this paper, your review will be automatically added to Publons, you can then choose whether or not to claim your Publons credit. I understand this statement.

Yes Choose an item.
