# Supplementary material for: Evaluating the genome and resistome of extensively drug-resistant Klebsiella pneumoniae using native DNA and RNA Nanopore sequencing
Source: Gigascience. 2020 Feb 4;9(2):giaa002. doi: 10.1093/gigascience/giaa002 (PMC6998412; doi:10.1093/gigascience/giaa002)
Supplement: giaa002_Reviewer_2_Report_Original_Submission — David Eccles -- 7/1/2019 Reviewed [file giaa002_reviewer_2_report_original_submission.pdf]

Reviewer Report

Title: Evaluating the Genome and Resistome of Extensively Drug-Resistant Klebsiella pneumoniae using Native DNA and RNA Nanopore Sequencing

Version: Original Submission **Date:** 7/1/2019

Reviewer name: David Eccles

Reviewer Comments to Author:

This manuscript presents a rapid resistance-gene discovery experiment, using genome sequencing and assembly to identify potentially-active genes, combined with differential expression to determine drug-free resistome activity. This manuscript is differentiated from most other direct-RNA and cDNA nanopore research, in that it is the *expression* rather than the *structure* of the genes is evaluated here.
Bearing in mind that I cannot comment much on the biology side of things, I consider this manuscript to be a reasonable presentation of the experimental work that has been described, and recommend that it be accepted pending minor changes to figures, and clarification of multi-mapping results.
I would like to thank the authors for making their Nanopore sequence data public prior to review submission; it demonstrates a good open research ethic.
My specific comments regarding the manuscript follow:
** Text **
L133: This references a fairly old version of Canu (i.e. v1.5), which seems a bit strange given that Guppy v3.0.3 is also mentioned (L260). I note that Canu v1.8 was released before Guppy v3.0.3, and would be interested to know why this version of Canu was chosen.
L144: I don't have an encyclopaedic knowledge of bwa-mem command-line options. It would be helpful to explain what the options mean. I'm particularly interested in why the default options were not appropriate, and what (if any) compensations were made for multi-mapped reads.
L144: Why was minimap2 not used here? It was written by the same author as bwa-mem, but is specifically written to incorporate corrections to improve mapping for noisy Nanopore Direct RNA-seq [e.g. see https://github.com/lh3/minimap2#getting-started]
L145: I notice from L198 that there are gene copies in the data, with potentially high identity. Is there a particular reason why reads were mapped to the genome, rather than to transcriptome that merges

essentially-identical genes?
L153: Why was a more well-known differential expression package not used here (e.g. DESeq2 or EdgeR) for evaluating differential expression? Is there an advantage of VGAM for plasmid or small genome differential expression?
L198 (see also L145): How identical were these genes? Would this identity affect genome mapping? In situations with multiple copies of near-identical genes, do you have any evidence to suggest that only one copy was active?
L218: What was the MAPQ probability for these genes? If the MAPQ probability is less than 3, it means that a gene could be equally-well placed at at least two different sites (-log10(0.5) * 10 ~= 3), which is expected given the gene duplication in your assemblies. I don't think this would indicate that the mapping is bad, as such, although there may be other reasons for a poor mapping.
L228 (see also L198): more information about the similarity between the "correct" and "incorrect" gene would be useful; I notice that L335 mentions an identity for some genes of "greater than or equal to 80%". Do you have other evidence that systematic sequencing error would lead to reads being assigned to the incorrect gene?
L245 (see also L218): Were there multiple fosA transcripts in the genome? I can't see from Table 1 any indication of this, but maybe it's not clear enough for me. If not, can you suggest other reasons for the low MAPQ score? It seems like a lot of results are being thrown away because the MAPQ is low.
L336 (see also L228 and L198): Would 80% identity lead to a misclassification by BWA-MEM?
L341: I get a bit frustrated by people discussing accuracy from previous (typically quite old) nanopore papers as if it were a fixed thing, especially in a study that has produced a lot of other nanopore data. Nanopore technology changes quickly, and basecalling accuracy has made substantial improvements in particular over the last year. I'm not convinced a paper published in January 2018 would give a good estimate for accuracy called with guppy 3.0.3 (or 3.1.5, which is the latest that I'm aware of at the time of this review). Feel free to cite it, but I'd like to know [in the same breath] what the direct RNA accuracy was in *your* reads. L260-264 briefly discuss using different base-callers; how does that accuracy change depending on the base-caller?
** Figures **
Figure 1:

- Would work better as a side-by-side bar plot. The split graph makes it look like one side is negative, and the other side is positive.
- Order by colour / class rather tham abundance, with brackets indicating classifications.

Figure 2:

- This figure is unclear to me. If this figure is relative expression (e.g. the statistic used for the correlation plot in Figure 3), then the presented data should be relative proportions, probably in log space (e.g. log2(gene/rpsL)).
- Why was rpsL chosen for normalisation?

Figure 3:

- Were there any sample replicates? Are you able to estimate error in any measurements?
- The colour is confusing for this graph. You could try gene name for colour, and different plot symbols for different samples.

Figure 4:

- What do the bottom panels describe (e.g. gene expression level scatter plots comparing each sample with each other sample)? This is not stated in the figure legend.

Figure 5:

- I recommend changing this to a side-by-side bar plot, as the text indicates that the comparison of A vs B is important.

Methods

Are the methods appropriate to the aims of the study, are they well described, and are necessary controls included? Choose an item.

Conclusions

Are the conclusions adequately supported by the data shown? Choose an item.

Reporting Standards

Does the manuscript adhere to the journal's guidelines on minimum standards of reporting? Choose an item.

Choose an item.

Statistics

Are you able to assess all statistics in the manuscript, including the appropriateness of statistical tests used? Choose an item.

Quality of Written English

Please indicate the quality of language in the manuscript: Choose an item.

Declaration of Competing Interests

Please complete a declaration of competing interests, considering the following questions:

- Have you in the past five years received reimbursements, fees, funding, or salary from an organisation that may in any way gain or lose financially from the publication of this manuscript, either now or in the future?
- Do you hold any stocks or shares in an organisation that may in any way gain or lose financially from the publication of this manuscript, either now or in the future?
- Do you hold or are you currently applying for any patents relating to the content of the manuscript?
- Have you received reimbursements, fees, funding, or salary from an organization that holds or has applied for patents relating to the content of the manuscript?
- Do you have any other financial competing interests?
- Do you have any non-financial competing interests in relation to this paper?

If you can answer no to all of the above, write 'I declare that I have no competing interests' below. If your reply is yes to any, please give details below.

I have received free training, sequencing kits, and flow cells from Oxford Nanopore in return for my participation in the ONT community.

I agree to the open peer review policy of the journal. I understand that my name will be included on my report to the authors and, if the manuscript is accepted for publication, my named report including any attachments I upload will be posted on the website along with the authors' responses. I agree for my report to be made available under an Open Access Creative Commons CC-BY license (http://creativecommons.org/licenses/by/4.0/). I understand that any comments which I do not wish to be included in my named report can be included as confidential comments to the editors, which will not be published.

Choose an item.

To further support our reviewers, we have joined with Publons, where you can gain additional credit to further highlight your hard work (see: https://publons.com/journal/530/gigascience). On publication of this paper, your review will be automatically added to Publons, you can then choose whether or not to claim your Publons credit. I understand this statement.

Yes Choose an item.
