# Supplementary material for: Evaluating the genome and resistome of extensively drug-resistant Klebsiella pneumoniae using native DNA and RNA Nanopore sequencing
Source: Gigascience. 2020 Feb 4;9(2):giaa002. doi: 10.1093/gigascience/giaa002 (PMC6998412; doi:10.1093/gigascience/giaa002)
Supplement: giaa002_Reviewer_3_Report_Original_Submission — Sheng CHEN -- 7/13/2019 Reviewed [file giaa002_reviewer_3_report_original_submission.pdf]

Reviewer Report

Title: Evaluating the Genome and Resistome of Extensively Drug-Resistant Klebsiella pneumoniae using Native DNA and RNA Nanopore Sequencing

Version: Original Submission **Date:** 7/13/2019

Reviewer name: Sheng CHEN

Reviewer Comments to Author:

The manuscript by Pitt et al interrogated the genome and transcriptome of PDR and XDR K. pneumoniae isolates using the Oxford Nanopore MinION device. This is the very first study which adopted nanopore approaches in direct bacterial mRNA sequencing. The authors established a methodology for adding poly(A) tail onto mRNA transcripts which will benefit future bacterial sequencing and diagnosis related studies. However, authors failed to explain clearly the advantage of using Nanopore for RNA sequencing to Illumina platform. In another word, why we need to develop RNA sequencing using Nanorpore since it is not an efficient way to do it and very complicated. In addition, the manuscript indeed showed that the coverage of RNA seq is very low and the correlation is not good. In my view, if there is no specific need to do RNA seq using Nanoproe platform, there is no need to develop it since the Illumina platform is very good already in this application. In addition, I also have the following major comments:

1. Line 169, section "Antibiotic resistance and the location of acquired resistance in the genome"
The authors reported the AMR genes and their location in this section. Since this is a technical manuscript, can the authors provide some sequencing information? The volume of data generated with time, coverage of each sequenced sample, the accuracy of the sequence, and the comparison of different assembly methods could be briefly discussed.
2. Line 256, only a low proportion of these RNA sequencing reads passed base-calling. Is it also related to the sample preparation apart from the inaccuracy of the base-calling software?
3. Would the authors compare the genome and transcriptome a little bit to link these data?
4. Line 381, "a number of resistance genes were identified that were not present in the final assembly. "The authors were expected to discuss why this happens and how to deal with these false positive data.

Methods

Are the methods appropriate to the aims of the study, are they well described, and are necessary controls included? Choose an item.

Conclusions

Are the conclusions adequately supported by the data shown? Choose an item.

Reporting Standards

Does the manuscript adhere to the journal's guidelines on [minimum standards of reporting?] Choose an item.

Choose an item.

Statistics

Are you able to assess all statistics in the manuscript, including the appropriateness of statistical tests used? Choose an item.

Quality of Written English

Please indicate the quality of language in the manuscript: Choose an item.

Declaration of Competing Interests

Please complete a declaration of competing interests, considering the following questions:

- Have you in the past five years received reimbursements, fees, funding, or salary from an organisation that may in any way gain or lose financially from the publication of this manuscript, either now or in the future?
- Do you hold any stocks or shares in an organisation that may in any way gain or lose financially from the publication of this manuscript, either now or in the future?
- Do you hold or are you currently applying for any patents relating to the content of the manuscript?
- Have you received reimbursements, fees, funding, or salary from an organization that holds or has applied for patents relating to the content of the manuscript?
- Do you have any other financial competing interests?
- Do you have any non-financial competing interests in relation to this paper?

If you can answer no to all of the above, write 'I declare that I have no competing interests' below. If your reply is yes to any, please give details below.

I declare that I have no competing interests

I agree to the open peer review policy of the journal. I understand that my name will be included on my report to the authors and, if the manuscript is accepted for publication, my named report including any attachments I upload will be posted on the website along with the authors' responses. I agree for my report to be made available under an Open Access Creative Commons CC-BY license (http://creativecommons.org/licenses/by/4.0/). I understand that any comments which I do not wish to be included in my named report can be included as confidential comments to the editors, which will not be published.

Choose an item.

To further support our reviewers, we have joined with Publons, where you can gain additional credit to further highlight your hard work (see: https://publons.com/journal/530/gigascience). On publication of this paper, your review will be automatically added to Publons, you can then choose whether or not to claim your Publons credit. I understand this statement.

Yes Choose an item.
